# Targeting the FtsZ Allosteric Binding Site with a Novel Fluorescence Polarization Screen, Cytological and Structural Approaches for Antibacterial Discovery

**DOI:** 10.1021/acs.jmedchem.0c02207

**Published:** 2021-04-28

**Authors:** Sonia Huecas, Lidia Araújo-Bazán, Federico M. Ruiz, Laura B. Ruiz-Ávila, R. Fernando Martínez, Andrea Escobar-Peña, Marta Artola, Henar Vázquez-Villa, Mar Martín-Fontecha, Carlos Fernández-Tornero, María L. López-Rodríguez, José M. Andreu

**Affiliations:** † Centro de Investigaciones Biológicas Margarita Salas, CSIC, Ramiro de Maeztu 9, 28040 Madrid, Spain; ‡ Dept. Química Orgánica, Facultad de Ciencias Químicas, UCM, Avda. Complutense s/n, 28040 Madrid, Spain

## Abstract

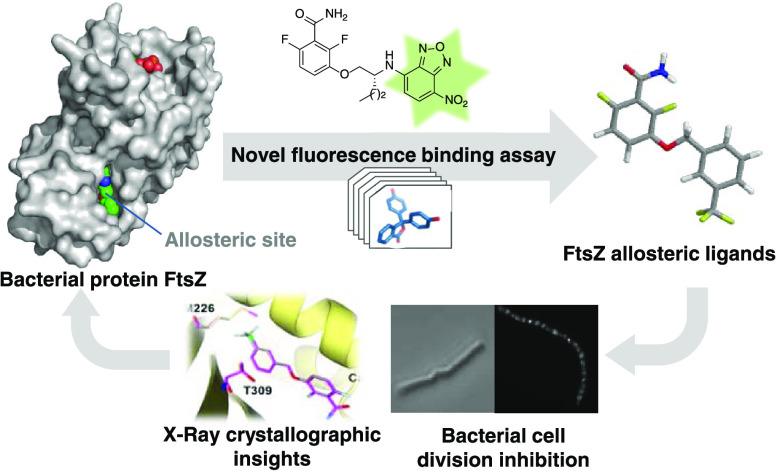

Bacterial resistance to antibiotics makes previously manageable infections again disabling and lethal, highlighting the need for new antibacterial strategies. In this regard, inhibition of the bacterial division process by targeting key protein FtsZ has been recognized as an attractive approach for discovering new antibiotics. Binding of small molecules to the cleft between the N-terminal guanosine triphosphate (GTP)-binding and the C-terminal subdomains allosterically impairs the FtsZ function, eventually inhibiting bacterial division. Nonetheless, the lack of appropriate chemical tools to develop a binding screen against this site has hampered the discovery of FtsZ antibacterial inhibitors. Herein, we describe the first competitive binding assay to identify FtsZ allosteric ligands interacting with the interdomain cleft, based on the use of specific high-affinity fluorescent probes. This novel assay, together with phenotypic profiling and X-ray crystallographic insights, enables the identification and characterization of FtsZ inhibitors of bacterial division aiming at the discovery of more effective antibacterials.

## Introduction

**Figure 1:**
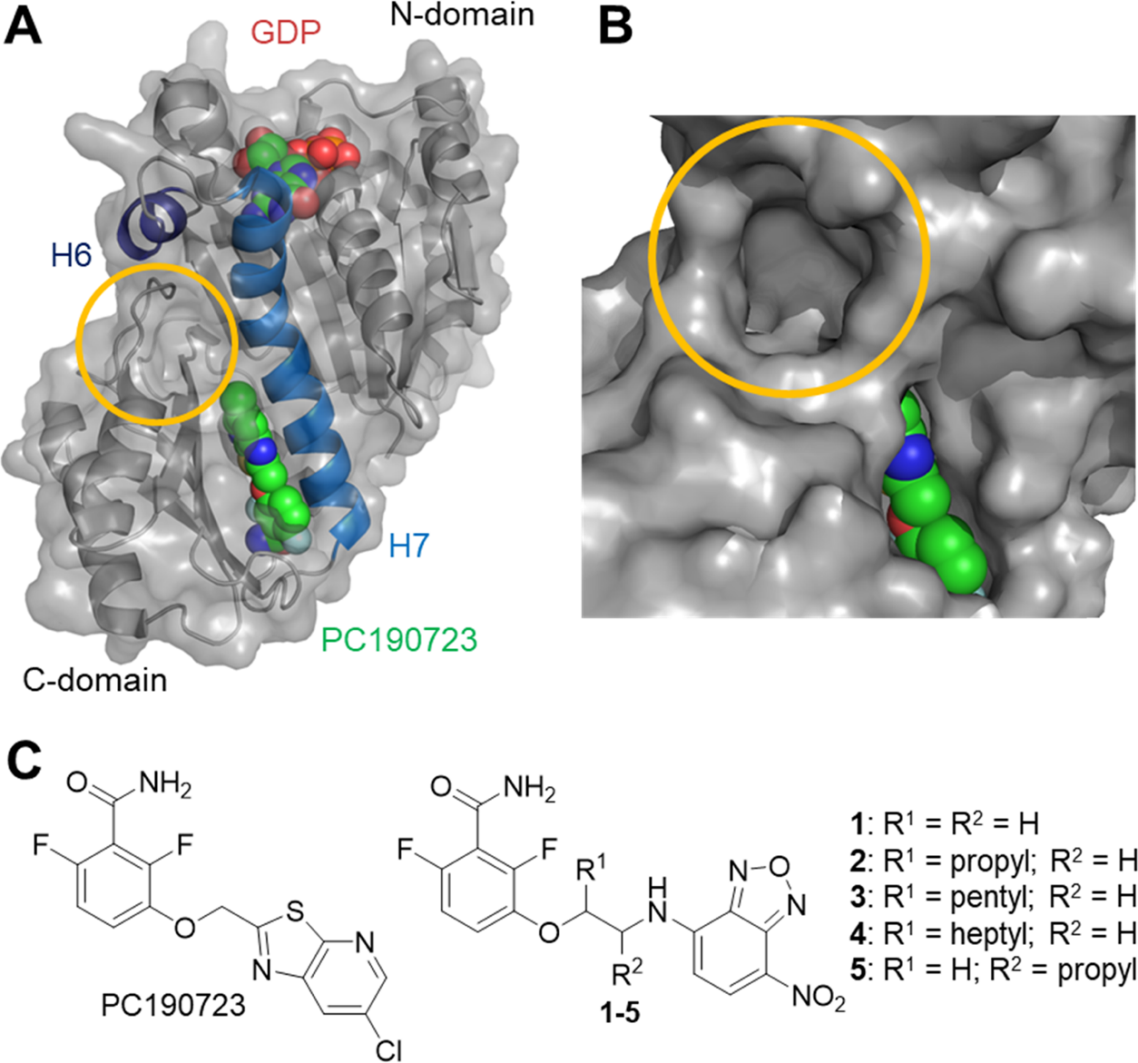
Fluorescent probes designed for the allosteric binding site of FtsZ. (A) Representation of the core structure of *S. aureus* FtsZ (SaFtsZ) (PDB entry 4DXD), showing the nucleotide (red-green spheres) in its binding pocket and PC190723 (green-blue-red spheres) bound into a cleft between the N-terminal and C-terminal domains. The yellow circle marks a cavity above PC190723 potentially available for ligand binding and the positions of helices H6 and H7 within the structure are highlighted. (B) Zoom of the encircled surface. (C) Chemical structures of PC190723, compound **1**,^26^ and the fluorescent probes **2**–**5** employed in this work.

New antibiotics are urgently needed to cope with the global rise of bacterial pathogens resistant to antibiotics in use, which renders lethal infections that once were treatable and controllable.^1−4^ To discover new antibiotics, processes essential for bacterial reproduction and spreading must be targeted, such as bacterial cell division, a clinically unexplored target. While eukaryotic mitosis has been vastly exploited for cancer treatments, targeting the bacterial cell division remains a clinical challenge. In this context, considerable knowledge has been gathered on the function, structure, dynamics, and interacting partners of essential cell division protein, FtsZ, since its localization forming a ring at the division site was discovered.^5^ The FtsZ ring orchestrates the assembly of the divisomal machinery in most bacteria.^6^ It is formed by FtsZ clusters that undergo treadmilling and guide cell envelope invagination at the division site.^7−9^ Bacterial division and FtsZ have been recognized as attractive targets for discovering new antibiotics.^10−12^ However, for many inhibitors reported in the literature, FtsZ targeting has not been really evidenced, slowing the progress in FtsZ-inhibitor development.^13^ FtsZ was validated as an antibacterial target of the potent antistaphylococcal experimental inhibitor PC190723 (Figure 1),^14^ which is synergistic with β-lactams.^2,15^ However, in spite of considerable synthetic efforts, the benzamide derivative PC190723, many analogues, and other inhibitors,^16^ have not made it into the clinic, possibly due to unsuitable pharmacological properties and a relatively high frequency of resistance mutations. Only one prodrug analogue of PC190723, TXA709^17^ (Chart S1), was designated as a qualified infectious disease product for *Staphylococcus aureus* infections and has recently completed a phase I clinical trial.^18^

FtsZ filaments form by head to tail self-association of FtsZ monomers; this is followed by hydrolysis of FtsZ-bound guanosine triphosphate (GTP) that triggers monomer dissociation. Cooperative assembly of single-stranded FtsZ filaments is made possible by FtsZ monomers switching between a low and a high association affinity state during the FtsZ filament assembly.^19−21^ In relaxed unassembled FtsZ monomers from several species, a side cleft between the N-terminal GTP-binding and the C-terminal subdomains is closed, whereas this cleft opens in tense FtsZ monomers from *S. aureus* (SaFtsZ) forming crystal filaments,^22^ providing a binding site for allosteric modulators of FtsZ assembly. Indeed, PC190723 acts as an allosteric inhibitor that stabilizes FtsZ filaments against disassembly upon GTP hydrolysis and selectively inhibits cell division in *Bacillus* and *Staphylococcus* species.^23,24^ PC190723 fits snugly in a flat conformation into its binding site at the bottom part of the open cleft in SaFtsZ crystals,^15,25^ leaving apparently little possibility for chemical modification. However, there is an unfilled pocket above connected by a tunnel to PC190723 (encircled in Figure 1A,B) and also a broader cavity around this pocket, formed by helices H6 and H7 and the C-terminal subdomain. These cavities are known since the first FtsZ structure was described^26^ but lack cognate ligands. Thus, there appears to be room for other ligands binding into the interdomain cleft in addition to PC190723.

We developed fluorescent probes for the interdomain cleft of FtsZ, in which a small fluorophore replaces the chloro-thiazolopyridine tail of inhibitor PC190723. Among them, 4-chloro-7-nitro-2,1,3-benzoxadiazole (NBD) probe **1** (Figure 1C) was employed to demonstrate the FtsZ assembly switch in solution, in which the side cleft between the N- and C-terminal domains, containing the benzamide binding site, opens in assembled FtsZ subunits and closes in unassembled monomers.^27^ The missing crystal structures of closed, relaxed SaFtsZ monomers were then determined,^28,29^ supporting an equilibrium between the open and closed-cleft conformers, relaxing to closed by the dissociation of tight longitudinal contacts in SaFtsZ filaments.^30^ The FtsZ polymerization-induced assembly switch enables the filament treadmilling displacement^29^ that is required for the FtsZ function in cell division, underscoring the importance of targeting the interdomain cleft to inhibit FtsZ filament dynamics. Mechanistic and structural studies together with complementary cytological profiling methods could allow the characterization of effective FtsZ inhibitors.^31,32^ Nonetheless, the lack of appropriate chemical tools to develop a binding screen against this site has hampered the discovery of new FtsZ allosteric inhibitors.

**Figure 2:**
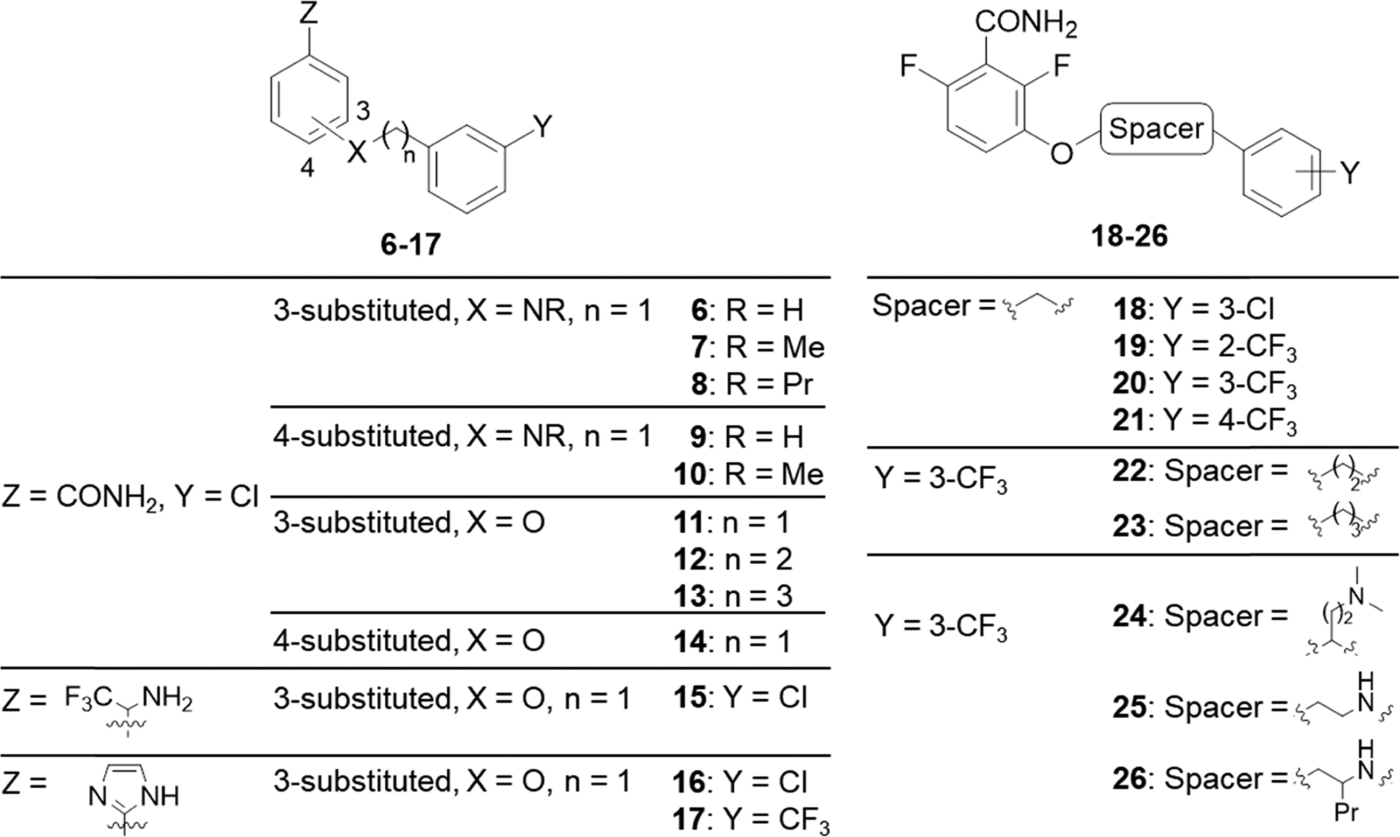
Chemical structures of compounds **6**–**26** evaluated as FtsZ inhibitors in this work.

In this work, we describe the first competitive binding assay for screening FtsZ ligands based on the use of new high-affinity fluorescent probes **2** and **5** (Figure 1C). Complementary phenotypic assays and X-ray crystallography have allowed the validation of this screen for the identification of FtsZ allosteric inhibitors. Hence, a series of structurally simplified benzamide analogues **6**–**26** (Figure 2) have been synthesized and evaluated in this new assay, identifying FtsZ-targeting inhibitors of bacterial division. Altogether, the biological and structural studies reported in this work provide relevant information about the molecular recognition of FtsZ allosteric ligands, which may contribute to the discovery of more effective antibacterial agents.

## Chemistry

**Scheme 1:**
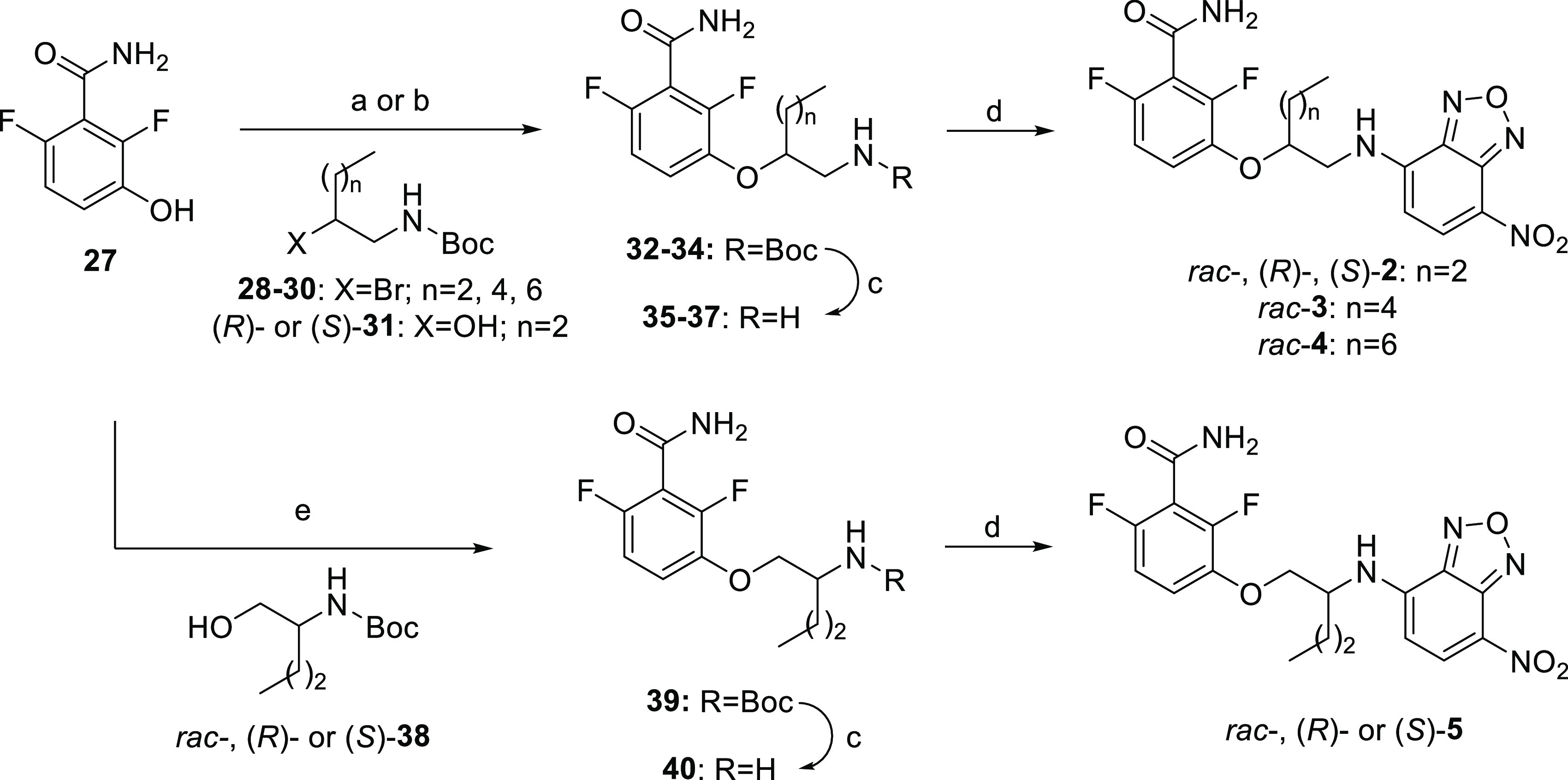
Synthesis of Compounds **2**−**5** Reagents and conditions: (a) **28**–**30**, K_2_CO_3_, NaI, dimethylformamide (DMF), rt, 24 h, 14–23%; (b) (*R*)- or (*S*)-**31**, diisopropyl azodicarboxylate (DIAD), PBu_3_, DMF, MW, 150 °C, 2 h, 28–31%; (c) trifluoroacetic acid (TFA), dichloromethane (DCM), rt, 1 h, 70–90%; (d) NBD-Cl, Cs_2_CO_3_, MeCN, 80 °C, 1 h, 7–50%; and (e) *rac*-, (*R*)-, or (*S*)-**38**, DIAD, PBu_3_, DMF, 80 °C, 48 h, 18–19%.

The synthesis of fluorescent probes **2**–**5** is outlined in Scheme 1. Racemic probes **2**–**4** were prepared starting from 2,6-difluoro-3-hydroxybenzamide (**27**), which was O-alkylated with the appropriate *N*-Boc-2-bromoalkylamine **28**–**30**, followed by the removal of the Boc group by treatment with trifluoroacetic acid and coupling of the resulting intermediates **35**–**37** with NBD chloride. For the synthesis of (*R*)- and (*S*)-**2**, the Mitsunobu reaction under microwave (MW) irradiation between benzamide **27** and the corresponding enantiomeric form of *N*-Boc-aminoalcohol **31**, subsequent deprotection of the amino group, and reaction with NBD chloride afforded the desired enantioenriched probes. Racemic probe **5** and its enantiomers were obtained by applying the same sequence of Mitsunobu reaction, deprotection, and NBD-coupling but using the appropriate form of primary alcohol **38**.

**Scheme 2:**
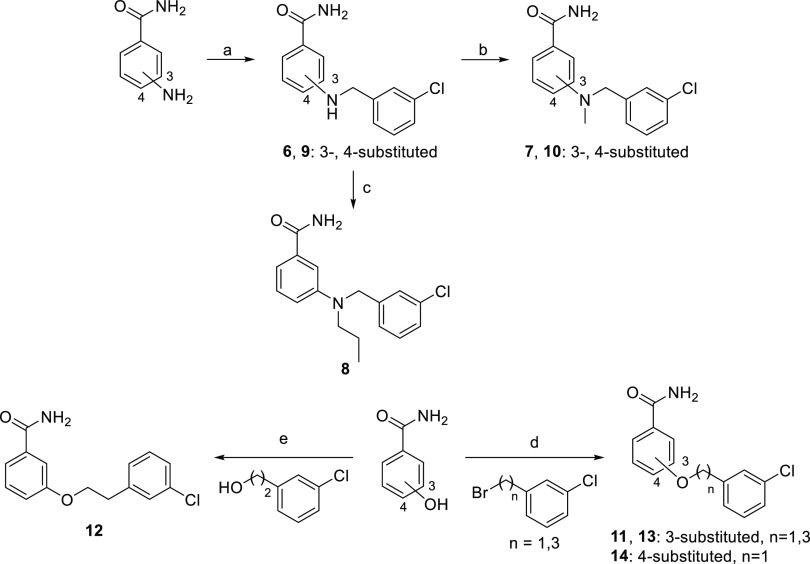
Synthesis of Compounds **6**−**14** Reagents and conditions: (a) (i) 3-chlorobenzaldehyde, MW, 100 °C, 10 min; (ii) H_2_, Pd/C, MeOH, rt, 18 h, 32–74%; (b) MeI, K_2_CO_3_, DMF, 60 °C, 18 h, 31–35%; (c) (i) CH_3_CH_2_CHO, MeOH, rt, 18 h; (ii) NaBH_3_CN, MeOH, rt, 72 h, 52%; (d) K_2_CO_3_, NaI, DMF, rt, 24 h, 47–80%; and (e) PPh_3_, diethyl azodicarboxylate (DEAD), Et_3_N, tetrahydrofuran (THF), rt, 24 h, 46%.

Regarding the synthesis of compounds **6**–**14** (Scheme 2), derivatives **6** and **9** were prepared by reductive amination of 3-chlorobenzaldehyde with 3- and 4-aminobenzamide, respectively. *N*-Methyl analogues **7** and **10** were synthesized by alkylation of regioisomers **6** and **9** with iodomethane, whereas reductive amination between **6** and propionaldehyde provided *N*-propyl derivative **8**. For the synthesis of *O*-(alkylphenyl)benzamides **11**–**14**, 3- or 4-hydroxybenzamide was alkylated with the proper bromoalkylaryl derivative under classical Williamson conditions (**11**, **13**, and **14**) or with 2-(3-chlorophenyl)ethanol in a Mitsunobu reaction to obtain compound **12** (Scheme 2).

**Scheme 3:**
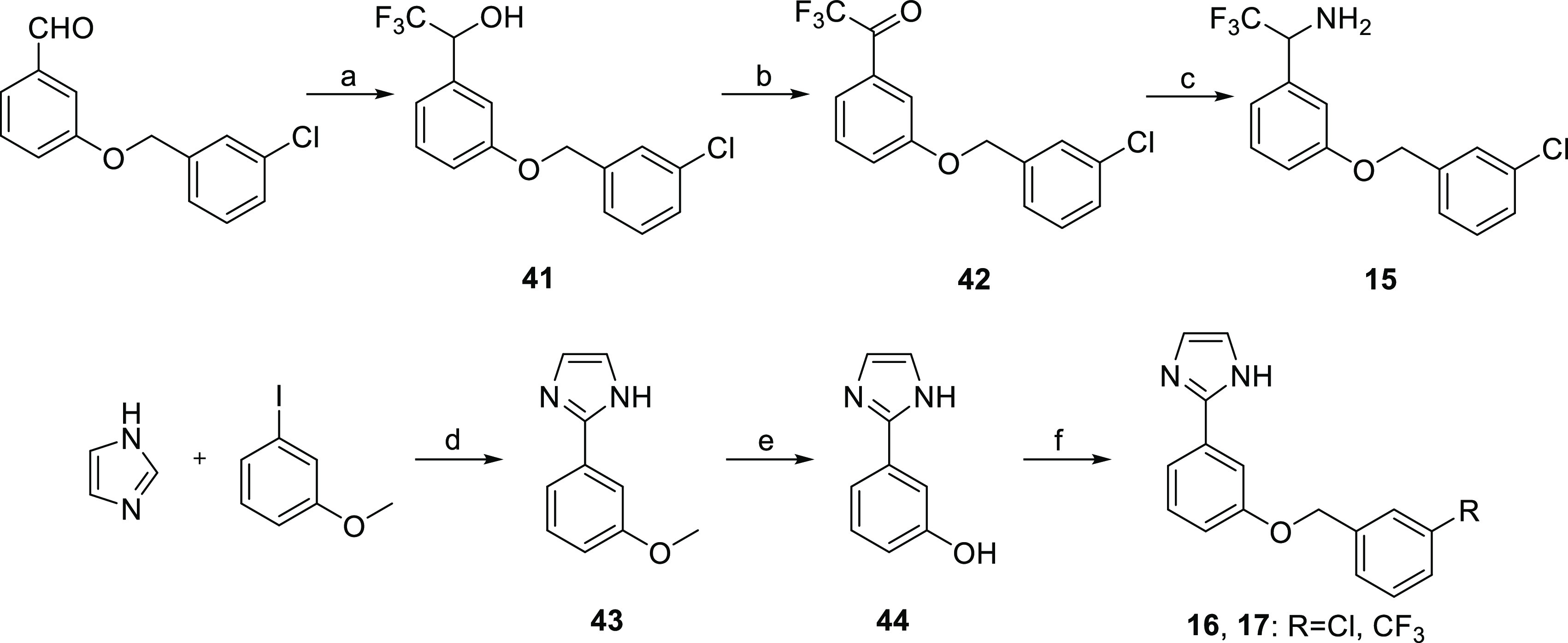
Synthesis of Compounds **15**−**17** Reagents and conditions: (a) (i) TMSCF_3_, K_2_CO_3_, DMF, rt, 48 h; (ii) 2 M HCl, rt, 4 h, 87%; (b) Dess–Martin periodinane, DCM, rt, 18 h, 99%; (c) (i) (CH_3_)_3_CSONH_2_, Ti(OiPr)_4_, Et_2_O, reflux, 24 h; (ii) NaBH_4_, Et_2_O, rt, 24 h; (iii) 4 M HCl, 1,4-dioxane, rt, 1 h, 24%; (d) Pd(OAc)_2_, CuI, DMF, MW, 200 °C, 40 min, 53%; (e) BBr_3_, DCM, 0 °C to rt, 24 h, 95%; and (f) 3-chlorobenzyl bromide or 3-(trifluoromethyl)benzyl bromide, K_2_CO_3_, NaI, DMF, rt, 24 h, 72–75%.

Benzamide bioisosteres **15**–**17** were synthesized as depicted in Scheme 3. The reaction of 3-[(3-chlorobenzyl)oxy]benzaldehyde with trimethyl(trifluoromethyl)silane afforded trifluoromethyl alcohol **41**, which was oxidized to ketone **42** with the Dess–Martin reagent, followed by reductive amination using *tert*-butanesulfinamide as an ammonia equivalent to provide target compound **15**. Imidazole derivatives **16** and **17** were prepared by the Pd- and Cu-mediated cross-coupling reaction of imidazole and 3-iodoanisole, subsequent demethylation with boron tribromide of **43**, and O-benzylation of phenol **44** with the corresponding chloro- or trifluoromethyl-substituted benzyl bromide.

**Scheme 4:**
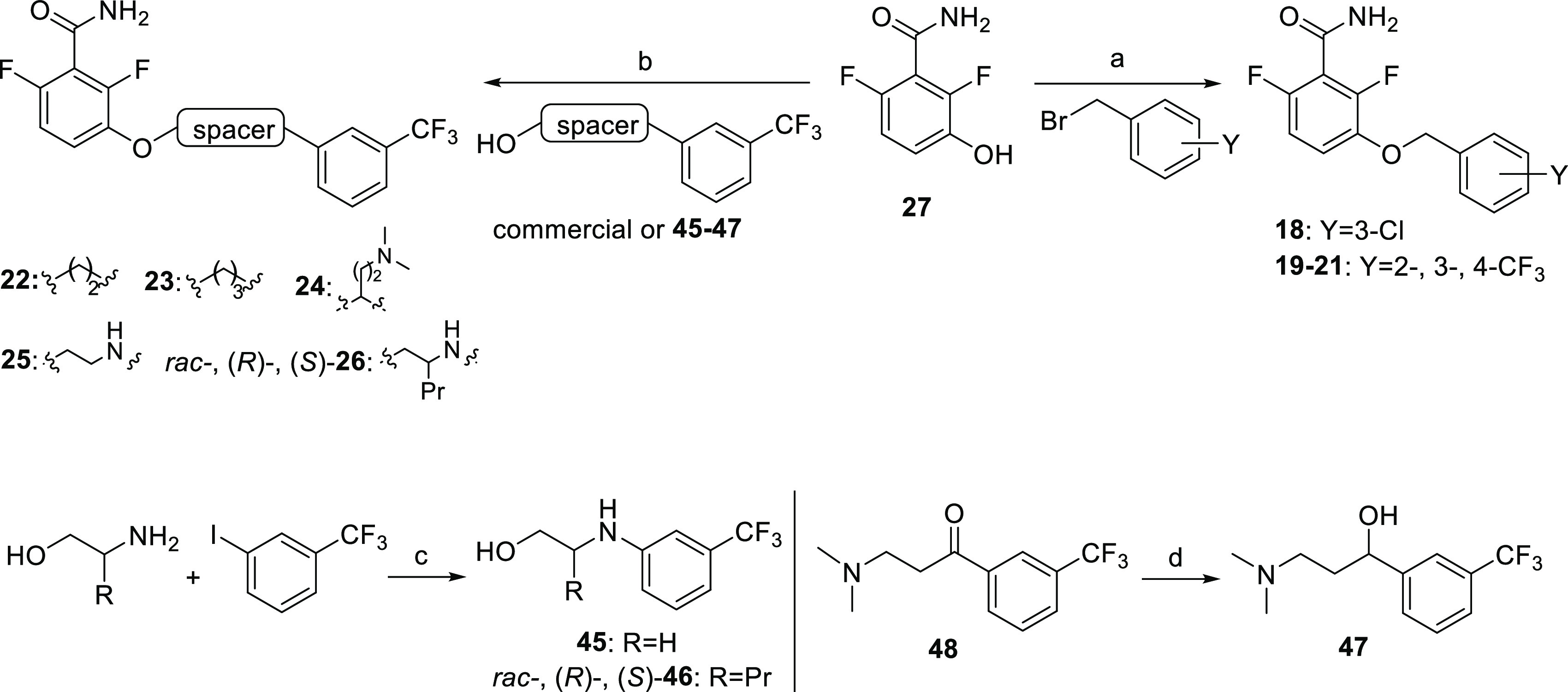
Synthesis of Compounds **18**−**26** Reagents and conditions: (a) K_2_CO_3_, NaI, DMF, rt, 24 h, 81–98%; (b) PBu_3_, DIAD, DMF, MW, 150 °C, 1.5 h, 45–50%; (c) K_3_PO_4_, CuI, (CH_2_OH)_2_, *i*-PrOH, MW, 150 °C, 105 min, 46–56%; and (d) LiAlH_4_, THF, rt, 2 h, 76%.

2,6-Difluorobenzamides **18**–**26** were synthesized starting from benzamide **27** (Scheme 4). Direct alkylation of **27** with the adequately substituted benzyl bromide under basic conditions afforded compounds **18**–**21** and Mitsunobu reaction of **27** with commercial or synthesized alcohols **45**–**47** gave analogues **22**–**26**. Primary alcohols **45** and **46** were prepared by the Ullman-type reaction between 1-iodo-3-(trifluoromethyl)benzene and 2-aminoethanol or 2-aminopentanol, respectively. Secondary alcohol **47** was obtained by the reduction of ketone **48** with lithium aluminum hydride.

## Results and Discussion

### Enhanced Affinity Fluorescent Probes for the Interdomain Cleft of FtsZ

**Figure 3:**
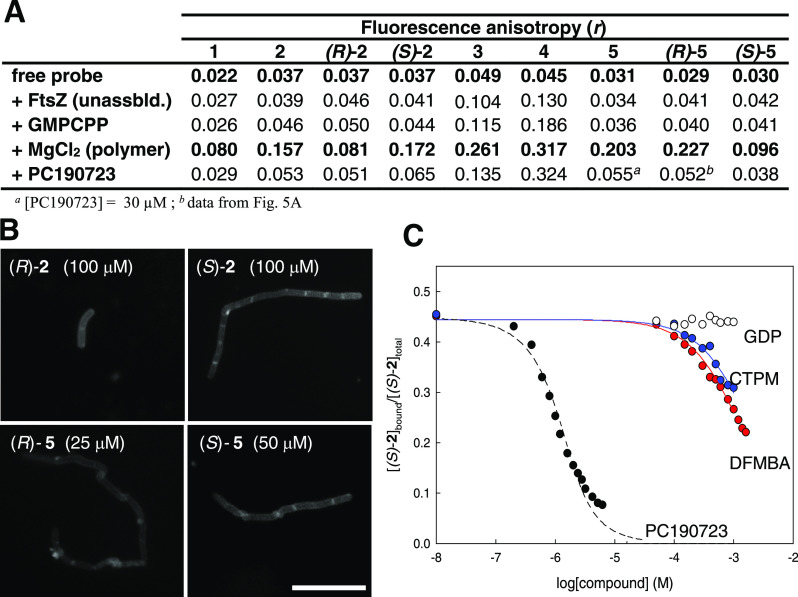
Specific binding of fluorescent probes to FtsZ′s allosteric site, activity on bacterial cells, and competitive binding assay for ligands binding to the site. (A) Fluorescence anisotropy values of the free probes (10 μM) in buffer, to which FtsZ (10 μM, unassembled) was added, followed by the slowly hydrolyzable GTP analogue GMPCPP (0.1 mM). BsFtsZ polymerization was then induced by the addition of MgCl_2_ (10 mM) and anisotropy values were recorded 5–10 min later. Nonfluorescent PC190723 (10 μM) was subsequently added to displace the fluorescent probe and anisotropy was measured 5 min later. (B) Fluorescence microscopy images of *B. subtilis* cells treated for 1.5 h with the minimal division inhibitory concentrations (MDC) of (*S*)-**2** and each enantiomer of **5** (Table S1), acquired employing the probe fluorescence. (C) Displacement curves of (*S*)-**2** (3 μM) from stabilized FtsZ-GMPCPP polymers (8 μM binding sites) by PC190723 (black circles, *K*_D_ < 0.1 μM), and its moieties 2,6-difluoro-3-methoxybenzamide (DFMBA, *K*_D_ = 0.67 ± 0.05 mM) (red), and 6-(chloro[1,3]thiazolo[5,4-*b*]pyridin-2-yl)methanol (CTPM, *K*_D_ = 1.0 ± 0.1 mM) (blue). Solid lines are best fits to the data of a single site competition model (Experimental Section). The addition of guanosine diphosphate (GDP) (void circles) does not displace (*S*)-**2** from its binding site.

A key step in the development of a fluorescence-based binding assay targeting the FtsZ interdomain cleft is the generation of specific probes for this site. These tools should undergo a sensitive modification of their fluorescence properties when bound to the protein, which returns to those of the free probe when displaced by a reference ligand such as PC190723. Competition assays of this type require that the probe and the competitor have commensurate affinities, resulting in a limited measurement range of competitor affinities with a single probe.^33^ Thus, specific medium affinity probes are required for the effective detection of weak but specifically competing molecules during inhibitor screening, whereas high-affinity probes are needed for the measurement of the dissociation constant (*K*_D_) of high-affinity compounds. Our previous studies demonstrated that probe **1** (Figure 1C) and its analogues with longer spacers between the difluorobenzamide ring and the fluorophore specifically bind in a narrow affinity range (*K*_D_ = 11–26 μM) with moderate fluorescence anisotropy changes^27^ that were deemed insufficient for the development of a competitive assay. However, their biochemical effects, together with molecular dynamics simulations of model complexes, supported the notion that an extension of the PC190723 binding site is available for inhibitors binding into the FtsZ interdomain cleft.^27^ Thus, taking probe **1** as the starting point, we resorted to adding aliphatic side chains to the spacer to gain additional hydrophobic interactions by increasing the length of the aliphatic chain according to the space available in the allosteric interdomain cleft. These new NBD-based compounds **2**–**5** (Figure 1C and Scheme 1) were characterized by their fluorescence spectra (Figure S1), the anisotropy changes upon specific reversible binding to FtsZ polymers (Figure 3A), fluorescence microscopy of *Bacillus subtilis* cells (Figure 3B), and binding affinity titrations (Figure S2 and Table S1). FtsZ polymers were formed in the presence of the slowly hydrolyzable GTP analogue GMPCPP and magnesium. The four probes showed large anisotropy values in the presence of FtsZ polymers compared to the free probe (Figure 3A). However, the recovery of the initial anisotropy values after the addition of excess competing PC190723, supporting binding specificity, was only achieved in the case of probes **1**, **2**, and **5**. Compounds **3** and **4**, with the longer side chains, exhibited an increase in background anisotropy with unassembled FtsZ in the absence of magnesium that were deemed nonspecific. In addition, the anisotropy of **4** with FtsZ polymers in the presence of magnesium was not reduced by PC190723 competition (Figure 3A). We thus focused on specific propyl-substituted probes **2** and **5**, and both enantiomers of each compound were synthesized and evaluated. The *S* enantiomer of **2** [(*S*)-**2**] displayed enhanced affinity (*K*_D_ = 8 ± 1 μM; Table S1) and a large anisotropy change compared to **1**, whereas (*R*)-**2** had a very small fluorescence anisotropy increase (Figure 3A). Better results were obtained when the propyl side chain was incorporated at the carbon next to the amino group, and (*R*)-**5** resulted in the highest affinity probe (*K*_D_ = 1.9 ± 0.6 μM) and broadest anisotropy changes, whereas (*S*)-**5** presented lower affinity (*K*_D_ = 15 ± 2 μM) (Table S1 and Figure 3A). The replacement of the NBD by boron dipyrromethene (BODIPY) or acrylodan fluorophores, as well as the substitution of the oxygen of the alkoxybenzamide by nitrogen, resulted in smaller or nonspecific anisotropy changes (probes **SP1**–**SP5**Figure S1, synthesis in Scheme S1).

Treating *B. subtilis* cells with probes **2** and **5** (Figure 3B) revealed that (*S*)-**2** and both enantiomers of **5** (at 25–100 μM) induced the characteristic filamentous phenotype due to the inhibition of cell division, supporting FtsZ targeting.^31,32^ These probes faintly stained intracellular structures, similarly to **1**,^27^ whereas (*R*)-**2** was inactive in these assays. Interestingly, the higher affinity probe (*R*)-**5** showed the best efficacy in the impairment of cell division and displayed antibacterial activity in *B. subtilis* (Table S1). Altogether, cytological profiling and in vitro binding measurements were in qualitative agreement and support the suitability of fluorescent probes (*S*)-**2** and (*R*)-**5** for the development of a competitive binding assay.

### Competitive Fluorescent Method for Ligand Binding into the FtsZ Interdomain Cleft

Capitalizing on the anisotropy decrease of the fluorescent benzamide probes (*S*)-**2** and (*R*)-**5** upon dissociation, we designed homogeneous assays to specifically determine the binding of any molecule to the PC190723 binding site in FtsZ polymers. It should be noted that this assay is more challenging than the ones described for ligands binding into the nucleotide orthosteric binding site of FtsZ monomers.^34,35^ In this new method, stabilized FtsZ polymers are required that do not disassemble upon nucleotide hydrolysis or by the action of other FtsZ polymerization inhibitors binding elsewhere in the protein molecule, producing misleading anisotropy changes. Gentle cross-linking of the FtsZ-GMPCPP polymers with 0.15% (v/v) glutaraldehyde, adapted from microtubule-fluorescent taxoid studies,^36^ rendered stabilized FtsZ from *B. subtilis* (BsFtsZ) polymer preparations that were resistant to depolymerization by GDP addition but still capable of probe binding with the same affinity values as noncross-linked polymers (Figure S2 and Table S1). We then employed mixtures of stabilized FtsZ polymers and probe (*S*)-**2** in competition assays, measuring the decrease in fluorescence anisotropy upon displacement of the probe to determine binding at increasing concentrations of competing test ligands. As a proof of concept, Figure 3C shows the competition binding isotherms of the reference high-affinity ligand PC190723 and its weakly binding moieties DFMBA and CTPM. Importantly, when the potent polymerization inhibitor GDP was used as a negative control, no change in probe anisotropy was observed.

### Binding Screen for Allosteric FtsZ Inhibitors Combined with Cytological Methods

**Figure 4:**
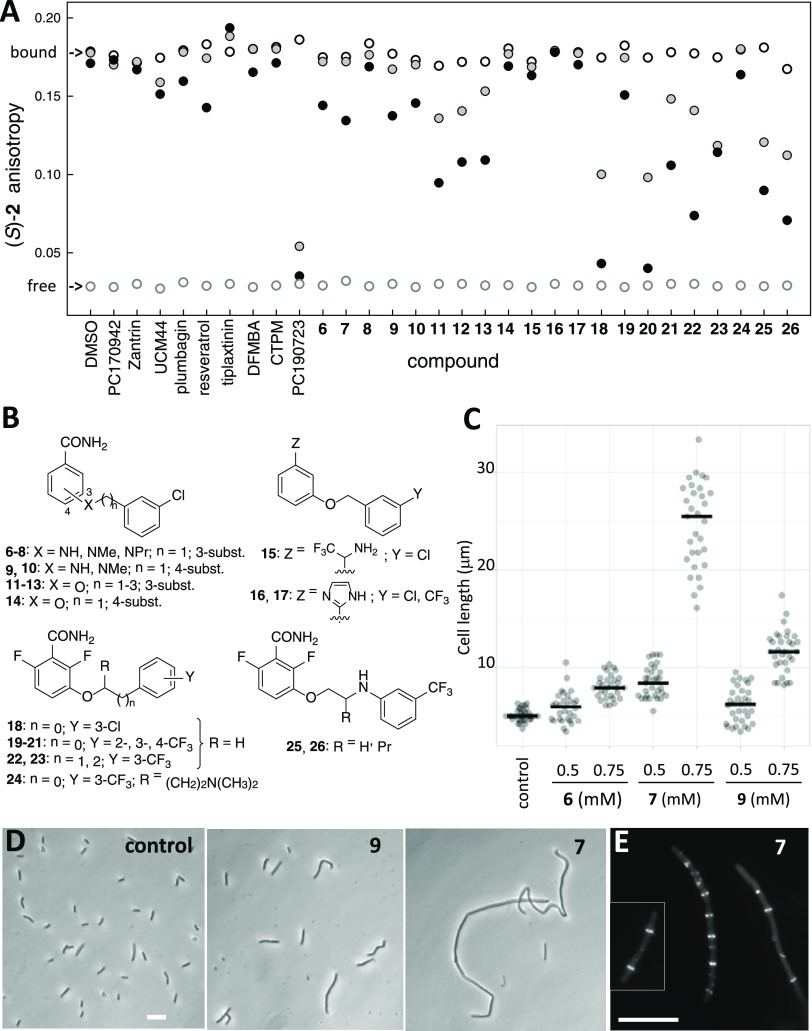
Fluorescence anisotropy screen for FtsZ allosteric inhibitors combined with cell-based methods. (A) Screen for FtsZ inhibitors employing the fluorescence anisotropy of probe (*S*)-**2** (3 μM) and stabilized FtsZ-GMPCPP polymers (8 μM binding sites). The anisotropy values of the partially bound probe (void black circles), the free probe (void gray circles), and the values with two concentrations of each compound, 20 μM (gray circles) and 200 μM (black circles) are shown. Control values with the corresponding concentrations of dimethyl sulfoxide (DMSO) vehicle (2% void and gray circles; 4%, black circle) are also shown. (B) Chemical structures of synthetic ligands **6**–**26**. (C) Effects of low-affinity inhibitors **6**, **7**, and **9** on *B. subtilis* 168 cell division. Raw data plots (*n* ≥ 30) are shown with the mean values marked (statistics in Table S2). The cells were incubated for 3 h with each compound, and cell lengths were measured. (D) Representative phase-contrast images of cells after 3 h with **7** or **9** (0.75 mM each). (E) Cellular localization of FtsZ-GFP in *B. subtilis* SU570 following growth for 1.5 h in the absence (inset) and presence of **7** (0.2 mM). Bars, 10 μm.

Once the fluorescence method for ligand binding into the interdomain cleft was set up, we evaluated its potential to screen for FtsZ allosteric inhibitors. Complementary to this screen, cytological profiling tests of the identified binders allow ascertaining their ability to target FtsZ in bacterial cells. Fluorescence anisotropy measurements of probe (*S*)-**2** at two concentrations of each tested compound (20 and 200 μM) were acquired; the solubility range of positively testing compounds was determined spectrophotometrically. Of note, the screen results can be displayed as raw anisotropy data, permitting to distinguish at a glance weak inhibitors from higher affinity candidates that markedly decrease the anisotropy values at both concentrations (Figure 4A). Among several previously described FtsZ inhibitors, two selective disruptors with an unknown binding site, PC170942^35,37^ and zantrin Z3,^38,39^ gave no evidence of binding in our assay, and the specific GTP-replacing FtsZ-inhibitor UCM44^35^ showed a marginal inhibitory effect (chemical structures in Chart S2). The promiscuous inhibitors resveratrol,^40^ plumbagin,^41^ and tiplaxtinin^42^ (Chart S2) weakly modified anisotropy and total fluorescence intensity, although none of them impaired *B. subtilis* cell division or GTP-FtsZ ring localization in our hands. It should be noticed that aggregating compounds may sequester the protein or the probe or may scatter highly polarized light, potentially interfering with the fluorescence anisotropy measurements. Colored and fluorescent compounds can also interfere. Compounds initially testing positive in the anisotropy screen should thus be checked for any unexpected changes of the total fluorescence intensity in the same samples, as well as for any artefactual effects on replicates omitting FtsZ.

Next, *N*- or *O*-(alkylphenyl)benzamides **6**–**14**, **18**–**26**, and amide bioisosteres **15**–**17** were evaluated as FtsZ allosteric inhibitors (Figures 2 and 4A,B). Different types of structural modifications around the three main structural features of benzamide inhibitors^16^ were explored: (i) linear or branched alkylenamino or alkylenoxy linkers of different lengths as spacers, similar to the recent structure–activity relationship studies;^43^ (ii) difluorination of the benzamide core; and (iii) the position and effects of substituents around the phenyl ring selected as the aromatic moiety. Starting with lower affinity compounds, 3- and 4-aminobenzamides **6**, **7**, **9**, and **10** were identified as weak binders (Figure 4A). Complementary competition measurements indicated *K*_D_ values around 0.2 mM in an affinity order of **7** > **9** ≥ **6** ≥ **10** (Figure S3). Interestingly, compound **7** with a methylamino linker, markedly inhibited *B. subtilis* cell division at 0.75 mM (Figure 4C), inducing a characteristic filamentous phenotype (Figure 4D), whereas its propyl-amino analogue **8** was toxic to the bacterial cells. In addition, compound **7** (0.2 mM) effectively induced irregularly spaced Z-rings and delocalization of GFP-FtsZ into punctate *foci* (Figure 4E), characteristic of alkoxybenzamide inhibitors.^14,44^

These results indicate that our fluorescence binding screen method combined with phenotypic tests can effectively detect relatively weak FtsZ-targeting compounds, which may lead to more effective cell division inhibitors. Note that **7** inhibits *B. subtilis* division at a ∼20-fold lower concentration than the weak inhibitor 3-methoxybenzamide,^44^ which was the starting point in the PC190723 development.^14^ The other way around, compounds inhibiting bacterial division may now be tested in fluorescence anisotropy assays for potentially targeting the allosteric interdomain cleft (this work) or the nucleotide-binding site of FtsZ.^34,35^

### Enhancing the Affinity of Simplified Benzamide Inhibitors

**Figure 5:**
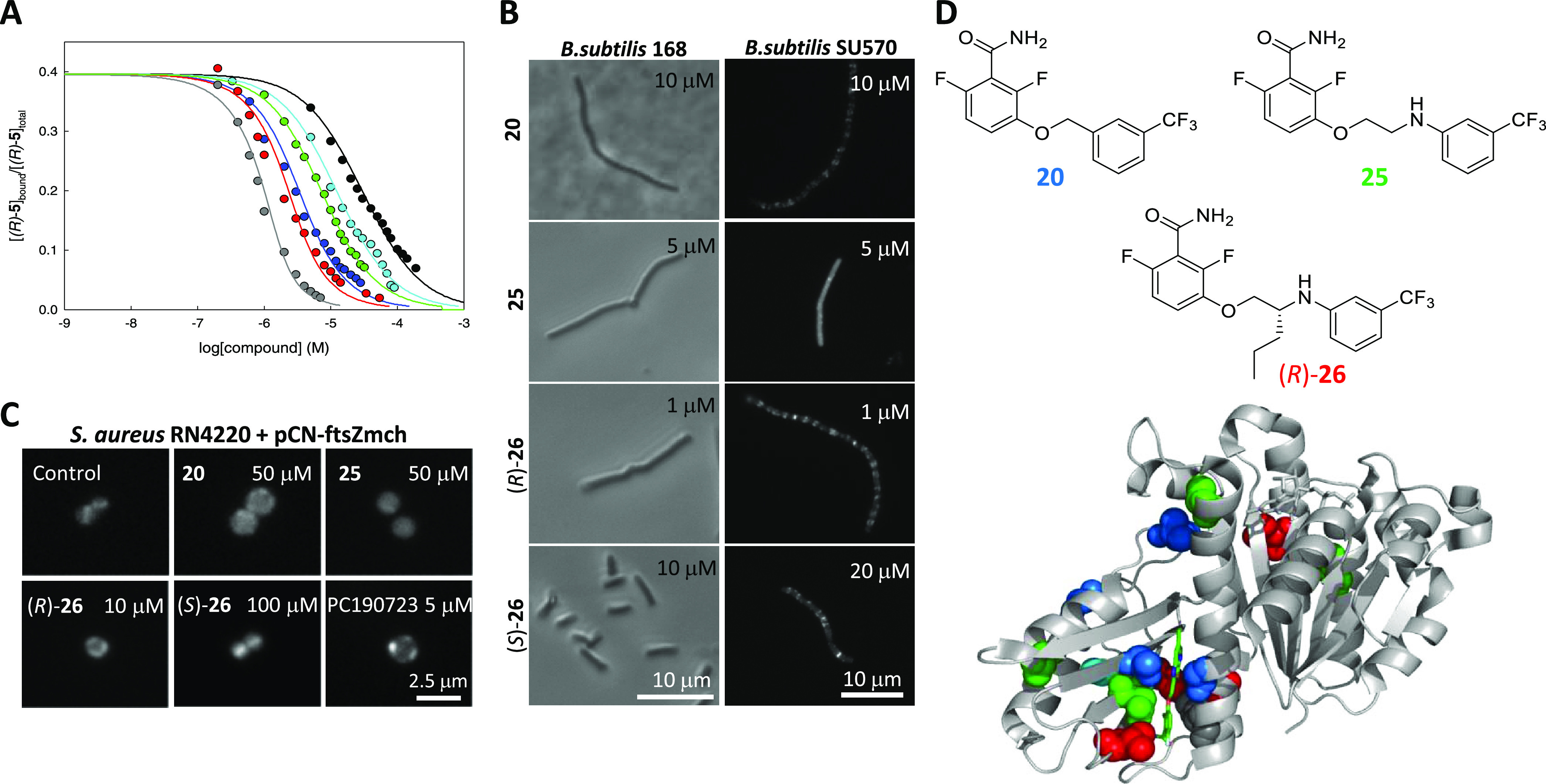
Simplified benzamide FtsZ inhibitors with enhanced affinity and phenotype. (A) Displacement curves of probe (*R*)-**5** (3 μM) from stabilized BsFtsZ-GMPCPP polymers (2.7 μM binding sites) by synthetic compounds **11** (black), **20** (blue), **25** (green), (*R*)-**26** (red), (*S*)-**26** (cyan), and by PC190723 (gray). Lines correspond to the best-fitted competition curve in each case. See *K*_D_ values in Table 1. (B) Left column, *B. subtilis* 168 cells after 3 h of culture with **20**, **25**, and (*R*)- and (*S*)-**26**, observed by phase-contrast microscopy; right column, cellular localization of FtsZ-GFP (*B. subtilis* SU570) following growth for 1.5 h in the presence of the same inhibitors. (C) *S. aureus* cells expressing FtsZ-mCherry (RN4220 + pCN-ftsZmch) grown for 1.5 h in the absence (control) or presence of **20**, **25**, (*R*)-, (*S*)-**26**, and PC190723, observed by fluorescence microscopy. (D) Top, structures of inhibitors **20**, **25**, and (*R*)-**26**. Bottom, mapping on the SaFtsZ structure (gray cartoon diagram of PDB entry 3VOA) of the amino acid residues modified in mutants resistant to **20** (blue), **25** (green), and (*R*)-**26** (red spheres).

Evaluation of 3-alkoxybenzamide **11**(45) showed that this oxygen derivative was more effective than nitrogen analogues **6**–**10** in our anisotropy screen (Figure 4A). Compound **11** had more than 10-fold lower *K*_D_ (10 μM, Figure 5A) and a minimal cell division inhibitory concentration (MDC) value (Table 1) than its 3-aminobenzamide analogue **6**. The *K*_D_ values, determined with the high-affinity probe (*R*)-**5**, are useful to compare the affinities of the different inhibitors among them and correlate with the corresponding MDC values. Derivatives of **11**, bearing an ethoxy or a propoxy spacer (**12** or **13**, respectively), had similar screen results (Figure 4A) and cell division inhibitory activities (Table 1), whereas the 4-substituted analogue **14** was toxic. Replacement of the amide group of **11** by isosteres gave compounds **15**–**17** with reduced inhibition (Figure 4A) that were also toxic to the cells. However, the addition of fluorine atoms at 2- and 6-positions of the benzamide ring of **11** (as in PC190723) rendered the 3-chloro derivative **18**(45,46) and the 3-trifluoromethyl analogue **20** that were both stronger competitors (Figure 4A) with 1.3 and 0.7 μM *K*_D_ values, respectively (Figure 5A and Table 1). Compound **20** induced a marked divisomal inhibition phenotype on *B. subtilis*, consisting of cell filamentation and GFP-FtsZ delocalization (Figure 5B and Table 1; raw data plots in Figure S4 and statistics in Table S2), as well as characteristic enlarged spherical cells in *S. aureus* and FtsZ-mCherry delocalization (Figure 5C). Analogues of **20** with ethoxy and propoxy linkers **22** and **23**, respectively, were weaker binders, as were the 2- and 4-trifluoromethyl analogues **19** and **21**. Adding a positively charged dimethylaminoethyl side chain at the linker rendered analogue **24** water-soluble, although canceled binding and cellular activity (Figure 4A and Table 1). At this point, we sought to further enhance inhibitor affinity with an analogous strategy to that employed with the fluorescent probes above. The starting point was compound **25** with an aminoethoxy linker between the two aromatic subunits, which is a higher affinity 3-trifluoromethylphenyl equivalent of the NBD probe **1** with comparable *K*_D_ and cellular activity values to **20** (Figure 5 and Table 1). The incorporation of a propyl side chain to the linker in (*R*)-**26**, equivalent to probe (*R*)-**5**, resulted in maximal affinity in the series (*K*_D_ = 0.2 μM) with a 1 μM division inhibitory activity on *B. subtilis* cells, comparable to PC190723 (Figure 5B and Table 1; raw data plots in Figure S4 and statistics in Table S2). The (*S*)-**26** enantiomer has 10–20-fold weaker affinity and MDC than the (*R*)-enantiomer, which can be related to the 8-fold reduced affinity of probe (*S*)-**5** with respect to (*R*)-**5**.

**Table 1:** Inhibition of Bacterial Cell Division and Growth by FtsZ Inhibitors

compd	*K*_D_^a^ (μM)	MDC^b^ (μM) *B. subtilis*	MDC (μM) *S. aureusMu50*	MIC^c^ (μM) *B. subtilis*	MIC (μM) *S. aureusMu50*
**11**	9.3 ± 0.5	50	ND^d^	50	>100
**12**	7.1 ± 0.5	50	ND	100	>100
**13**	9.5 ± 0.5	25	ND	100	>100
**18**	1.3 ± 0.1	10	50	25	100
**19**	71 ± 6	>100^e^	ND	>100^e^	>100^e^
**20**	0.7 ± 0.2 (0.7 ± 0.2)^f^	10	50	25	50^g^
**21**	6.7 ± 0.4	25	ND	50	100
**22**	5.9 ± 0.4	15	100	50	200
**23**	1.5 ± 0.1	5	25	10	50
**24**	145 ± 10	>50	>100	>100	>100
**25**	1.9 ± 0.2 (0.3 ± 0.1)^f^	5	50	10	100
*rac*-**26**	1.1 ± 0.1	2.5	25	5	50^h^
(*R*)-**26**	0.2 ± 0.1 (<0.1)^f^	1	10	2.5	25^i^
(*S*)-**26**	3.8 ± 0.2 (7.0 ± 0.5)^f^	>10	100^j^	50	100
PC190723	<0.1 (<0.1)^f^	2.5	1	5	5

a Binding affinities (*K*_D_) to stabilized polymers of FtsZ from *B. subtilis* are the mean ± standard error of the mean (SEM).

b MDC, minimal division inhibitory concentration.

c MIC, minimal growth inhibitory concentration.

d ND, not determined.

e Above compound solubility in the culture medium.

f In parenthesis, binding affinity values to stabilized SaFtsZ polymers.

g MIC = 25 μM on *S. aureus* subsp. *aureus* (ATCC 25923), *Staphylococcus haemolyticus*, and *Staphylococcus epidermidis*.

h MIC = 100 μM on *S. aureus* subsp. *aureus*, 50 μM on *S. haemolyticus*, and *S. epidermidis*.

i MIC = 25 μM on *S. aureus* subsp. *aureus*, *S. haemolyticus*, and *S. epidermidis*.

j Toxic at this concentration. Approximate MDC values of toxic compounds, omitted from the table, were: **15** 250 μM, **16** 50 μM, **17** 50 μM, and **14**, MIC > 500 μM.

### Antibacterial Activity of Inhibitors and FtsZ Cleft Targeting in *S. aureus*

Inspection of MDC and MIC values (Table 1) shows that the inhibitor activity on *B. subtilis* follows the affinity improvement along the compound series, reaching values similar to PC190723. The affinities of binding of **20**, **25**, and (*S*)-**26** to FtsZ from *B. subtilis* and *S. aureus* are reasonably similar (Table 1), as may be expected from the conservation of the PC109723 binding site in these two species.

Compound **20** is active against methicillin-resistant *S. aureus* Mu50, *S. haemolyticus*, and *S. epidermidis* (MIC 25–50 μM). However, in contrast to PC190723, the MDC value of **20** on *S. aureus* Mu50 is 5-fold higher than on *B. subtilis*, suggesting that the 3-trifluoromethylphenyl tail confers somewhat less activity against this strain than the chloro-thiazolopyridine tail. Then, the *S. aureus* MIC marginally decreases from **20** to (*R*)-**26**, compared to a 10-fold decrease in *B. subtilis*, indicating that the linker modifications that enhance affinity improve the antistaphylococcal activity little. We explored the cytotoxic effect of the best FtsZ inhibitors **18**, **20**, **25**, and (*R*)-**26** on human lung fibroblasts IMR90 (Table S3) and none of them significantly affected cell viability at their MIC value on *S. aureus* Mu50. Determination of the minimal bactericidal concentration (MBC) values showed that inhibitors **18** (MBC 100 μM), **20** (MBC 100 μM), **25** (MBC 100 μM), and (*R*)-**26** (MBC 25 μM) are bactericidal for *S. aureus*, as PC190723 (MBC 5 μM).

Puzzled by the comparatively high MDC and MIC values on *S. aureus*, we analyzed spontaneous *S. aureus* Mu50 (ATCC700699) mutants resistant to **20**, **25**, or (*R*)-**26**, which easily generated resistant strains (Table S4). Most mutations mapped to the *ftsZ* gene, at positions corresponding to amino acids (P115), (V151), A182, **G196**, **V214**, A237, L249, L261, M262, **N263**, (A285), V297, T309, and (T358). These residues predominantly cluster around the interdomain cleft (exceptions in parenthesis) and concentrate at the PC190723 binding site (Figure 5D); several of them are typically modified in PC190723-resistant mutants^14^ (in the bold type above). Therefore, the resistance mutation results strongly support that the inhibitors tested target *S. aureus* FtsZ at the interdomain cleft. The reduced activity in comparison to *B. subtilis* might be caused by permeability differences or by the action of staphylococcal multidrug resistance efflux pumps.

### Structural Insights into Inhibitor Binding to *S. aureus* FtsZ

**Figure 6:**
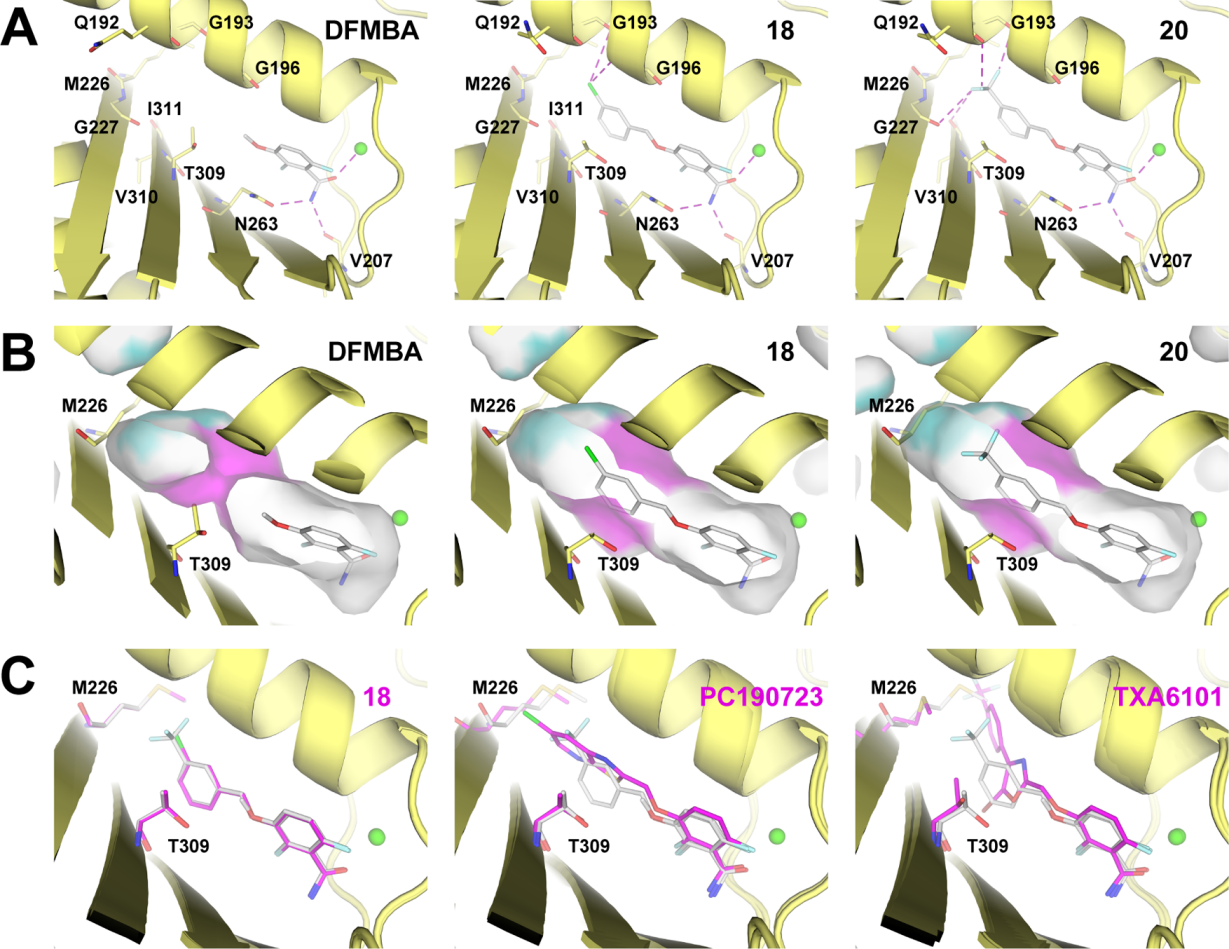
Crystal structures of FtsZ-inhibitor complexes. (A) Ligand–protein interactions in the binding site, indicated by dashed magenta lines (see the main text and Figure S6; the green sphere represents a coordinated metal ion). Complex structures have been deposited at the PDB with accession numbers: SaFtsZ-DFMBA, 6YD1; SaFtsZ-**18**, 6YD5; SaFtsZ-**20**, 6YD6. (B) Binding site surface, shown in light gray around the ligands. Magenta surface: Thr309 and Gly196. Cyan surface: Met226 and Gly193. (C) Structural comparison between the SaFtsZ-**20** (gray) complex with SaFtsZ-**18**, PC190723 (PDB: 4DXD), and TXA6101 (PDB: 5XDU) complexes (pink carbon atoms).

To confirm the proposed interaction of the inhibitors identified in our fluorescence screening, as a validation of the ligand-binding assay, we were able to determine the crystal structures of the SaFtsZ folded core (residues 12–316)^34^ in complex with inhibitors **18**, **20**, and DFMBA to 1.55–1.70 Å resolution (Figure 6 and Table S5). Omit electron density maps around the compounds clearly prove that the three molecules bind into the interdomain cleft (Figure S5), as PC190723,^15,25^ TXA6101, and TXA707^47^ (chemical structures in Chart S1). The protein structure in the three complexes is essentially the same as in inhibitor-free SaFtsZ (PDB 3VOA, mean root-mean-square deviation (rmsd) = 0.39 Å), the SaFtsZ-PC190723 complex (PDB 4DXD, mean rmsd = 0.41 Å), and the SaFtsZ-TXA6101 complex (PDB 5XDU, mean rmsd = 0.43 Å). Compounds **18** and **20** slightly increase the distance between helix H7 and the C-terminal domain by 0.5 Å as compared to DFMBA, while a larger displacement of 1 Å is observed in the other cases.

The protein interactions with the superimposable benzamide rings of **18**, **20**, DFMBA, PC190723, and TXA6101 are conserved (Figures 6A and S6). Nevertheless, these interactions appear insufficient for strong binding, as suggested by the 66% occupancy of DFMBA, compatible with its low affinity, while that of **18** and **20** is 100%. On the other hand, the halogenated phenyl moieties of **18** and **20** bind deep into the pocket with their benzene rings connecting to T309 and I311. This last residue adopts the same conformation in all examined complexes, while T309 acts as a gate presenting two different conformations, closed and open. The first is observed with DFMBA, the smaller bound molecule not reaching T309, and with TXA6101. In this case, the hydroxyl group of the T309 side chain points to the solvent, while the methyl group lies next to G196, thus closing the central region of the binding site (Figure 6B, purple). The open conformation is observed in the presence of **18**, **20**, or PC190723. In this arrangement, the T309 side chain points its hydroxyl group toward N263 and allows access to the central region of the binding site (Figure 6B, purple). Interestingly, compounds **18** and **20** adopt the flattest geometry, with their aromatic rings located in the same plane, as compared to PC190723 and TXA6101 (Figure 6C). This geometry places the halogenated benzene ring close to the position occupied by the oxazole ring of the flexible compound TXA6101 (Chart S1), where the 5-bromo group interacting with a deep hydrophobic pocket was linked to the enhanced compound activity.^31^ Unlike TXA6101, compounds **18**, **20**, and PC190723 do not displace the M226 side chain (Figure 6C), which constitutes another gate (Figure 6B, cyan) giving access to a ligand-induced pocket. Compounds **18** and **20** differ in their terminal halogenated group (Figure 6A). The chlorine atom of **18** is placed at 4 Å from both the O atom of G193 and the α-carbon of G196, while the trifluoromethyl group of **20** interacts with the backbone carbonyl oxygens of Q192, G193, G227, and V310.

Following crystal soaking with PC190723, we could reproduce a complex structure superimposable with 4DXD (not shown). In contrast, systematic soaking and cocrystallization experiments under similar conditions, employing the inhibitors with modified spacers between the benzamide and phenyl rings [**22**–**25**, (*R*)*-*, and (*S*)-**26**], all of the probes [**1**, (*R*)*-*, (*S*)-**2**, **3**, **4**, (*R*)-, and (*S*)-**5**], as well as probes with longer spacers,^27^ gave FtsZ electron density maps clearly showing an empty interdomain cleft. Note, however, that each of these ligands binds specifically to FtsZ polymers in solution, several of them with low micromolar affinities (Table 1) and higher solubility than PC190723 (Table S6). Recently, the crystal structure of SaFtsZ in complex with a fluorescent BODIPY derivative of TXA6101 bound into the interdomain cleft has been reported. Nonetheless, this probe (BOFP, Chart S1) binds to unassembled SaFtsZ in solution without nucleotide and magnesium, as well as to Gram-negative FtsZs, in puzzling contrast with its complex filament crystal structure,^48^ the specificity of our probes, and the characteristic selectivity of PC190723 analogues.

SaFtsZ mutations conferring resistance to the inhibitors can be analyzed based on the SaFtsZ-**18** and -**20** complex structures. In particular, mutations G196S, M262I, and T309I confer resistance to **20**. The trifluoromethyl group of **20** is placed close to G196 and the introduction of a side chain in this position would produce steric clashes similarly to PC190723. Residue M262 locates at the center of the β-sheet that forms one side of the ligand-binding cleft. While its side chain points away from the cleft, the M262I mutation can modify the hydrophobic packing of the β-sheet and, thus, potentially affect inhibitor binding. An important modification is introduced in the binding cleft with the T309I mutation. The introduction of a bulkier residue will likely impede its movement, keeping closed the central region of the binding site, thus clogging the binding of compounds.

In summary, our crystallographic analysis confirms the allosteric site for compounds **18** and **20** and provides insights into the binding to FtsZ of simplified benzamide-based bacterial cell division inhibitors. Their halogenated phenyl rings, replacing the larger chloro-thiazolopyridine moiety of PC190723, structurally superpose to the middle bromo-oxazole ring of TXA6101 upon binding to FtsZ. Potential ligand extensions might reach the ligand-induced cavity occupied by the TXA6101 trifluoromethyl group or the upper unfilled cavity in FtsZ.

## Conclusions

The allosteric benzamide binding site at the cleft between the nucleotide-binding and GTPase-activating domains of FtsZ has been explored in this work. This cleft opens when FtsZ switches into its active conformation for assembly into dynamic polar filament clusters that guide bacterial division. We have developed a novel competitive assay employing specific fluorescent probes to screen for allosteric inhibitors and evaluate their binding affinity. Fluorescence polarization screening, together with cytological profiling and X-ray crystallography, has permitted us to identify effective, structurally simplified benzamide FtsZ-targeting inhibitors of bacterial division in *B. subtilis* and methicillin-resistant *S. aureus*. Our in vitro competitive binding assay and in situ cell-based methodology, combined with structural insights into ligand extension, should facilitate the discovery and validation of new structurally diverse FtsZ allosteric inhibitors leading to more effective antibacterial agents.

## Experimental Section

### Synthesis

Unless stated otherwise, starting materials, reagents, and solvents were purchased as high-grade commercial products from Abcr, Acros, Scharlab, Sigma-Aldrich, or Thermo Fisher Scientific and were used without further purification. All nonaqueous reactions were performed under an argon atmosphere in oven-dried glassware. Tetrahydrofuran (THF), diethyl ether, and dichloromethane (DCM) were dried using a Pure Solv Micro 100 Liter solvent purification system. Triethylamine was dried over KOH and distilled before use. Reactions under MW irradiation were performed in a Biotage Initiator 2.5 reactor. Reactions were monitored by analytical thin-layer chromatography (TLC) on silica gel plates supplied by Merck (Kieselgel 60 F-254) with detection by UV light (254 nm) or 10% phosphomolybdic acid solution in EtOH. Flash chromatography was performed on a Varian 971-FP flash purification system using silica gel cartridges (Varian, particle size 50 μm). Melting points (mp, uncorrected) were determined on a Stuart Scientific electrothermal apparatus. Infrared (IR) spectra were measured on a Bruker Tensor 27 instrument equipped with a Specac attenuated total reflection (ATR) accessory of 5200–650 cm^–1^ transmission range; frequencies (ν) are expressed in cm^–1^. Nuclear magnetic resonance (NMR) spectra were recorded on a Bruker Avance III 700 MHz (^1^H, 700 MHz; ^13^C, 175 MHz), Bruker Avance 500 MHz (^1^H, 500 MHz; ^13^C, 125 MHz), or Bruker DPX 300 MHz (^1^H, 300 MHz; ^13^C, 75 MHz) instruments at the Universidad Complutense de Madrid (UCM) NMR core facilities. Chemical shifts (δ) are expressed in parts per million relative to internal tetramethylsilane; coupling constants (*J*) are in hertz (Hz). The following abbreviations are used to describe peak patterns when appropriate: singlet (s), doublet (d), triplet (t), quartet (q), quintet (qt), sextet (sext), multiplet (m), broad (br), and apparent (app). Two-dimensional (2D) NMR experiments (heteronuclear multiple quantum coherence (HMQC) and heteronuclear multiple bond correlation (HMBC)) of representative compounds were carried out to assign protons and carbons of the new structures. High-resolution mass spectrometry (HRMS) was carried out on a Bruker FTMS APEX-Q-IV spectrometer in an electrospray ionization (ESI) mode or on a Bruker matrix-assisted laser desorption ionization time-of-flight (MALDI-TOF)/TOF ULTRAFLEX spectrometer at the UCM’s mass spectrometry core facility. High-performance liquid chromatography coupled to mass spectrometry (HPLC-MS) analysis was performed using an Agilent 1200LC-MSD VL instrument. LC separation was achieved with a Zorbax Eclipse XDB-C18 column (5 μm, 4.6 mm × 150 mm) or a Zorbax SB-C3 column (5 μm, 2.1 mm × 50 mm), both together with a guard column (5 μm, 4.6 mm × 12.5 mm). The gradient mobile phases consisted of A (95:5 water/acetonitrile) and B (5:95 water/acetonitrile) with 0.1% ammonium hydroxide and 0.1% formic acid as the solvent modifiers. MS analysis was performed with an ESI source. The capillary voltage was set to 3.0 kV and the fragmentor voltage was set at 72 eV. The drying gas temperature was 350 °C, the drying gas flow was 10 L/min, and the nebulizer pressure was 20 psi. Spectra were acquired in a positive or negative ionization mode from 100 to 1200 *m*/*z* and in a UV mode at four different wavelengths (210, 230, 254, and 280 nm). Optical rotation [α] was measured on an Anton Paar MCP 100 modular circular polarimeter using a sodium lamp (λ = 589 nm) with a 1 dm path length; concentrations (c) are given as g/100 mL. The enantiomeric excess (ee) was determined by HPLC using a chiral column (Chiralpak IA, 5 μm, 4.6 mm × 150 mm) and hexane/isopropanol/triethylamine/trifluoroacetic acid (70/30/0.3/0.1) as mobile phase (1 mL/min). HPLC traces were compared to racemic samples obtained by mixing equal amounts of the enantiopure compounds obtained independently.

Spectroscopic data of all described compounds were consistent with the proposed structures. Satisfactory HPLC-MS chromatograms were obtained for all tested compounds, which confirmed a purity of at least 95%.

### General Procedure for the Synthesis of Final Compounds **2**–**5**

To a solution of amine **35**–**37** or **40** (1 equiv) and Cs_2_CO_3_ (3 equiv) in dry acetonitrile (50 mL/mmol), a solution of NBD-Cl (1–1.2 equiv) in dry acetonitrile (1 mL/mmol) was added and the reaction was stirred at 80 °C for 1 h. Then, the solvent was evaporated under reduced pressure and the crude was purified by chromatography (from hexane to hexane/EtOAc, 1:2) to afford final compounds **2**–**5**.

#### 2,6-Difluoro-3-[(1-{[(7-nitro-2,1,3-benzoxadiazol-4-yl)amino]methyl}butyl)oxy]benzamide (**2**)

Obtained from amine **35** (28 mg, 0.11 mmol), NBD-Cl (22 mg, 0.11 mmol) and Cs_2_CO_3_ (108 mg, 0.33 mmol) as an oil in 50% yield (23 mg). *R*_f_ (hexane/EtOAc, 1:2) 0.52; IR (ATR) ν 3351 (NH), 1683 (CO), 1623, 1507, 1489 (Ar); ^1^H NMR (700 MHz, CDCl_3_) δ 0.99 (t, *J* = 7.5, 3H, CH_3_), 1.46–1.54 (m, 2H, CH_2_), 1.68–1.77 (m, 1H, 1/2CH_2_), 1.81–1.89 (m, 1H, 1/2CH_2_), 3.67–3.82 (m, 2H, CH_2_N), 4.44–4.52 (m, 1H, CHO), 6.00 (br s, 2H, NH_2_), 6.25 (d, *J* = 8.5, 1H, CH_NBD_), 6.59 (br s, 1H, NH), 6.88 (td, *J* = 9.0, 1.5, 1H, H_5_), 7.07 (td, *J* = 9.0, 5.0, 1H, H_4_), 8.49 (d, *J* = 8.5, 1H, CH_NBD_); ^13^C NMR (CDCl_3_, 175 MHz) δ 14.2 (CH_3_), 18.6, 34.1 (2CH_2_), 46.9 (CH_2_N), 80.3 (CHO), 99.3 (br s, CH_NBD_), 111.9 (dd, *J*_C–F_ = 23.5, 4.0, C_5_), 114.6 (dd, *J*_C–F_ = 20.9, 16.8, C_1_), 122.0 (dd, *J* = 9.8, 2.3, C_4_), 124.6 (C_NBD_), 136.4 (CH_NBD_), 142.1 (dd, *J*_C–F_ = 11.0, 3.0, C_3_), 144.0 (2C_NBD_), 144.4, (C_NBD_), 151.6 (dd, *J*_C–F_ = 254.0, 7.0, CF), 154.7 (dd, *J*_C–F_ = 249.0, 5.5, CF), 161.8 (CONH_2_); ESI-HRMS (calcd, found for C_18_H_21_F_2_N_6_O_5_ [M + NH_4_]^+^): 439.1536, 439.1548.

##### (*R*)-**2**

Obtained from amine (*R*)-**35** (60 mg, 0.23 mmol), NBD-Cl (56 mg, 0.28 mmol), and Cs_2_CO_3_ (151 mg, 0.46 mmol) as an oil in 31% yield (30 mg). [α]_D_^24^ = +35.0 (*c* = 0.16, CHCl_3_). Chiral HPLC (*t*_R_, min): 7.99, ee = 98%.

##### (*S*)-**2**

Obtained from amine (*S*)-**35** (65 mg, 0.25 mmol), NBD-Cl (60 mg, 0.30 mmol), and Cs_2_CO_3_ (164 mg, 0.50 mmol) as an oil in 33% yield (35 mg). [α]_D_^24^ = −41.0 (*c* = 0.16, CHCl_3_). Chiral HPLC (*t*_R_, min): 6.82, ee = 99%.

#### 2,6-Difluoro-3-[(1-{[(7-nitro-2,1,3-benzoxadiazol-4-yl)amino]methyl}hexyl)oxy]benzamide (**3**)

Obtained from amine **36** (17 mg, 0.06 mmol), NBD-Cl (12 mg, 0.06 mmol), and Cs_2_CO_3_ (59 mg, 0.18 mmol) as an oil in 30% yield (8 mg). *R*_f_ (hexane/EtOAc, 1:2) 0.42; IR (ATR) ν 3314 (NH), 1675 (CO), 1626, 1582, 1487 (Ar); ^1^H NMR (700 MHz, CDCl_3_) δ 0.90 (t, *J* = 7.0, 3H, CH_3_), 1.30–1.34 (m, 4H, 2CH_2_), 1.44–1.49 (m, 2H, CH_2_), 1.70–1.76 (m, 1H, 1/2CH_2_), 1.83–1.88 (m, 1H, 1/2CH_2_), 3.69–3.76 (m, 1H, 1/2CH_2_N), 3.79–3.85 (m, 1H, 1/2CH_2_N), 4.43–4.50 (m, 1H, CHO), 5.96–5.99 (m, 2H, NH_2_), 6.25 (d, *J* = 8.6, 1H, CH_NBD_), 6.58 (t, *J* = 5.7, 1H, NH), 6.89 (td, *J* = 9.0, 1.9, 1H, H_5_), 7.07 (td, *J* = 9.0, 5.2, 1H, H_4_), 8.49 (d, *J* = 8.6, 1H, CH_NBD_); ^13^C NMR (CDCl_3_, 175 MHz) δ 14.1 (CH_3_), 22.6, 24.9, 31.8, 32.0 (4CH_2_), 46.8 (CH_2_N), 80.6 (CHO), 99.2 (br s, CH_NBD_), 111.9 (dd, *J*_C–F_ = 23.5, 3.5, C_5_), 114.6 (dd, *J*_C–F_ = 20.9, 17.3, C_1_), 122.3 (dd, *J*_C–F_ = 9.8, 2.2, C_4_), 124.9 (C_NBD_), 136.3 (CH_NBD_), 142.0 (dd, *J*_C–F_ = 11.5, 2.5, C_3_), 143.8, 144.0, 144.5 (3C_NBD_), 151.8 (dd, *J*_C–F_ = 254.0, 7.0, CF), 154.9 (dd, *J*_C–F_ = 249.0, 5.0, CF), 161.4 (CONH_2_); ESI-HRMS (calcd, found for C_20_H_20_F_2_N_5_O_5_ [M – H]^−^): 448.1438, 448.1419.

#### 2,6-Difluoro-3-[(1-{[(7-nitro-2,1,3-benzoxadiazol-4-yl)amino]methyl}nonyl)-oxy]benzamide (**4**)

Obtained from amine **37** (110 mg, 0.26 mmol), NBD-Cl (62 mg, 0.26 mmol), and Cs_2_CO_3_ (254 mg, 0.78 mmol) as an oil in 11% yield (14 mg). *R*_f_ (hexane/EtOAc, 1:1) 0.42; IR (ATR) ν 3426 (NH), 1700 (CO), 1378, 1364 (Ar); ^1^H NMR (700 MHz, acetone-*d*_6_) δ 0.86 (t, *J* = 7.1, 3H, CH_3_), 1.25–1.36 (m, 10H, 5CH_2_), 1.50–1.59 (m, 2H, CH_2_), 1.85–1.88 (m, 2H, CH_2_), 4.01 (m, 2H, CH_2_N), 4.82 (qt, *J* = 5.7, 2H, CHO), 6.61 (d, *J* = 8.8, 1H, CH_NBD_), 6.90 (td, *J* = 9.0, 1.8, 1H, H_5_), 7.15 (br s, 1H, 1/2NH_2_), 7.26 (td, *J* = 9.2, 5.2, 1H, H_4_), 7.37 (br s, 1H, 1/2NH_2_), 8.31 (br s, 1H, NH), 8.53 (d, *J* = 8.7, 1H, CH_NBD_); ^13^C NMR (175 MHz, acetone-*d*_6_) δ 14.3 (CH_3_), 23.3, 25.6, 30.2 (3CH_2_), 30.3 (2CH_2_), 32.6, 32.8 (2CH_2_), 47.8 (br s, CH_2_N), 80.1 (CHO), 100.2 (br s, CH_NBD_), 111.7 (dd, *J*_C–F_ = 23.4, 3.9, C_5_), 117.6 (dd, *J*_C–F_ = 24.3, 20.4, C_1_), 119.3 (dd, *J*_C–F_ = 9.0, 1.7, C_4_), 123.9 (C_NBD_), 137.7 (br s, CH_NBD_), 143.4 (dd, *J*_C–F_ = 11.1, 3.2, C_3_), 145.1 (C_NBD_), 145.6 (2C_NBD_), 150.7 (dd, *J*_C–F_ = 249.7, 8.6, CF), 153.9 (dd, *J*_C–F_ = 243.2, 6.5, CF), 162.0 (CONH_2_); ESI-HRMS (calcd, found for C_23_H_26_F_2_N_5_O_5_ [M – H]^−^): 490.1902, 490.1891.

#### 2,6-Difluoro-3-{2-[(4-nitro-2,1,3-benzoxadiazol-7-yl)amino]pentoxy}benzamide (**5**)

Obtained from amine **40** (15 mg, 0.06 mmol), NBD-Cl (11 mg, 0.06 mmol), and Cs_2_CO_3_ (55 mg, 0.18 mmol) as an oil in 13% yield (3 mg). Chromatography: DCM/EtOAc, 9:1–7:3. *R*_f_ (hexane/EtOAc, 1:2) 0.32; IR (ATR) ν 3390 (NH), 1726 (CO), 1634, 1485 (Ar); ^1^H NMR (700 MHz, methanol-*d*_4_) δ 1.01 (t, *J* = 7.5, 3H, CH_3_), 1.46–1.60 (m, 2H, CH_2_), 1.81–1.91 (m, 2H, CH_2_), 4.19–4.21 (m, 1H, 1/2CH_2_O), 4.30 (dd, *J* = 10.0, 4.0, 1H, 1/2CH_2_O), 4.37 (br s, 1H, CHN), 6.54 (d, *J* = 9.0, 1H, CH_NBD_), 6.93 (td, *J* = 9.0, 2.0, 1H, H_5_), 7.18 (td, *J* = 9.0, 5.0, 1H, H_4_), 8.52 (d, *J* = 8.5, 1H, CH_NBD_); ^13^C NMR (175 MHz, methanol-*d*_4_) δ 14.2 (CH_3_), 20.3, 34.0 (2CH_2_), 54.8 (CHN), 73.3 (CH_2_O), 100.4 (CH_NBD_), 111.9 (dd, *J*_C–F_ = 23.0, 4.0, C_5_), 116.8 (dd, *J*_C–F_ = 24.0, 20.0, C_1_), 117.9 (d, *J*_C–F_ = 10.0, C_4_), 123.3 (C_NBD_), 138.5 (CH_NBD_), 144.7 (dd, *J*_C–F_ = 11.0, 3.0, C_3_), 145.6 (2C_NBD_), 145.9 (C_NBD_), 150.4 (dd, *J*_C–F_ = 251.5, 8.0, CF), 154.3 (dd, *J*_C–F_ = 244.0, 6.0, CF), 165.2 (CONH_2_); ESI-HRMS (calcd., found for C_18_H_16_F_2_N_5_O_5_ [M – H]^−^): 420.1125, 420.1124.

##### (*R*)-**5**

Obtained from amine (*R*)-**40** (40 mg, 0.15 mmol), NBD-Cl (31 mg, 0.16 mmol), and Cs_2_CO_3_ (100 mg, 0.30 mmol) as an oil in 30% yield (19 mg).

##### (*S*)-**5**

Obtained from amine (*S*)-**40** (141 mg, 0.55 mmol), NBD-Cl (110 mg, 0.55 mmol), and Cs_2_CO_3_ (538 mg, 1.65 mmol) as an oil in 7% yield (19 mg).

### General Procedure for the Synthesis of Compounds **18** and **20**

To a suspension of compound **27** (1 equiv) in anhydrous DMF (6 mL/mmol), potassium carbonate (1.5 equiv), sodium iodide (0.2 equiv), and the corresponding bromo derivative (1.5 equiv) were added. The reaction mixture was stirred at rt for 24 h. Then, the mixture was diluted in EtOAc and washed with brine (3×). The organic phase was dried (Na_2_SO_4_), filtered, and concentrated under reduced pressure. The residue was purified by chromatography (hexane to hexane/EtOAc, 2:8) to yield the desired compounds.

#### 3-[(3-Chlorobenzyl)oxy]-2,6-difluorobenzamide (**18**)

Obtained from **27** (237 mg, 1.4 mmol) and 3-chlorobenzyl bromide (0.25 mL, 2.0 mmol) as a white solid in 89% yield (293 mg). *R*_f_ (hexane/EtOAc, 3:7) 0.62; mp: 125–127 °C (lit.^45^ 121–122 °C); IR (ATR) ν 3378, 3192 (NH), 1655 (CO), 1594, 1492, 1455 (Ar); ^1^H NMR (300 MHz, acetone-*d*_6_) δ 5.23 (s, 2H, CH_2_), 6.94 (td, *J* = 9.0, 2.0, 1H, H_5_), 7.19 (br s, 1H, 1/2NH_2_), 7.26 (td, *J* = 9.0, 5.0, 1H, H_4_), 7.36–7.47 (m, 4H, H_3′_-H_5′_, 1/2NH_2_), 7.54 (br s, 1H, H_2′_); ^13^C NMR (75 MHz, acetone-*d*_6_) δ 71.4 (CH_2_), 111.6 (dd, *J* = 23.0, 4.5, C_5_), 117.2 (dd, *J* = 9.4, 2.7, C_4_), 117.6 (t, *J* = 19.9, C_1_), 126.9 (CH_Ar_), 128.3 (C_2′_), 129.0, 131.1 (2CH_Ar_), 134.8 (C_3′_), 140.1 (C_1′_), 144.0 (dd, *J*_C–F_ = 11.0, 3.0, C_3_), 150.0 (dd, *J*_C–F_ = 249.9, 8.0, CF), 153.8 (dd, *J*_C–F_ = 243.6, 6.6, CF), 162.1 (CONH_2_); ESI-HRMS (calcd found for C_14_H_11_ClF_2_NO_2_ [M(^35^Cl) + H]^+^): 298.0441, 298.0453; (calcd found for C_14_H_11_ClF_2_NO_2_ [M(^37^Cl) + H]^+^): 300.0411, 300.0424.

#### 2,6-Difluoro-3-{[3-(trifluoromethyl)benzyl]oxy}benzamide (**20**)

Obtained from **27** (217 mg, 1.3 mmol) and 3-(trifluoromethyl)benzyl bromide (0.28 mL, 1.9 mmol) as a white solid in 98% yield (272 mg). *R*_f_ (hexane/EtOAc, 3:7) 0.60; mp: 140–143 °C; IR (ATR) ν 3371, 3194 (NH), 1655 (CO), 1592, 1495, 1456 (Ar); ^1^H NMR (500 MHz, acetone-*d*_6_) δ 5.33 (s, 2H, CH_2_), 6.98 (td, *J* = 9.0, 2.0, 1H, H_5_), 7.19 (br s, 1H, 1/2NH_2_), 7.30 (td, *J* = 9.0, 5.0, 1H, H_4_), 7.46 (br s, 1H, 1/2NH_2_), 7.65–7.72 (m, 2H, H_4′_, H_5′_), 7.81 (d, *J* = 7.5 1H, H_6′_), 7.85 (s, 1H, H_2′_); ^13^C NMR (125 MHz, acetone-*d*_6_) δ 71.6 (CH_2_), 111.6 (dd, *J*_C–F_ = 23.3, 3.1, C_5_), 117.3 (dd, *J*_C–F_ = 9.5, 2.0, C_4_), 117.6 (t, *J*_C–F_ = 23.9, C_1_), 125.1 (d, *J*_C–F_ = 2.8, C_2′_) 125.3 (q, *J*_C–F_ = 271.5, CF_3_), 125.7 (d, *J*_C–F_ = 2.7, C_4′_) 130.4 (C_5′_), 131.2 (q, *J*_C–F_ = 32.0, C_3′_), 132.3 (C_6′_), 139.1 (C_1′_), 144.1 (dd, *J* = 11.0, 2.0, C_3_), 150.1 (dd, *J*_C–F_ = 250.0, 8.0, CF), 153.9 (dd, *J*_C–F_ = 243.0, 6.0, CF), 162.1 (CONH_2_); ESI-HRMS (calcd found for C_15_H_11_F_5_NO_2_ [M + H]^+^): 332.0710, 332.0720.

### General Procedure for the Synthesis of Compounds **25** and **26**

A solution of compound **27**, alcohol **45** or **46** (1 equiv), tributylphosphine (1 equiv), and diisopropyl azodicarboxylate (1 equiv) in anhydrous DMF (8 mL/mmol) was stirred under MW irradiation at 150 °C for 90 min. Then, the reaction was diluted with EtOAc (30 mL) and washed with brine (3 × 20 mL). The organic phase was dried, filtered, and concentrated under reduced pressure. The residue was purified by chromatography (from hexane to hexane/EtOAc, 1:1) to yield the desired final compounds **25** and **26**.

#### 2,6-Difluoro-3-(2-{[3-(trifluoromethyl)phenyl]amino}ethoxy)benzamide (**25**)

Obtained from **27** (80 mg, 0.46 mmol) and alcohol **45** (95 mg, 0.46 mmol) as an oil in 49% yield (81 mg). *R*_f_ (hexane/EtOAc, 1:1) 0.25; IR (ATR) ν 3354, 3189 (NH), 1671 (CO), 1614, 1490 (Ar); ^1^H NMR (300 MHz, methanol-*d*_4_) 3.57 (t, *J* = 5.4, 2H, CH_2_N), 4.22 (t, *J* = 5.4, 2H, CH_2_O), 6.79–7.03 (m, 3H, H_2′_, H_4′_, H_6′_, H_5_), 7.18 (td, *J* = 9.2, 5.1, 1H, H_4_), 7.28 (t, *J* = 7.8, 1H, H_5′_); ^13^C NMR (75 MHz, methanol-*d*_4_) δ mixture of rotamers: 44.1 (CH_2_N), 70.1 (CH_2_O), 110.1 (q, *J*_C–F_ = 4.0, C_2′_), 111.9 and 112.1 (dd, *J*_C–F_ = 23.2, 4.2, C_5_), 114.4 (q, *J*_C–F_ = 3.9, C_4′_), 117.2 (br s, C_1_, C_6′_), 117.9 (dd, *J*_C–F_ = 9.3, 2.8) and 119.6 (dd, *J*_C–F_ = 9.2, 4.0, C_4_), 125.9 (q, *J*_C–F_ = 271.5, CF_3_), 130.8 (C_5′_), 132.5 (q, *J*_C–F_ = 31.4, C_3′_), 144.9 (dd, *J*_C–F_ = 10.9, 3.4, C_3_), 149.9 (C_1′_), 150.5 (dd, *J*_C–F_ = 251.5, 7.6, CF), 154.2 (dd, *J*_C–F_ = 243.7, 6.0, CF), 165.4 (CONH_2_); MALDI-HRMS (calcd found for C_16_H_13_F_5_N_2_O_2_ [M]^+^): 360.0897, 360.0903.

#### 2,6-Difluoro-3-[(2-{[3-(trifluoromethyl)phenyl]amino}pentyl)oxy]benzamide (**26**)

Obtained from **27** (52 mg, 0.30 mmol) and alcohol **46** (75 mg, 0.30 mmol) as a colorless oil in 45% yield (30 mg). *R*_f_ (hexane/EtOAc, 1:1) 0.48; IR (ATR) ν 3363 (NH), 1685 (CO), 1491, 1445 (Ar); ^1^H NMR (300 MHz, CDCl_3_) δ 0.98 (t, *J* = 7.0, 3H, CH_3_), 1.41–1.69 (m, 3H, 31/2CH_2_), 1.77–1.84 (m, 1H, 1/2CH_2_), 3.75–3.79 (m, 1H, CHN), 4.04 (AB system, *J* = 9.3, 3.9, 2H, CH_2_O), 5.95 (br s, 2H, NH_2_), 6.77 (d, *J* = 8.1, 1H, H_6′_), 6.82 (s, 1H, H_2′_), 6.87 (dd, *J* = 9.1, 1.8, 1H, H_5_), 6.93 (d, *J* = 8.0, 1H, H_4′_), 6.96 (td, *J* = 9.1, 5.1, 1H, H_4_), 7.25 (t, *J* = 7.8, 1H, H_5′_); ^13^C NMR (75 MHz, CDCl_3_) δ 14.2 (CH_3_), 19.5 (*C*H_2_CH_3_), 34.4 (*C*H_2_CH), 52.5 (CHN), 72.0 (CH_2_O), 109.4 (q, *J*_C–F_ = 3.8, C_2′_), 111.4 (dd, *J*_C–F_ = 23.7, 4.3, C_5_), 114.1 (q, *J*_C–F_ = 4.0, C_4′_), 114.2 (q, *J*_C–F_ = 36.3, C_1_), 116.4 (C_6′_), 117.7 (dd, *J*_C–F_ = 9.8, 3.0, C_4_), 125.2 (q, *J*_C–F_ = 272.4, CF_3_), 130.0 (C_5′_), 131.9 (q, *J*_C–F_ = 31.7, C_3′_), 144.0 (dd, *J*_C–F_ = 11.4, 3.4, C_3_), 147.6 (C_1′_), 150.6 (dd, *J*_C–F_ = 254.9, 7.2, CF), 153.9 (dd, *J*_C–F_ = 247.2, 5.4, CF), 162.0 (CONH_2_); ESI-HRMS (calcd found for C_19_H_19_F_5_N_2_O_2_Na [M + Na]^+^): 425.1264, 425.1700.

##### (*R*)-**26**

Obtained from **27** (50 mg, 0.29 mmol) and alcohol (*R*)-**46** (73 mg, 0.29 mmol) as an oil in 49% yield (57 mg). [α]_D_^24^ = +29.0 (*c* = 0.15, CHCl_3_).

##### (*S*)-**26**

Obtained from **27** (116 mg, 0.67 mmol) and alcohol (*S*)-**46** (150 mg, 0.61 mmol) as an oil in 36% yield (88 mg). [α]_D_^24^ = −28.5 (*c* = 0.15, CHCl_3_).

### Fluorescent Probes and Inhibitors

All tested compounds were dissolved in DMSO (spectroscopic grade, Merck) before use and kept frozen and dry. Previously reported compounds PC190723, DFMBA, CTPM, UCM44, and PC170942 were synthesized as described.^23,35^ Zantrin Z3 was obtained from Mcule. Plumbagin, resveratrol, and tiplaxtinin were purchased from Sigma-Aldrich.

### Protein Purification and Assembly Conditions

FtsZ from *B. subtilis* (BsFtsZ) was prepared as described previously.^35^ FtsZ was assembled in 50 mM *N*-(2-hydroxyethyl)piperazine-*N*′-ethanesulfonic acid (HEPES)-KOH, 50 mM KCl, 1 mM ethylenediaminetetraacetic acid (EDTA), pH 6.8 (HEPES buffer) plus 10 mM MgCl_2_, and 1 mM GTP or 0.1 mM GMPCPP at 25 °C. FtsZ assembly was monitored by light scattering.^34^

Full-length *S. aureus* FtsZ (SaFtsZ) and the truncated protein SaFtsZ(12–315) were purified as described.^34^ SaFtsZ was assembled in 50 mM MES, 50 mM KCl, 1 mM EDTA, pH 6.5 plus 10 mM MgCl_2_, and 0.1 mM GMPCPP.

### Fluorescence and Anisotropy Measurements

The fluorescence spectra and anisotropy values of the different probes were acquired with a Fluoromax-4 (Horiba Jobin Yvon) photon-counting L-format spectrofluorometer using 2 mm × 10 mm cells at 25 °C, employing the maximum excitation and emission wavelength for each probe with 2 and 5 nm bandwidths, respectively. Anisotropy values were automatically measured as *r* = (Ivv – *G* Ivh)/(Ivv + 2*G* Ivh), where the subscripts refer to the vertical (v) or horizontal (h) positions of the excitation (first subscript) and emission (second subscript) polarizers, and *G* = Ihv/Ihh. Total intensity values were simultaneously determined as *I*_T_ = Ivv +2*G* Ivh. Note that measurement correction procedures with T-format (double channel) fluorometers and with plate readers are slightly different.

### Preparation of Cross-Linked FtsZ Polymers

FtsZ (10–15 μM) was assembled in HEPES buffer, 10 mM MgCl_2_, 50 μM GMPCPP at 25 °C for 10 min, and then 0.15% (v/v) glutaraldehyde^36^ (distilled grade for microscopy, TAAB Laboratories, U.K.) was added to the solution that was incubated at 25 °C for 10 min more. (This was the minimal glutaraldehyde concentration determined to stabilize FtsZ polymers specifically binding probe 1.) The remains of the cross-linking agent were quenched by adding 60 mM NaBH_4_, the sample was incubated on ice for 10 min and degassed.^36^ Cross-linked polymers were centrifuged for 10 min at 8200*g* (5000 rpm) and 4 °C in 15 mL Falcon tubes employing a Rotina 380R (Hettich) centrifuge, the supernatant was removed and the pellet was resuspended in the same volume of HEPES buffer, 10 mM MgCl_2_, containing 5 μM GMPCPP. Fixed FtsZ polymers were active in binding assays after more than 2 days at 4 °C; they could also be frozen in liquid nitrogen with a small loss of binding capacity. However, they were observed to precipitate above 20 μM FtsZ concentration. Cross-linked polymers of SaFtsZ were similarly prepared.

### Stoichiometry and Affinity of Binding of the Fluorescent Probes to FtsZ Polymers

The stoichiometry of binding of (*R*)-**5** to stabilized FtsZ polymers was measured using a centrifugation assay adapted from ref (49). Then, FtsZ polymers (10 μM) were incubated at 25 °C in HEPES buffer, 10 mM MgCl_2_, 2% DMSO, and 0.1 mM GMPCPP at 25 °C in the presence of different probe concentrations, in a final volume of 0.1 mL. The samples were then centrifuged for 20 min at 100 000 rpm and 25 °C in a TLA-100 rotor. After centrifugation, the supernatant was carefully withdrawn and the pellets were resuspended in the same volume of buffer. The concentration of the free probe was determined spectrophotometrically in the supernatant, employing an extinction coefficient ε_483_ = 25 400 M^–1^ cm^–1^ for (*R*)-**5** and the concentration of probe bound to FtsZ was calculated as the difference of the known total concentration of probe minus the free concentration. To calculate the FtsZ polymer concentration, 5 μL of the resuspended pellet was applied to sodium dodecyl sulfate (SDS)-12% polyacrylamide gels, stained with Coomassie blue, scanned with a CS-800 calibrated densitometer (BioRad), and the protein bands quantified using Quantity One software (BioRad).

Binding affinity of the probes to FtsZ was measured by an increase in anisotropy of the probe (adapted from ref (49)). Fixed concentrations of the probes (3 μM) were titrated with different FtsZ polymer concentrations (0–40 μM) in HEPES buffer with Mg^2+^, to obtain the anisotropy increment, Δ*r*_max_, corresponding to the bound probe. The increase in anisotropy was plotted against the FtsZ polymer concentration (calculated by subtracting from the total protein concentration the critical concentration for polymerization under the same conditions) and iteratively least-squares fitted with an isotherm of binding to one site. The estimated values of Δ*r*_max_ were used to approximate the free FtsZ concentrations, and these new values were employed again until an unchanging Δ*r*_max_ value was obtained. The convergent data were used to calculate the binding constant of the fluorescent probe to FtsZ polymers (Table S1).

### Affinity of Ligands Competing with the Fluorescent Probes

Competition assays were performed by measuring, through the decrease in fluorescence anisotropy, the displacement of the fluorescent probes from stabilized FtsZ polymers. Increasing concentrations of competing ligands were added to the mixtures of cross-linked FtsZ polymers (10–15 μM) with probe **1** (3 μM), 7–8 μM FtsZ polymers with probe (*S*)-**2** (3 μM), or 2–3 μM FtsZ polymers with (*R*)-**5** (3 μM). HEPES buffer with 10 mM MgCl_2_ and 0.1 mM GMPCPP was employed, with a final volume of 0.5 mL, and the anisotropy measured at 25 °C. Under these conditions, the initial fraction of the probe bound was between 0.4 and 0.5. The fraction of the probe bound (Vb) was calculated
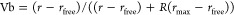
where *r*_free_ and *r*_max_ are the anisotropy values of the free and bound probes, respectively, and *R* is the ratio between the fluorescence intensities of the bound and free probe (Table S1). The binding data were plotted against the competing ligand concentration and fitted by competition equilibrium of the ligand and the probe for the same binding site. The resulting system of equations^50^ was numerically solved using the Equigra v. 5.0 program,^33^ which provided the best-fitted value of the competitor binding constant.

### Cellular Methods

*B. subtilis* 168 and *Staphylococcus* spp. were grown in cation adjusted Mueller–Hinton broth (CAMHB; Becton, Dickinson and Company) at 37 °C. *B. subtilis* SU570 were grown in the same medium at 30 °C. In all cases, the cells were grown to an absorbance of 0.1–0.2 at 600 nm and then incubated either with the compounds or DMSO vehicle. Microscopy assays and cell measurement were performed as previously described.^31^ MIC values were determined by a broth macrodilution method.^51^ MBC values were determined as the lowest concentration of inhibitor at which there was a 99.9% reduction in colony-forming units/mL compared to the inoculum. To construct a *S. aureus* strain expressing FtsZ-mCherry, the plasmid pCN-ftsZmch^9^ was electroporated into RN4220 and 0.1 μM cadmium chloride (Sigma-Aldrich) was used to induce the expression of the construct under the control of the P*cad* promoter.

Isolation of FtsZ-inhibitor-resistant mutants was performed by plating 100 μL of *S. aureus* Mu50 culture (∼10^8^ cells/mL) into CAMHB supplemented with the corresponding FtsZ inhibitor at a concentration higher than the MIC and 2% DMSO. The plates were incubated for 24 h at 37 °C, and several colonies were picked for further analysis. Genomic DNA was extracted from each colony using the Wizard Genomic DNA Purification Kit (Promega) and was used in polymerase chain reactions (PCR) for amplification of *ftsZ* using the primers SaFtsZ-F (5′ TGGCCAATAAAACTAGGAG 3′) and SaFtsZ-R (5′ TGTTATCTGATGATTTGTGTTG 3′). The resulting PCR products were purified using the DNA gel extraction kit (Cultek) and sequenced (Macrogen Inc., Spain). Sequence analysis was performed with BLAST (NCBI).

The sensitivity of the IMR90 cell line to compounds was tested through a standard 3-(4,5-dimethylthiazol-2-yl)-2,5-diphenyltetrazolium bromide (MTT) assay.^52^ The results were reported as the viability percentage of the tested compound relative to vehicle (DMSO), obtained from two or three independent experiments performed in triplicate.

### Crystallization and Structure Determination

Crystallization assays were carried out using purified truncated SaFtsZ(12–316) at 14 mg/mL. For cocrystallization experiments, compounds (at 1–4 mM concentrations, depending on solubility) and 4% (v/v) 1-methyl-2-pyrrolidone (compound solvent; Sigma, analytical standard) were included in the protein solution. Crystals were grown at 295 K by vapor diffusion (sitting drop) under previously employed conditions:^25,34^ 0.2 M lithium sulfate, 10% ethylene glycol, 0.1 M Tris/HCl, pH 8.4–9.0, and 24–28% PEG5000 MME. The crystals employed to solve the structure of the SaFtsZ-DFMBA complex (Table S5) were obtained with 4 mM DFMBA at 27% PEG5000 MME, pH 8.7. Crystal soaking experiments were typically made with 5–20 mM compound and 10–20% (v/v) 1-methyl-2-pyrrolidone for 16–20 h. For the SaFtsZ-**18** and SaFtsZ-**20** complex structures (Table S5), crystals grown in 28% PEG5000 MME, pH 8.6, and 25% PEG5000 MME, pH 8.4, respectively, were soaked in a 10 mM compound and 20% (v/v) 1-methyl-2-pyrrolidone. All crystals were flash-cooled by immersion in liquid nitrogen. Diffraction data were collected at the ALBA synchrotron (Spain) and the ESRF synchrotron (France). All data were processed using XDS^53^ and Aimless from the CCP4 Suite.^51^ Data collection and refinement statistics are presented in Table S5. The structures were determined through molecular replacement using Molrep^54^ and the PDB entry 3VOA as a searching model. Model building and refinement were done using Coot^55^ and PHENIX,^56^ respectively. Refinement statistics are summarized in Table S5. Structural figures were prepared using PyMOL (Schrödinger Inc.).

## Supporting Information Available

Fluorescent benzamide probes synthesized in this work (Figure S1); isotherms of binding of the fluorescent probes **1**, (*S*)-**2**, and (*R*)-**5** to FtsZ polymers measured by fluorescence anisotropy (Figure S2); competition isotherms of weak FtsZ inhibitors from the binding screen (Figure S3); effect of high affinity FtsZ inhibitors on bacterial cell division (Figure S4); expanded view of the SaFtsZ binding cleft, with omit electron density maps (green), contoured at 3σ around compounds DFMBA, **18**, and **20** (Figure S5); ligand interaction diagram of residues located in the binding site (Figure S6); binding parameters of the fluorescent probes to FtsZ-GMPCPP polymers and activity on bacterial cells (Table S1); cell length of *B. subtilis* 168 and diameter of *S. aureus* cells incubated with FtsZ inhibitors (Table S2); cytotoxic effect of FtsZ inhibitors in IMR90 cell line (Table S3); spontaneous mutations of inhibitor-resistant *S. aureus* strains (Table S4); data collection and refinement statistics (Table S5); structure of FtsZ inhibitors TXA707, TXA709 and TXA6101, and fluorescent probe BOFP (Chart S1); structure of previously identified FtsZ inhibitors screened for binding against the interdomain cleft of the protein in this work (Chart S2); synthetic scheme S1; detailed synthetic procedures and characterization data of intermediates **31**–**37**, **39**–**47**, and **50**; final compounds **6–17**, **19**, **21**, **22**–**24** and probes **SP1**–**SP5**; solubility of all tested compounds (Table S6); and NMR spectra of final compounds and HPLC traces analysis (PDF)
Molecular formula strings (CSV)

## Accession Codes

PDB codes 6YD1, 6YD5, and 6YD6 correspond to SaFtsZ in complex with DFMBA, compound **18**, and compound **20**. The authors will release the atomic coordinates and experimental data upon article publication.

## Abbreviations

BsFtsZ: FtsZ from *Bacillus subtilis*
CTPM: 6-(chloro[1,3]thiazolo[5,4-*b*]pyridin-2-yl)methanol
DFMBA: 2,6-difluoro-3-methoxybenzamide
*K*_D_: dissociation constant
MDC: minimal cell division inhibitory concentration
MW: microwave
NBD: 7-nitrobenzoxadiazole
SaFtsZ: FtsZ from *Staphylococcus aureus*

## References

[ref1] Cassini A.; Hogberg L. D.; Plachouras D.; Quattrocchi A.; Hoxha A.; Simonsen G. S.; Colomb-Cotinat M.; Kretzschmar M. E.; Devleesschauwer B.; Cecchini M.; Ouakrim D. A.; Oliveira T. C.; Struelens M. J.; Suetens C.; Monnet D. L.; Attributable deaths and disability-adjusted life-years caused by infections with antibiotic-resistant bacteria in the EU and the European Economic Area in 2015: a population-level modelling analysis. Lancet Infect. Dis. 2019, 19, 56–66. 10.1016/S1473-3099(18)30605-4. 30409683 PMC6300481

[ref2] Foster T. J. Can β-lactam antibiotics be resurrected to combat MRSA?. Trends Microbiol. 2019, 27, 26–38. 10.1016/j.tim.2018.06.005. 30031590

[ref3] World Health Organization. Antibiotic Resistance 2020; WHO, 2020. Can be found under: https://www.who.int/news-room/fact-sheets/detail/antibiotic-resistance.

[ref4] Centers for Disease Control and Prevention. 2019 Antibiotic Resistance Threats Report; WHO, 2019. Can be found under: https://www.cdc.gov/drugresistance/biggest-threats.html.

[ref5] Bi E.; Lutkenhaus J. FtsZ ring structure associated with division in *Escherichia coli*. Nature 1991, 354, 161–164. 10.1038/354161a0. 1944597

[ref6] den Blaauwen T.; Hamoen L.; Levin P. A. The divisome at 25: the road ahead. Curr. Opin. Microbiol. 2017, 36, 85–94. 10.1016/j.mib.2017.01.007. 28254403 PMC6436919

[ref7] Bisson-Filho A. W.; Hsu Y. P.; Squyres G. R.; Kuru E.; Wu F.; Jukes C.; Sun Y.; Dekker C.; Holden S.; VanNieuwenhze M. S.; Brun Y. V.; Garner E. C. Treadmilling by FtsZ filaments drives peptidoglycan synthesis and bacterial cell division. Science 2017, 355, 739–743. 10.1126/science.aak9973. 28209898 PMC5485650

[ref8] Yang X.; Lyu Z.; Miguel A.; McQuillen R.; Huang K. C.; Xiao J. GTPase activity–coupled treadmilling of the bacterial tubulin FtsZ organizes septal cell wall synthesis. Science 2017, 355, 744–747. 10.1126/science.aak9995. 28209899 PMC5851775

[ref9] Monteiro J. M.; Pereira A. R.; Reichmann N. T.; Saraiva B. M.; Fernandes P. B.; Veiga H.; Tavares A. C.; Santos M.; Ferreira M. T.; Macario V.; VanNieuwenhze M. S.; Filipe S. R.; Pinho M. G. Peptidoglycan synthesis drives an FtsZ-treadmilling-independent step of cytokinesis. Nature 2018, 554, 528–532. 10.1038/nature25506. 29443967 PMC5823765

[ref10] Lock R. L.; Harry E. J. Cell-division inhibitors: new insights for future antibiotics. Nat. Rev. Drug Discovery 2008, 7, 324–338. 10.1038/nrd2510. 18323848

[ref11] Schaffner-Barbero C.; Martin-Fontecha M.; Chacon P.; Andreu J. M. Targeting the assembly of bacterial cell division protein FtsZ with small molecules. ACS Chem. Biol. 2012, 7, 269–277. 10.1021/cb2003626. 22047077

[ref12] den Blaauwen T.; Andreu J. M.; Monasterio O. Bacterial cell division proteins as antibiotic targets. Bioorg. Chem. 2014, 55, 27–38. 10.1016/j.bioorg.2014.03.007. 24755375

[ref13] Kusuma K. D.; Payne M.; Ung A. T.; Bottomley A. L.; Harry E. J. FtsZ as an antibacterial target: status and guidelines for progressing this avenue. ACS Infect. Dis. 2019, 5, 1279–1294. 10.1021/acsinfecdis.9b00055. 31268666

[ref14] Haydon D. J.; Stokes N. R.; Ure R.; Galbraith G.; Bennett J. M.; Brown D. R.; Baker P. J.; Barynin V. V.; Rice D. W.; Sedelnikova S. E.; Heal J. R.; Sheridan J. M.; Aiwale S. T.; Chauhan P. K.; Srivastava A.; Taneja A.; Collins I.; Errington J.; Czaplewski L. G. An inhibitor of FtsZ with potent and selective anti-staphylococcal activity. Science 2008, 321, 1673–1675. 10.1126/science.1159961. 18801997

[ref15] Tan C. M.; Therien A. G.; Lu J.; Lee S. H.; Caron A.; Gill C. J.; Lebeau-Jacob C.; Benton-Perdomo L.; Monteiro J. M.; Pereira P. M.; Elsen N. L.; Wu J.; Deschamps K.; Petcu M.; Wong S.; Daigneault E.; Kramer S.; Liang L.; Maxwell E.; Claveau D.; Vaillancourt J.; Skorey K.; Tam J.; Wang H.; Meredith T. C.; Sillaots S.; Wang-Jarantow L.; Ramtohul Y.; Langlois E.; Landry F.; Reid J. C.; Parthasarathy G.; Sharma S.; Baryshnikova A.; Lumb K. J.; Pinho M. G.; Soisson S. M.; Roemer T. Restoring methicillin-resistant Staphylococcus aureus susceptibility to β-lactam antibiotics. Sci. Transl. Med. 2012, 4, 126ra135 10.1126/scitranslmed.3003592. 22440737

[ref16] Casiraghi A.; Suigo L.; Valoti E.; Straniero V. Targeting bacterial cell division: a binding-site centered approach to the most promising inhibitors of the essential protein FtsZ. Antibiotics 2020, 9, 69 10.3390/antibiotics9020069. PMC7167804 32046082

[ref17] Kaul M.; Mark L.; Parhi A. K.; LaVoie E. J.; Pilch D. S. Combining the FtsZ-targeting prodrug TXA709 and the cephalosporin cefdinir confers synergy and reduces the frequency of resistance in methicillin-resistant Staphylococcus aureus. Antimicrob. Agents Chemother. 2016, 60, 4290–4296. 10.1128/AAC.00613-16. 27161635 PMC4914665

[ref18] Taxis Pharmaceuticals Pipeline, 2020. Can be found under: https://www.taxispharma.com/research-development/our-pipeline/.

[ref19] Huecas S.; Llorca O.; Boskovic J.; Martin-Benito J.; Valpuesta J. M.; Andreu J. M. Energetics and geometry of FtsZ polymers: nucleated self-assembly of single protofilaments. Biophys. J. 2008, 94, 1796–1806. 10.1529/biophysj.107.115493. 18024502 PMC2242775

[ref20] Dajkovic A.; Mukherjee A.; Lutkenhaus J. Investigation of regulation of FtsZ assembly by SulA and development of a model for FtsZ polymerization. J. Bacteriol. 2008, 190, 2513–2526. 10.1128/JB.01612-07. 18245292 PMC2293196

[ref21] Miraldi E. R.; Thomas P. J.; Romberg L. Allosteric models for cooperative polymerization of linear polymers. Biophys. J. 2008, 95, 2470–2486. 10.1529/biophysj.107.126219. 18502809 PMC2517016

[ref22] Matsui T.; Han X.; Yu R. J.; Yao M.; Tanaka I. Structural change in FtsZ induced by intermolecular interactions between bound GTP and the T7 loop. J. Biol. Chem. 2014, 289, 3501–3509. 10.1074/jbc.M113.514901. 24347164 PMC3916551

[ref23] Andreu J. M.; Schaffner-Barbero C.; Huecas S.; Alonso D.; Lopez-Rodriguez M. L.; Ruiz-Avila L. B.; Nuñez-Ramirez R.; Llorca O.; Martin-Galiano A. J. The antibacterial cell division inhibitor PC190723 is an FtsZ polymer-stabilizing agent that induces filament assembly and condensation. J. Biol. Chem. 2010, 285, 14239–14246. 10.1074/jbc.M109.094722. 20212044 PMC2863232

[ref24] Elsen N. L.; Lu J.; Parthasarathy G.; Reid J. C.; Sharma S.; Soisson S. M.; Lumb K. J. Mechanism of action of the cell-division inhibitor PC190723: modulation of FtsZ assembly cooperativity. J. Am. Chem. Soc. 2012, 134, 12342–12345. 10.1021/ja303564a. 22793495

[ref25] Matsui T.; Yamane J.; Mogi N.; Yamaguchi H.; Takemoto H.; Yao M.; Tanaka I. Structural reorganization of the bacterial cell-division protein FtsZ from *Staphylococcus aureus*. Acta Crystallogr., Sect. D: Biol. Crystallogr. 2012, 68, 1175–1188. 10.1107/S0907444912022640. 22948918

[ref26] Löwe J.; Amos L. A. Crystal structure of the bacterial cell-division protein FtsZ. Nature 1998, 391, 203–206. 10.1038/34472. 9428770

[ref27] Artola M.; Ruiz-Avila L. B.; Ramirez-Aportela E.; Martinez R. F.; Araujo-Bazan L.; Vazquez-Villa H.; Martin-Fontecha M.; Oliva M. A.; Martin-Galiano A. J.; Chacon P.; Lopez-Rodriguez M. L.; Andreu J. M.; Huecas S. The structural assembly switch of cell division protein FtsZ probed with fluorescent allosteric inhibitors. Chem. Sci. 2017, 8, 1525–1534. 10.1039/C6SC03792E. 28616148 PMC5460597

[ref28] Fujita J.; Harada R.; Maeda Y.; Saito Y.; Mizohata E.; Inoue T.; Shigeta Y.; Matsumura H. Identification of the key interactions in structural transition pathway of FtsZ from Staphylococcus aureus. J. Struct. Biol. 2017, 198, 65–73. 10.1016/j.jsb.2017.04.008. 28456664

[ref29] Wagstaff J. M.; Tsim M.; Oliva M. A.; Garcia-Sanchez A.; Kureisaite-Ciziene D.; Andreu J. M.; Lowe J. A polymerization-associated structural switch in FtsZ that enables treadmilling of model filaments. mBio 2017, 8, e00254-17 10.1128/mBio.00254-17. 28465423 PMC5414002

[ref30] Ramírez-Aportela E.; Lopez-Blanco J. R.; Andreu J. M.; Chacon P. Understanding nucleotide-regulated FtsZ filament dynamics and the monomer assembly switch with large-scale atomistic simulations. Biophys. J. 2014, 107, 2164–2176. 10.1016/j.bpj.2014.09.033. 25418101 PMC4223170

[ref31] Araújo-Bazán L.; Ruiz-Avila L. B.; Andreu D.; Huecas S.; Andreu J. M. Cytological profile of antibacterial FtsZ inhibitors and synthetic peptide MciZ. Front. Microbiol. 2016, 7, 1558 10.3389/fmicb.2016.01558. 27752253 PMC5045927

[ref32] Araújo-Bazán L.; Huecas S.; Valle J.; Andreu D.; Andreu J. M. Synthetic developmental regulator MciZ targets FtsZ across Bacillus species and inhibits bacterial division. Mol. Microbiol. 2019, 111, 965–980. 10.1111/mmi.14198. 30636070

[ref33] Díaz J. F.; Buey R. M. Characterizing ligand-microtubule binding by competition methods. Methods Mol. Med. 2007, 137, 245–260. 10.1007/978-1-59745-442-1_17. 18085234

[ref34] Huecas S.; Canosa-Valls A. J.; Araújo-Bazán L.; Ruiz F. M.; Laurents D. V.; Fernández-Tornero C.; Andreu J. M. Nucleotide-induced folding of cell division protein FtsZ from Staphylococcus aureus. FEBS J. 2020, 287, 4048–4067. 10.1111/febs.15235. 31997533

[ref35] Ruiz-Avila L. B.; Huecas S.; Artola M.; Vergonos A.; Ramirez-Aportela E.; Cercenado E.; Barasoain I.; Vazquez-Villa H.; Martin-Fontecha M.; Chacon P.; Lopez-Rodriguez M. L.; Andreu J. M. Synthetic inhibitors of bacterial cell division targeting the GTP-binding site of FtsZ. ACS Chem. Biol. 2013, 8, 2072–2083. 10.1021/cb400208z. 23855511

[ref36] Andreu J. M.; Barasoain I. The interaction of baccatin III with the taxol binding site of microtubules determined by a homogeneous assay with fluorescent taxoid. Biochemistry 2001, 40, 11975–11984. 10.1021/bi010869+. 11580273

[ref37] Stokes N. R.; Sievers J.; Barker S.; Bennett J. M.; Brown D. R.; Collins I.; Errington V. M.; Foulger D.; Hall M.; Halsey R.; Johnson H.; Rose V.; Thomaides H. B.; Haydon D. J.; Czaplewski L. G.; Errington J. Novel inhibitors of bacterial cytokinesis identified by a cell-based antibiotic screening assay. J. Biol. Chem. 2005, 280, 39709–39715. 10.1074/jbc.M506741200. 16174771

[ref38] Margalit D. N.; Romberg L.; Mets R. B.; Hebert A. M.; Mitchison T. J.; Kirschner M. W.; RayChaudhuri D. Targeting cell division: Small-molecule inhibitors of FtsZ GTPase perturb cytokinetic ring assembly and induce bacterial lethality. Proc. Natl. Acad. Sci. U.S.A. 2004, 101, 11821–11826. 10.1073/pnas.0404439101. 15289600 PMC511058

[ref39] Anderson D. E.; Kim M. B.; Moore J. T.; O’Brien T. E.; Sorto N. A.; Grove C. I.; Lackner L. L.; Ames J. B.; Shaw J. T. Comparison of small molecule inhibitors of the bacterial cell division protein FtsZ and identification of a reliable cross-species inhibitor. ACS Chem. Biol. 2012, 7, 1918–1928. 10.1021/cb300340j. 22958099 PMC3514448

[ref40] Hwang D.; Lim Y. H. Resveratrol antibacterial activity against Escherichia coli is mediated by Z-ring formation inhibition via suppression of FtsZ expression. Sci. Rep. 2015, 5, 10029 10.1038/srep10029. 25942564 PMC4419592

[ref41] Bhattacharya A.; Jindal B.; Singh P.; Datta A.; Panda D. Plumbagin inhibits cytokinesis in *Bacillus subtilis* by inhibiting FtsZ assembly--a mechanistic study of its antibacterial activity. FEBS J. 2013, 280, 4585–4599. 10.1111/febs.12429. 23841620

[ref42] Sun N.; Zheng Y. Y.; Du R. L.; Cai S. Y.; Zhang K.; So L. Y.; Cheung K. C.; Zhuo C.; Lu Y. J.; Wong K. Y. New application of tiplaxtinin as an effective FtsZ-targeting chemotype for an antimicrobial study. MedChemComm 2017, 8, 1909–1913. 10.1039/C7MD00387K. 30108711 PMC6072346

[ref43] Straniero V.; Suigo L.; Casiraghi A.; Sebastián-Pérez V.; Hrast M.; Zanotto C.; Zdovc I.; De Giuli Morghen C.; Radaelli A.; Valoti E. Benzamide derivatives targeting the cell division protein FtsZ: Modifications of the linker and the benzodioxane scaffold and their effects on antimicrobial activity. Antibiotics 2020, 9, 160 10.3390/antibiotics9040160. PMC7235863 32260339

[ref44] Adams D. W.; Wu L. J.; Czaplewski L. G.; Errington J. Multiple effects of benzamide antibiotics on FtsZ function. Mol. Microbiol. 2011, 80, 68–84. 10.1111/j.1365-2958.2011.07559.x. 21276094

[ref45] Qiang S.; Wang C.; Venter H.; Li X.; Wang Y.; Guo L.; Ma R.; Ma S. Synthesis and biological evaluation of novel FtsZ-targeted 3-arylalkoxy-2,6-difluorobenzamides as potential antimicrobial agents. Chem. Biol. Drug. Des. 2016, 87, 257–264. 10.1111/cbdd.12658. 26348110

[ref46] Chai W. C.; Whittall J. J.; Song D.; Polyak S. W.; Ogunniyi A. D.; Wang Y.; Bi F.; Ma S.; Semple S. J.; Venter H. Antimicrobial action and reversal of resistance in MRSA by difluorobenzamide derivatives targeted FtsZ. Antibiotics 2020, 9, 873 10.3390/antibiotics9120873. PMC7762090 33291418

[ref47] Fujita J.; Maeda Y.; Mizohata E.; Inoue T.; Kaul M.; Parhi A. K.; LaVoie E. J.; Pilch D. S.; Matsumura H. Structural flexibility of an inhibitor overcomes drug resistance mutations in Staphylococcus aureus FtsZ. ACS Chem. Biol. 2017, 12, 1947–1955. 10.1021/acschembio.7b00323. 28621933 PMC5705026

[ref48] Ferrer-González E.; Fujita J.; Yoshizawa T.; Nelson J. M.; Pilch A. J.; Hillman E.; Ozawa M.; Kuroda N.; Al-Tameemi H. M.; Boyd J. M.; LaVoie E. J.; Matsumura H.; Pilch D. S. Structure-guided design of a fluorescent probe for the visualization of FtsZ in clinically important gram-positive and gram-negative bacterial pathogens. Sci. Rep. 2019, 9, 20092 10.1038/s41598-019-56557-x. 31882782 PMC6934700

[ref49] Huecas S.; Schaffner-Barbero C.; Garcia W.; Yebenes H.; Palacios J. M.; Diaz J. F.; Menendez M.; Andreu J. M. The interactions of cell division protein FtsZ with guanine nucleotides. J. Biol. Chem. 2007, 282, 37515–37528. 10.1074/jbc.M706399200. 17977836

[ref50] Schaffner-Barbero C.; Gil-Redondo R.; Ruiz-Avila L. B.; Huecas S.; Lappchen T.; den Blaauwen T.; Diaz J. F.; Morreale A.; Andreu J. M. Insights into nucleotide recognition by cell division protein FtsZ from a mant-GTP competition assay and molecular dynamics. Biochemistry 2010, 49, 10458–10472. 10.1021/bi101577p. 21058659

[ref51] Artola M.; Ruiz-Avila L. B.; Vergonos A.; Huecas S.; Araujo-Bazan L.; Martin-Fontecha M.; Vazquez-Villa H.; Turrado C.; Ramirez-Aportela E.; Hoegl A.; Nodwell M.; Barasoain I.; Chacon P.; Sieber S. A.; Andreu J. M.; Lopez-Rodriguez M. L. Effective GTP-replacing FtsZ inhibitors and antibacterial mechanism of action. ACS Chem. Biol. 2015, 10, 834–843. 10.1021/cb500974d. 25486266

[ref52] Marín-Ramos N. I.; Balabasquer M.; Ortega-Nogales F. J.; Torrecillas I. R.; Gil-Ordóñez A.; Marcos-Ramiro B.; Aguilar-Garrido P.; Cushman I.; Romero A.; Medrano F. J.; Gajate C.; Mollinedo F.; Philips M. R.; Campillo M.; Gallardo M.; Martín-Fontecha M.; López-Rodríguez M. L.; Ortega-Gutiérrez S. A potent isoprenylcysteine carboxylmethyltransferase (ICMT) inhibitor improves survival in Ras-driven acute myeloid leukemia. J. Med. Chem. 2019, 62, 6035–6046. 10.1021/acs.jmedchem.9b00145. 31181882

[ref53] Kabsch W. XDS. Acta Crystallogr., Sect. D: Biol. Crystallogr. 2010, 66, 125–132. 10.1107/S0907444909047337. 20124692 PMC2815665

[ref54] Winn M. D.; Ballard C. C.; Cowtan K. D.; Dodson E. J.; Emsley P.; Evans P. R.; Keegan R. M.; Krissinel E. B.; Leslie A. G. W.; McCoy A.; McNicholas S. J.; Murshudov G. N.; Pannu N. S.; Potterton E. A.; Powell H. R.; Read R. J.; Vagin A.; Wilson K. S. Overview of the CCP4 suite and current developments. Acta Crystallogr., Sect. D: Biol. Crystallogr. 2011, 67, 235–242. 10.1107/S0907444910045749. 21460441 PMC3069738

[ref55] Emsley P.; Lohkamp B.; Scott W. G.; Cowtan K. Features and development of Coot. Acta Crystallogr., Sect. D: Biol. Crystallogr. 2010, 66, 486–501. 10.1107/S0907444910007493. 20383002 PMC2852313

[ref56] Adams P. D.; Afonine P. V.; BunkoczNi G.; Chen V. B.; Davis I. W.; Echols N.; Headd J. J.; Hung L.-W.; Kapral G. J.; Grosse-Kunstleve R. W.; McCoy A. J.; Moriarty N. W.; Oeffner R.; Read R. J.; Richardson D. C.; Richardson J. S.; Terwilliger T. C.; Zwart P. H. PHENIX: a comprehensive Python-based system for macromolecular structure solution. Acta Crystallogr., Sect. D: Biol. Crystallogr. 2010, 66, 213–221. 10.1107/S0907444909052925. 20124702 PMC2815670

