# Supplementary material for: Targeting the FtsZ Allosteric Binding Site with a Novel Fluorescence Polarization Screen, Cytological and Structural Approaches for Antibacterial Discovery
Source: J Med Chem. 2021 Apr 28;64(9):5730–45. doi: 10.1021/acs.jmedchem.0c02207 (PMC8478281; doi:10.1021/acs.jmedchem.0c02207)

Supporting Information

Targeting the FtsZ allosteric binding site with a novel fluorescence polarization screen, cytological and structural approaches for antibacterial discovery

Sonia Huecas,◊[a] Lidia Araújo-Bazán,◊[a] Federico M. Ruiz,[a] Laura B. Ruiz-Ávila,[a] R. Fernando Martínez,[b] Andrea Escobar-Peña,[b] Marta Artola,[b] Henar Vázquez-Villa,[b] Mar Martín-Fontecha,[b] Carlos Fernández-Tornero,*[a] María L. López-Rodríguez,*[b] and José M. Andreu*[a]

[a] Centro de Investigaciones Biológicas Margarita Salas CSIC, Ramiro de Maeztu 9, 28040 Madrid, Spain

[b] Dept. Química Orgánica, Facultad de Ciencias Químicas, UCM, Avda. Complutense s/n, 28040 Madrid, Spain

* Corresponding authors: cftornero@cib.csic.es, mluzlr@ucm.es, j.m.andreu@cib.csic.es.

Table of Contents

1. Supplementary Figures, Tables, and Charts S2
2. Supplementary Scheme S11
3. Materials and Methods S12
    3.1. Chemistry S12
    3.2. Solubility of Compounds S34
4. References S35
5. NMR Spectra of Compounds **2-26** and **SP1**-**SP5** S37
6. HPLC Traces Analysis S66

1. Supplementary Figures, Tables, and Charts

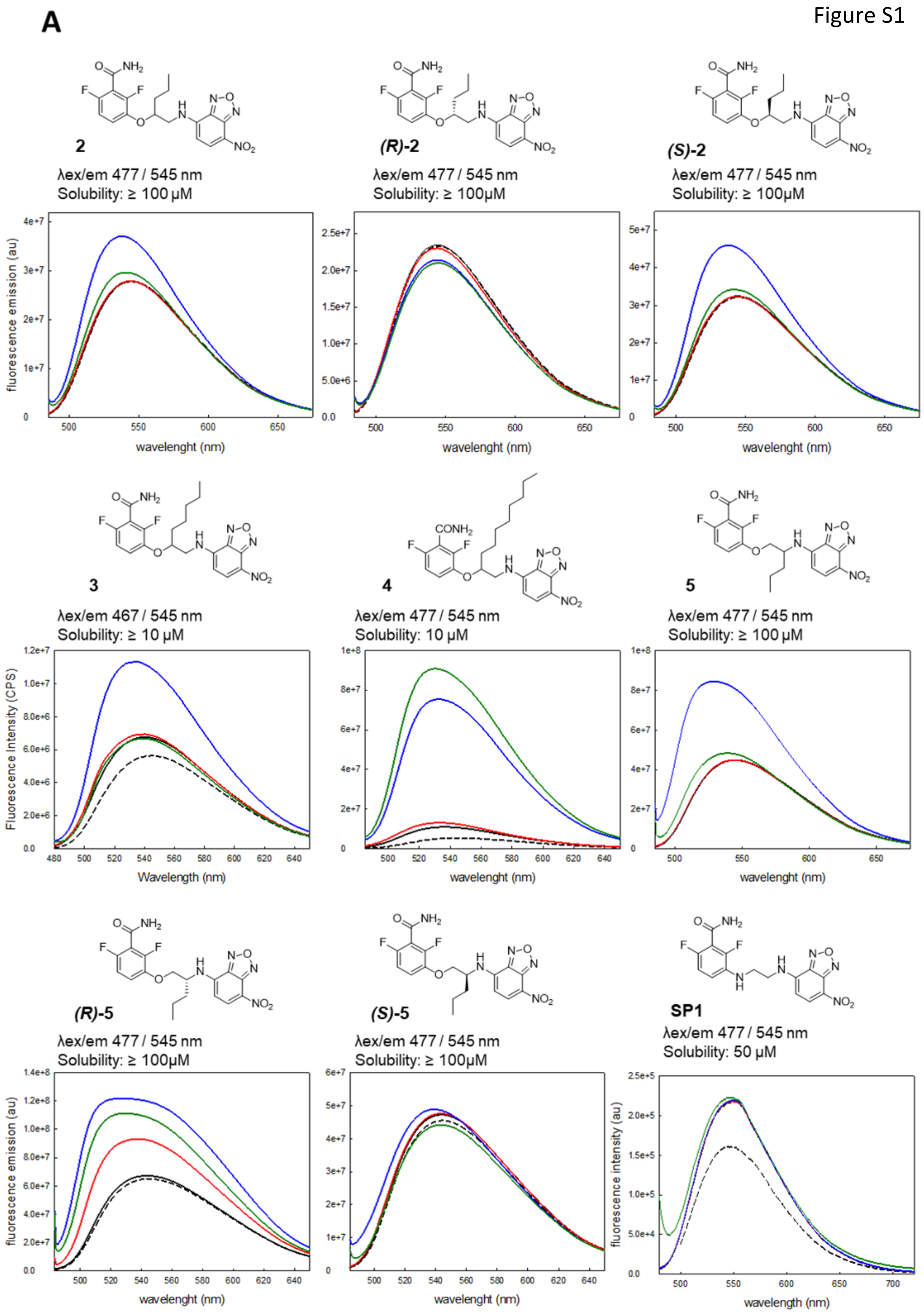

Figure S1 cont.

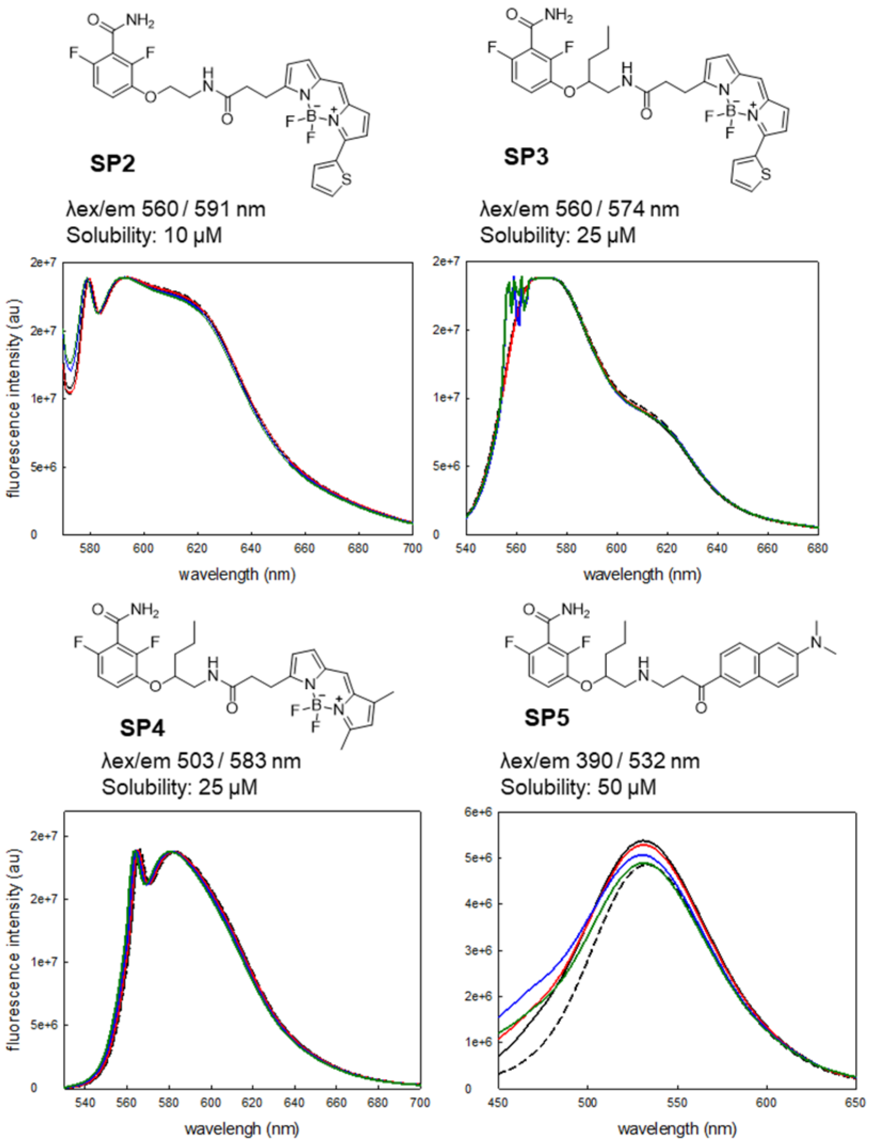

B

	Fluorescence anisotropy (*r*)				
	**SP1**	**SP2**	**SP3**	**SP4**	**SP5**
**Probe**	0.080	0.002	0.005	0.003	0.074
+ **BsFtsZ**	0.084	0.004	0.015	0.004	0.085
+ **GMPCPP**	0.084	0.007	0.034	0.004	0.088
+ **$MgCl_2$**	**0.167**	**0.006**	**0.110**	**0.013**	**0.086**
+ **PC190723**	0.167	0.002	0.035	0.003	0.086

Figure S1. Fluorescent benzamide probes synthesized in this work. A. Chemical structures, solubility in HEPES buffer with 2% DMSO, fluorescence excitation and emission maxima, and emission spectra of each compound alone (10 µM, dashed line), and following consecutive additions of 10 µM BsFtsZ (black line), 0.1 mM GMPCPP (red line), 10 mM $MgCl_2$ (blue line) and 10 µM PC190723 (green line). **B.** Complementary fluorescence anisotropy values of probes **SP1**–**SP5**. All the measurements were performed in 50 mM HEPES-KOH, 50 mM KCl, 1 mM EDTA, pH 6.8, with 2% DMSO.

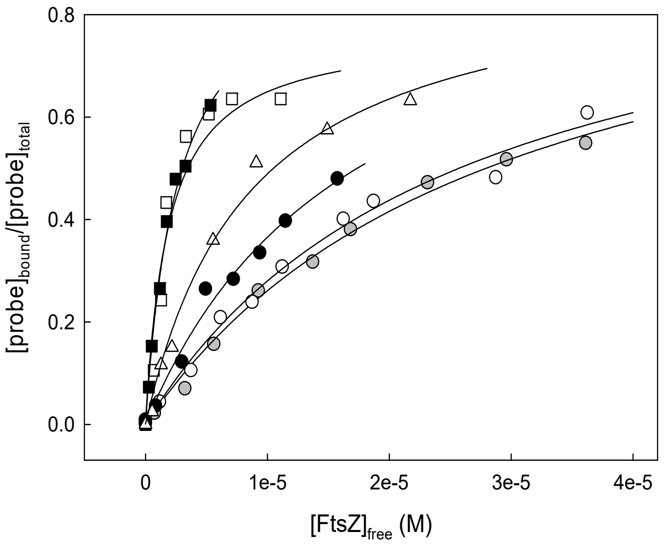

Figure S2. Isotherms of binding of the fluorescent probes 1, (*S*)-2, and (*R*)-5 to FtsZ polymers measured by fluorescence anisotropy. Gray, void and black circles: binding of **1** to FtsZ polymers formed in the presence of GTP (1 mM), GMPCPP (0.1 mM) and GMPCPP (0.1 mM) - stabilized with glutaraldehyde, respectively. Triangles, binding of (*S*)-**2** to FtsZ-GMPCPP polymers. Void and black squares, binding of (*R*)-**5** to FtsZ-GMPCPP polymers and glutaraldehyde-stabilized FtsZ-GMPCPP polymers, respectively. The lines correspond to best fit binding parameters for a one site binding equilibrium (Table S1). The stoichiometry of probe binding to glutaraldehyde-stabilized FtsZ polymers was 0.6 molecules of (*R*)-**5** per FtsZ monomer, spectrophotometrically determined following polymer pelleting (Methods).

Table S1. Binding parameters of the fluorescent probes to FtsZ-GMPCPP polymers and activity on bacterial cells.

	r_{min} [(1)]	r_{max} [(1)]	I_{max}/I_{min} [(1)]	K_D (μM) [(1)]	MDC[(2)](μM) *B. subtilis*	MDC[(2)](μM) *S. aureus Mu50*	MIC[(2)](μM) *B. subtilis*	MIC[(2)](μM) *S. aureus Mu50*
**1**	0.024	0.213	1.0	26 ± 3	200[(4)]	>200[(4)]	800[(4)]	n.d.[(5)]
**1**[(3)]	0.024	0.26	1.0	17 ± 1				
S-**2**	0.028	0.258	2.4	8 ± 1	100	>100	250	>250
R-**2**	0.038	0.067	1.0	n.d.[(5)]	>100	>100	>250	>250
S-**5**	0.025	0.178	1.7	15 ± 2	>50	>100	250	>250
R-**5**	0.029	0.272	6.2	1.9 ± 0.6	25	>100	25	>250[(6)]
R-**5**[(3)]	0.029	0.268	5.0	2.0 ± 0.5				

[(1)] r_{min} (free probe), r_{max} (all probe bound), I_{max}/I_{min} and K_D are best fit values from binding titrations (Fig. S2 and Methods); r_{max} is thus different from the anisotropy values corresponding partially bound probe in Figure 2A. [(2)] MDC, minimal division inhibitory concentration; MIC, minimal growth inhibitory concentration. [(3)] Binding to stabilized FtsZ polymers. [(4)] See reference 1. [(5)] n.d., not determined. [(6)] 250 μM (*R*)-**5** partially inhibited growth.

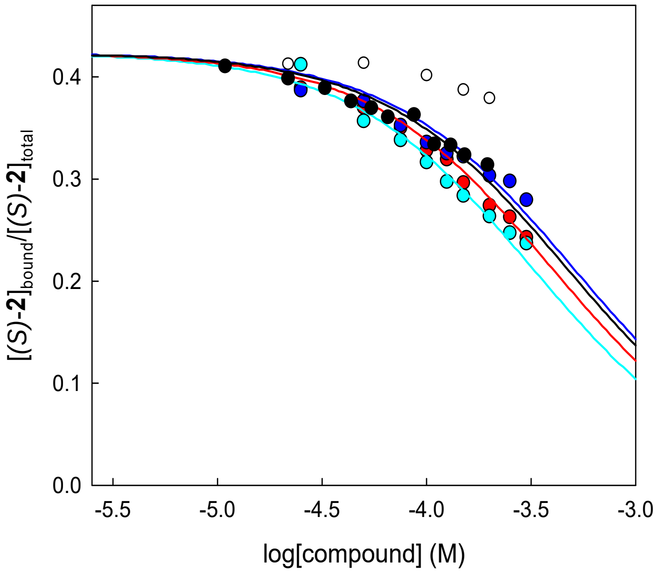

Figure S3. Competition isotherms of weak FtsZ inhibitors from the binding screen. Displacement curves of (*S*)-**2** (3 µM) from stabilized FtsZ-GMPCPP polymers (8 µM) by **6** (black, K_D = 233 ± 25 µM), **7** (cyan, K_D = 159 ± 18 µM), **9** (red symbols, K_D = 196 ± 12 µM) and **10** (blue, K_D = 250 ± 20 µM); equivalent volume of DMSO was added as controls (void circles). The lines correspond to the best fit of each competition curve in each case.

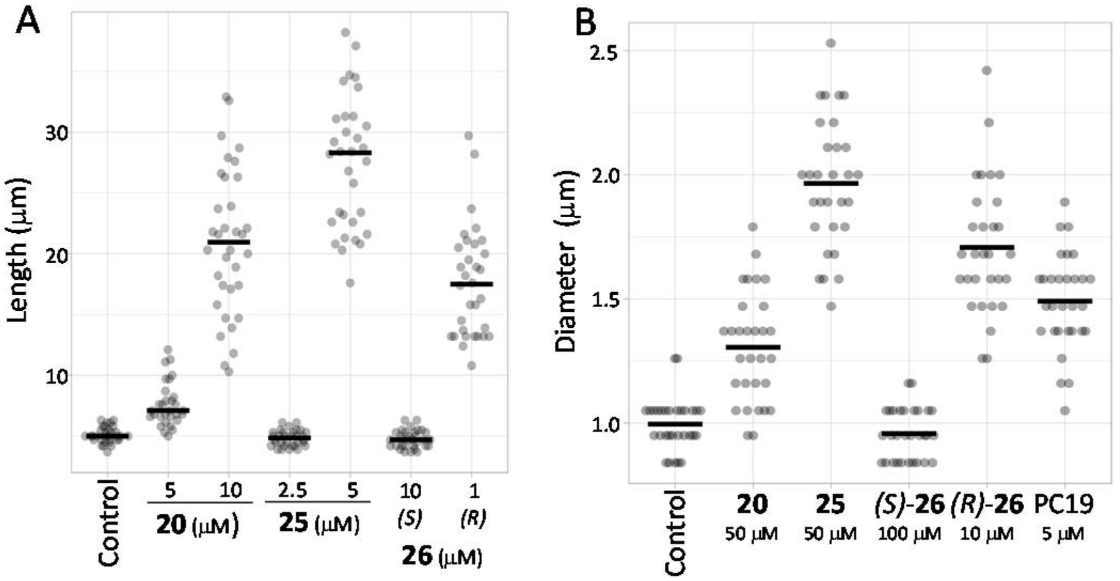

Figure S4. Effect of high affinity FtsZ inhibitors on bacterial cell division. Raw data plots with mean values marked (n ≥ 30). **A**. Cells of *B. subtilis* were incubated during 3 h with compounds **20**, **25** and **26** and their effects on cell division were quantified by measuring the cell length. **B**. Cells of *S. aureus* were incubated during 3 h with compounds **20**, **25**, **26**, or PC190723 and their effects on cell division were quantified by measuring the diameter of the cells.

Table S2. Cell length of *B. subtilis* 168 and diameter of *S. aureus* cells incubated with FtsZ inhibitors.[a]

B. subtilis 168							
	Control	**6** 0.5 mM	**6** 0.75 mM	**7** 0.5 mM	**7** 0.75 mM	**9** 0.5 mM	**9** 0.75 mM
length (μm)	5.1 ± 0.1	5.9 ± 0.3	8.0 ± 0.2	8.5 ± 0.3	24.7 ± 0.8	6.1 ± 0.3	11.6 ± 0.4
	**20** 5 μM	**20** 10 μM	**25** 2.5 μM	**25** 5 μM	(*S*)-**26** 10 μM	(*R*)-**26** 1 μM	
length (μm)	7.6 ± 0.3	20.9 ± 1.0	4.8 ± 0.1	27.4 ± 0.9	4.9 ± 0.2	17.6 ± 0.8	
S. aureus							
	Control	**20** 50 μM	**25** 50 μM	(*S*)-**26** 100 μM	(*R*)-**26** 10 μM	PC19 5 μM	
diameter (μm)	1.00 ± 0.02	1.31 ± 0.03	1.96 ± 0.04	0.96 ± 0.01	1.71 ± 0.04	1.49 ± 0.03	

[a] Mean and standard error are shown in all cases (n ≥ 30)

Table S3. Cytotoxic effect of FtsZ inhibitors in IMR90 cell line.[a]

Compd	Concentration (μM)	Viability (%)
**18**	100	90 ± 7
**20**	50	84 ± 5
**25**	100	88 ± 1
(R)-**26**	25	100 ± 1

[a] Data from two to three independent experiments performed in triplicate.

Table S4. Spontaneous mutations of inhibitor-resistant *S. aureus* strains.[a]

Strain	**20**<br>(2xMIC, 100 µM[b])<br>(FOR[c] 2.1 x 10^{-6})	**25**<br>(1.5xMIC,150 µM)<br>(FOR 1.6 x 10^{-6})	***(R)*-26**<br>(4xMIC, 100 µM)<br>(FOR 2.5 x 10^{-6})	**PC190723**<br>(8xMIC, 23 µM)<br>(FOR 2.0 x 10^{-8})
**# 1**	G196S<br>known site, H7	M262I<br>cleft, S8	L261V<br>cleft, S8	G196S<br>known site
**# 2**	L249V cleft top<br>loop H9-S8	M262I, T358P<br>cleft, off cleft C-tail	V214I known site<br>cleft back, H8	N263K<br>known site
**# 3**	M262I<br>Cleft, S8	M262I, N263Y<br>cleft, cleft, S8	not in *ftsZ*	L200I<br>known site
**# 4**	A285V off cleft,<br>c-ter H10	P115L<br>off cleft, N-ter H4	V151L<br>off cleft, N-ter H5	
**# 5**	not in *ftsZ*	A237L<br>off cleft, C-ter H9	V297I<br>cleft bottom, S9	
**# 6**	T309I<br>cleft, S10	A182T<br>cleft top, H7	V151L<br>off cleft, N-ter H5	

[a] The corresponding aminoacid change in FtsZ is indicated. [b] Concentrations were limited by compounds solubility in culture medium. [c] FOR: frequency of resistance

Table S5. Data collection and refinement statistics.

	SaFtsZ-DFMBA	SaFtsZ-18	SaFtsZ-20
**Wavelength (Å)**	0.97901	0.97895	0.97918
**Resolution range (Å)**	41.15 - 1.70 (1.76 - 1.70)	40.24 - 1.55 (1.60 - 1.55)	40.16 - 1.70 (1.76 - 1.70)
**Space group**	C 1 2 1	C 1 2 1	C 1 2 1
**Unit cell: a, b, c (Å)** α, β, γ (°)	71.3 51.1 87.8 90 110.44 90	72.4 50.1 88.6 90 111.49 90	71.7 50.1 88.3 90 111.06 90
**Total reflections**	119014 (6349)	134018 (7039)	95229 (4992)
**Unique reflections**	32199 (3208)	41526 (4196)	31638 (3192)
**Multiplicity**	3.7 (1.9)	3.2 (1.7)	3.0 (1.6)
**Completeness (%)**	98.20 (98.34)	96.23 (97.24)	97.43 (98.37)
**Mean I/sigma(I)**	13.79 (1.89)	5.76 (1.78)	9.80 (2.03)
**Wilson B-factor**	25.49	19.95	23.65
**R-merge**	0.02 (0.34)	0.06 (0.25)	0.03 (0.30)
**R-meas**	0.03 (0.48)	0.09 (0.36)	0.04 (0.43)
**R-pim**	0.02 (0.34)	0.06 (0.25)	0.03 (0.30)
**CC1/2**	0.99 (0.84)	0.98 (0.90)	0.99 (0.82)
**CC***	1 (0.95)	0.99 (0.97)	0.99 (0.94)
**Reflections used in refinement**	32182 (3200)	41500 (4187)	31631 (3191)
**Reflections used for R-free**	1550 (144)	2034 (196)	1529 (164)
**R-work**	0.17 (0.27)	0.18 (0.22)	0.16 (0.24)
**R-free**	0.21 (0.33)	0.23 (0.28)	0.19 (0.30)
**CC(work)**	0.96 (0.87)	0.96 (0.93)	0.97 (0.85)
**CC(free)**	0.96 (0.72)	0.91 (0.90)	0.95 (0.81)
**Number of non-hydrogen atoms**	2467	2566	2513
**macromolecules**	2218	2281	2248
**ligands**	42	60	63
**solvent**	207	225	202
**Protein residues**	305	307	307
**RMS(bonds)**	0.014	0.005	0.011
**RMS(angles)**	1.39	0.86	1.20
**Ramachandran favored (%)**	98.35	98.69	98.36
**Ramachandran allowed (%)**	1.65	1.31	1.64
**Ramachandran outliers (%)**	0.00	0.00	0.00
**Rotamer outliers (%)**	0.86	0.41	0.42
**Clashscore**	7.51	2.99	2.81
**Average B-factor**	32.83	28.37	29.41
**macromolecules**	32.27	27.54	28.81
**ligands**	23.84	25.13	23.64
**solvent**	40.72	37.63	37.86
**PDB code**	6YD1	6YD5	6YD6

Statistics for the highest-resolution shell are shown in parentheses.

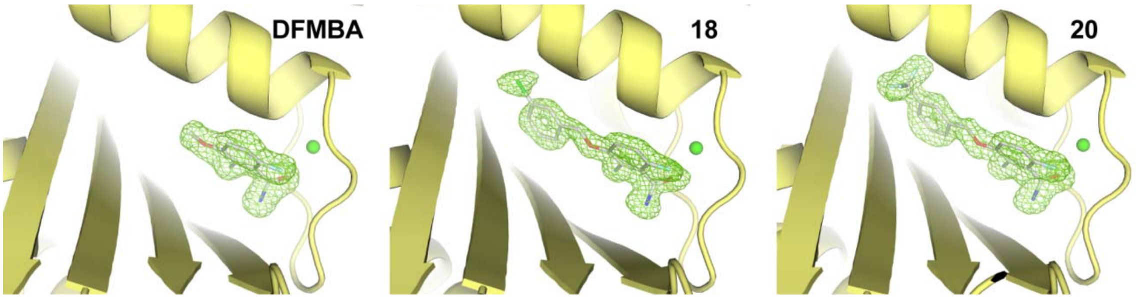

Figure S5. Expanded view of the SaFtsZ binding cleft, with omit electron density maps (green), contoured at 3 sigma around compounds DFMBA, **18** and **20**.

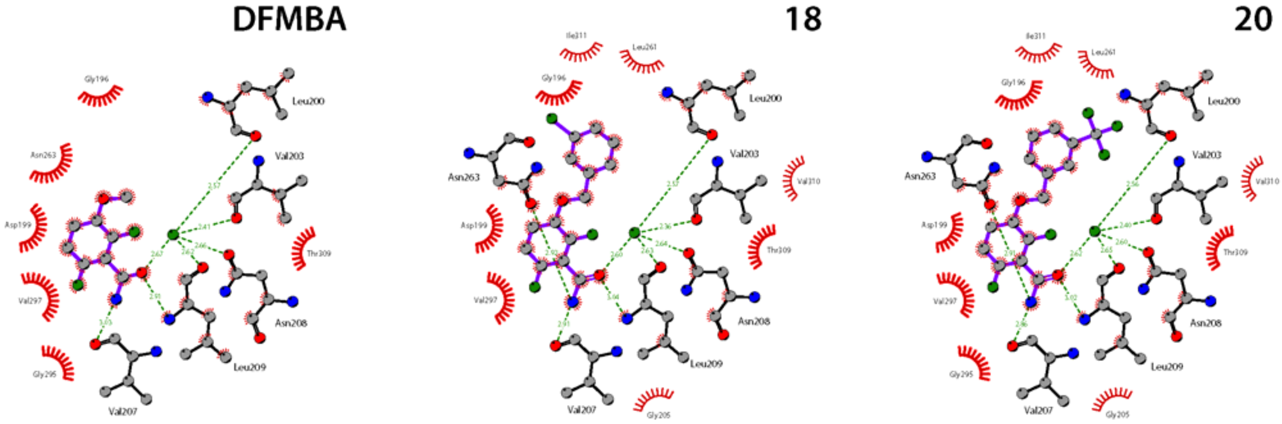

Figure S6. Ligand interaction diagram of residues located in the binding site. Ligands DFMBA, **18**, and **20** and protein bonds are in purple and black, respectively. Green dotted lines depict metal coordination or hydrogen bonds with distances in ångström, while hydrophobic contacts are shown as brown flashes around an atom or residue. The figure was prepared with LIGPLOT.[2]

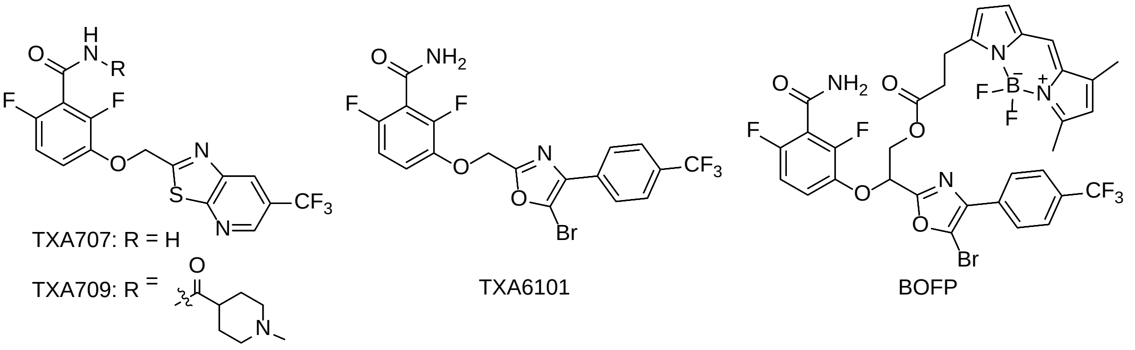

Chart S1. Structure of FtsZ inhibitors TXA707, TXA709 and TXA6101, and fluorescent probe BOFP.

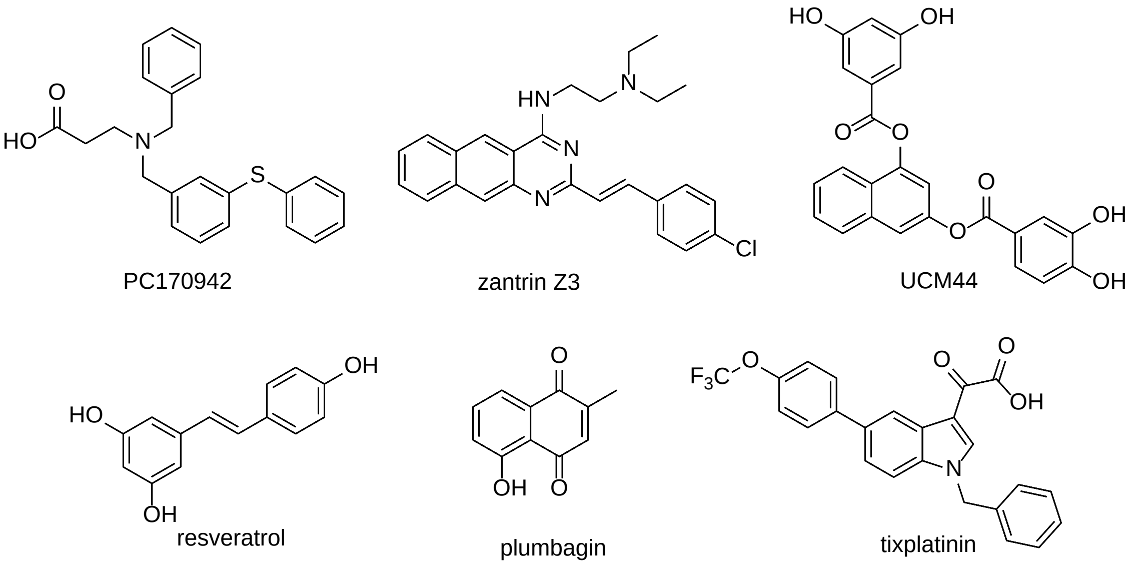

Chart S2. Structure of previously identified FtsZ inhibitors screened for binding against the interdomain cleft of the protein in this work.

2. Supplementary Scheme S1

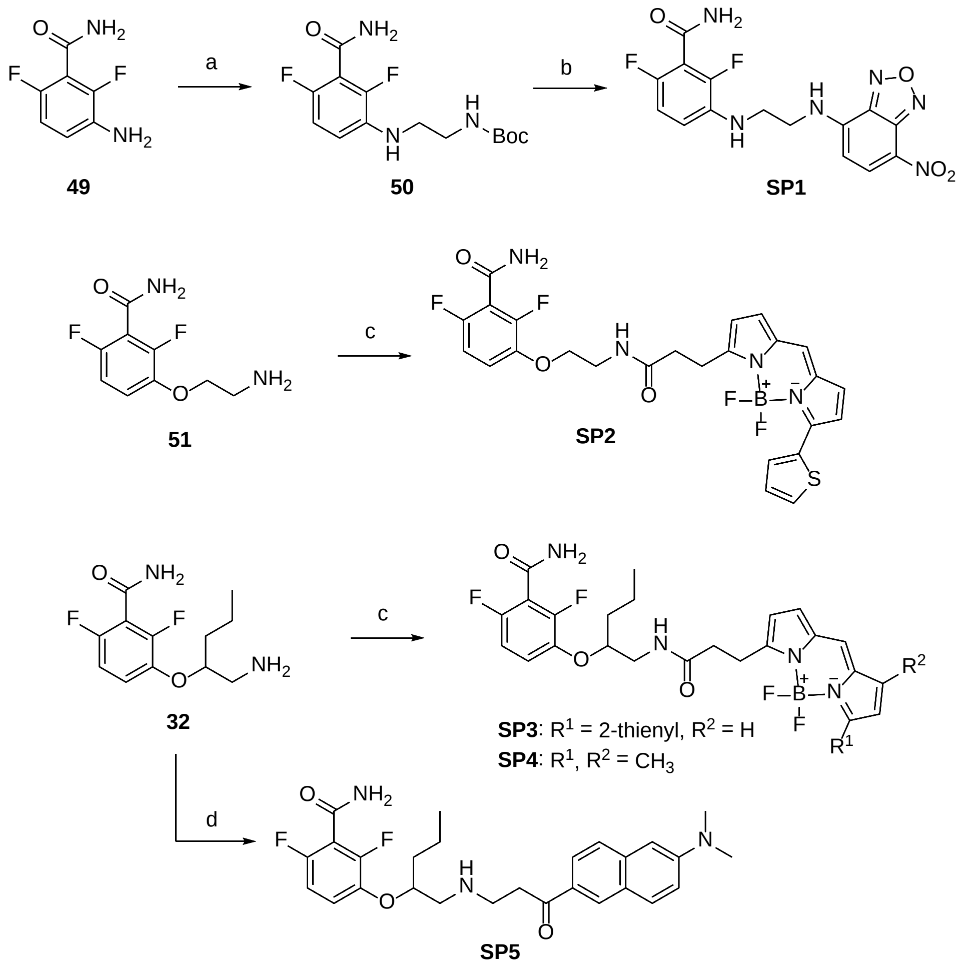

Scheme S1. Reagents and conditions: a) i. *N*-Boc-2-aminoacetaldehyde, AcOH, MeOH, rt, 2 h; ii. $NaBH_3CN$, rt, 24 h, 64%; b) i. TFA, DCM, rt, 2 h, quant.; ii. Cl-NBD, Cs_2CO_3, MeCN, 80 ºC, 2 h, 47%; c) BODIPY-558/568 NHS ester or BODIPY-FL NHS ester, Et_3N, DCM, rt, 24 h, 24-95%; d) acrylodan, EtOH, 65 ºC, 24 h, 32%.

3. Materials and Methods

3.1. Chemistry

The following compounds were synthesized as previously described and their spectroscopic data correspond with those reported: 2,6-difluoro-3-{2-[(7-nitro-2,1,3-benzoxadiazol-4-yl)amino]ethoxy}benzamide (**1**),[3] 2,6-difluoro-3-hydroxybenzamide (**27**),[4] *N*-Boc-2-bromopentylamine (**28**),[5] *N*-Boc-2-bromoheptylamine (**29**),[5] *N*-Boc-2-bromodecylamine (**30**),[5] 2-(Boc-amino)-1-pentanol (**38**),[6] 3-(dimethylamino)-1-[3-(trifluoromethyl)phenyl]propan-1-one (**48**),[7] 3-amino-2,6-difluorobenzamide (**49**),[8] and 3-(2-aminoethoxy)-2,6-difluorobenzamide (**51**).[1]

- **General Procedure for the Synthesis of Racemic Compounds 32-34.** To a solution of compound **27** (1 equiv), K_2CO_3 (3 equiv) and NaI (0.2 equiv) in anhydrous DMF (10 mL/mmol), a solution of the corresponding bromo derivative **28-30** (1 equiv; 1 mL/mmol) in anhydrous DMF was added dropwise. The reaction mixture was stirred at rt overnight. Then, the reaction was concentrated under reduced pressure and the residue was dissolved in EtOAc and washed with a saturated aqueous solution of $NaHCO_3$ and with water. The organic layer was dried (Na_2SO_4), filtered and the solvent was evaporated under reduced pressure. The crude was purified by chromatography (from hexane to EtOAc) to afford the corresponding intermediates **32-34**.

3-[(1-*N*-Boc-Aminopentan-2-yl)oxy]-2,6-difluorobenzamide (32). Obtained from **27** (162 mg, 0.94 mmol) and *N*-Boc-2-bromopentylamine (**28**) (249 mg, 0.94 mmol) as an oil in 15% yield (48 mg).

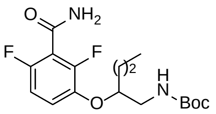

R_f (hexane/EtOAc, 2:1) 0.50; IR (ATR) ν 3376 (NH), 1683 (CO), 1612, 1509, 1490 (Ar); ^{1}H NMR (700 MHz, $CDCl_3$) δ 0.93 (t, J = 7.5, 3H, CH_3), 1.43 (s, 11H, $(CH_3)_3C$, CH_2), 1.55-1.60 (m, 1H, ½CH_2), 1.64-1.70 (m, 1H, ½CH_2), 3.25-3.29 (m, 1H, ½CH_2N), 3.42-3.44 (m, 1H, ½CH_2N), 4.29 (m, 1H, CHO), 4.89 (br s, 1H, NH), 6.02 (br s, 2H, NH_2), 6.86-6.89 (m, 1H, H_5), 7.07-7.11 (m, 1H, H_4); ^{13}C NMR (175 MHz, $CDCl_3$) δ 14.2 (CH_3), 18.6 (CH_2), 28.5 ((*C*H_3)$_3$C), 34.1 (CH_2), 43.9 (CH_2N), 79.8 ((CH_3)$_3$*C*), 80.6 (CHO), 111.5 (dd, J_{C-F} = 23.1, 4.0, C_5), 114.1 (dd, J_{C-F} = 14.0, 9.0, C_1), 120.3 (dd, J_{C-F} = 9.9, 2.3, C_4), 143.3 (dd, J_{C-F} = 10.9, 3.2, C_3), 151.2 (dd, J_{C-F} = 254.0, 7.7, CF), 154.0 (dd, J_{C-F} = 247.5, 5.0, CF), 156.2 (NHCO), 162.1 ($CONH_2$); ESI-MS 259.2 $[M-Boc+H]^+$.

3-[(1-*N*-Boc-Aminoheptan-2-yl)oxy]-2,6-difluorobenzamide (33). Obtained from **27** (176 mg, 1.0 mmol) and *N*-Boc-2-bromoheptylamine (**29**) (294 mg, 1.0 mmol) as an oil in 14% yield (56 mg).

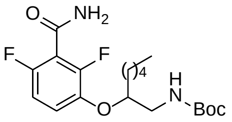

R_f (hexane/EtOAc, 1:2) 0.69; IR (ATR) ν 3380 (NH), 1684 (CO), 1519, 1489 (Ar); ^{1}H NMR (700 MHz, $CDCl_3$) δ 0.86-0.90 (m, 3H, CH_3), 1.28-1.31 (m, 4H, $2CH_2$), 1.42 (s, 11H, $(CH_3)_3C$, CH_2), 1.56-1.61 (m, 1H, ½CH_2), 1.64-1.70 (m, 1H, ½CH_2), 3.24-3.28 (m, 1H, ½CH_2N), 3.42-3.44 (m, 1H, ½CH_2N), 4.26 (m, 1H, CHO), 4.91 (br s, 1H, NH), 6.06 (br s, 1H, ½NH_2), 6.22 (br s, 1H, ½NH_2), 6.87 (app t, J = 9.0, 1H, H_5), 7.06-7.09 (m, 1H, H_4); ^{13}C NMR (175 MHz, $CDCl_3$) δ 14.1 (CH_3), 22.6 (CH_2), 24.9 (CH_2), 28.5 ((CH$_3$)$_3$C), 31.6 (CH_2), 31.9 (CH_2), 43.9 (CH_2N), 79.7 ((CH_3)$_3$$C$), 80.8 (CHO), 111.5 (dd, $J_{C\text{-}F}$ = 23.5, 3.4, C_5), 114.2 (t, $J_{C\text{-}F}$ = 18.7, C_1), 120.3 (d, $J_{C\text{-}F}$ = 8.8, C_4), 143.2 (dd, $J_{C\text{-}F}$ = 11.1, 3.4, C_3), 151.1 (dd, $J_{C\text{-}F}$ = 254.0, 7.0, CF), 154.0 (d, $J_{C\text{-}F}$ = 247.4, 4.6, CF), 156.2 (NHCO), 162.2 ($CONH_2$); ESI-MS 287.2 $[M\text{-}Boc+H]^+$.

3-[(1-*N*-Boc-Aminodecan-2-yl)oxy]-2,6-difluorobenzamide (34). Obtained from **27** (300 mg, 1.7 mmol) and *N*-Boc-2-bromodecylamine (**30**) (403 mg, 1.2 mmol) as an oil in 23% yield (115 mg).

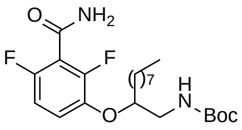

R_f (hexane/EtOAc, 6:4) 0.53; IR (ATR) ν 3356 (NH), 1681 (C=O), 1489, 1367 (Ar); ^{1}H NMR (300 MHz, $CDCl_3$) δ 0.85 (t, J = 6.7, 3H, CH_3), 1.24 (br s, 10H, $5CH_2$), 1.41-1.42 (m, 11H, CH_2, $(CH_3)_3C$), 1.54-1.69 (m, 2H, CH_2), 3.22-3.29 (m, 1H, ½CH_2N), 3.39-3.43 (m, 1H, ½CH_2N), 4.25 (br s, 1H, CHO), 4.96 (br s, 1H, NH), 6.16 (br s, 1H, ½NH_2), 6.58 (br s, 1H, ½NH_2), 6.84 (td, J = 9.1, 1.8, 1H, H_5), 7.38 (td, J = 8.9, 5.3 1H, H_4); ^{13}C NMR (75 MHz, $CDCl_3$) δ 14.5 (CH_3), 23.0, 25.5 ($2CH_2$), 28.7 ((CH$_3$)$_3$C), 29.6, 29.8, 30.0 ($3CH_2$), 32.2 ($2CH_2$), 44.1 (CH_2N), 80.0 ((CH_3)$_3$$C$), 81.1 (CHO), 111.7 (dd, $J_{C\text{-}F}$ = 23.3, 4.3, C_5), 114.6 (dd, $J_{C\text{-}F}$ = 20.5, 16.8, C_1), 120.5 (dd, $J_{C\text{-}F}$ = 9.9, 2.3, C_4), 143.3 (dd, $J_{C\text{-}F}$ = 11.2, 3.3, C_3), 151.3 (dd, $J_{C\text{-}F}$ = 253.9, 6.9, CF), 154.7 (dd, $J_{C\text{-}F}$ = 256.6, 7.1, CF), 156.5 (NHCO), 162.8 ($CONH_2$); ESI-MS 328.2 $[M+H]^+$.

- **Enantioselective Synthesis of (*R*)- and (*S*)-32.**

General Procedure for the Synthesis of (*R*)- and (*S*)-31. To an open-air solution of the (*R*,*R*)- or (*S*,*S*)-(salen)-Co(II) complex [(*R*,*R*)- or (*S*,*S*)-(*N*,*N′*-bis(3,5-di-*tert*-butylsalicylidene)-1,2-cyclohexanediaminocobalt(II)] (4.4 mol%) in undistilled methyl

tert-butyl ether (TBME, 0.3 mL/mmol) in a vial, *p*-nitrobenzoic acid (8.8 mol%) was added. The vial was closed with a rubber stopper and the mixture was stirred at rt till the red color turned to dark brown. Next, *tert*-butyl carbamate (1 equiv) and TBME (0.1 mL/mmol) were added. After 5 min stirring, 1,2-epoxypentane (2.2 equiv) was added dropwise and stirring was continued at rt for 24 h. Then, the mixture was flushed through a plug of silica gel and the solvent was removed under reduced pressure. The residue was purified by chromatography (from DCM to DCM:EtOAc 7:3) to afford the corresponding highly enantioenriched *N*-protected 1,2-aminoalcohol.

(*R*)-*tert*-Butyl (2-hydroxypentyl)carbamate [(*R*)-31]. Obtained from *tert*-butyl carbamate (308 mg, 2.6 mmol) and (*S*,*S*)-(salen)-Co(II) complex (70 mg, 0.12 mmol) as an oil in 61% yield (325 mg). The spectroscopic data were in agreement with those previously reported.[9]

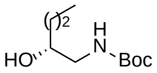

$[\alpha]_D^{24}$ = – 11.5 (c = 0.24, $CHCl_3$) [lit.:[9] $[\alpha]_D^{24}$ = – 13.2 (c = 0.98, $CHCl_3$, for *ee* ≥ 96%)

(*S*)-*tert*-Butyl (2-hydroxypentyl)carbamate [(*S*)-31]. Obtained from *tert*-butyl carbamate (308 mg, 2.6 mmol) and (*R*,*R*)-(salen)-Co(II) complex (70 mg, 0.12 mmol) as an oil in 56% yield (300 mg). The spectroscopic data were in agreement with those reported for enantiomer *R*.

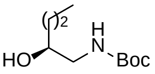

$[\alpha]_D^{24}$ = + 12.0 (c = 0.24, $CHCl_3$)

General Procedure for the Synthesis of (*R*)- and (*S*)-32. To a solution of compound **27** (1 equiv) in anhydrous DMF (10 mL/mmol), (*S*)- or (*R*)-**31** (1 equiv), tributylphosphine (1 equiv), and diisopropyl azodicarboxylate (1 equiv) were added and the reaction mixture was stirred under MW irradiation at 150 ºC from 2 h. Then, the mixture was diluted with EtOAc and the organic layer was washed with brine (3x20 mL), dried (Na_2SO_4), and filtered. The solvents were removed under reduced pressure and the residue was purified by chromatography (from hexane to hexane:EtOAc 6:4) to yield (*R*)- or (*S*)-**32**.

(*R*)-3-[(1-*N*-Boc-Aminopentan-2-yl)oxy]-2,6-difluorobenzamide [(*R*)-32].

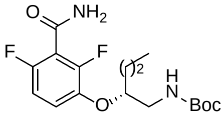

Obtained from **27** (244 mg, 1.4 mmol) and (*S*)-**31** (287 mg, 1.4 mmol) as an oil in 28% yield (140 mg). The spectroscopic data were

in agreement with those described for *rac*-**32**.

(*S*)-3-[(1-*N*-Boc-Aminopentan-2-yl)oxy]-2,6-difluorobenzamide [(*S*)-32].

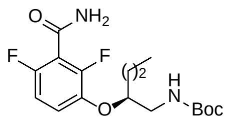

Obtained from **27** (298 mg, 1.7 mmol) and (*R*)-**31** (350 mg, 1.7 mmol) as an oil in 31% yield (190 mg). The spectroscopic data were in agreement with those described for *rac*-**32**.

- **General Procedure for the Synthesis of *rac*-, (*R*)-, and (*S*)-39.** To a solution of **27** (1 equiv) in anhydrous DMF (5 mL/mmol), alcohol *rac*-, (*R*)-, or (*S*)-**38** (1 equiv), tributylphosphine (1 equiv), and diisopropyl azodicarboxylate (1 equiv) were added, and the reaction mixture was stirred at 80 ºC for 48 h. Then, the mixture was diluted with EtOAc (20 mL) and the organic layers were washed with brine (3x20 mL), dried (Na_2SO_4), and filtered. The solvents were removed under reduced pressure and the residue was purified by chromatography (from hexane to hexane:EtOAc 6:4) to yield *rac*-, (*R*)-, or (*S*)-**39**.

3-[(2-*N*-Boc-aminopentyl)oxy]-2,6-difluorobenzamide (39). Obtained from **27** (400 mg, 2.3 mmol) and racemic alcohol **38** (466 mg, 2.3 mmol) as an oil in 18% yield (147 mg).

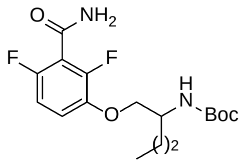

R_f (hexane/EtOAc, 1:2) 0.58; IR (ATR) ν 3317, 3192 (NH), 1676 (CO), 1627, 1492, 1461 (Ar); ^{1}H NMR (700 MHz, $CDCl_3$) δ 0.94 (t, *J* = 7.5, 3H, CH_3), 1.34-1.41 (m, 2H, CH_2), 1.44 (s, 9H, $(CH_3)_3C$), 1.54-1.59 (m, 1H, ½CH_2), 1.63-1.66 (m, 1H, ½CH_2), 3.90 (m, 1H, CHN), 4.00 (m, 2H, CH_2O), 4.75 (d, *J* = 8.5, 1H, NH), 6.06 (br s, 1H, ½NH_2), 6.20 (br s, 1H, ½NH_2), 6.87 (td, *J* = 9.1, 1.5, 1H, H_5), 7.01-7.04 (m, 1H, H_4); ^{13}C NMR (175 MHz, $CDCl_3$) δ 14.0 (CH_3), 19.4 (CH_2), 28.5 ((CH$_3$)$_3$C), 33.9 (CH_2), 49.9 (CHN), 72.1 (CH_2O), 79.7 ((CH_3)$_3$*C*), 111.3 (dd, $J_{C\text{-}F}$ = 23.5, 4.0, C_5), 114.1 (dd, $J_{C\text{-}F}$ = 20.5, 16.5, C_1), 117.3 (d, $J_{C\text{-}F}$ = 8.5, C_4), 144.0 (dd, $J_{C\text{-}F}$ = 11.1, 3.0, C_3), 150.2 (dd, $J_{C\text{-}F}$ = 255.0, 6.5, CF), 153.7 (dd, $J_{C\text{-}F}$ = 246.5, 3.0, CF), 155.7 (NHCO), 162.2 ($CONH_2$); ESI-MS 259.2 $[M\text{-}Boc+H]^+$.

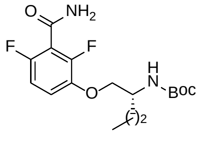

(*R*)-39. Obtained from **27** (675 mg, 3.9 mmol) and (*R*)-**38** (793 mg, 3.9 mmol) as an oil in 18% yield (250 mg). The spectroscopic data were in agreement with those described for *rac*-**39.**

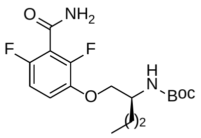

(*S*)-39. Obtained from **27** (395 mg, 2.3 mmol) and (*S*)-**38** (464 mg, 2.3 mmol) as an oil in 19% yield (150 mg). The spectroscopic data were in agreement with those described for *rac*-**39**.

- **General Procedure for the Synthesis of 35-37 and 40.** To a solution of Boc derivatives **32-34, 39** (1 equiv) in anhydrous DCM (10 mL/mmol), trifluoroacetic acid (TFA) (20 equiv) was added dropwise and the reaction mixture was stirred at rt for 1 h. Then, the mixture was concentrated under reduced pressure and the residue was dissolved in DCM and neutralized with a saturated aqueous solution of $NaHCO_3$. The aqueous phase was then extracted with DCM and the organic layer was dried (Na_2SO_4), filtered, and concentrated under reduced pressure to afford amines **35-37** and **40**, which were purified by chromatography (DCM to DCM:MeOH 8:2).

3-[(1-Aminopentan-2-yl)oxy]-2,6-difluorobenzamide (*rac*-35). Obtained from **32** (40 mg, 0.11 mmol) in 83% yield (24 mg). ESI-MS 258.1 $[M+H]^+$.

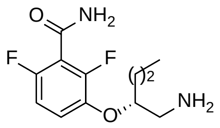

(*R*)-35. Obtained from (*R*)-**32** (120 mg, 0.33 mmol) in 81% yield (69 mg).

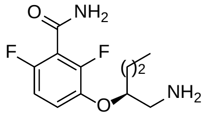

(*S*)-35. Obtained from (*S*)-**32** (128 mg, 0.36 mmol) in 83% yield (77 mg).

3-[(1-Aminoheptan-2-yl)oxy]-2,6-difluorobenzamide (36). Obtained from **33** (24 mg, 0.06 mmol) in 85% yield (27 mg). ESI-MS 289.2 $[M+H]^+$.

3-[(1-(Aminodecan-2-yl)oxy]-2,6-difluorobenzamide (37). Obtained from **34** (110 mg, 0.26 mmol) in 80% yield (68 mg). ESI-MS 329.2 $[M+H]^+$.

3-[(2-Aminopentyl)oxy]-2,6-difluorobenzamide (40). Obtained from **39** (110 mg, 0.26 mmol) in 90% yield (60 mg). ESI-MS 258.1 $[M+H]^+$.

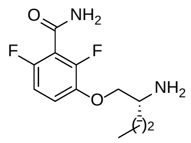

(*R*)-40. Obtained from (*R*)-**39** (266 mg, 0.74 mmol) in 70% yield (135 mg).

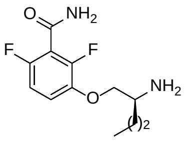

(*S*)-40. Obtained from (*S*)-**39** (225 mg, 0.63 mmol) in 75% yield (121 mg).

- **Synthesis and Characterization Data of Compounds 6-14**

General Procedure for the Synthesis of 6, 9. A mixture of 3-chlorobenzaldehyde (1 equiv) and the corresponding aminobenzamide (1.2 equiv) was stirred at 100 °C under MW irradiation for 10 min. The crude was purified by chromatography with neutralized silica (hexane/EtOAc, 2:1 to 1:3) to afford the pure imine intermediate. Next, the imine was dissolved in methanol (25 mL/mmol) and the solution was pumped through an H-Cube® hydrogenation reactor at a flow-rate of 1 mL/min at 40 °C under full-H_2 mode, using a 10% Pd/C CatCart® cartridge. Solvent was removed under reduced pressure and the residue was purified by chromatography (hexane to hexane/EtOAc, 1:2) to afford the corresponding amine **6**, **9**.

3-[(3-Chlorobenzyl)amino]benzamide (6). Obtained from 3-aminobenzamide (270 mg, 2.0 mmol) and 3-chlorobenzaldehyde (0.19 mL, 1.7 mmol) in 32% yield (137 mg).

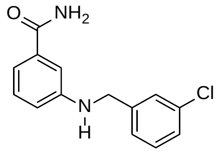

R_f (EtOAc) 0.24; IR (ATR) ν 3346, 3199 (NH), 1655 (CO), 1604, 1582, 1513, 1489 (Ar); ^{1}H NMR (300 MHz, acetone-d_6) δ 4.43 (s, 2H, CH_2), 5.80 (br s, 1H, NH), 6.45 (br s, 1H, ½NH_2) 6.77-6.80 (m, 1H, H_4), 7.13-7.15 (m, 2H, H_5, H_6), 7.24-7.27 (m, 2H, H_2, $H_{6'}$), 7.31-7.36 (m, 2H, $H_{4'}$, $H_{5'}$) 7.44 (s, 1H, $H_{2'}$); ^{13}C NMR (75 MHz, acetone-d_6) δ 47.3 (CH_2), 112.6 (C_2), 116.3 (C_4), 116.5 (C_6), 126.6 ($C_{4'}$), 127.7 ($C_{6'}$), 127.9 ($C_{2'}$), 129.7 (C_5), 130.9 ($C_{5'}$), 134.6 ($C_{3'}$), 136.4 (C_1), 143.8 ($C_{1'}$), 149.5 (C_3), 169.4 ($CONH_2$); ESI-HRMS (calcd., found for $C_{14}H_{14}ClN_2O$ [M(^{35}Cl)+H]$^+$): 261.0744, 261.0741; (calcd., found for $C_{14}H_{14}ClN_2O$ [M(^{37}Cl)+H]$^+$): 263.0771; 263.0775.

4-[(3-Chlorobenzyl)amino]benzamide (9). Obtained from 4-aminobenzamide (209 mg, 1.5 mmol) and 3-chlorobenzaldehyde (0.14 mL, 1.3 mmol) in 74% yield (244 mg).

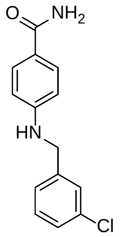

R_f (EtOAc) 0.23; IR (ATR) ν 3338, 3203 (NH), 1647 (CO), 1603, 1569, 1528, 1489 (Ar); ^{1}H NMR (300 MHz, DMSO-d_6) δ 4.34 (s, 2H, CH_2), 6.55 (d, J = 9.0, 2H, H_3, H_5), 6.83 (d, J = 8.5, 1H, ½NH_2), 6.91 (br s, 1H, NH), 7.26-7.35 (m, 3H, $H_{4'}$ - $H_{6'}$), 7.39 (s, 1H, $H_{2'}$), 7.61 (d, J = 9.0, 2H, H_2, H_6), 7.71 (d, J = 8.5, 1H, ½NH_2); ^{13}C NMR (75 MHz, DMSO-d_6) δ 45.3 (CH_2),

111.1 (C_3, C_5), 121.5 (C_1), 125.8 ($C_{4'}$), 126.7 ($C_{6'}$), 126.9 ($C_{2'}$), 129.0 (C_2, C_6), 130.2 ($C_{5'}$), 133.1 ($C_{3'}$), 142.6 ($C_{1'}$), 150.8 (C_4), 167.9 ($CONH_2$); ESI-HRMS (calcd., found for $C_{14}H_{14}ClN_2O$ $[M(^{35}Cl)+H]^+$): 261.0744, 261.0741; (calcd., found for $C_{14}H_{14}ClN_2O$ $[M(^{37}Cl)+H]^+$): 263.0771; 263.0775.

General procedure for the synthesis of 7,10. To a solution of the corresponding secondary amine **6, 9** (1 equiv) in anhydrous DMF (11 mL/mmol), methyl iodide (3 equiv) and potassium carbonate (4 equiv) were added, and the reaction was stirred at 60 °C for 24 h. After this time, the mixture was diluted with EtOAc and washed with brine (3 x 25 mL). The organic phase was dried (Na_2SO_4), filtered, and concentrated under reduced pressure. The residue was purified by chromatography (hexane to hexane/EtOAc, 1:1) to afford the desired compound.

3-[(3-Chlorobenzyl)(methyl)amino]benzamide (7). Obtained from **6** (80 mg, 0.31 mmol) as a white solid in 35% yield (29 mg).

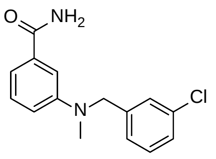

R_f (EtOAc) 0.24; mp: 126-127 °C; IR (ATR) ν 3380, 3188 (NH), 1645 (CO), 1599, 1574 (Ar); ^1H NMR (300 MHz, acetone-d_6) δ 3.10 (s, 3H, CH_3), 4.66 (s, 2H, CH_2), 6.52 (br s, 1H, ½NH_2), 6.87-6.92 (m, 1H, H_4), 7.20-7.34 (m, 6H, H_2, H_5, H_6, $H_{3'}$-$H_{5'}$), 7.37 (s, 1H, $H_{2'}$); ^{13}C NMR (75 MHz, acetone-d_6) δ 39.0 (CH_3), 56.3 (CH_2), 112.3 (C_2), 115.9 (C_4), 116.4 (C_6), 126.1 ($C_{4'}$), 127.5 ($C_{6'}$), 127.7 (C_5), 129.8 ($C_{2'}$), 131.1 ($C_{5'}$), 134.8 ($C_{3'}$), 136.3 (C_1), 142.8 ($C_{1'}$), 150.3 (C_3), 169.5 ($CONH_2$); ESI-HRMS (calcd., found for $C_{15}H_{16}ClN_2O$ $[M(^{35}Cl)+H]^+$): 275.0946, 275.0946; (calcd., found for $C_{15}H_{16}ClN_2O$ $[M(^{37}Cl)+H]^+$): 277.0916; 277.0917

4-[(3-Chlorobenzyl)(methyl)amino]benzamide (10). Obtained from **9** (73 mg, 0.28 mmol) as a yellow solid in 31% yield (24 mg).

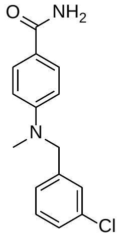

R_f (hexane/EtOAc, 1:2) 0.40; mp: 104-106 °C; IR (ATR) ν 3345 (NH), 1647 (CO), 1602, 1527, 1477 (Ar); ^1H NMR (300 MHz, DMSO-d_6) δ 3.08 (s, 3H, CH_3), 4.66 (s, 2H, CH_2), 6.69 (d, J = 9.0, 2H, H_3, H_5), 6.93 (br s, 1H, ½NH_2), 7.14 (d, J = 7.5, 1H, $H_{6'}$), 7.22 (s, 1H, $H_{2'}$), 7.27-7.38 (m, 2H, $H_{4'}$, $H_{5'}$), 7.61 (br s, 1H, ½NH_2), 7.70 (d, J = 9.0, 2H, H_2, H_6); ^{13}C NMR (75 MHz, DMSO-d_6) δ 38.7 (CH_3), 54.4 (CH_2), 110.7 (C_3, C_5), 121.3 (C_1), 125.3 ($C_{6'}$), 126.4 ($C_{2'}$), 126.8 ($C_{4'}$), 129.0 (C_2, C_6), 130.4 ($C_{5'}$), 133.2 ($C_{3'}$), 141.4 ($C_{1'}$), 150.8 (C_4),

167.8 ($CONH_2$); ESI-HRMS (calcd., found for $C_{15}H_{16}ClN_2O$ $[M(^{35}Cl)+H]^+$): 275.0946, 275.0944; (calcd., found for $C_{15}H_{16}ClN_2O$ $[M(^{37}Cl)+H]^+$): 277.0916; 277.0916.

3-[(3-Chlorobenzyl)(propyl)amino]benzamide (8). To a solution of amine **6** (30 mg, 0.12 mmol) in methanol (3 mL), propionaldehyde (20 mg, 0.35 mmol) was added and the mixture was stirred at rt for 18 h. Then, sodium cyanoborohydride (22 mg, 0.35 mmol) was added and the reaction was stirred at rt for 72 h. The solvent was evaporated under reduced pressure and the residue was purified by chromatography (hexane to hexane/EtOAc, 1:2) to afford compound **8** in 52% yield (18 mg).

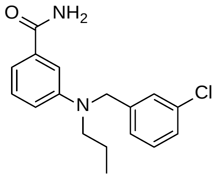

R_f (EtOAc) 0.62; IR (ATR) ν 3404, 3199 (NH), 1650 (CO), 1615, 1598, 1573 (Ar); ^{1}H NMR (300 MHz, acetone-d_6) δ 0.95 (t, J = 7.5, 3H, CH_3), 1.70 (sext, J = 7.5, 2H, CH_2), 3.48 (t, J = 7.5, 2H, CH_2N), 4.67 (s, 2H, CH_2Ar), 6.55 (br s, 1H, ½NH_2), 6.82 (dt, J = 7.1, 2.6, 1H, H_4), 7.15-7.27 (m, 5H, H_5, H_6, $H_{2'}$, $H_{4'}$, $H_{6'}$), 7.33-7.36 (m, 2H, H_2, $H_{5'}$); ^{13}C NMR (75 MHz, acetone-d_6,) δ 11.5 (CH_3), 21.0 (CH_2), 53.8 (CH_2N), 54.4 (CH_2Ar), 112.1 (C_2), 115.7 (C_4), 115.9 (C_6), 126.0 ($C_{4'}$), 127.3 ($C_{6'}$), 127.6 ($C_{2'}$), 129.8 (C_5), 131.0 ($C_{5'}$), 134.8 ($C_{3'}$), 136.3 (C_1), 143.0 ($C_{1'}$), 149.2 (C_3), 169.5 ($CONH_2$); ESI-HRMS (calcd., found for $C_{17}H_{19}ClN_2NaO$ $[M(^{35}Cl)+Na]^+$): 325.1078, 325.1078; (calcd., found for $C_{17}H_{19}ClN_2NaO$ $[M(^{37}Cl)+Na]^+$): 327.1050, 327.1049.

General Procedure for the Synthesis of 11, 13, and 14. To a suspension of 3- or 4-hydroxybenzamide (1-1.5 equiv), sodium iodide (0.2 equiv) and potassium carbonate (3 equiv) in anhydrous DMF (6 mL/mmol), the corresponding bromo derivative (1-1.5 equiv) was added. The reaction was stirred at rt for 24 h. Then, the mixture was diluted in EtOAc and washed with brine (3 x). The organic phase was dried (Na_2SO_4), filtered, and concentrated under reduced pressure. The residue was purified by chromatography (hexane to hexane/EtOAc, 2:8) to yield the desired compounds.

3-[(3-Chlorobenzyl)oxy]benzamide (11).[10] Obtained from 3-hydroxybenzamide (225 mg, 1.6 mmol) and 3-chlorobenzylbromide (0.32 mL, 2.5 mmol) as a white solid in 47% yield (203 mg).

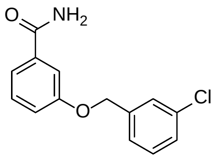

R_f (hexane/EtOAc, 2:8) 0.48; mp: 113-115 ºC (lit.[10] mp 106-108 °C); IR (ATR) ν 3333, 3156 (NH), 1662 (CO), 1630, 1581, 1477, 1446 (Ar), 1251 (CO); ^{1}H NMR (300 MHz, acetone-d_6) δ 5.20 (s, 2H, CH_2), 6.73 (br s, 1H, ½NH_2), 7.19 (ddd, J = 8.2, 2.6, 1.0, 1H, H_4), 7.35-7.48 (m, 5H, $H_{2'}$, $H_{4'}$-$H_{6'}$, ½NH_2), 7.53-7.56 (m, 2H, H_5, H_6), 7.61 (dd, J = 2.5, 1.6, 1H, H_2); ^{13}C NMR (75 MHz, acetone-d_6,) δ 69.6 (CH_2), 114.6 (C_2), 118.8 (C_4), 120.9 (C_6), 126.7 ($C_{2'}$), 128.2 (C_5), 128.6 ($C_{5'}$), 130.3 ($C_{6'}$), 131.0 ($C_{4'}$), 134.7 ($C_{3'}$), 136.9 (C_1), 140.7 ($C_{1'}$), 159.5 (C_3), 168.6 ($CONH_2$); ESI-HRMS (cald., found for $C_{14}H_{12}ClNNaO_2$ [M(^{35}Cl)+Na]$^+$): 284.0449, 284.0448; (cald., found for $C_{14}H_{12}ClNNaO_2$ [M(^{37}Cl)+Na]$^+$): 286.0419, 286.0421.

3-[3-(3-Chlorophenyl)propoxy]benzamide (13). Obtained from 3-hydroxybenzamide (129 mg, 0.94 mmol) and 1-(3-bromopropyl)-3-chlorobenzene (147 mg, 0.63 mmol) as a white solid in 54% yield (98 mg).

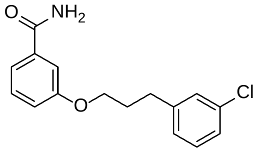

R_f (hexane/EtOAc, 3:7) 0.36; mp: 108-111 ºC; IR (ATR) ν 3363, 3185 (NH), 1655 (CO), 1587, 1449 (Ar); ^{1}H NMR (300 MHz, acetone-d_6) δ 2.09-2.16 (m, 2H, CH_2), 2.84 (t, J = 7.5, 2H, CH_2Ar), 4.06 (t, J = 6.0, 2H, CH_2O), 6.69 (br s, 1H, ½NH_2), 7.09 (ddd, J = 8.2, 2.5, 1.1, 1H, H_4), 7.20-7.23 (m, 2H, $H_{2'}$, $H_{4'}$), 7.28-7.33 (m, 2H, $H_{5'}$, $H_{6'}$), 7.35 (t, J = 8.0, 1H, H_5), 7.46 (br s, 1H, ½NH_2), 7.50-7.52 (m, 2H, H_2, H_6); ^{13}C NMR (75 MHz, acetone-d_6,) δ 31.4 (CH_2), 32.4 (CH_2Ar), 67.7 (CH_2O), 114.2 (C_2), 118.5 (C_4), 120.5 (C_6), 126.8 ($C_{4'}$), 127.9 ($C_{6'}$), 129.3 ($C_{2'}$), 130.2 (C_5), 130.9 ($C_{5'}$), 134.5 ($C_{3'}$), 136.8 (C_1), 145.2 ($C_{1'}$), 160.0 (C_3), 168.7 ($CONH_2$); ESI-HRMS (cald., found for $C_{16}H_{16}ClNNaO_2$ [M(^{35}Cl)+Na]$^+$): 312.0762, 312.0769; (cald., found for $C_{16}H_{16}ClNNaO_2$ [M(^{37}Cl)+Na]$^+$): 314.0732, 314.0742.

4-[(3-Chlorobenzyl)oxy]benzamide (14). Obtained from 4-hydroxybenzamide (150 mg, 1.1 mmol) and 3-chlorobenzylbromide (0.12 mL, 1.1 mmol) as a white solid (mp: 113-115 °C), in 80% yield (230 mg).

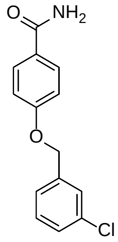

R_f (hexane/EtOAc, 2:8) 0.29; mp: 171-173 °C; ^{1}H NMR (300 MHz, methanol-d_4) δ 5.16 (s, 2H, CH_2), 7.06 (d, J = 9.0, 2H, H_3, H_5), 7.28-7.40 (m, 3H, $H_{3'}$-$H_{6'}$), 7.47 (br s, 1H, $H_{2'}$), 7.85 (d, J = 9.0, 2H, H_2, H_6); ^{13}C NMR (75 MHz, methanol-d_4) δ 70.2 (CH_2), 115.6 (C_3, C_5), 126.8 (CH_{Ar}), 127.5 (C_1), 128.4 ($C_{2'}$), 129.0 (CH_{Ar}), 130.7 (C_2, C_6), 131.2 (CH_{Ar}), 135.5

($C_{3'}$), 140.7 ($C_{1'}$), 162.9 (C_4), 172.0 ($CONH_2$); ESI-MS 262.1 $(M+H)^+$. MALDI-HRMS (calcd., found for $C_{14}H_{12}ClNO_2$ $[M]^+$): 261.0557, 261.0553.

3-[2-(3-Chlorophenyl)ethoxy]benzamide (12). To a solution of 3-hydroxybenzamide (122 mg, 0.89 mmol) in anhydrous THF (5 mL), 2-(3-chlorophenyl)ethanol (0.12 mL, 0.89 mmol), triphenylphosphine (350 mg, 1.3 mmol), trimethylamine (0.25 mL, 1.8 mmol) and diethyl azodicarboxylate (0.21 mL, 1.3 mmol) were added. The reaction was stirred at rt overnight. Then, the mixture was diluted with EtOAc (20 mL) and washed with brine (2x20 mL). The organic phase was dried (Na_2SO_4), filtered, and concentrated under reduced pressure. The residue was purified by chromatography (from hexane to hexane/EtOAc, 2:8), to afford **12** as a white solid in 46% yield (111 mg).

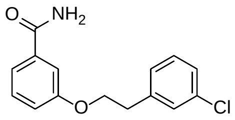

R_f (hexane/EtOAc, 3:7) 0.36; mp: 108-111 ºC; IR (ATR) ν 3363, 3185 (NH), 1655 (CO), 1587, 1449 (Ar); ^{1}H NMR (700 MHz, acetone-d_6) δ 3.13 (t, J = 6.5, 2H, CH_2Ar), 4.29 (t, J = 6.5, 2H, CH_2O), 6.61 (br s, 1H, ½NH_2), 7.09 (ddd, J = 8.3, 2.5, 0.9, 1H, H_4), 7.26-7.27 (m, 1H, $H_{4'}$), 7.33-7.36 (m, 3H, H_5, $H_{5'}$, $H_{6'}$), 7.42 (s, 1H, $H_{2'}$), 7.44 (br s, 1H, ½NH_2), 7.49-7.51 (m, 2H, H_2, H_6); ^{13}C NMR (175 MHz, acetone-d_6) δ 35.8 (CH_2Ar), 69.0 (CH_2O), 114.1 (C_2), 118.5 (C_4), 120.7 (C_6), 127.3 ($C_{4'}$), 128.5 ($C_{6'}$), 129.9 ($C_{2'}$), 130.2 (C_5), 130.8 ($C_{5'}$), 134.5 ($C_{3'}$), 136.8 (C_1), 142.1 ($C_{1'}$), 159.7 (C_3), 168.6 ($CONH_2$); ESI-HRMS (calcd., found for $C_{15}H_{14}ClNNaO_2$ $[M(^{35}Cl)+Na]^+$): 298.0605, 298.0614; (calcd., found for $C_{15}H_{14}ClNNaO_2$ $[M(^{37}Cl)+Na]^+$): 300.0576, 300.0586.

- **Synthesis and Characterization of Compounds 15-17**

1-{3-[(3-Chlorobenzyl)oxy]phenyl}-2,2,2-trifluoroethanol (41). To a solution of 3-[(3-chlorobenzyl)oxy]benzaldehyde (1.01 g, 4.1 mmol) in anhydrous DMF (10 mL), trimethyl(trifluoromethyl)silane (0.73 mL, 4.9 mmol) and potassium carbonate (6 mg, 0.04 mmol) were added and the reaction was stirred at rt for 24 h. After this time, one more equivalent of trimethyl(trifluoromethyl)silane was added (0.6 mL, 4.1 mmol) and the reaction was stirred at rt for additional 24 h. Then, 2 M HCl (2.0 mL, 4.1 mmol) was added and the mixture was stirred at rt for 4 h. The reaction was diluted with EtOAc (20 mL) and washed with brine (3 x 20 mL). The organic phase was dried (Na_2SO_4),

filtered, and concentrated under reduced pressure. The residue was purified by chromatography (from hexane to hexane/EtOAc, 9:1) to afford intermediate **41** in 87% yield (1.13 g).

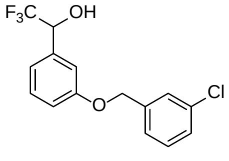

R_f (hexane/EtOAc, 5:1) 0.30; IR (ATR) ν 3452 (OH), 1596, 1488, 1453 (Ar); ^{1}H NMR (300 MHz, acetone-d_6) δ 2.62 (d, J = 4.5, 1H, OH), 4.96-5.02 (m, 1H, CHO), 5.05 (s, 2H, CH$_2$), 7.00 (ddd, J = 8.3, 2.6, 0.8, 1H, H$_4$), 7.08 (d, J = 7.6, 1H, H$_6$), 7.12 (br s, 1H, H$_2$), 7.30-7.36 (m, 4H, H$_5$, H$_{4'}$- H$_{6'}$), 7.45 (s, 1H, H$_{2'}$); ^{13}C NMR (75 MHz, CDCl$_3$) δ 69.3 (CH$_2$), 72.8 (q, J = 32.0, CHO), 114.1 (C$_2$), 116.0 (C$_4$), 120.4 (C$_6$), 124.3 (q, $J_{C\text{-}F}$ = 282.0, CF$_3$), 125.6 (C$_{4'}$), 127.6 (C$_{2'}$), 128.4, (C$_{6'}$), 129.9, 130.0 (C$_5$, C$_{5'}$), 134.7 (C$_{3'}$), 135.6 (C$_1$), 138.8 (C$_{1'}$), 158.8 (C$_3$).

1-{3-[(3-Chlorobenzyl)oxy]phenyl}-2,2,2-trifluoroethanone (42). To a solution of compound **41** (600 mg, 1.9 mmol) in anhydrous DCM (10 mL), Dess-Martin periodinane (1.20 g, 2.8 mmol) was added and the reaction was stirred at rt for 24 h. The mixture was diluted with DCM (10 mL) and washed with a saturated aqueous solution of sodium bicarbonate/sodium thiosulfate (2 x 20 mL). The organic phase was dried (Na_2SO_4), filtered, and concentrated under reduced pressure to give ketone **42** as a colourless oil in 99% yield (596 mg), which was used in the next step without further purification.

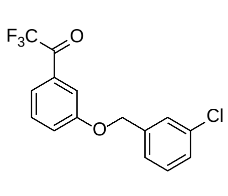

R_f (hexane/EtOAc, 5:1) 0.30; IR (ATR) ν 1717 (CO), 1597, 1581, 1489 (Ar); ^{1}H NMR (700 MHz, acetone-d_6) δ 5.11 (s, 2H, CH$_2$), 7.31-7.35 (m, 4H, H$_4$, H$_{4'}$- H$_{6'}$), 7.46 (s, 1H, H$_{2'}$), 7.48 (t, J = 8.0, 1H, H$_5$), 7.64 (s, 1H, H$_2$), 7.69 (d, J = 7.5, 1H, H$_6$); ^{13}C NMR (175 MHz, CDCl$_3$) δ 69.6 (CH$_2$), 115.2 (C$_2$), 116.7 (q, J = 291.0, CF$_3$), 123.1 (C$_4$), 123.4 (C$_6$), 125.6 (C$_{6'}$), 127.7 (C$_{2'}$), 128.6, 130.1 (C$_{4'}$, C$_{5'}$), 130.4 (C$_5$), 131.3 (C$_1$), 134.8 (C$_{3'}$), 138.2 (C$_{1'}$), 158.9 (C$_3$), 180.4 (q, $J_{C\text{-}F}$ = 35.0, COCF$_3$).

1-{3-[(3-Chlorobenzyl)oxy]phenyl}-2,2,2-trifluoroethanamine (15). To a solution of compound **42** (244 mg, 0.78 mmol) in anhydrous diethyl ether (10 mL), 2-methyl-2-propanesulfinamide (117 mg, 0.97 mmol) and titanium(IV) isopropoxide (0.57 mL, 2.0 mmol) were added and the reaction was refluxed for 24 h. Then, sodium borohydride (88 mg, 2.3 mmol) was added and the reaction was stirred at rt for 24 h. The reducing

agent was quenched with water, the mixture was filtered through celite and washed with EtOAc. The aqueous phase was extracted with EtOAc (3 x 20 mL) and the combined organic phases were dried (Na_2SO_4), filtered, and concentrated under reduced pressure to obtain the corresponding α–trifluoromethyl sulfinamide. Next, the resulting sulfinamide (325 mg, 0.77 mmol) was dissolved in methanol (10 mL) and 4 M hydrochloric acid (0.97 mL, 3.9 mmol) was added. The reaction was stirred at rt for 1 h. Then, a saturated aqueous solution of $NaHCO_3$ was added to neutralize the acid and the mixture was concentrated under reduced pressure. The residue was purified by chromatography (from hexane to hexane/EtOAc, 8.5:1.5) to afford final compound **15** as a solid in 24% yield (60 mg).

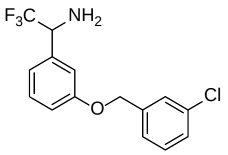

R_f (hexane/EtOAc, 5:1) 0.12; mp: 59-60 ºC; IR (ATR) ν 3407, 3337 (NH), 1600, 1587 (Ar); ^{1}H NMR (700 MHz, $CDCl_3$) δ 4.37 (q, J = 7.4, 1H, CH), 5.05 (s, 2H, CH_2), 6.97 (dd, J = 8.2, 2.0, 1H, H_4), 7.04 (d, J = 7.7, 1H, H_6), 7.07 (s, 1H, H_2), 7.30-7.33 (m, 4H, H_5, $H_{4'}$-$H_{6'}$), 7.45 (s, 1H, $H_{2'}$); ^{13}C NMR (175 MHz, $CDCl_3$) δ 58.0 (q, J = 29.7, CH), 69.3 (CH_2), 114.7 (C_2), 115.2 (C_4), 120.8 (C_6), 125.5 ($C_{6'}$), 125.7 (q, $J_{C\text{-}F}$ = 281.4, CF_3), 127.6 ($C_{2'}$), 128.3, 129.9, 130.0 ($3CH_{Ar}$), 134.7 ($C_{3'}$), 137.2 (C_1), 138.9 ($C_{1'}$), 158.8 (C_3); MALDI-HRMS (calcd., found for $C_{15}H_{13}ClF_3NO$ $[M(^{35}Cl)]^+$): 315.0638, 315.0632; (calcd., found for $C_{15}H_{13}ClF_3NO$ $[M(^{37}Cl)]^+$): 317.0608, 317.0631.

2-(3-Methoxyphenyl)-1*H*-imidazole (43). A mixture of 3-iodoanisole (0.22 mL, 1.8 mmol), imidazole (252 mg, 3.7 mmol), palladium acetate (21 mg, 0.09 mmol), and copper(I) iodide (704 mg, 3.7 mmol) in anhydrous DMF (10 mL) was stirred under MW irradiation at 200 °C for 40 min. Then, the reaction was diluted in EtOAc (30 mL), poured into a saturated aqueous solution of NH_4Cl and stirred for 30 min. Then, the mixture was extracted with EtOAc (3 x 40 mL) and the combined organic phases were washed with brine (2 x 50 mL), dried (Na_2SO_4), filtered, and concentrated under reduced pressure. The residue was purified by chromatography (from DCM to DCM/MeOH, 8:2) to afford intermediate **43** in 53% yield (171 mg). The spectroscopic data were in agreement with those previously reported.[12]

2-(3-Hydroxyphenyl)-1*H*-imidazole (44). To a solution of compound **43** (142 mg, 0.82 mmol) in anhydrous DCM (20 mL) at 0 °C, a 1 M solution of BBr_3 in DCM (1.6 mL, 1.6 mmol) was added and the reaction was stirred at low temperature for 30 min and then at rt for 24 h. Then, the mixture was diluted in EtOAc (20 mL), neutralized

with a saturated aqueous solution of $NaHCO_3$ and the aqueous phase was extracted with EtOAc (3 x 50 mL). The organic phases were dried (Na_2SO_4), filtered, and concentrated under reduced pressure to give **44** as a white solid in 95% yield (124 mg), which was used for the next step without further purification.

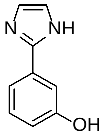

R_f (DCM/MeOH, 9:1) 0.29; ^{1}H-NMR (300 MHz, methanol-d_4): δ 6.82 (ddd, *J* = 8.0, 2.3, 1.6, 1H, H_4), 7.12 (s, 2H, $2H_{imidazole}$), 7.23-7.32 (m, 2H, H_2, H_5, H_6). ^{13}C-NMR (methanol-d_4, 75 MHz): δ 113.4 (C_2), 116.9, 117.6 (C_4, C_6), 123.7 ($2CH_{imidazole}$), 131.0 (C_5), 132.4 (C_1), 148.1 ($C_{imidazole}$), 159.2 (C_3). ESI-MS 161.1 $[M+H]^+$.

2-{3[(3-Chlorobenzyl)oxy]phenyl}-1*H*-imidazole (16). Obtained from compound **44** (50 mg, 0.32 mmol) and 3-chlorobenzyl bromide (28 µL, 0.21 mmol), following the general procedure for the synthesis of **11, 13,** and **14,** as a white solid in 72% yield (43 mg).

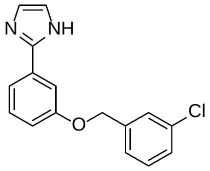

R_f (hexane/EtOAc, 2:8) 0.43; mp: 170-171 ºC; IR (ATR) ν 1742 (C=N), 1591, 1465 (Ar); ^{1}H NMR (500 MHz, methanol-d_4) δ 5.15 (s, 2H, CH_2), 7.02 (ddd, *J* = 8.2, 2.6, 0.9, 1H, H_4), 7.13 (s, 2H, $2CH_{imidazole}$), 7.32 (dt, *J* = 7.7, 1.8, 1H, $H_{4'}$) 7.35-7.41 (m, 3H, $H_{6'}$, H_5, $H_{5'}$), 7.46 (d, *J* = 7.8, 1H, H_6), 7.50 (br s, 1H, $H_{2'}$), 7.55 (br t, *J* = 2.1, 1H, H_2); ^{13}C NMR (125 MHz, methanol-d_4) δ 70.1 (CH_2), 112.8 (C_2), 116.4 (C_4), 119.1 (C_6), 124.0 ($2CH_{imidazole}$), 126.8 ($C_{6'}$), 128.4 ($C_{2'}$), 128.9 ($C_{4'}$), 131.1, 131.2 (C_5, $C_{5'}$), 132.8 (C_1), 135.4 ($C_{3'}$), 141.0 ($C_{1'}$), 147.8 ($C_{imidazole}$), 160.5 (C_3); MS (ESI): 285.0 ($[M(^{35}Cl)+H]^+$, 100%), 287.0 ($[M(^{37}Cl)+H]^+$, 35%); ESI-HRMS (calcd., found for $C_{16}H_{14}ClN_2O$ ($[M(^{35}Cl)+H]^+$): 285.0789, 285.0711; (calcd., found for $C_{16}H_{14}ClN_2O$ ($[M(^{37}Cl)+H]^+$): 287.0760, 287.0681.

2-(3{[3-(Trifluoromethyl)benzyl]oxy}phenyl)-1*H*-imidazole (17). Obtained from compound **44** (50 mg, 0.32 mmol) and 3-(trifluoromethyl)benzyl bromide (32 µL, 0.21 mmol), following the general procedure for the synthesis of **11**, **13**, and **14**, as a solid in 75% yield (50 mg).

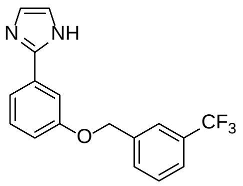

R_f (hexane/EtOAc, 2:8) 0.39; mp: 166-167 ºC; IR (ATR) ν 1741 (C=N), 1588, 1450 (Ar); ^{1}H NMR (500 MHz, methanol-d_4) δ 5.24 (s, 2H, CH_2), 7.05 (dd, *J* = 8.1, 1.8, 1H, H_4), 7.13 (s, 2H, $2CH_{imidazole}$), 7.38 (t, *J* = 7.9, 1H, H_5), 7.47 (d, *J* = 7.8, 1H, H_6),

7.57 (s, 1H, H_2), 7.57-7.65 (m, 2H, $H_{4'}$, $H_{6'}$), 7.75 (br d, *J* = 7.2, 1H, $H_{5'}$), 7.79 (s, 1H, $H_{2'}$); ^{13}C NMR (125 MHz, methanol-d_4) δ 70.1 (CH_2), 112.8 (C_2), 116.4 (C_4), 119.2 (C_6), 125.0 (d, $J_{C\text{-}F}$ = 3.9, $C_{2'}$), 125.6 (d, $J_{C\text{-}F}$ = 3.8, $C_{4'}$), 125.7 (q, $J_{C\text{-}F}$ = 271.3, CF_3), 128.8 ($2CH_{imidazole}$), 130.4 ($C_{6'}$), 131.2 (C_5), 131.9 (q, $J_{C\text{-}F}$ = 32.1, $C_{3'}$), 132.0 ($C_{5'}$), 132.9 (C_1), 140.0 ($C_{1'}$), 147.8 ($C_{imidazole}$), 160.5 (C_3); MS (ESI): 319.1 ($[M+H]^+$, 100%); ESI-HRMS (calcd., found for $C_{17}H_{14}F_3N_2O$ ($[M+H]^+$): 319.1053, 319.0950.

- **Synthesis and Characterization of Compounds 19, 21-24**

General Procedure for the Synthesis of 19 and 21. To a suspension of compound **27** (1 equiv) in anhydrous DMF (6 mL/mmol), potassium carbonate (1.5 equiv), sodium iodide (0.2 equiv), and the corresponding bromo derivative (1.5 equiv) were added. The reaction mixture was stirred at rt for 24 h. Then, the mixture was diluted in EtOAc and washed with brine (3x). The organic phase was dried (Na_2SO_4), filtered, and concentrated under reduced pressure. The residue was purified by chromatography (hexane to hexane/EtOAc, 2:8) to yield the desired compounds.

2,6-Difluoro-3-{[2-(trifluoromethyl)benzyl]oxy}benzamide (19). Obtained from **27** (148 mg, 0.85 mmol) and 2-(trifluoromethyl)benzyl bromide (0.19 mL, 1.3 mmol), as a white solid in 90% yield (252 mg).

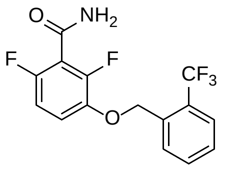

R_f (hexane/EtOAc, 1:1) 0.43; mp: 153-154 °C; IR (ATR) ν 3355 (NH), 1661 (CO), 1595, 1492, 1444 (Ar); ^{1}H NMR (300 MHz, $CDCl_3$) δ 5.31 (s, 2H, CH_2), 6.02 (br s, 1H, NH), 6.06 (br s, 1H, NH), 6.86 (td, *J* = 9.0, 2.0, 1H, H_5), 7.01 (td, *J* = 9.1, 5.1, 1H, H_4), 7.45 (t, *J* = 7.5, 1H, $H_{4'}$), 7.59 (t, *J* = 7.5, 1H, $H_{5'}$), 7.70 (d, *J* = 7.8, 1H, $H_{3'}$), 7.75 (d, *J* = 7.8 1H, $H_{6'}$); ^{13}C NMR (125 MHz, $CDCl_3$) δ 68.4 (CH_2), 111.4 (dd, $J_{C\text{-}F}$ = 23.8, 4.3, C_5), 114.2 (dd, $J_{C\text{-}F}$ = 20.5, 16.2, C_1), 117.9 (dd, $J_{C\text{-}F}$ = 9.8, 3.0, C_4), 124.6 (q, $J_{C\text{-}F}$ = 273.4, CF_3), 126.2 (q, J = 5.6, $C_{3'}$), 127.5 (q, $J_{C\text{-}F}$ = 3.8, $C_{2'}$), 128.3 ($C_{4'}$), 128.8 ($C_{6'}$), 132.5 ($C_{5'}$), 134.7 ($C_{1'}$), 143.4 (dd, *J* = 11.4, 3.5, C_3), 152.5 (dd, $J_{C\text{-}F}$ = 255.1, 7.0, CF), 154.0 (dd, $J_{C\text{-}F}$ = 247.5, 7.0, CF), 162.0 ($CONH_2$); ESI-HRMS (calcd., found for $C_{15}H_{11}F_5NNaO_2$ $[M+Na]^+$): 354.0529, 354.0538.

2,6-Difluoro-3-{[4-(trifluoromethyl)benzyl]oxy}benzamide (21). Obtained from **27** (107 mg, 0.62 mmol) and 4-(trifluoromethyl)benzyl bromide (0.14 mL, 0.92 mmol), as a white solid in 81% yield (252 mg).

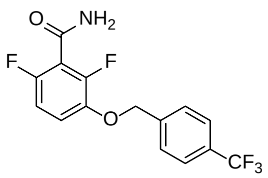

R_f (hexane/EtOAc, 1:1) 0.40; mp: 116-117 °C; IR (ATR) ν 3352 (NH), 1653 (CO), 1492, 1450 (Ar); ^{1}H NMR (300 MHz, $CDCl_3$) δ 5.17 (s, 2H, CH_2), 6.04 (br s, 1H, ½NH_2), 6.29 (br s, 1H, ½NH_2), 6.86 (td, *J* = 9.1, 2.0, 1H, H_5), 7.01 (td, *J* = 9.1, 5.1, 1H, H_4), 7.54 (t, *J* = 7.7, 1H, $H_{2'}$, $H_{6'}$), 7.65 (d, *J* = 7.6, 2H, $H_{3'}$, $H_{5'}$); ^{13}C NMR (75 MHz, $CDCl_3$) δ 71.7 (CH_2), 111.4 (dd, J_{C-F} = 23.8, 4.3, C_5), 114.4 (dd, J_{C-F} = 20.3, 16.3, C_1), 118.0 (dd, J_{C-F} = 9.8, 3.2, C_4), 124.2 (q, J_{C-F} = 270.3, CF_3), 126.2 (q, J_{C-F} = 3.9, $C_{3'}$, $C_{5'}$), 127.5 (q, J_{C-F} = 32.6, $C_{4'}$), 128.3 ($C_{2'}$, $C_{6'}$), 140.2 ($C_{1'}$), 143.4 (dd, *J* = 11.4, 3.5, C_3), 150.9 (dd, J_{C-F} = 266.6, 6.2, CF), 154.1 (dd, J_{C-F} = 260.2, 6.2, CF), 162.0 ($CONH_2$); ESI-HRMS (calcd., found for $C_{15}H_{11}F_5NNaO_2$ $[M+Na]^+$): 354.0529, 354.0523.

General Procedure for the Synthesis of Compounds 45, 46. A mixture of 1-iodo-3-(trifluoromethyl)benzene (1 equiv), tripotassium phosphate (2 equiv), copper (I) iodide (0.04 equiv), the corresponding aminoalcohol (2-aminoethanol or 2-aminopentan-1-ol, 1 equiv), ethylene glycol (2 equiv) in 2-propanol (13 mL/mmol of iodobenzene) was stirred under MW irradiation at 150 °C for 105 min. Then, the reaction was diluted in EtOAc (30 mL), poured into water and the organic layer was extracted with EtOAc (2 x 40 mL). The combined organic phases were washed with brine, dried (Na_2SO_4), filtered, and concentrated under reduced pressure. The residue was purified by chromatography (from hexane to hexane/EtOAc, 1:1) to yield the desired aminoalcohols **45**, **46**.

2-{[3-(Trifluoromethyl)phenyl]amino}ethan-1-ol (45). Obtained from 2-aminoethan-1-ol (0.18 mL, 3 mmol) and 1-iodo-3-(trifluoromethyl)benzene (0.43 mL, 3 mmol) as an off-white solid in 48% yield (296 mg).

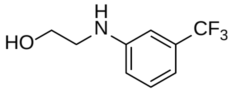

R_f (hexane/EtOAc, 1:1) 0.35; mp: 46-47 °C; IR (ATR) ν 3405 (OH, NH), 1616, 1598, 1454 (Ar); ^{1}H NMR (300 MHz, methanol-d_4) δ 3.25 (t, *J* = 5.8, 2H, CH_2N), 3.72 (t, *J* = 5.8, 2H, CH_2O), 6.84 (app d, *J* = 8.1, 3H, H_2, H_4, H_5), 7.24 (t, *J* = 7.7, 1H, H_6); ^{13}C NMR (75 MHz, methanol-d_4) δ 46.6 (CH_2N), 61.4 (CH_2O), 109.6 (q, J_{C-F} = 4.0, C_2), 113.7 (q, J_{C-F} = 4.0, C_4), 116.8 (C_6), 126.0 (q, J_{C-F} = 271.3, CF_3), 130.7 (C_5), 132.4 (q, J_{C-F} = 31.3, C_3), 150.7 (C_1); ESI-MS 206.1 $[M+H]^+$.

2-{[(3-Trifluoromethyl)phenyl]amino}pentan-1-ol (46). Obtained from 2-aminopentan-1-ol (0.11 mL, 0.97 mmol) and 1-iodo-3-(trifluoromethyl)benzene (0.14 mL, 0.97 mmol) as an oil in 56% yield (134 mg).

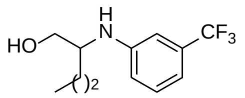

R_f (hexane/EtOAc, 1:1) 0.56; ^{1}H NMR (300 MHz, $CDCl_3$) δ 0.94 (t, *J* = 7.2, 3H, CH_3), 1.34-1.62 (m, 4H, $(CH_2)_2$), 1.80 (br s, 1H, OH), 3.48-3.57 (m, 1H, CHN), 3.56 (dd, *J* = 10.6, 5.4, 1H, ½CH_2O), 3.76 (dd, *J* = 10.5, 3.7, 1H, ½CH_2O), 6.79 (dd, *J* = 8.2, 2.4, 1H, H_6), 6.84 (s, 1H, H_2), 6.94 (d, *J* = 7.7, 1H, H_4), 7.24 (t, *J* = 7.9, 1H, H_5); ^{13}C NMR (75 MHz, $CDCl_3$) δ 14.2 (CH_3), 19.5 (CH_2CH_3), 34.3 (CH_2CH), 55.0 (CHN), 64.4 (CH_2O), 109.8 (q, J_{C-F} = 3.9, C_4), 114.2 (q, J_{C-F} = 4.0, C_2), 116.5 (C_6), 124.4 (q, J_{C-F} = 272.1, CF_3), 129.9 (C_5), 131.8 (q, J_{C-F} = 31.8, C_3), 148.1 (C_1); ESI-HRMS (calcd., found for $C_{11}H_{15}F_3NO$ $[M+H]^+$): 234.1106, 234.1101.

(*R*)-**46**. Obtained from (*R*)-2-aminopentan-1-ol (0.14 mL, 1 mmol) and 1-iodo-3-(trifluoromethyl)benzene (0.17 mL, 1 mmol) as an oil in 46% yield (128 mg).

(*S*)-**46**. Obtained from (*S*)-2-aminopentan-1-ol (0.10 mL, 0.95 mmol) and 1-iodo-3-(trifluoromethyl)benzene (0.12 mL, 0.95 mmol) as an oil in 49% yield (120 mg).

3-(Dimethylamino)-1-[3-(trifluoromethyl)phenyl]propan-1-ol (47). To a suspension of lithium aluminum hydride (30 mg, 0.80 mmol) in anhydrous THF (4 mL), a solution of 3-(dimethylamino)-1-[3-(trifluoromethyl) phenyl]propan-1-one (**48**, 150 mg, 0.61 mmol) in anhydrous THF (8 mL) was added dropwise at 0 ºC and the reaction mixture was stirred at rt for 2 h. Then, the mixture was quenched with a saturated aqueous solution of $NaHCO_3$ at 0 ºC. The mixture was extracted with EtOAc (2 x 25 mL) and the combined organic layers were washed with water and brine, dried (Na_2SO_4), filtered, and concentrated under reduced pressure. The residue was purified by chromatography (from DCM to DCM/MeOH/NH_3, 9:1:0.1) to yield alcohol **47** as a colourless oil in 76% yield (115 mg).

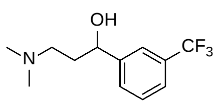

R_f (DCM/MeOH, 9:1): 0.53; ^{1}H NMR (300 MHz, $CDCl_3$) δ 1.71-1.91 (m, 2H, CH_2CH), 2.32 (s, 6H, 2CH_3), 2.48 (ddd, *J* = 12.6, 5.6, 3.4 Hz, 1H, ½CH_2N), 2.70 (ddd, *J* = 12.9, 9.6, 3.5, 1H, ½CH_2N), 3.48 (br s, 1H, OH), 4.99 (dd, *J* = 8.2, 3.4, 1H, CHO), 7.42-7.57 (m, 3H, H_4-H_6), 7.67 (s, 1H, H_2); ^{13}C NMR (75 MHz, $CDCl_3$) δ 34.5 (CH_2CH), 45.4 (2CH_3), 58.5 (CH_2N), 75.4 (CHO), 122.6 (q, *J* = 3.9, C_2),123.9 (q, *J* = 3.8, C_4), 124.5 (q, *J* = 272.8, CF_3), 128.7 (C_6), 129.1 (C_5), 130.7 (d, *J* = 32.7, C_3), 146.3 (C_1). ESI-HRMS (calcd., found for $C_{12}H_{17}F_3NO$ $[M+H]^+$): 248.1262, 248.1258.

General Procedure for the Synthesis of Compounds 22-24. A solution of compound **27**, the proper commercial alcohol or **47** (1 equiv), tributylphosphine (1 equiv), and diisopropyl azodicarboxylate (1 equiv) in anhydrous DMF (8 mL/mmol) was stirred under MW irradiation at 150 °C for 90 min. Then, the reaction was diluted with EtOAc (30 mL) and washed with brine (3 x 20 mL). The organic phase was dried, filtered and concentrated under reduced pressure. The residue was purified by chromatography (from hexane to hexane/EtOAc, 1:1) to yield the desired final compounds **22-26**.

2,6-Difluoro-3-{2-[3-(trifluoromethyl)phenyl]ethoxy}benzamide (22). Obtained from **27** (100 mg, 0.58 mmol) and 2-[3-(trifluoromethyl)phenyl]ethan-1-ol (118 mg, 0.58 mmol), as a white solid in 45% yield (95 mg).

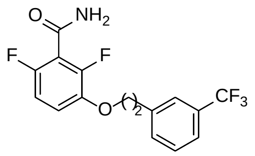

R_f (hexane/EtOAc, 1:1) 0.44; mp: 100-102 °C; IR (ATR) ν 3315, 3196 (NH), 1629 (CO), 1442 (Ar); ^{1}H NMR (300 MHz, $CDCl_3$) δ 3.17 (t, J = 6.6, 2H, CH_2Ar), 4.23 (t, J = 6.6, 2H, CH_2O), 6.00 (br s, 1H, ½NH_2), 6.16 (br s, 1H, ½NH_2), 6.85 (td, J = 9.1, 1.8, 1H, H_5), 6.95 (td, J = 9.0, 5.1, 1H, H_4), 7.41-7.54 (m, 4H, $H_{2'}$, $H_{4'}$-$H_{6'}$); ^{13}C NMR (75 MHz, $CDCl_3$) δ 35.7 (CH$_2$Ar), 70.8 (CH_2O), 111.3 (dd, J_{C-F} = 23.8, 4.3, C_5), 117.5 (dd, J_{C-F} = 9.8, 3.2, C_4), 118.3 (t, J_{C-F} = 20.3, C_1), 123.8 (q, J_{C-F} = 3.8, $C_{2'}$), 124.3 (q, J_{C-F} = 271.8, CF_3), 125.9 (q, J_{C-F} = 3.8, $C_{4'}$), 129.2 ($C_{5'}$), 130.8 (q, J_{C-F} = 32.2, $C_{3'}$), 132.7 ($C_{6'}$), 138.9 ($C_{1'}$), 143.8 (dd, J = 11.4, 3.5, C_3), 150.4 (dd, J_{C-F} = 266.4, 6.3, CF), 154.2 (dd, J_{C-F} = 260.8, 6.3, CF), 162.2 ($CONH_2$); ESI-HRMS (calcd., found for $C_{16}H_{12}F_5NNaO_2$ $[M+Na]^+$): 368.0686, 368.0697.

2,6-Difluoro-3-{3-[3-(trifluoromethyl)phenyl]propoxy}benzamide (23). Obtained from **27** (100 mg, 0.58 mmol) and 2-[3-(trifluoromethyl)phenyl]propan-1-ol (122 mg, 0.58 mmol), as a white solid in 48% yield (80 mg).

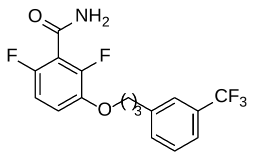

R_f (hexane/EtOAc, 1:1) 0.56; mp: 96-98 °C; IR (ATR) ν 3375, 3191 (NH), 1650 (CO), 1595, 1492, 1450 (Ar); ^{1}H NMR (300 MHz, $CDCl_3$) δ 2.09-2.19 (m, 2H, CH_2), 2.89 (t, J = 8.4, 2H, CH_2Ar), 4.00 (t, J = 6.1 Hz, 2H, CH_2O), 6.01 (br s, 1H, ½NH_2), 6.10 (br s, 1H, ½NH_2), 6.86 (td, J = 9.1, 1.7, 1H, H_5), 6.96 (td, J = 9.0, 5.2, 1H, H_4), 7.39-7.48 (m. 4H, $H_{2'}$, $H_{4'}$-$H_{6'}$); ^{13}C NMR (75 MHz, $CDCl_3$) δ 30.7 (CH_2), 31.9 (CH$_2$Ar), 69.2 (CH_2O), 109.1 (t, J_{C-F} = 20.3, C_1),, 111.3 (dd, J_{C-F} = 23.7, 4.3, C_5), 117.4 (dd, J_{C-F} = 9.7, 3.4, C_4), 123.2 (q, J_{C-F} = 3.9, $C_{2'}$), 124.4 (q, J_{C-F} = 270.3, CF_3), 125.3 (q, J_{C-F} = 3.8, $C_{4'}$), 129.1 ($C_{5'}$),

130.9 (q, $J_{C\text{-}F}$ = 32.1, $C_{3'}$), 132.1 ($C_{6'}$), 142.1 ($C_{1'}$), 144.0 (dd, J = 11.1, 3.4, C_3), 152.1 (d, $J_{C\text{-}F}$ = 254.1, CF), 155.3 (d, $J_{C\text{-}F}$ = 258.2, CF), 162.2 ($CONH_2$); ESI-HRMS (calcd., found for $C_{17}H_{15}F_5NNaO_2$ $[M+Na]^+$): 382.0842, 382.0829.

3-{3-(Dimethylamino)-1-[3-(trifluoromethyl)phenyl]propoxy}-2,6-difluoro-benzamide (24). Obtained from **27** (69 mg, 0.36 mmol) and alcohol **47** (90 mg, 0.36 mmol), as an oil in 50% yield (40 mg). The free base was dissolved in dichloromethane (2 mL) and a solution of 2 M HCl in diethyl ether (2.2 mL) was added dropwise. The mixture was kept at rt for 2 h and the solvent was removed under reduced pressure.

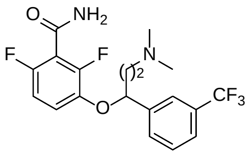

R_f (DCM/MeOH, 9:1, free base) 0.26; ^{1}H NMR (300 MHz, $CDCl_3$) δ 1.90-2.03 (m, 1H, ½CH_2CH), 2.16-2.31 (m, 1H, ½CH_2CH), 2.25 (s, 6H, 2CH_3), 2.36-2.42 (m, 1H, ½CH_2N), 2.48-2.57 (m, 1H, ½CH_2N), 5.28 (dd, J = 8.0, 5.1, 1H, CH), 6.09 (br s, 1H, ½NH_2), 6.23 (br s, 1H, ½NH_2), 6.71 (td, J = 9.1, 1.7, 1H, H_5), 6.82 (td, J = 9.0, 5.3, 1H, H_4), 7.47 (t, J = 7.9, 1H, $H_{5'}$) 7.56 (d, J = 7.9, 2H, $H_{4'}$,$H_{6'}$), 7.63 (s, 1H, $H_{2'}$). ESI-HRMS (calcd., found for $C_{19}H_{20}F_5N_2O_2$ $[M+H]^+$): 403.1444, 403.1438.

- **Synthesis and Characterization of Compounds SP1-SP5**

***tert*-Butyl (2-{[3-(aminocarbonyl)-2,4-difluorophenyl]amino}ethyl)carbamate (50)**. To a solution of **49** (206 mg, 1.2 mmol) and *N*-Boc-2-aminoacetaldehyde (191 mg, 1.2 mmol) in anhydrous methanol (3 mL), acetic acid (69 µL, 1.2 mmol) was added at rt and the mixture was stirred for 2 h. Next, sodium cyanoborohydride (75 mg, 1.2 mmol) was added in one portion, and the reaction was stirred for 24 h at rt. Then, the solvent was evaporated under reduced pressure, and the crude was purified by chromatography (hexane/EtOAc, 1:1 to 1:2) to afford **50** as a colorless oil in 64% yield (241 mg).

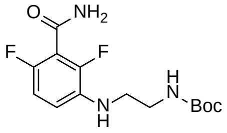

R_f (hexane/EtOAc, 1:3) 0.57; IR (ATR) ν 3352 (NH), 1675 (CO), 1513 (Ar); ^{1}H NMR (700 MHz, methanol-d_4) δ 1.44 (s, 9H, $(CH_3)_3C$), 3.21-3.31 (m, 4H, 2CH_2), 6.77-6.86 (m, 2H, H_4, H_5); ^{13}C NMR (175 MHz, methanol-d_4) δ 28.7 ((*C*H_3)$_3$C), 40.5 (*C*H_2NHCO), 44.6 (ArNHCH_2), 80.2 ((CH_3)$_3$*C*), 111.9 (dd, $J_{C\text{-}F}$ = 22.2, 3.6, C_5), 113.4 (dd, $J_{C\text{-}F}$ = 8.4, 5.4, C_4), 115.4 (dd, $J_{C\text{-}F}$ = 23.0, 19.6, C_1), 135.1 (dd, $J_{C\text{-}F}$ = 11.9, 2.1, C_3), 148.4 (dd, $J_{C\text{-}F}$ = 244.3, 7.1, CF), 151.4 (dd, $J_{C\text{-}F}$ = 238.5, 6.3, CF), 158.8 (NHCO), 166.3 ($CONH_2$); ESI-MS 216.1 $[M\text{-}Boc+H]^+$.

Compound SP1. To a solution of **50** (13 mg, 0.04 mmol) in anhydrous DCM (1 mL), TFA (64 µL, 0.82 mmol) was added dropwise and the reaction mixture was stirred at rt for 2 h. Then, the solvent was removed by azeotropic distillation with toluene (2x) under reduced pressure to afford the trifluoroacetate salt of 3-[(2-aminoethyl)amino]-2,6-difluorobenzamide (**51**) in quantitative yield, which was used in the next step without further purification.

To a solution of **51** (18 mg, 0.04 mmol), and Cs_2CO_3 (65 mg, 0.20 mmol) in dry acetonitrile (3 mL), a solution of Cl-NBD (10 mg, 0.05 mmol) in dry acetonitrile (0.5 mL) was added and the reaction was stirred at 80 ºC for 2 h. Then, the solvent was evaporated under reduced pressure and the crude was purified by chromatography (from hexane to hexane/EtOAc, 1:2) to afford compound **SP1** as an orange oil in 47% yield (7 mg).

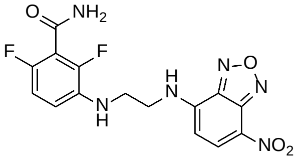

R_f (hexane/EtOAc, 1:3) 0.23; IR (ATR) ν 3355 (NH), 1672 (CO), 1585, 1494, 1446 (Ar); ^{1}H NMR (700 MHz, methanol-d_4) δ 3.57 (t, J = 6.1, 2H, CH_2CH$_2$NHNBD), 3.75 (br s, 2H, CH_2NHNBD), 6.37 (d, J = 8.9, 1H, CH_{NBD}), 6.84-6.90 (m, 2H, H_4, H_5), 8.51 (d, J = 8.8, 1H, CH_{NBD}); ^{13}C NMR (175 MHz, methanol-d_4) δ 42.8 (CH$_2$CH$_2$NHNBD), 43.8 (CH$_2$NHNBD), 99.7 (CH_{NBD}), 112.5 (dd, $J_{C\text{-}F}$ = 22.3, 3.7, C_5), 113.6 (dd, $J_{C\text{-}F}$ = 8.1, 4.6, C_4), 115.6 (dd, $J_{C\text{-}F}$ = 23.0, 19.3, C_1), 123.4 (C_{NBD}), 134.6 (dd, $J_{C\text{-}F}$ = 12.0, 2.6, C_3), 138.1 (CH_{NBD}), 145.5 ($2C_{NBD}$), 146.0 (C_{NBD}), 148.5 (dd, $J_{C\text{-}F}$ = 244.5, 6.6, CF), 151.5 (dd, $J_{C\text{-}F}$ = 239.0, 6.5, CF), 166.1 ($CONH_2$); ESI-HRMS (calcd., found for $C_{15}H_{11}F_2N_6O_4$ [M-H]$^-$): 377.0815, 377.0782.

General Procedure for the Synthesis of SP2-SP4. To a solution of amine **32** or **51** (1.5 equiv) and Et_3N (3 equiv) in anhydrous DCM (50 mL/mmol) at rt, *N*-hydroxysuccinimide (NHS) ester of the corresponding BODIPY derivative (1 equiv) was added and the reaction was stirred for 24 h. Then, the mixture was diluted with DCM and washed with water. The organic phase was dried (Na_2SO_3), filtered and concentrated under reduced pressure. The residue was purified by chromatography (DCM/EtOAc) to yield the desired compound **SP2-SP4**.

Compound SP2. Obtained from **51** (3.2 mg, 0.015 mol) and BODIPY-558/568 NHS ester (4.4 mg, 0.010 mmol), as a purple oil in 24% yield (1.3 mg). Chromatography: DCM/EtOAc, 3:1 to 1:5.

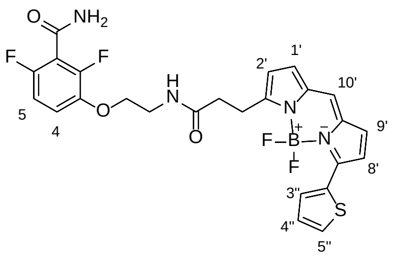

R_f (DCM/EtOAc, 1:1) 0.13; IR (ATR) ν 3405 (NH), 1680 (CO), 1606, 1547, 1484 (Ar); ^{1}H NMR (300 MHz, methanol-d_4) δ 2.68 (t, J = 7.4, 2H, CH_2CO), 3.31-3.33 (m, 2H, CH_2CH_2CO), 3.57 (t, J = 5.3, 2H, CH_2N), 4.09 (t, J = 5.4, 2H, CH_2O), 6.46 (d, J = 4.3, 1H, $H_{2'}$), 6.87 (d, J = 4.4, 1H, $H_{8'}/H_{9'}$), 6.92 (td, J = 9.0, 2.0, 1H, H_5), 7.05 (d, J = 4.1, 1H, $H_{1'}$), 7.10-7.19 (m, 3H, H_4, $H_{4''}$, $H_{8'}/H_{9'}$), 7.41 (s, 1H, $H_{10'}$), 7.64 (dd, J = 5.1, 0.9, 1H, $H_{5''}$), 8.11 (dd, J = 3.8, 0.9, 1H, $H_{3''}$); ESI-HRMS (calcd., found for $C_{25}H_{21}BF_4N_4NaO_3S$ $[M+Na]^+$): 567.1256, 567.1251.

Compound SP3. Obtained from **32** (3.9 mg, 0.015 mmol) and BODIPY-558/568 NHS ester (4.4 mg, 0.010 mmol), as a purple oil in 30% yield (1.8 mg). Chromatography: DCM/EtOAc, 5:1 to 1:1.

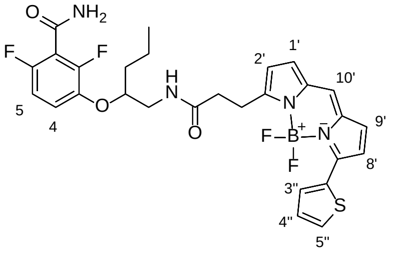

R_f (DCM/EtOAc, 1:1) 0.28; IR (ATR) ν 3395 (NH), 1671 (C=O), 1605, 1480 (Ar); ^{1}H NMR (700 MHz, methanol-d_4) δ 0.92 (t, J = 7.5, 3H, CH_3), 1.39-1.44 (m, 1H, ½CH_2CH_3), 1.46-1.51 (m, 1H, ½CH_2CH_3), 1.58-1.64 (m, 2H, CH_2CH), 2.63-2.68 (m, 2H, CH_2CO), 3.29-3.33 (m, 2H, CH_2CH_2CO), 3.41-3.46 (m, 2H, CH_2N), 4.34-4.37 (m, 1H, CHO), 6.46 (d, J = 4.1, 1H, $H_{2'}$), 6.88 (d, J = 4.3, 1H, $H_{8'}/H_{9'}$), 6.91 (td, J = 9.0, 1.7, 1H, H_5), 7.10 (d, J = 4.2, 1H, $H_{1'}$), 7.18 (dd, J = 5.0, 3.9, 1H, $H_{4''}$), 7.20 (d, J = 4.3, 1H, $H_{8'}/H_{9'}$), 7.24 (td, J = 9.2, 5.2, 1H, H_4), 7.44 (s, 1H, $H_{10'}$), 7.65 (dd, J = 5.0, 1.0, 1H, $H_{5''}$), 8.12 (dd, J = 3.8, 1.0, 1H, $H_{3''}$); ^{13}C NMR (175 MHz, methanol-d_4) δ 14.5 (CH_3), 19.5 (CH_2CH_3), 25.9 (CH_2CH_2CO), 35.6 (CH_2CH), 35.7 (CH_2CO), 43.5 (CH_2N), 80.2 (CHO), 111.9 (dd, $J_{C\text{-}F}$ = 23.0, 4.0, C_5), 116.7 (dd, $J_{C\text{-}F}$ = 24.0, 20.0, C_1), 119.7 ($C_{2'}$), 119.9 (d, $J_{C\text{-}F}$ = 10.0, C_4), 120.7 (CH_{bodipy}), 128.4 ($C_{10'}$), 129.6 ($C_{4''}$), 130.3 ($C_{5''}$), 131.2 ($C_{1'}$), 131.8 (CH_{bodipy}), 132.1 ($C_{3''}$), 135.0 ($C_{2''}$), 136.2 ($C_{10a''}$), 138.0 ($C_{9a''}$), 144.1 (dd, $J_{C\text{-}F}$ = 11.0, 3.0, C_3), 150.9 (dd, $J_{C\text{-}F}$ = 251.5, 8.0, CF), 151.7 ($C_{7'}$), 154.1 (dd, $J_{C\text{-}F}$ = 244.0, 6.0, CF), 161.8 ($C_{3'}$), 165.4 ($CONH_2$), 174.5 (CONH); ESI-HRMS (calcd., found for $C_{28}H_{27}BF_4N_4NaO_3S$ $[M+Na]^+$): 609.1725, 609.1839.

Compound SP4. Obtained from **32** (15 mg, 0.06 mmol) and BODIPY-FL NHS ester (16 mg, 0.04 mmol), as a red oil in 95% yield (20 mg). Chromatography: DCM/EtOAc, 4:1 to 1:1.

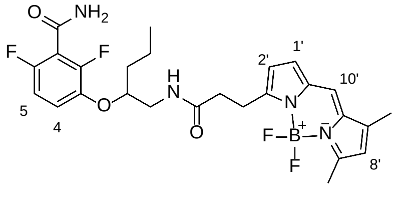

R_f (DCM/EtOAc, 1:1) 0.28; IR (ATR) ν 3351 (NH), 1683 (C=O), 1623, 1507, 1489 (Ar); ^{1}H NMR (700 MHz, $CDCl_3$) δ 0.90 (t, J = 7.3, 3H, CH_2CH_3), 1.35-1.45 (m, 2H, CH_2CH$_3$), 1.48-1.53 (m, 1H, ½CH_2CH), 1.59-1.63 (m, 1H, ½CH_2CH), 2.25 (s, 3H, $C_{9'}CH_3$), 2.53 (s, 3H, $C_{7'}CH_3$), 2.59-2.66 (m, 2H, CH_2CO), 3.22 (t, J = 7.5, 2H, CH_2CH$_2$CO), 3.30-3.34 (m, 1H, ½CH$_2$N), 3.54-3.58 (m, 1H, ½CH$_2$N), 4.21-4.24 (m, 1H, CHO), 5.87 (br s, 1H, ½NH$_2$), 6.09 (br s, 1H, NH), 6.12 (s, 1H, $H_{8'}$), 6.14 (br s, 1H, ½NH$_2$), 6.25 (d, J = 3.9, 1H, $H_{2'}$), 6.81-6.84 (m, 2H, H_5, $H_{1'}$), 7.05-7.08 (m, 2H, H_4, $H_{10'}$); ^{13}C NMR (175 MHz, $CDCl_3$) δ 11.5 ($C_{9'}$$CH_3$), 14.2 (CH$_2$$CH_3$), 15.1 ($C_{7'}$$CH_3$), 18.5 ($CH_2CH_3$), 25.0 ($CH_2CH_2$CO), 34.3 ($CH_2$CH), 36.0 ($CH_2$CO), 42.7 (CH$_2$N), 80.0 (CHO), 111.5 (dd, J = 23.6, 3.9, C_5), 114.2 (dd, J = 20.3, 17.2, C_1), 117.4 ($C_{2'}$), 120.1 (dd, J = 9.7, 2.5, C_4), 120.7 ($C_{8'}$), 124.0 ($C_{10'}$), 128.3 ($C_{1'}$), 133.4 ($C_{10a'}$), 135.3 ($C_{9a'}$), 143.1 (dd, J = 11.1, 3.4, C_3), 144.4 ($C_{9'}$), 150.8 (dd, J = 253.2, 7.0, CF), 153.9 (dd, J = 247.5, 5.4, CF), 157.0 ($C_{3'}$), 160.6 ($C_{7'}$), 162.1 (CONH$_2$), 172.2 (CONH); ESI-HRMS (calcd., found for $C_{26}H_{29}BF_4N_4NaO_3$ [M+Na]$^+$): 555.2161, 555.2257.

Compound SP5. To a solution of acrylodan (1-[6-(dimethylamino)-2-naphthyl]prop-2-en-1-one) (9 mg, 0.04 mmol) in absolute ethanol (2 mL) at rt, a solution of **32** (10 mg, 0.04 mmol) in absolute ethanol (0.5 mL) was added dropwise, and the reaction was stirred at 65 ºC for 24 h. Then, the solvent was evaporated under reduced pressure and the residue was purified by chromatography (EtOAc to EtOAc/MeOH 8:1) to yield **SP5** as a yellow oil in 32% yield (6 mg).

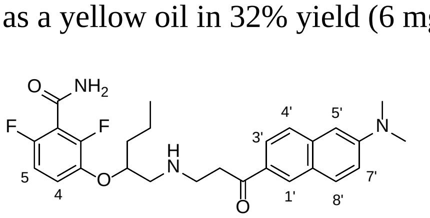

R_f (EtOAc/MeOH, 8:2) 0.41; IR (ATR) ν 3460 (NH), 1655, 1621 (CO), 1598, 1509 (Ar); ^{1}H NMR (700 MHz, methanol-d_4) δ 0.94 (t, J = 7.4, 3H, CH_3), 1.41-1.46 (m, 2H, CH_2CH$_3$), 1.62-1.66 (m, 1H, ½CH_2CH), 1.68-1.73 (m, 1H, ½CH_2CH), 2.98 (dd, J = 12.9, 3.8, 2H, ½CHCH_2N), 3.02 (dd, J = 12.9, 7.4, 2H, ½CHCH_2N), 3.10 (s, 6H, N(CH$_3$)$_2$), 3.13 (J = 6.3, 3.8, 2H, NCH_2CH$_2$CO), 3.37 (td, J = 6.3, 1.2, 2H,

CH_2CO), 4.45-4.48 (m, 1H, CHO), 6.92-6.95 (m, 2H, H_5, $H_{5'}$), 7.23-7.26 (m, 1H, H_4), 7.25 (dd, J = 9.1, 2.6, 1H, $H_{7'}$), 7.65 (d, J = 8.7, 1H, $H_{3'}$), 7.84 (d, J = 9.1, 1H, $H_{8'}$), 7.86 (dd, J = 8.7, 1.8, 1H, $H_{4'}$), 8.40 (d, J = 1.4, 1H, $H_{1'}$); ^{13}C NMR (175 MHz, methanol-d_4) δ 14.4 (CH_3), 19.3 (CH_2CH_3), 35.4 (CH_2CH), 37.9 (CH_2CO), 40.5 ($N(CH_3)_2$), 45.8 (NCH_2CH_2CO), 53.4 ($CHCH_2N$), 81.0 (CHO), 106.3 ($C_{5'}$), 112.2 (dd, J = 23.0, 3.8, C_5), 117.0 (dd, J = 23.6, 19.8, C_1), 117.6 ($C_{7'}$), 121.0 (d, J = 9.5, 2.6, C_4), 124.9 ($C_{4'}$), 126.5 (C_{napht}), 127.4 ($C_{3'}$), 131.1 ($C_{1'}$), 131.6 ($C_{8'}$), 131.8 (C_{napht}), 139.5 (C_{napht}), 143.8 (dd, J = 10.4, 2.7, C_3), 151.5 (d, J = 250.7, 7.9, CF), 152.1 ($C_{6'}$), 154.6 (dd, J = 244.1, 6.4, CF), 165.3 ($CONH_2$), 200.5 (CO); ESI-HRMS (calcd., found for $C_{27}H_{32}F_2N_3O_3$ $[M+H]^+$): 484.2406, 484.2466.

3.2. Solubility of Compounds. Stock solutions of compounds were prepared in DMSO (spectroscopic grade, Merck) and stored at -20 ºC. Compound solubility in 50 mM HEPES-KOH, 50 mM KCl, 1 mM EDTA, pH 6.8 (HEPES buffer) containing 2% DMSO was determined spectrophotometrically, monitoring the linear correlation between the absorbance and the compound concentration and the absence of light scattering. The solutions were optionally centrifuged at 100000 rpm for 20 min at 25 ºC in a TLA100 rotor with a Beckman Optima Max-XP ultracentrifuge. Solubility values for probes **1-5** and **SP1-SP5** are in Figure S1 and the values for compounds **6-26** are in Table S6, only to provide a comparative measurement of compound solubility in buffer with residual DMSO. Notice that in the presence of protein in biochemical experiments, in culture media in microbiological assays, or with 4 - 20 % (v/v) 1-methyl-2-pyrrolidone in crystallographic experiments the compounds could be dissolved at concentrations in practice higher than in Table S6.

Table S6. Solubility in HEPES buffer of compounds 6-26 and PC190723.

Compound	Solubility (μM)
**6**	≥ 100
**7**	≥ 100
**8**	5
**9**	≥ 100
**10**	≥ 100
**11**	100
**12**	≥ 100
**13**	25
**18**	≥ 100
**19**	50
**20**	50
**21**	100
**22**	>100
**23**	50
**24**	>1000 (soluble in buffer)
**25**	100
rac-**26**	50
(*R*)-**26**	100
(*S*)-**26**	100
**PC190723**	10

4. References

1. Artola, M.; Ruiz-Avila, L. B.; Ramirez-Aportela, E.; Martinez, R. F.; Araujo-Bazan, L.; Vazquez-Villa, H.; Martin-Fontecha, M.; Oliva, M. A.; Martin-Galiano, A. J.; Chacon, P.; Lopez-Rodriguez, M. L.; Andreu, J. M.; Huecas, S. The structural assembly switch of cell division protein FtsZ probed with fluorescent allosteric inhibitors. *Chem. Sci.* **2017**, *8*, 1525-1534.

2. Wallace, A. C.; Laskowski. R. A.; Thornton, J. M. LIGPLOT: a program to generate schematic diagrams of protein-ligand interactions. *Protein Eng.* **1996**, *8*, 127-134.

3. Artola, M.; Ruiz-Avila, L. B.; Vergonos, A.; Huecas, S.; Araujo-Bazan, L.; Martin-Fontecha, M.; Vazquez-Villa, H.; Turrado, C.; Ramirez-Aportela, E.; Hoegl, A.; Nodwell, M.; Barasoain, I.; Chacon, P.; Sieber, S. A.; Andreu, J. M.; Lopez-Rodriguez, M. L. Effective GTP-replacing FtsZ inhibitors and antibacterial mechanism of action.. *ACS Chem. Biol.* **2015**, *10*, 834-843.

4. Haydon, D. J.; Bennett, J. M.; Brown, D.; Collins, I.; Galbraith, G.; Lancett, P.; Macdonald, R.; Stokes, N. R.; Chauhan, P. K.; Sutariya, J. K.; Nayal, N.; Srivastava, A.; Beanland, J.; Hall, R.; Henstock, V.; Noula, C.; Rockley, C.; Czaplewski, L. Creating an antibacterial with in vivo efficacy: synthesis and characterization of potent inhibitors of the bacterial cell division protein FtsZ with improved pharmaceutical properties. *J. Med. Chem.* **2010**, *53*, 3927-3936.

5. Sliwinska, A.; Zwierzak, A. Regioselective aminobromination of terminal alkenes. *Tetrahedron* **2003**, *59*, 5927-5934.

6. Garcin, E. D.; Arvai, A. S.; Rosenfeld, R. J.; Kroeger, M. D.; Crane, B. R.; Andersson, G.; Andrews, G.; Hamley, P. J.; Mallinder, P. R.; Nicholls, D. J.; St-Gallay, S. A.; Tinker, A. C.; Gensmantel, N. P.; Mete, A.; Cheshire, D. R.; Connolly, S.; Stuehr, D. J.; Aberg, A.; Wallace, A. V.; Tainer, J. A.; Getzoff, E. D. Anchored plasticity opens doors for selective inhibitor design in nitric oxide synthase. *Nat. Chem. Biol.* **2008**, *4*, 700-707.

7. Lehmann, F.; Pilotti, A.; Luthman, K. Efficient large scale microwave assisted Mannich reactions using substituted acetophenones. *Mol. Divers.* **2003**, *7*, 145-152.

8. Lui, H. K.; Gao, W.; Cheung, K. C.; Jin, W. B.; Sun, N.; Kan, J. W. Y.; Wong, I. L. K.; Chiou, J.; Lin, D.; Chan, E. W. C.; Leung, Y.-C.; Chan, T. H.; Chen, S.; Chan, K.-F.; Wong, K.-Y. Boosting the efficacy of anti-MRSA β-lactam antibiotics via an easily accessible, non-cytotoxic and orally bioavailable FtsZ inhibitor. *Eur. J. Med. Chem.*, **2019**, *163*, 95-115.

9. Enders, D.; Haertwig, A.; Raabe, G.; Runsink, J. Diastereo- and enantioselective synthesis of vicinal amino alcohols by oxa michael addition of *N*-formylnorephedrine to nitro alkenes. *Eur. J. Org. Chem.* **1998**, 1771-1792.

10. Qiang, S.; Wang, C.; Venter, H.; Li, X.; Wang, Y.; Guo, L.; Ma, R.; Ma, S. Synthesis and biological evaluation of novel FtsZ-targeted 3-arylalkoxy-2,6-difluorobenzamides as potential antimicrobial agents. *Chem. Biol. Drug Des.* **2015**, *87*, 257-264.

11. Haneda, S.; Okui, A.; Ueba, C.; Hayashi, M. An efficient synthesis of 2-arylimidazoles by oxidation of 2-arylimidazolines using activated carbon–O_2 system and its application to palladium-catalyzed Mizoroki–Heck reaction. *Tetrahedon* **2007**, *63*, 2414–2417.

5. Spectra of Compounds 2-26 and SP1-SP5.

^{1}H NMR spectrum for **2** ($CDCl_3$, 300 MHz)

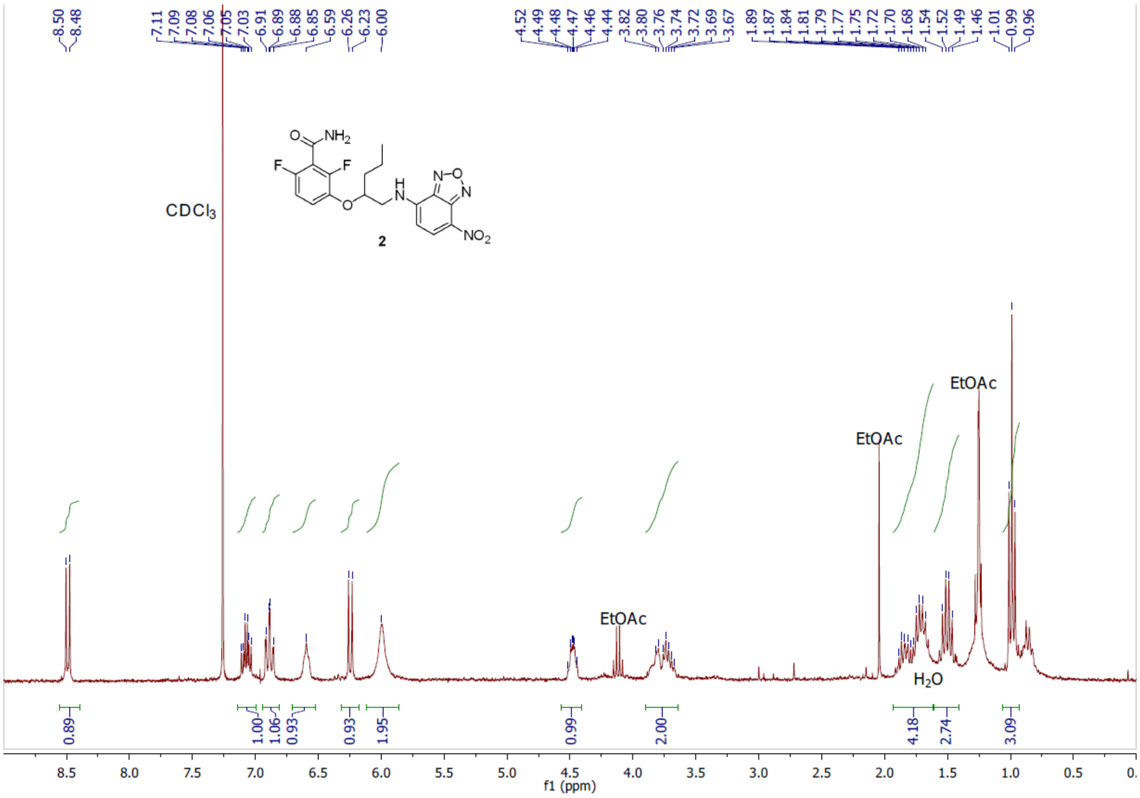

^{13}C NMR spectrum for **2** ($CDCl_3$, 175 MHz)

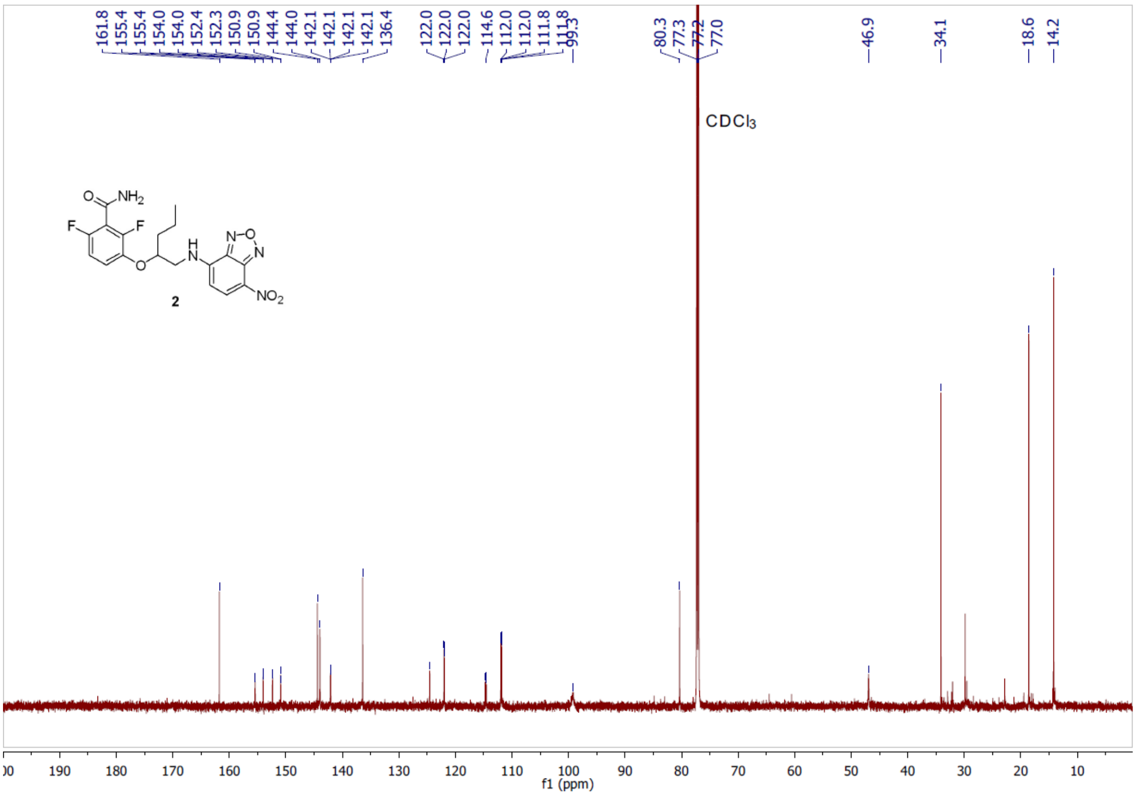

^{1}H NMR spectrum for **3** ($CDCl_3$, 300 MHz)

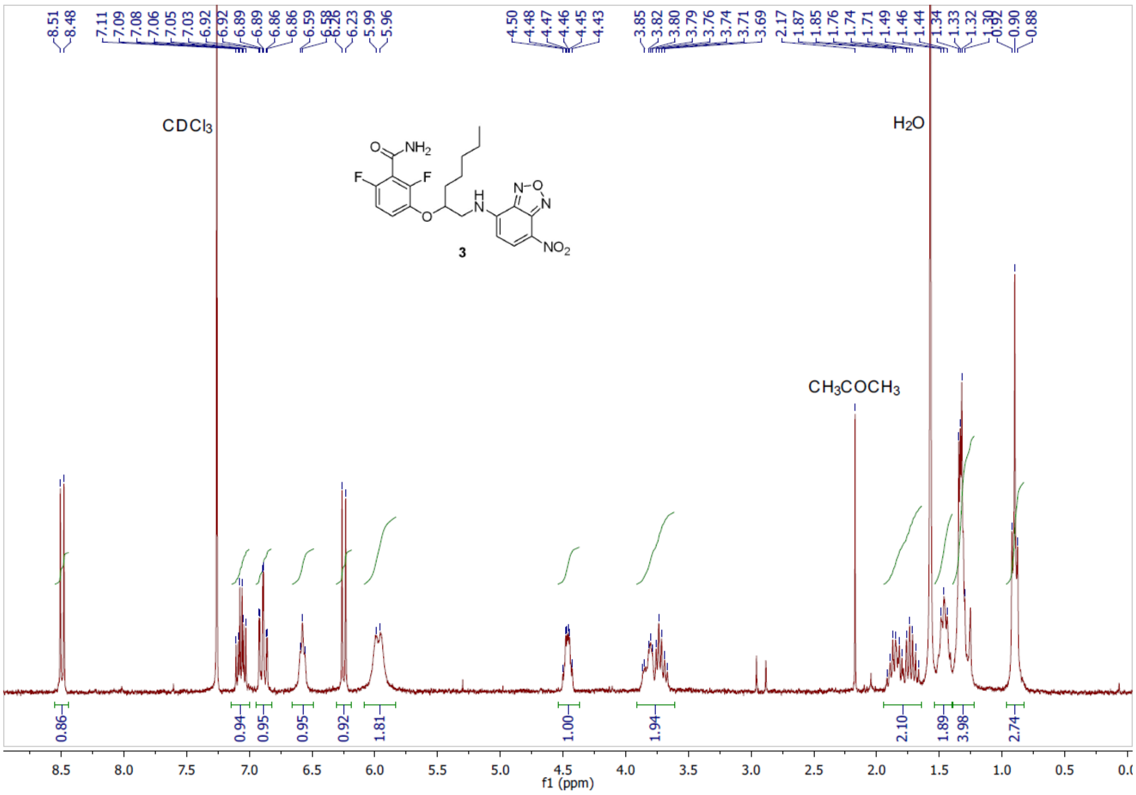

^{13}C NMR spectrum for **3** ($CDCl_3$, 175 MHz)

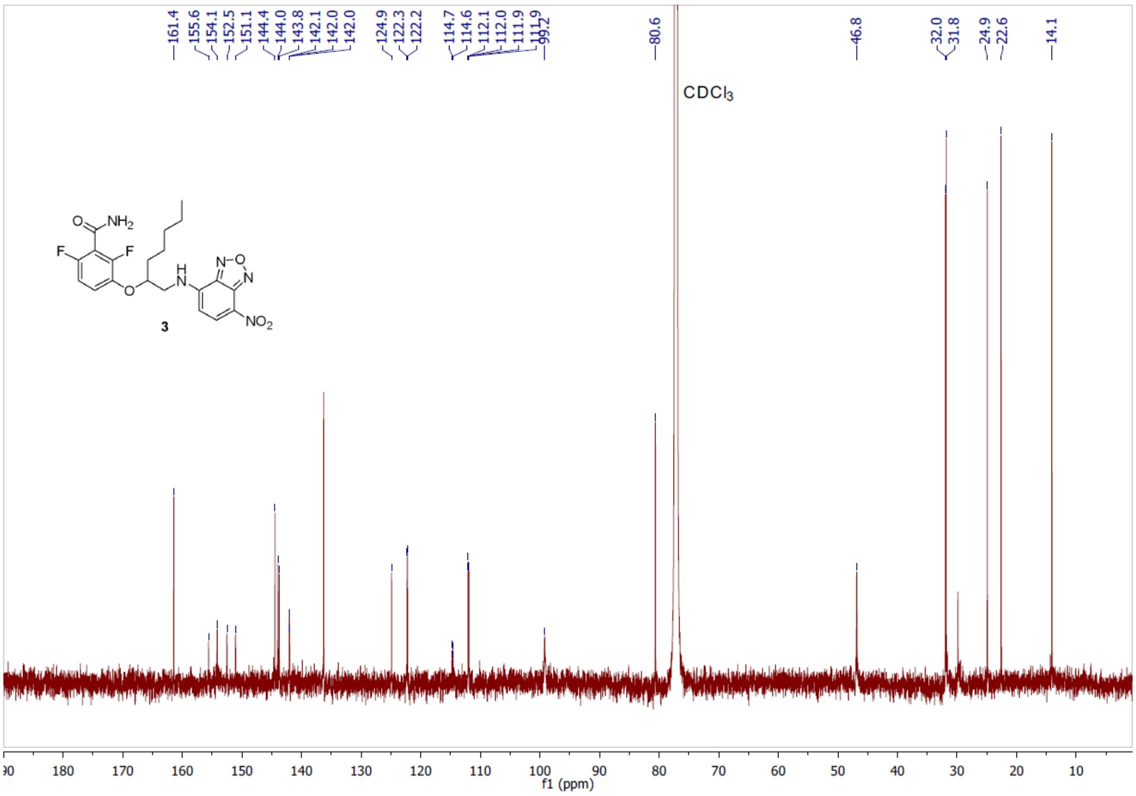

^{1}H NMR spectrum for **4** (($(CD_3)_2CO$, 700 MHz)

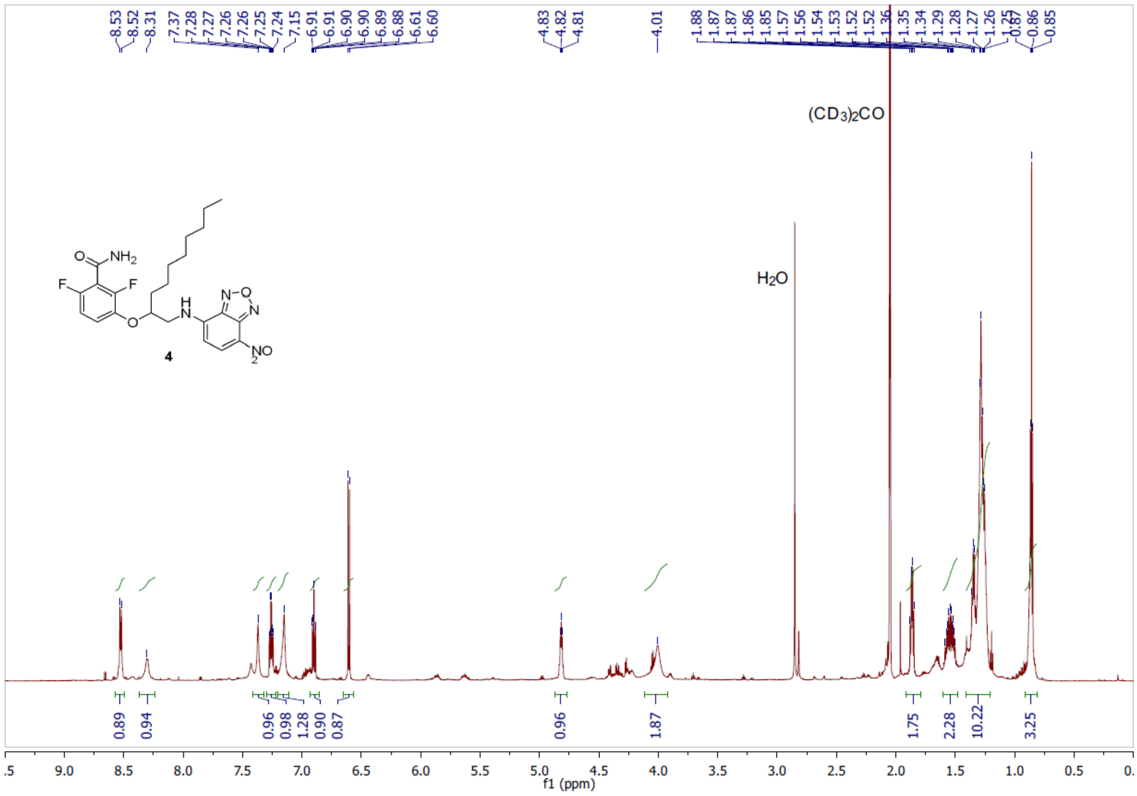

^{13}C NMR spectrum for **4** (($(CD_3)_2CO$, 175 MHz)

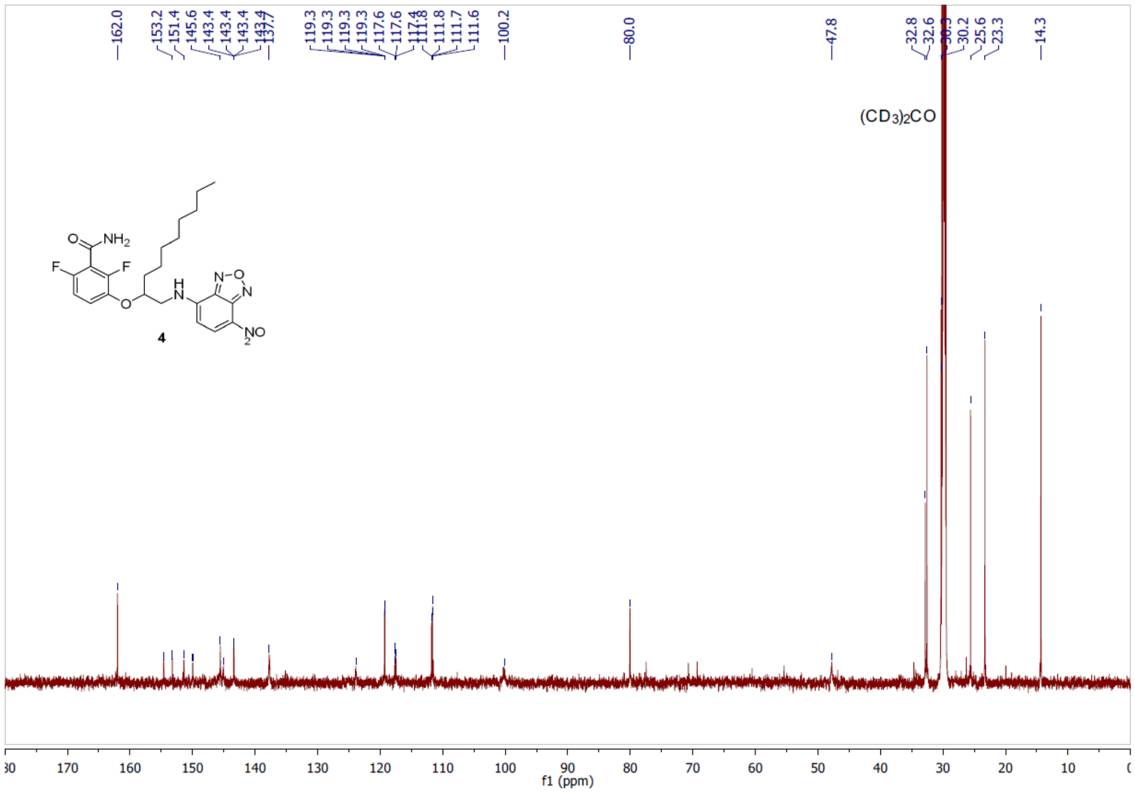

^{1}H NMR spectrum for **5** (CD_3OD, 700 MHz)

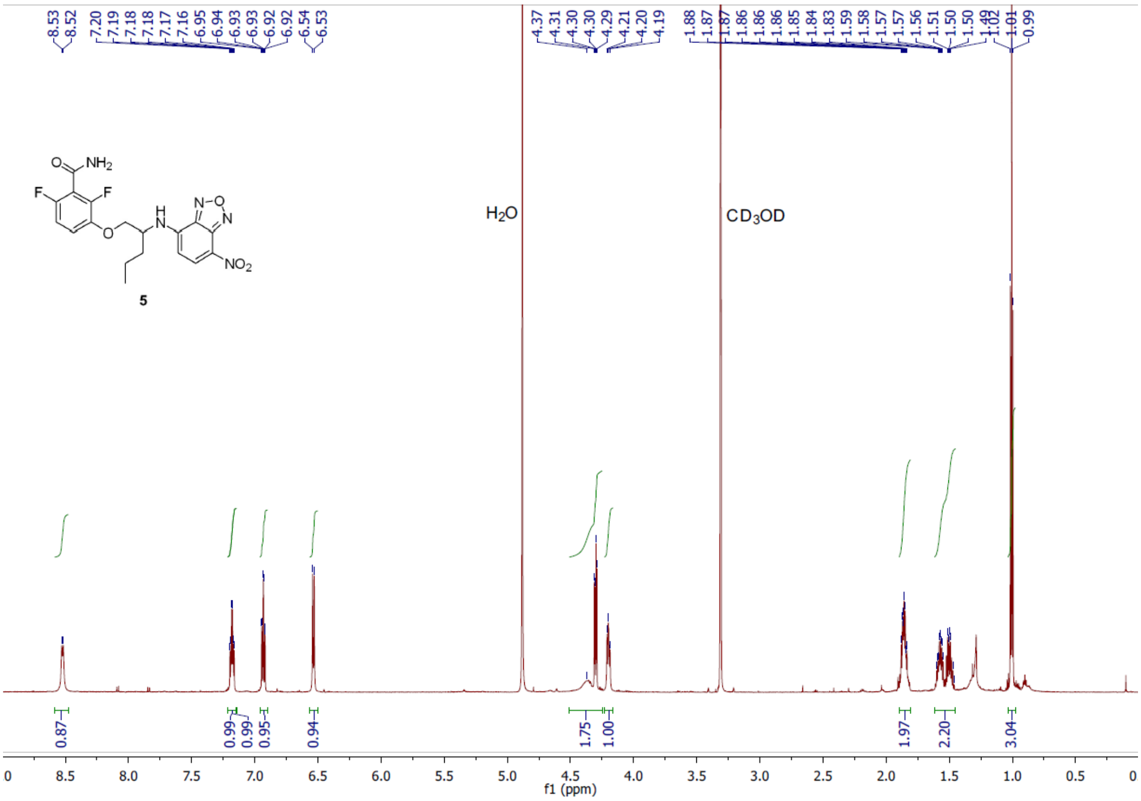

^{13}C NMR spectrum for **5** (CD_3OD, 175 MHz)

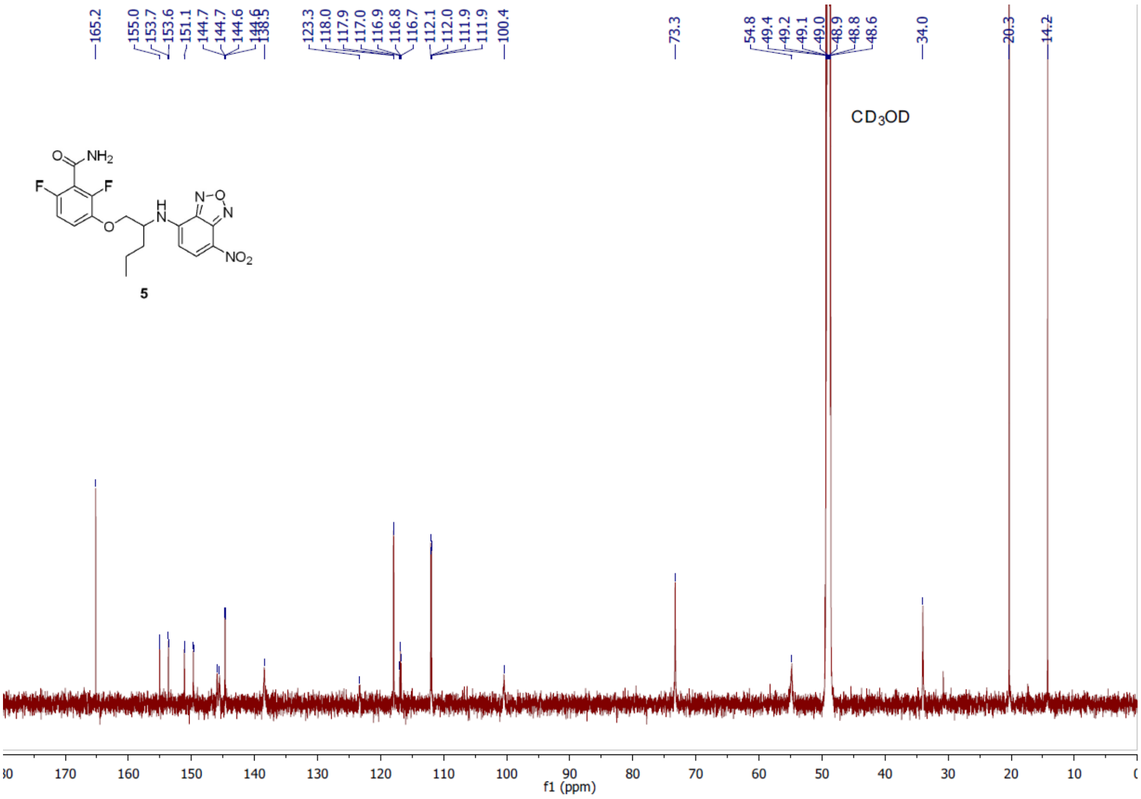

^{1}H NMR spectrum for **6** ($(CD_3)_2CO$, 300 MHz)

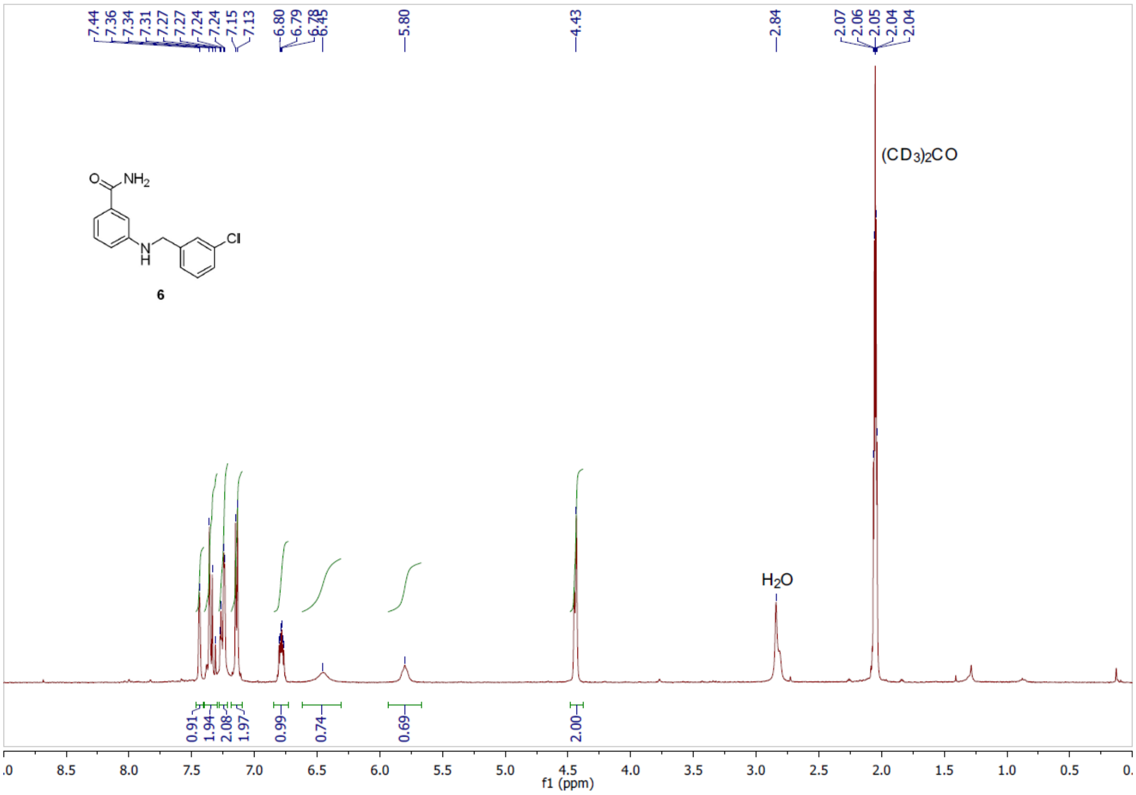

^{13}C NMR spectrum for **6** ($(CD_3)_2CO$, 75 MHz)

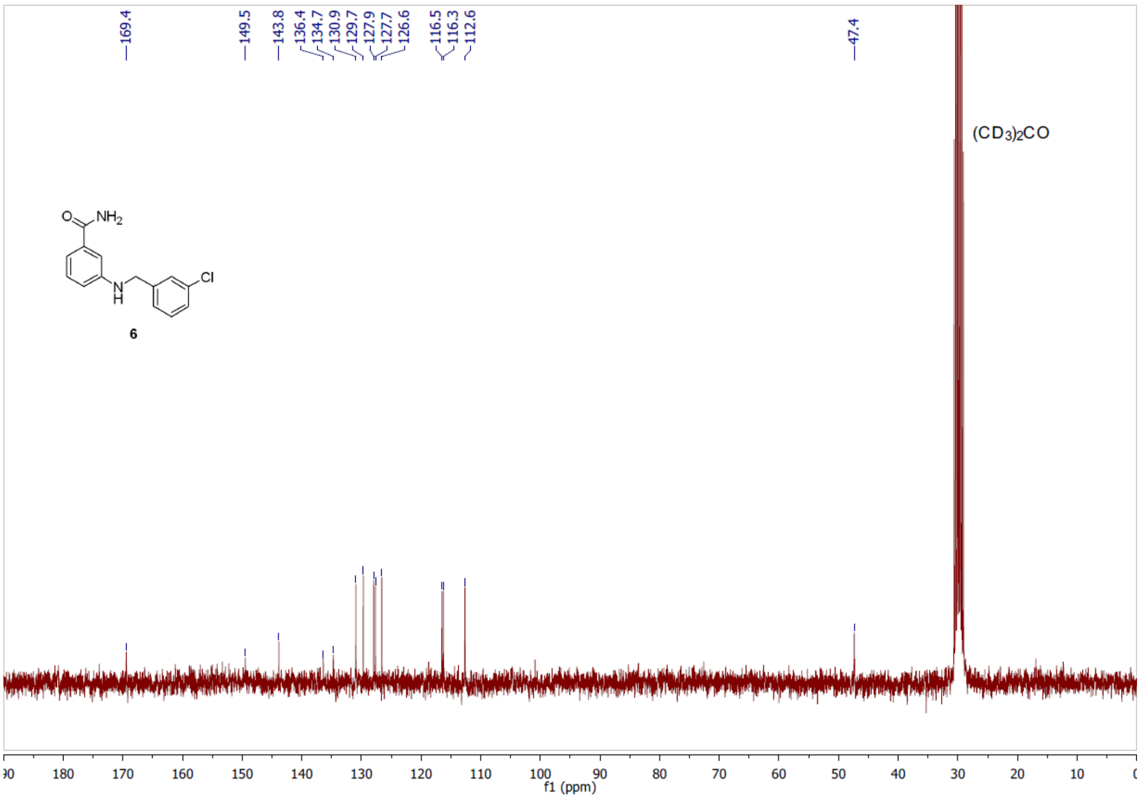

^{1}H NMR spectrum for **7** ($(CD_3)_2CO$, 300 MHz)

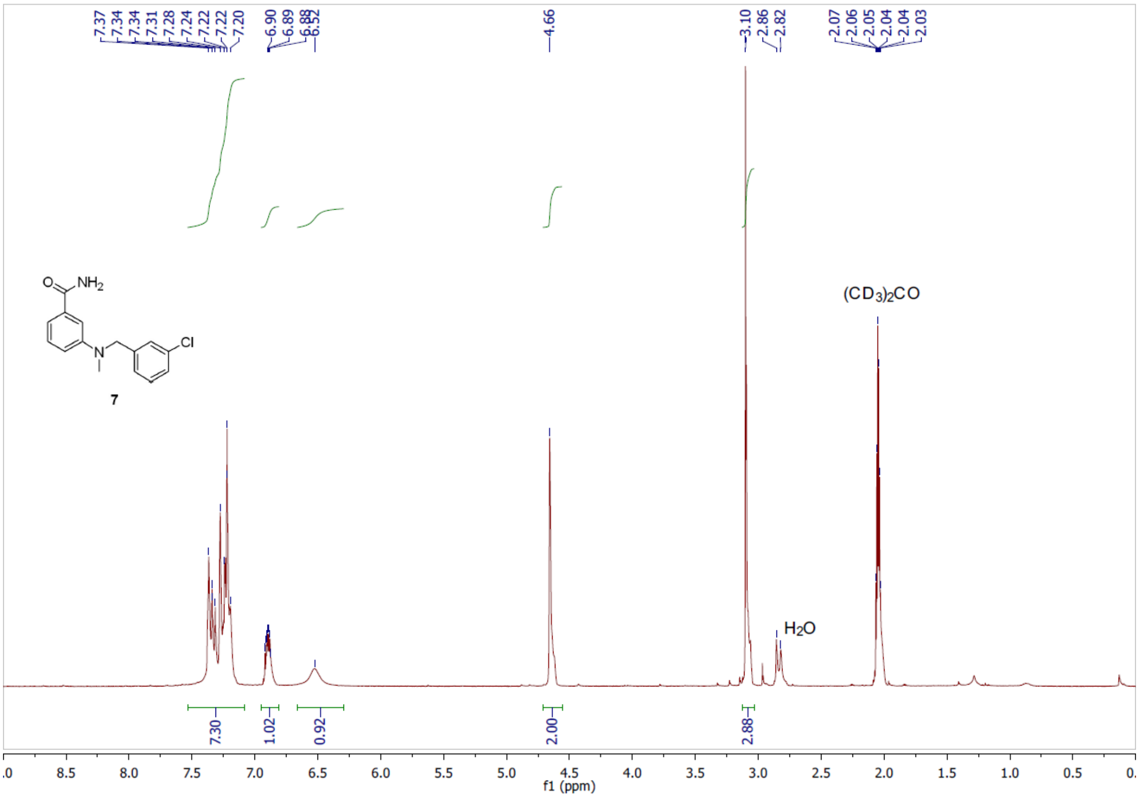

^{13}C NMR spectrum for **7** ($(CD_3)_2CO$, 75 MHz)

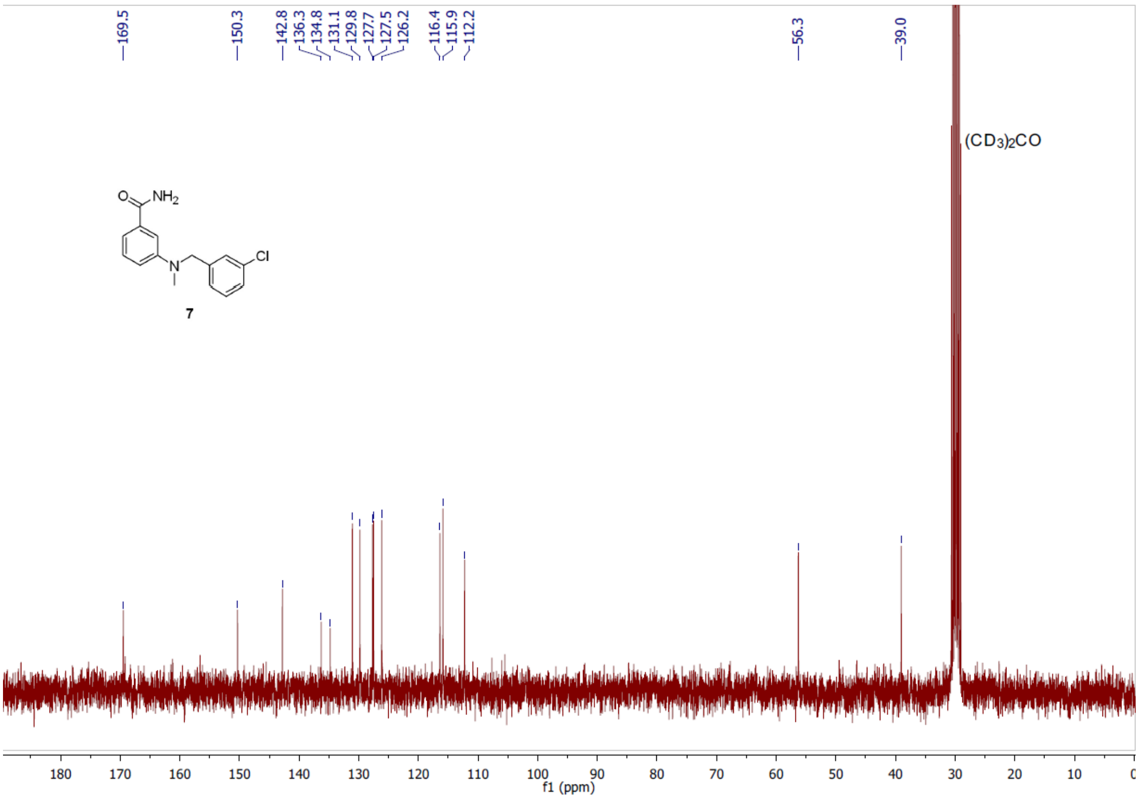

^{1}H NMR spectrum for **8** ($(CD_3)_2CO$, 300 MHz)

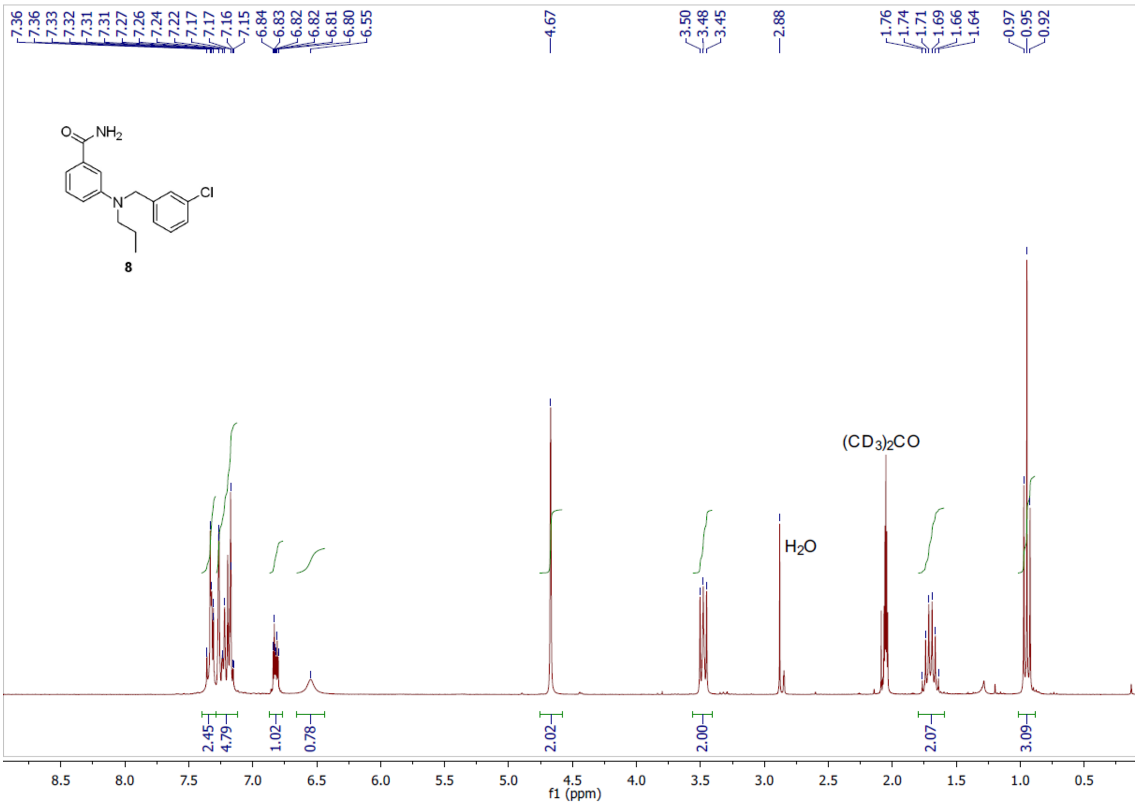

^{13}C NMR spectrum for **8** ($(CD_3)_2CO$, 75 MHz)

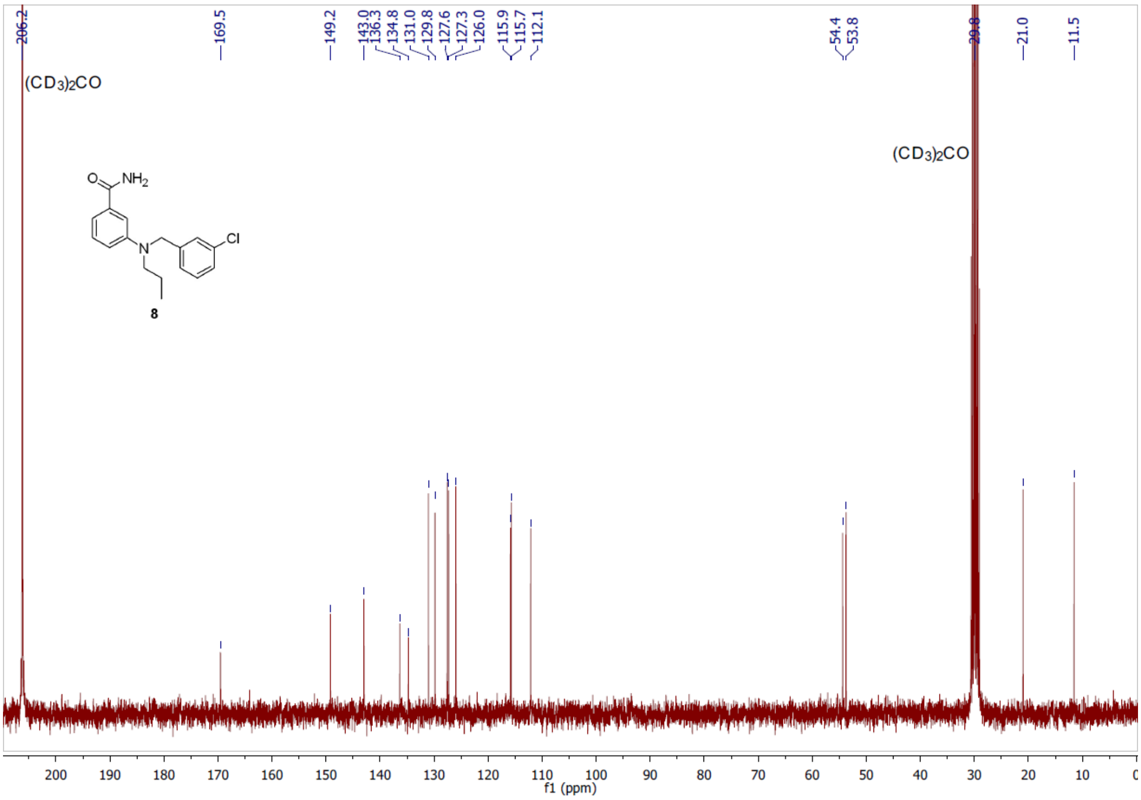

^{1}H NMR spectrum for **9** (($(CD_3)_2SO$, 300 MHz)

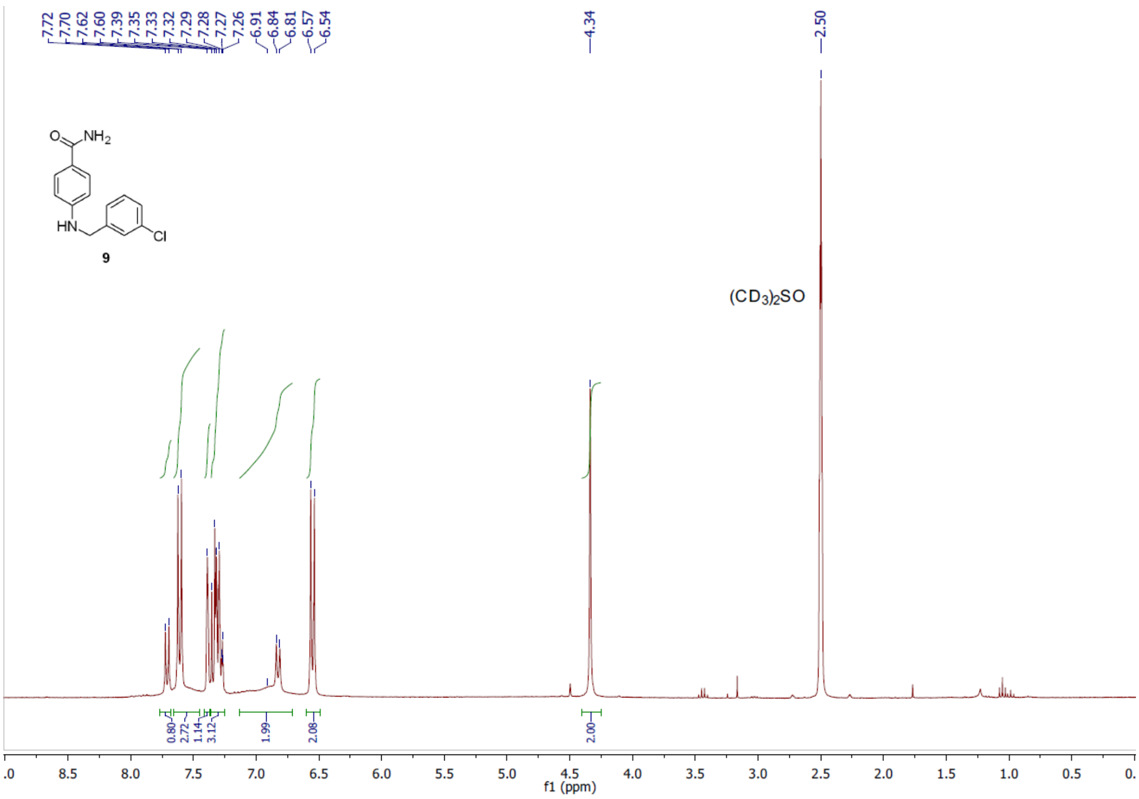

^{13}C NMR spectrum for **9** ($(CD_3)_2SO$, 75 MHz)

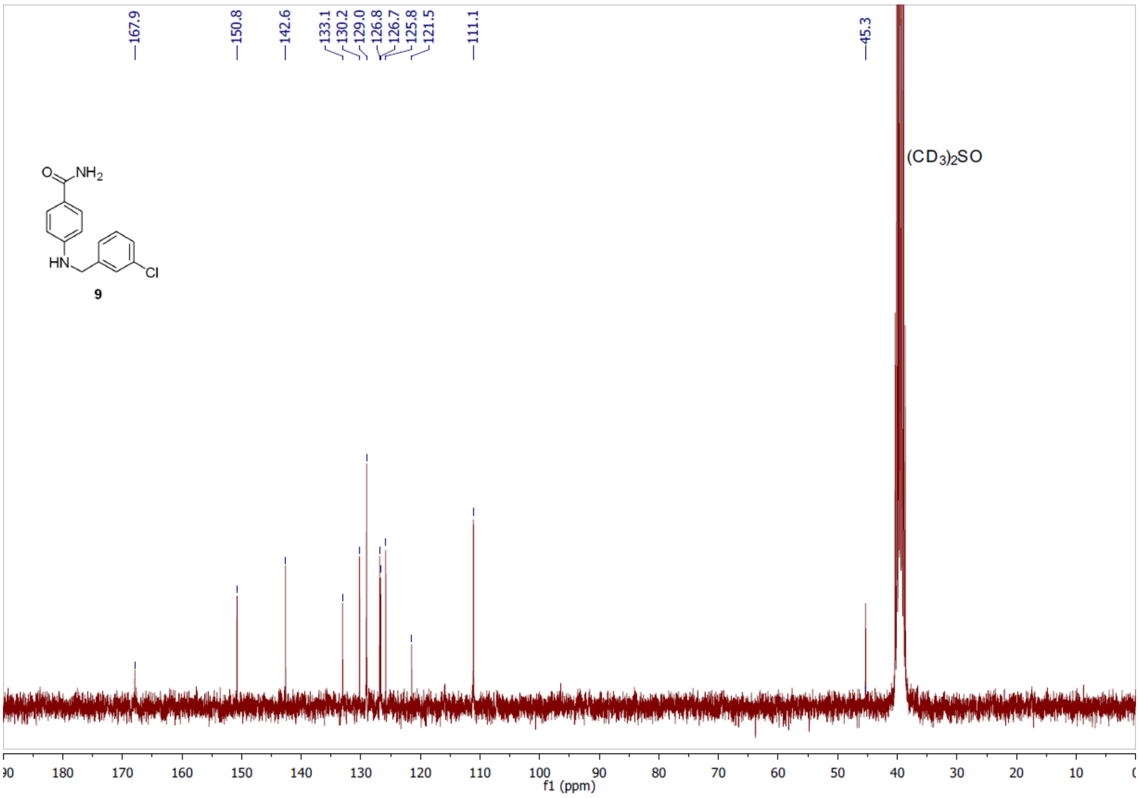

^{1}H NMR spectrum for **10** ($(CD_3)_2SO$, 300 MHz)

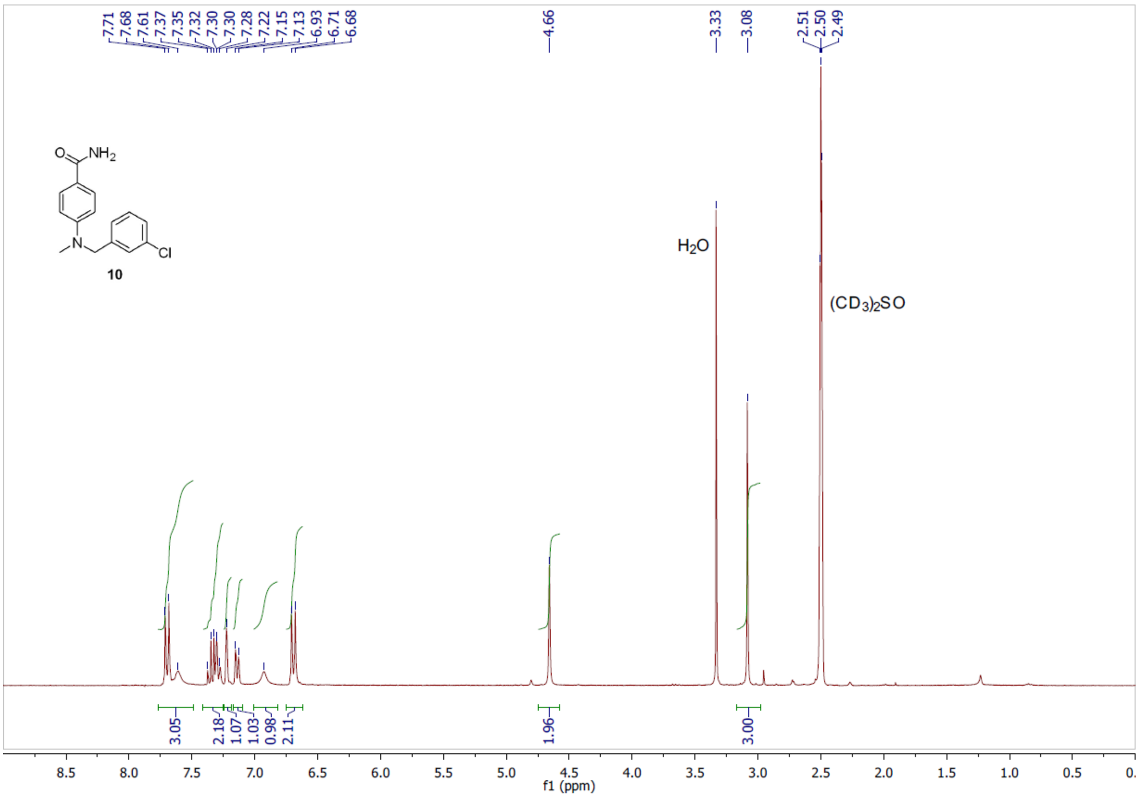

^{13}C NMR spectrum for **10** ($(CD_3)_2SO$, 75 MHz)

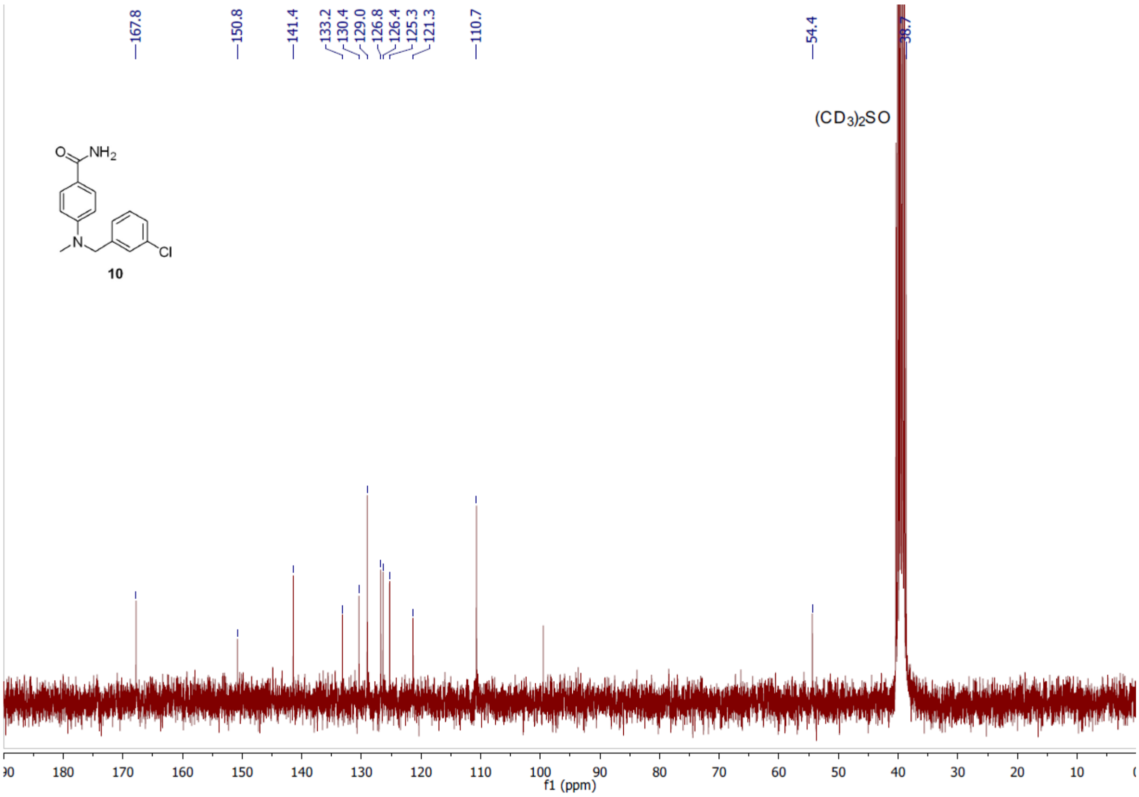

^{1}H NMR spectrum for **11** ($(CD_3)_2CO$, 300 MHz)

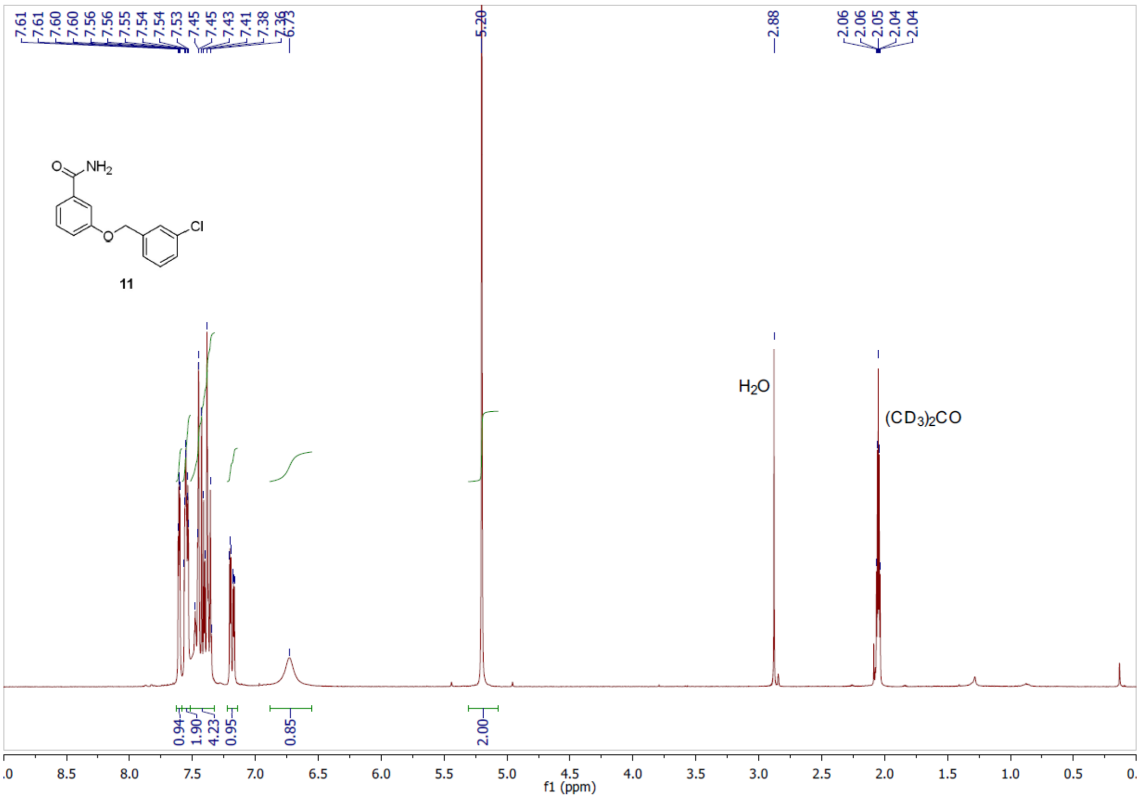

^{13}C NMR spectrum for **11** ($(CD_3)_2CO$, 75 MHz)

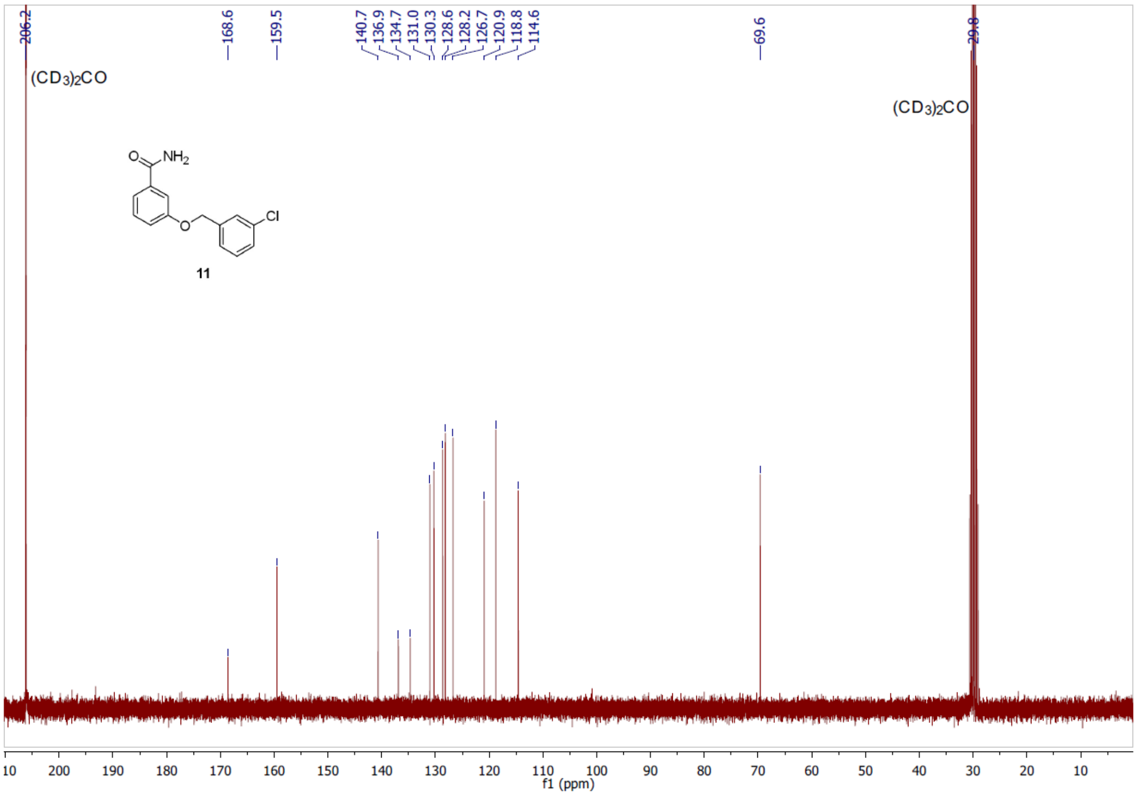

^{1}H NMR spectrum for **12** ($(CD_3)_2CO$, 700 MHz)

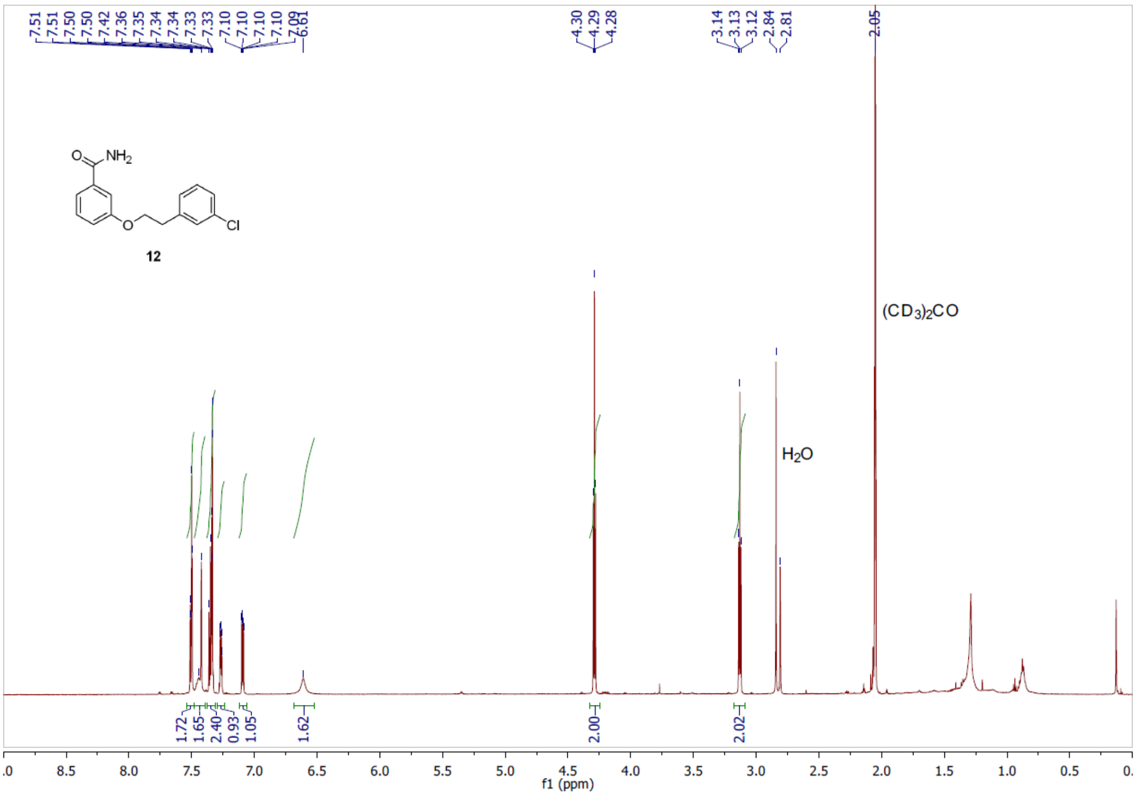

^{13}C NMR spectrum for **12** ($(CD_3)_2CO$, 175 MHz)

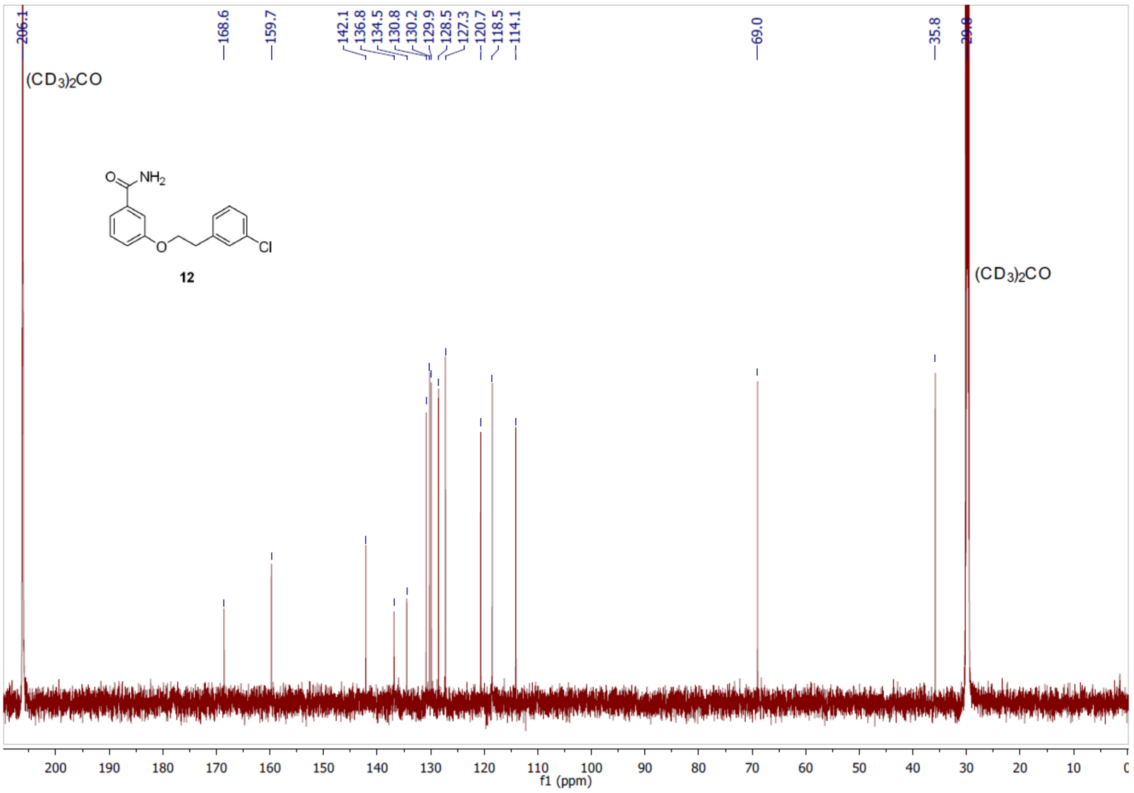

^{1}H NMR spectrum for **13** ($(CD_3)_2CO$, 300 MHz)

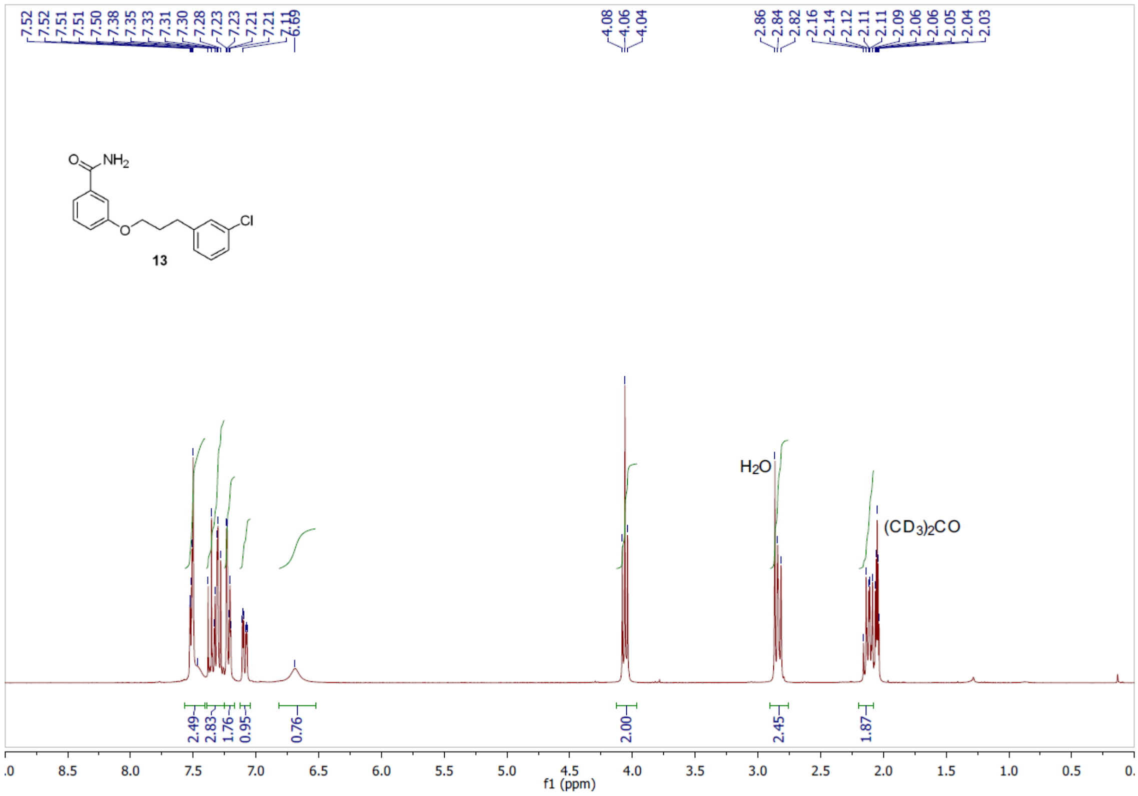

^{13}C NMR spectrum for **13** ($(CD_3)_2CO$, 75 MHz)

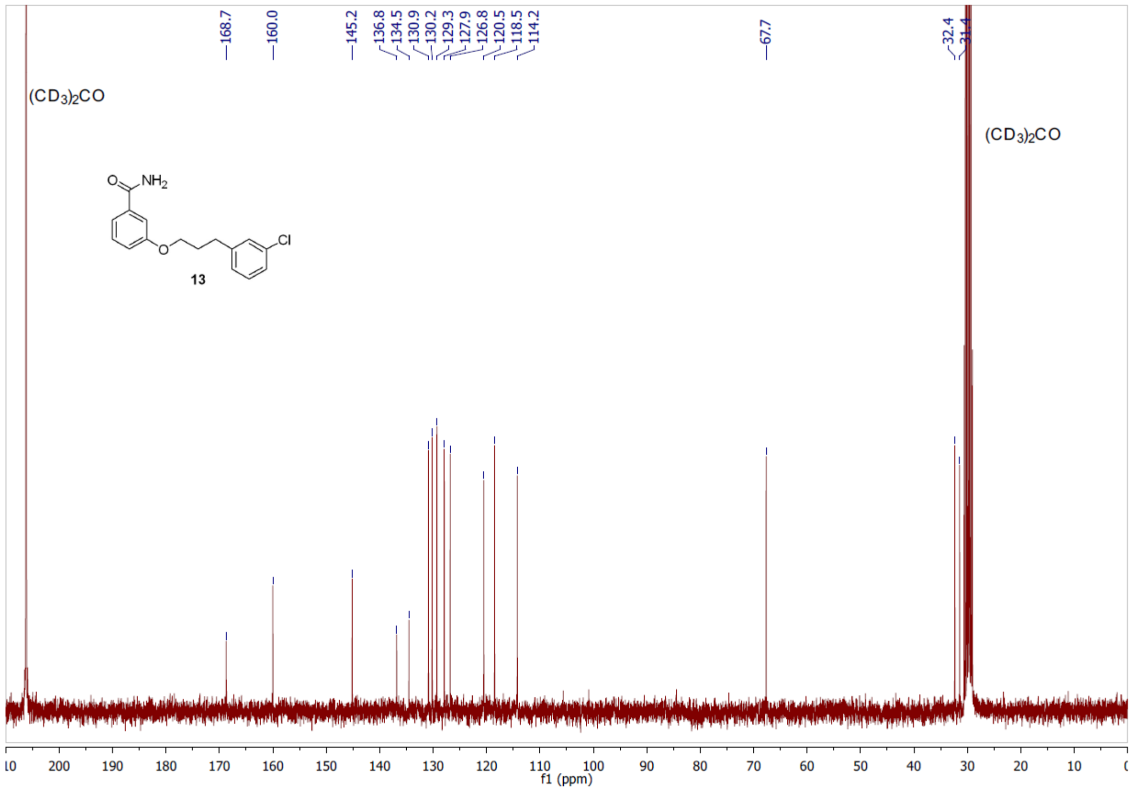

^{1}H NMR spectrum for **14** (CD_3OD, 300 MHz)

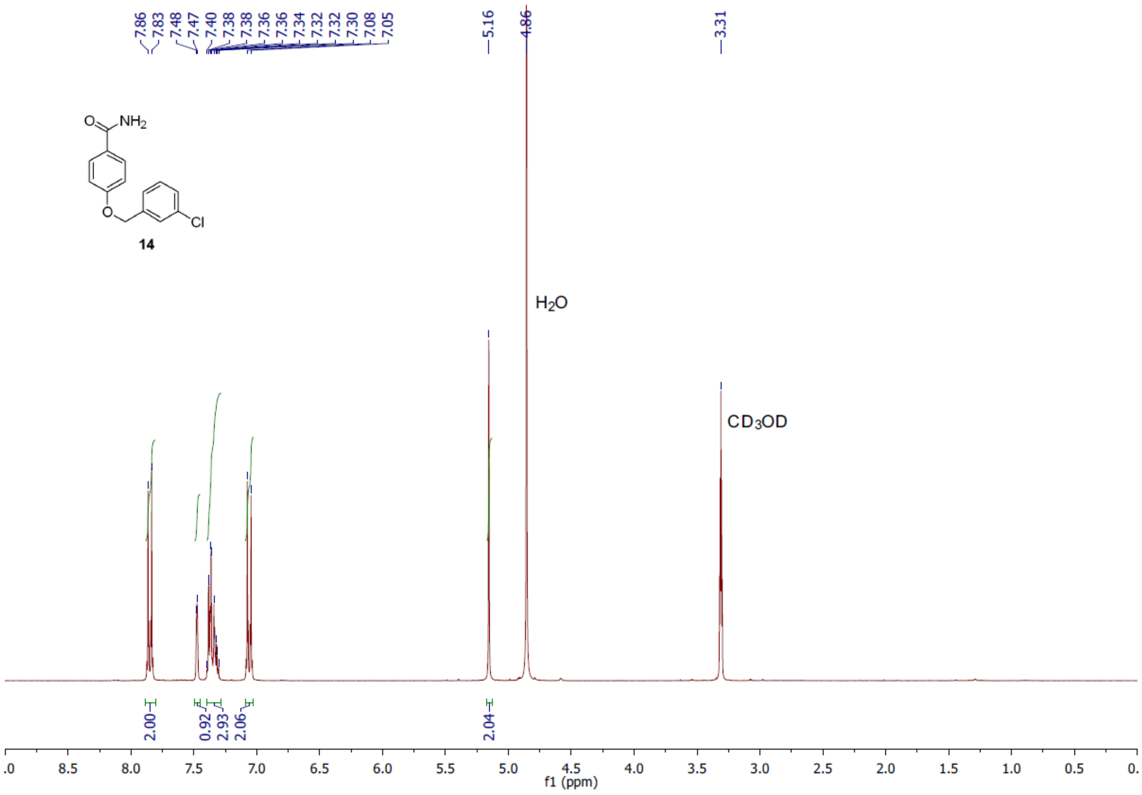

^{13}C NMR spectrum for **14** (CD_3OD, 75 MHz)

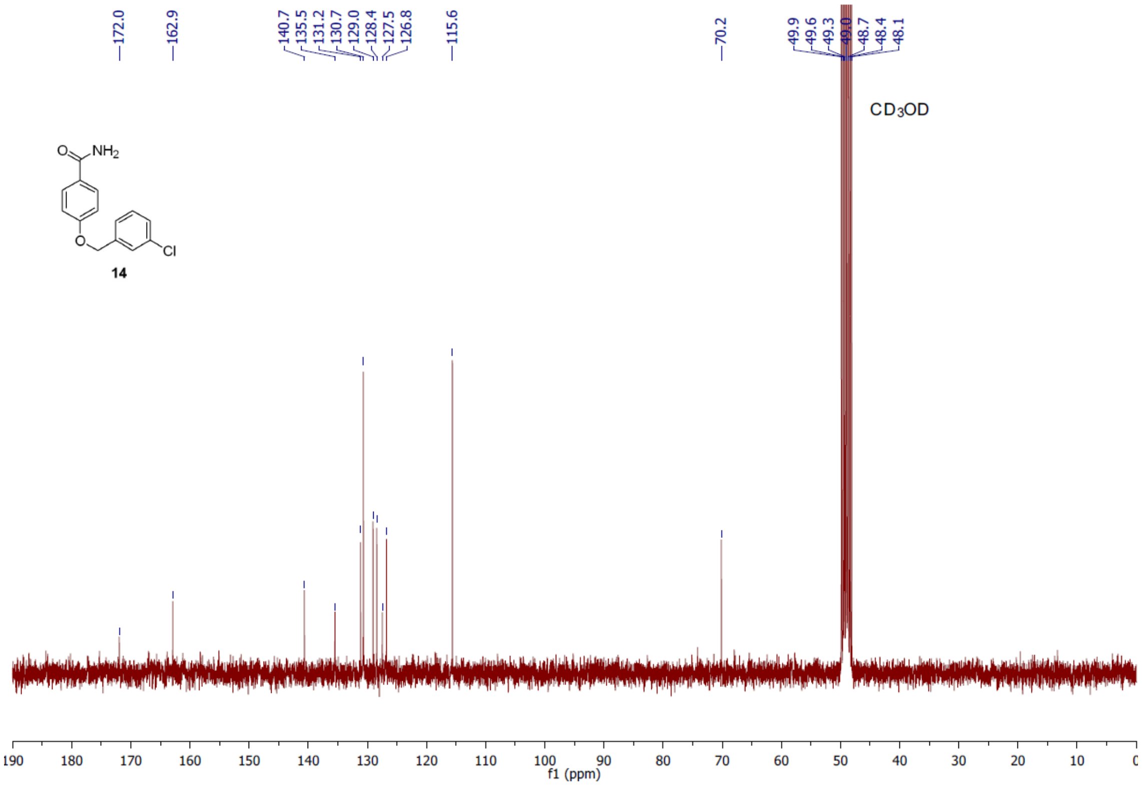

^{1}H NMR spectrum for **15** ($CDCl_3$, 700 MHz)

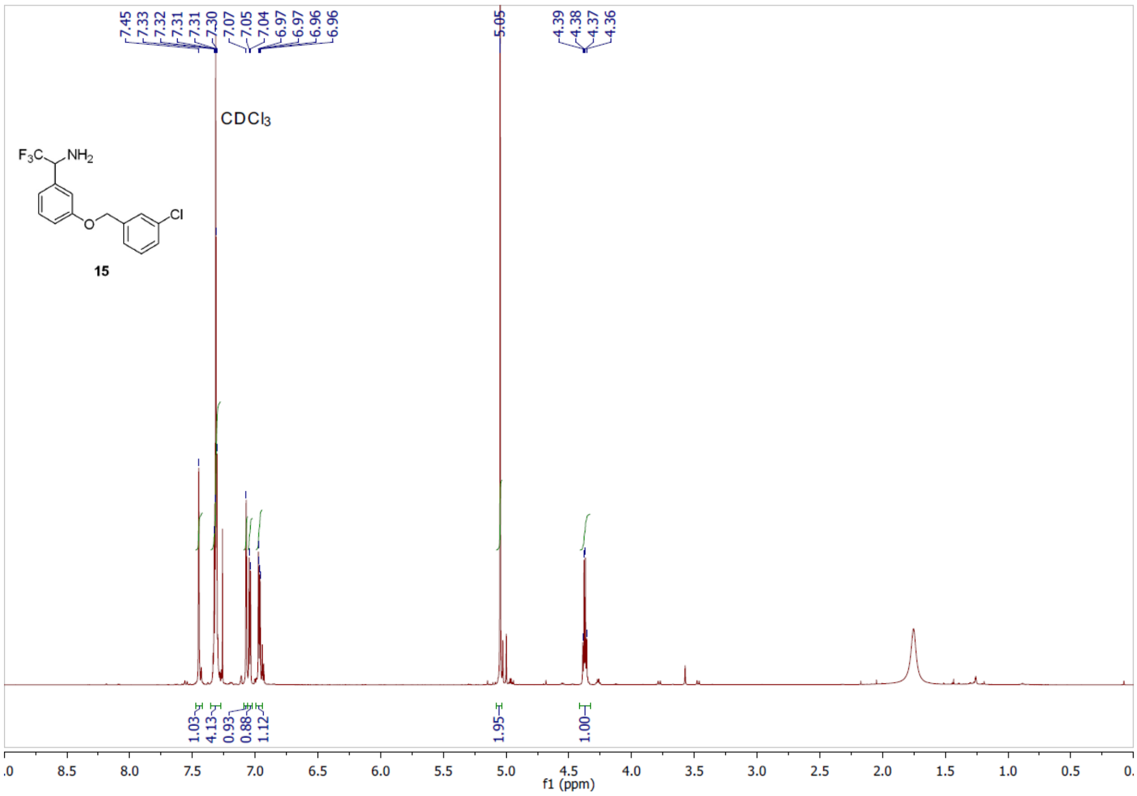

^{13}C NMR spectrum for **15** ($CDCl_3$, 175 MHz)

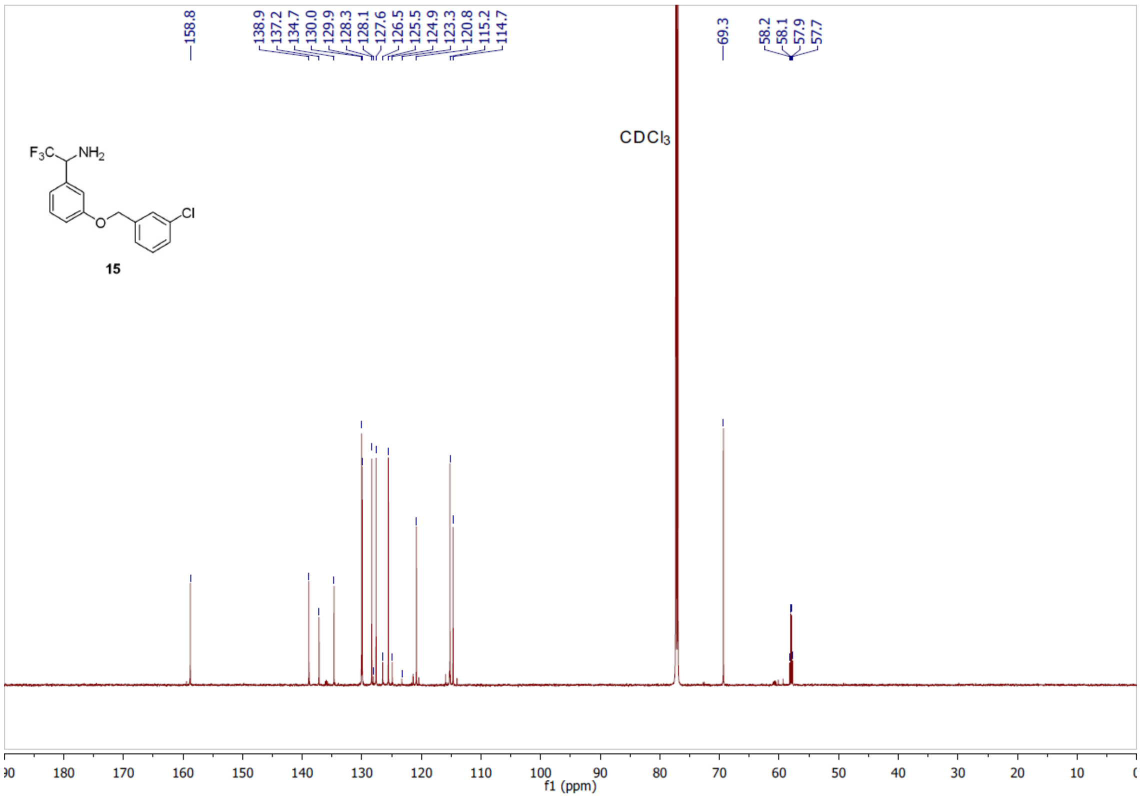

^{1}H NMR spectrum for **16** (CD_3OD, 500 MHz)

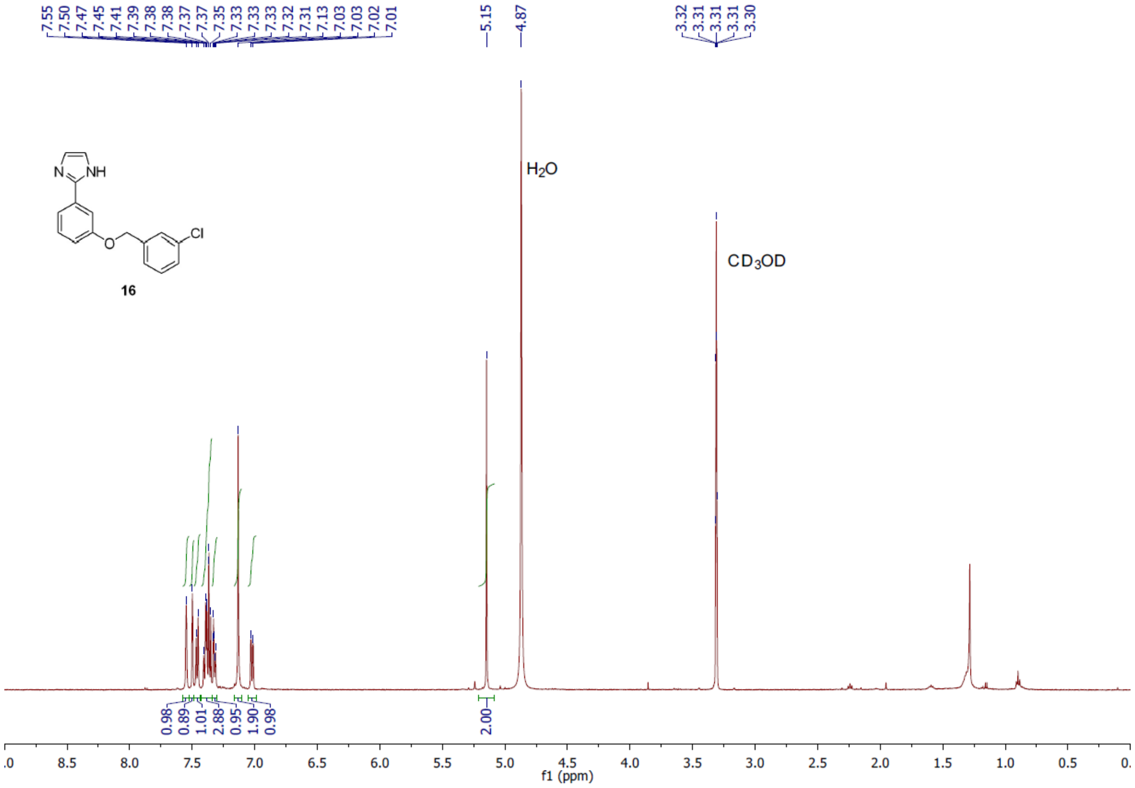

^{13}C NMR spectrum for **16** (CD_3OD, 125 MHz)

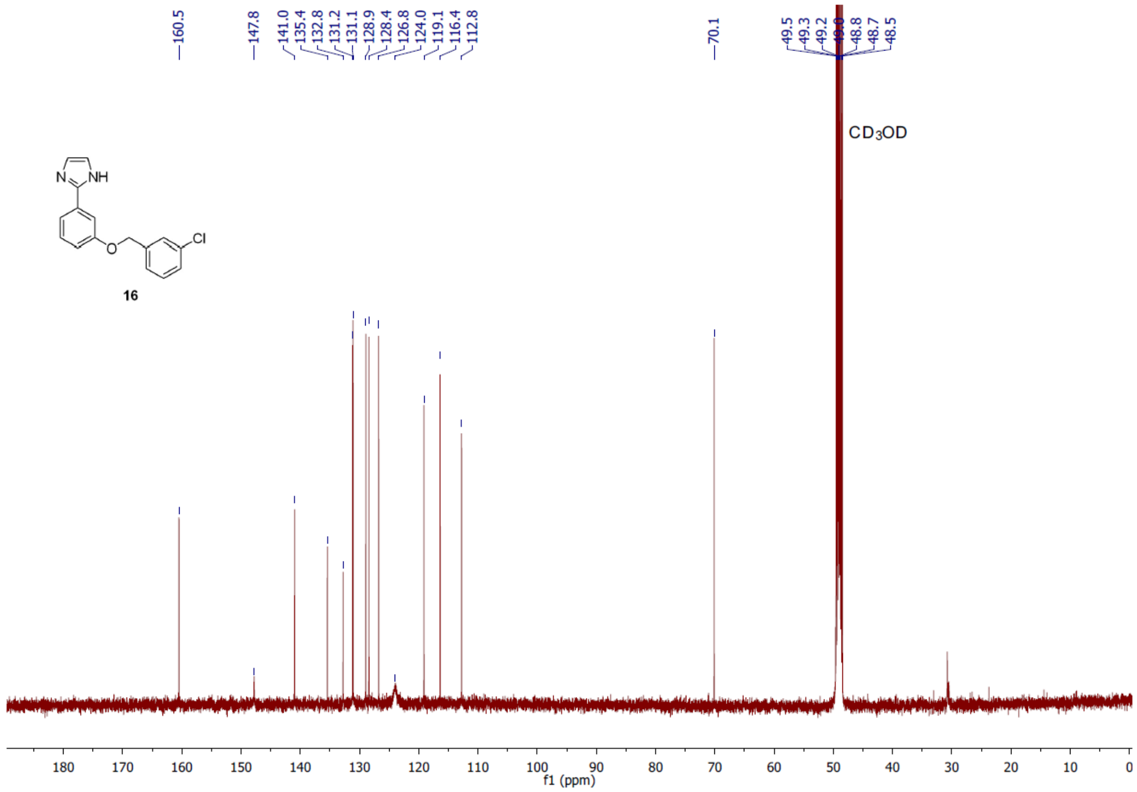

^{1}H NMR spectrum for **17** (CD_3OD, 500 MHz)

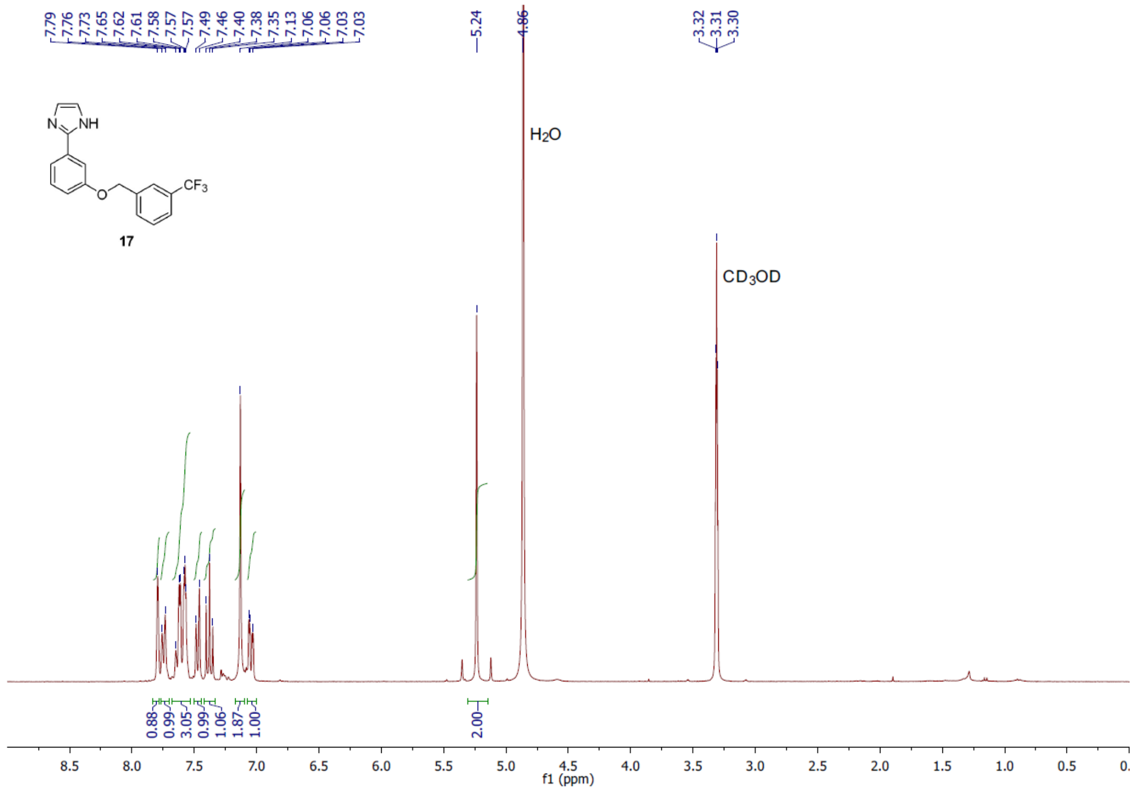

^{13}C NMR spectrum for **17** (CD_3OD, 125 MHz)

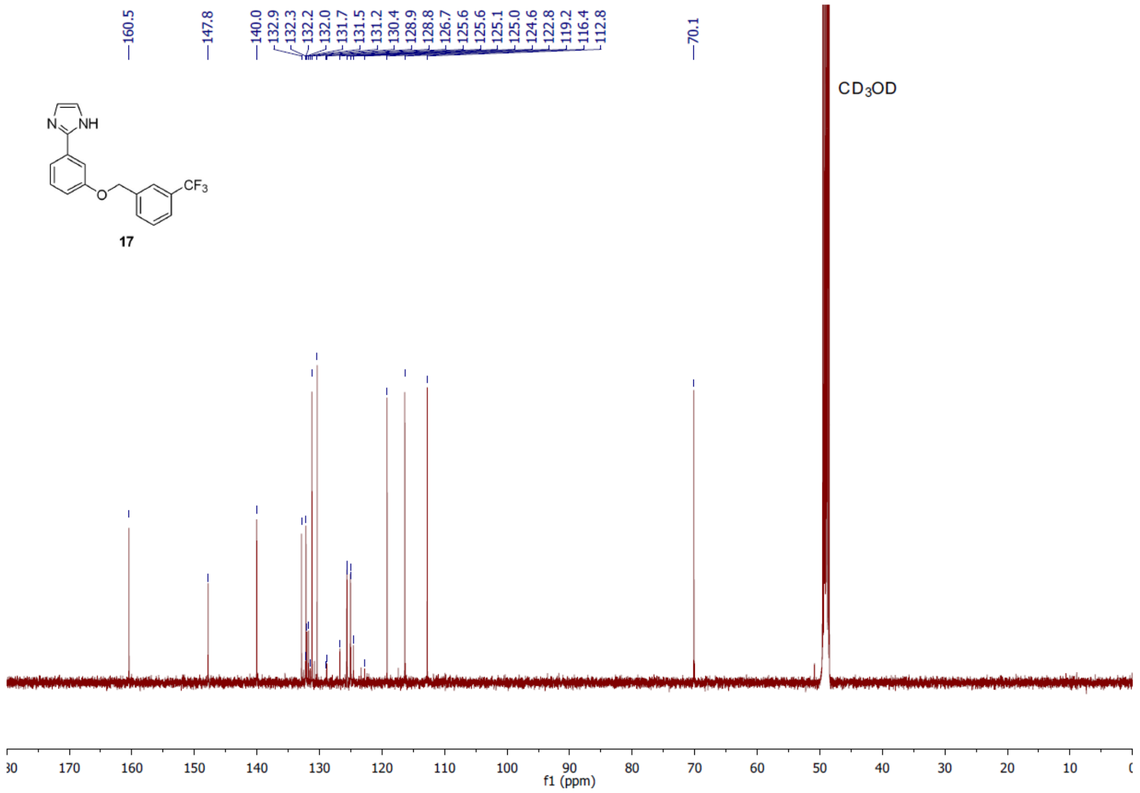

^{1}H NMR spectrum for **18** ($(CD_3)_2CO$, 300 MHz)

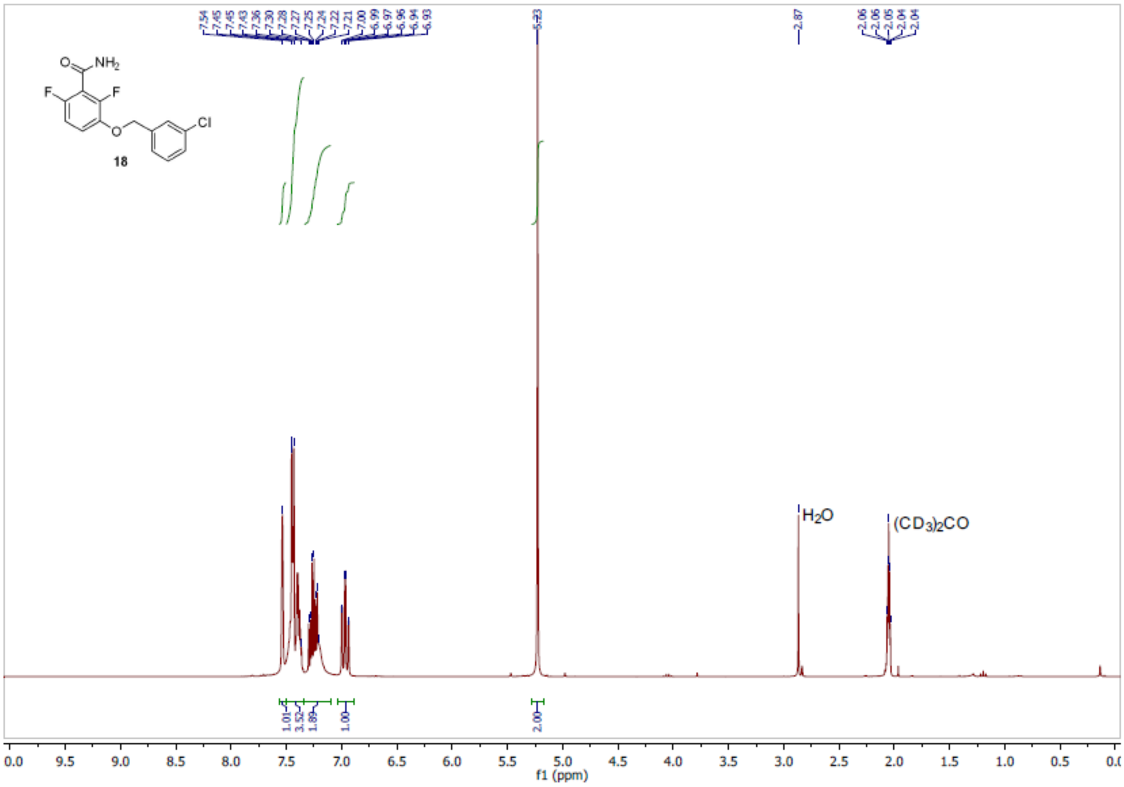

^{13}C NMR spectrum for **18** ($(CD_3)_2CO$, 75 MHz)

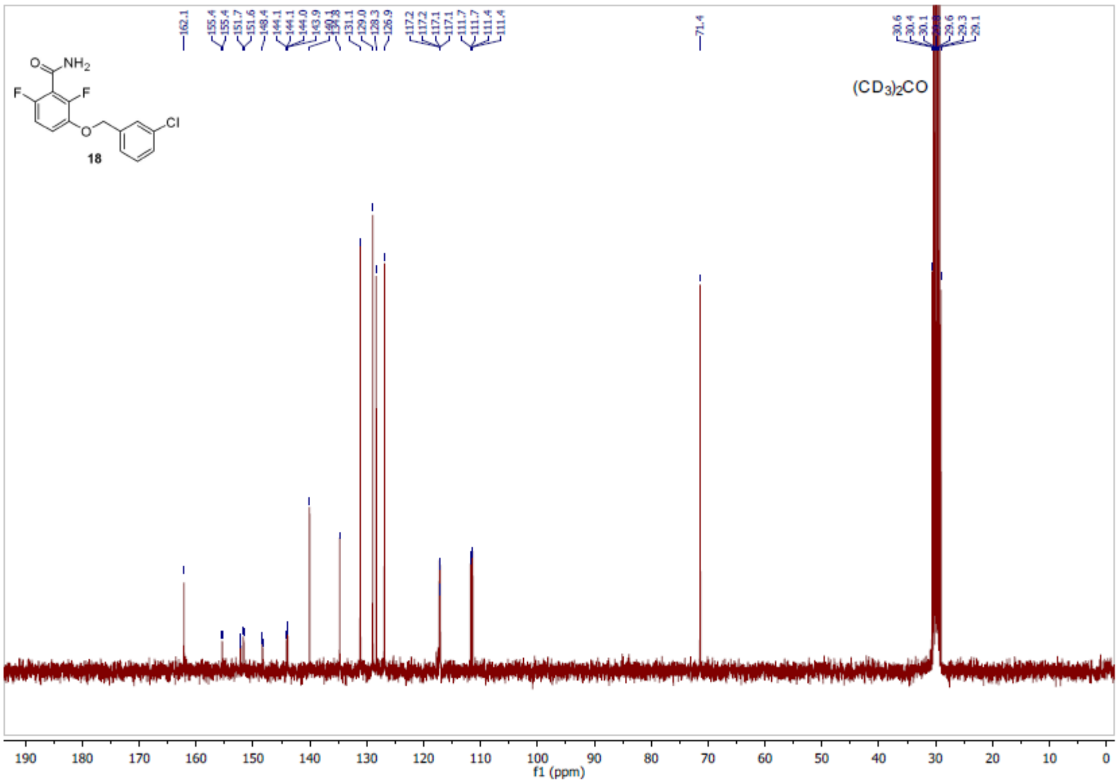

^{1}H NMR spectrum for **19** ($CDCl_3$, 500 MHz)

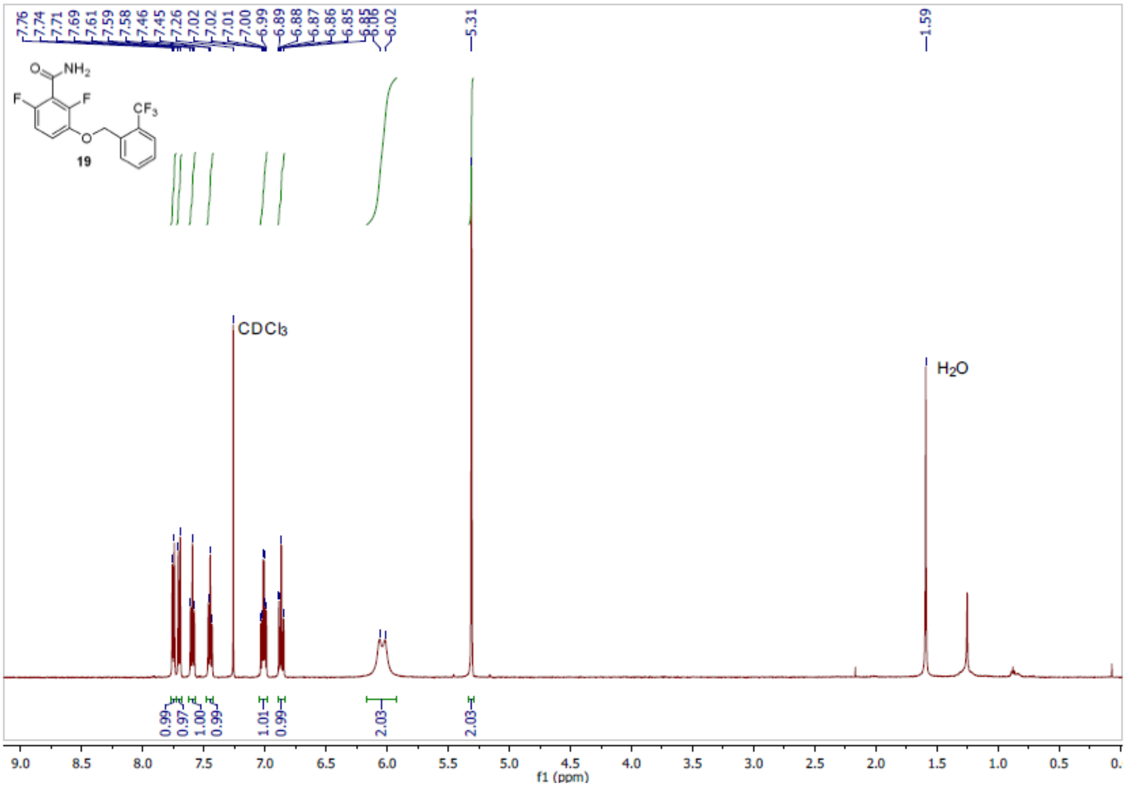

^{13}C NMR spectrum for **19** ($CDCl_3$, 125 MHz)

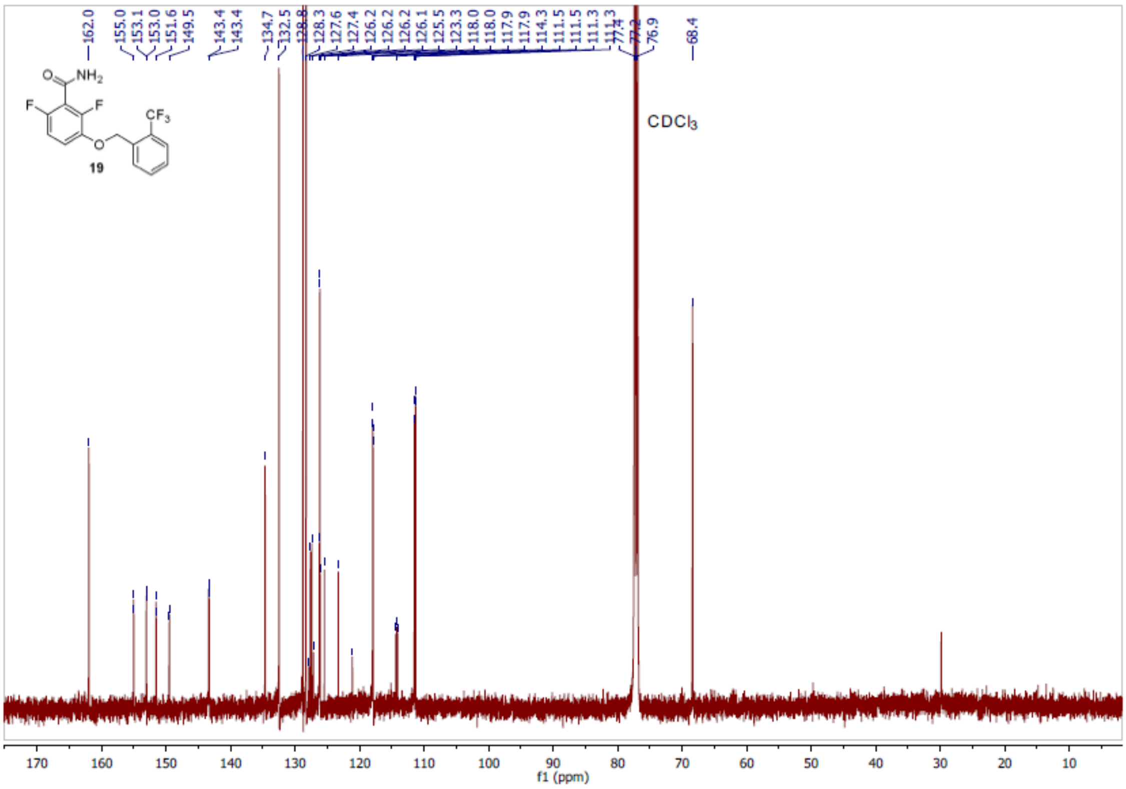

^1H NMR spectrum for **20** ($(CD_3)_2CO$, 500 MHz)

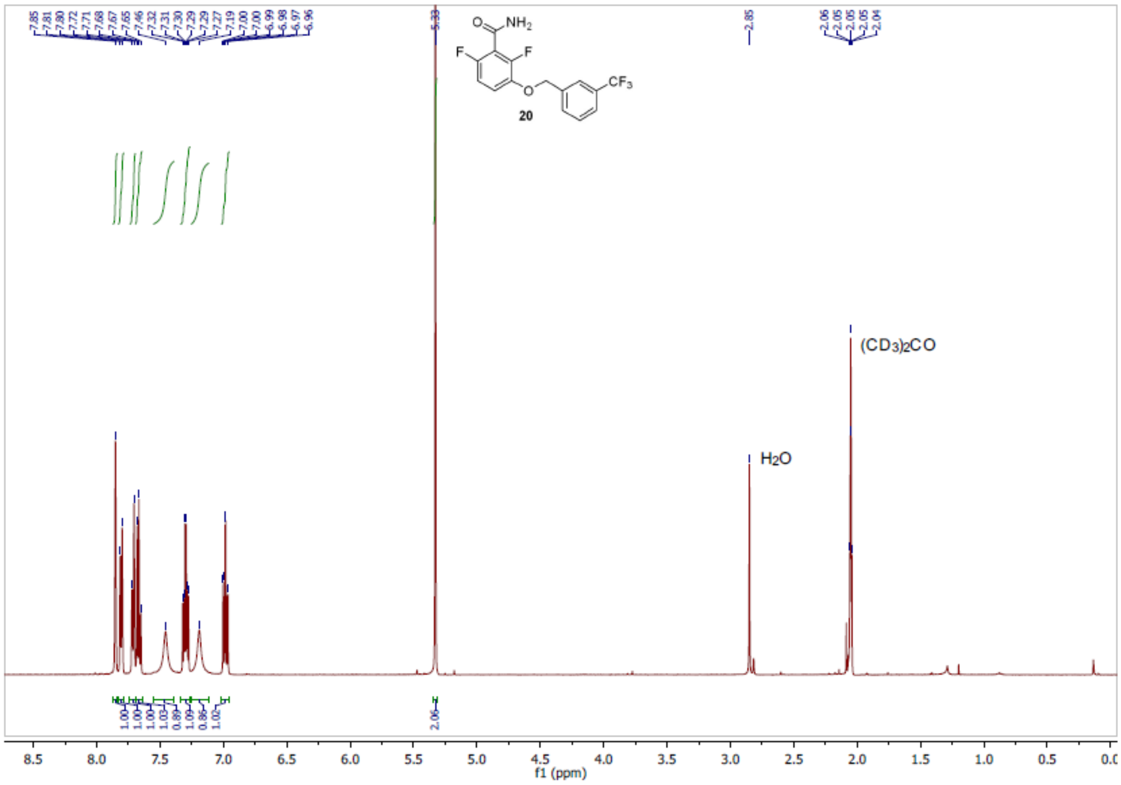

^{13}C NMR spectrum for **20** ($(CD_3)_2CO$, 125 MHz)

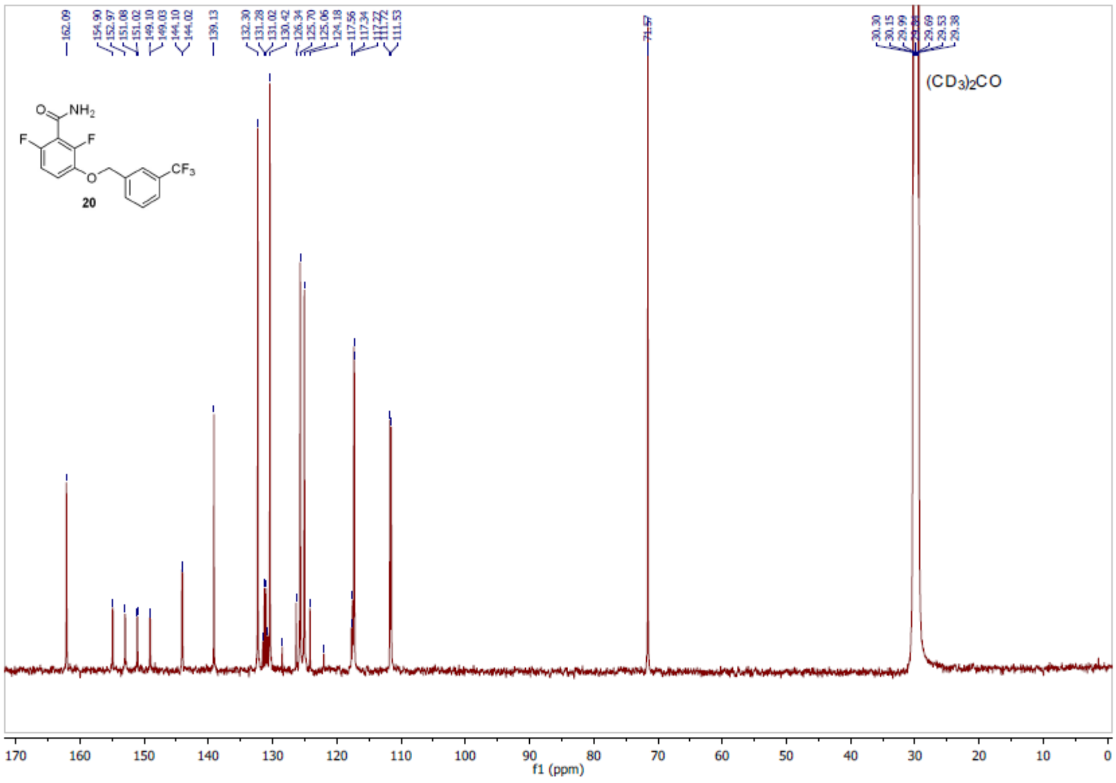

^{1}H NMR spectrum for **21** ($CDCl_3$, 300 MHz)

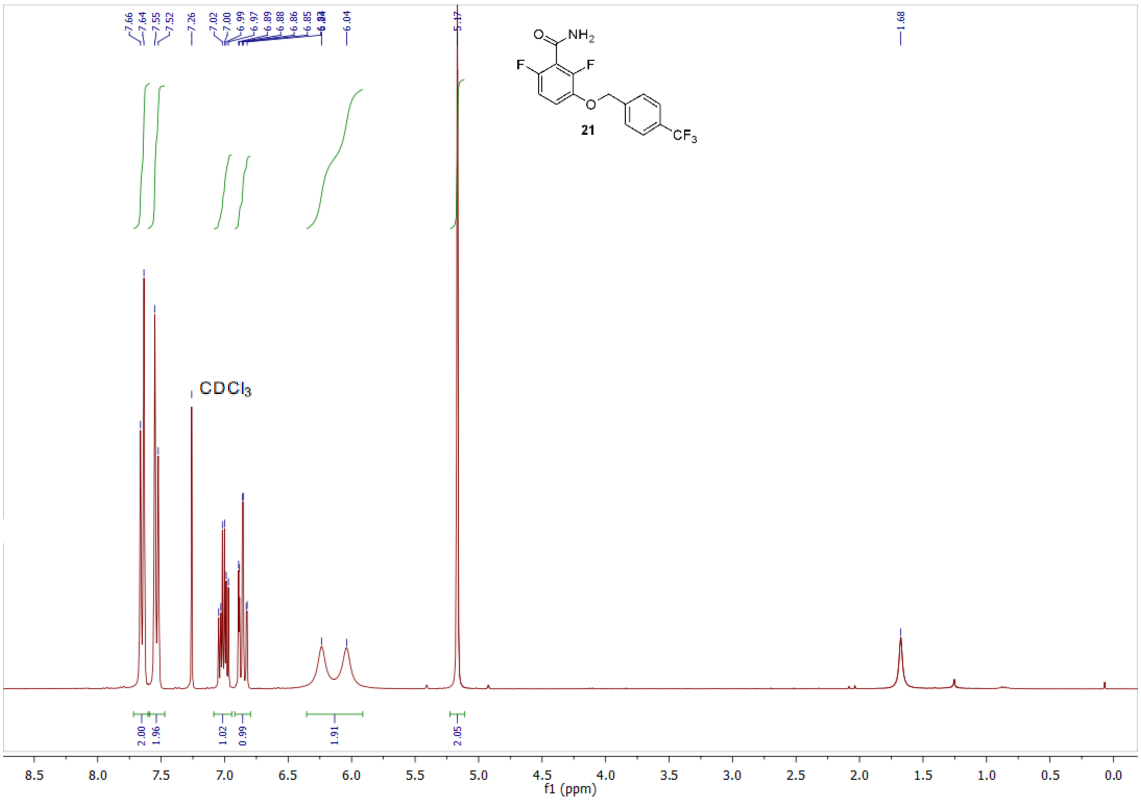

^{13}C NMR spectrum for **20** ($CDCl_3$, 125 MHz)

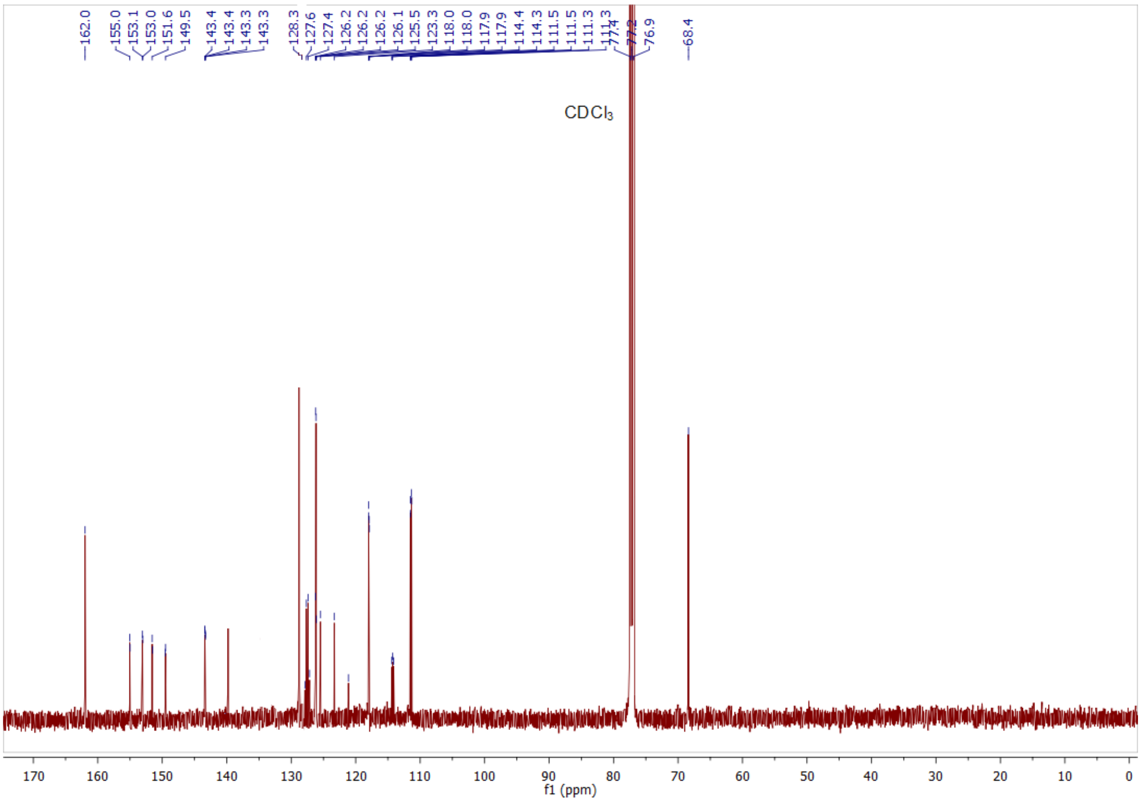

^{1}H NMR spectrum for **22** ($CDCl_3$, 300 MHz)

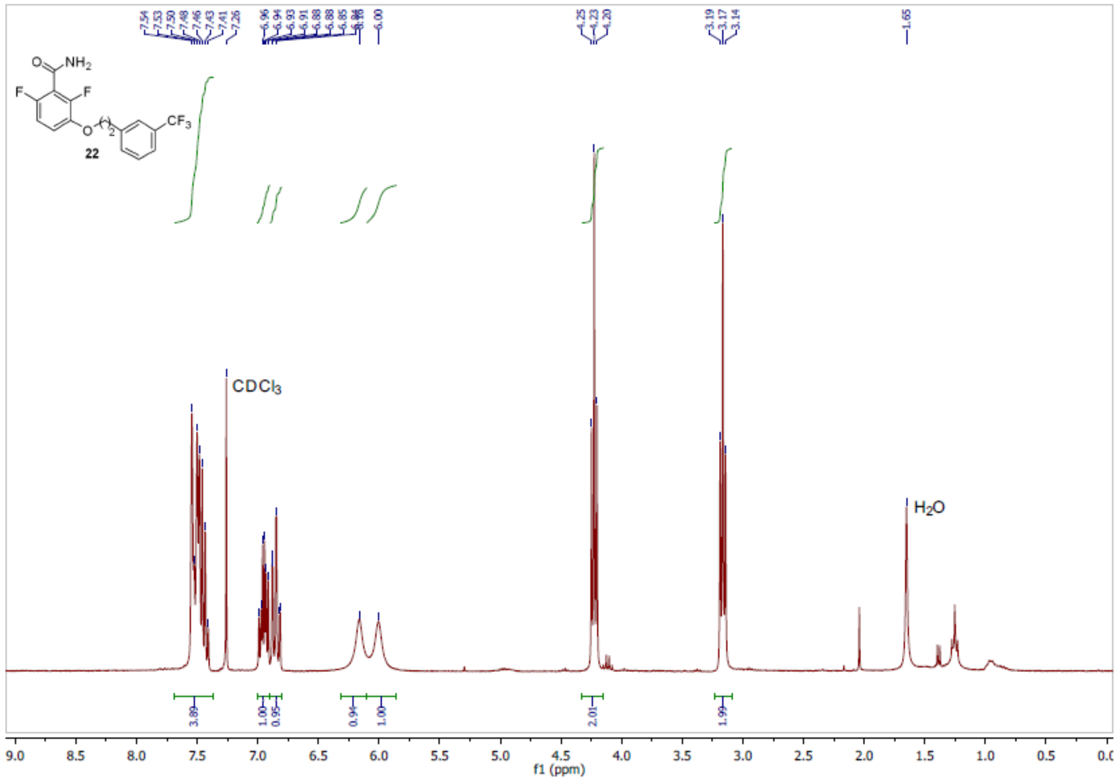

^{13}C NMR spectrum for **22** ($CDCl_3$, 75 MHz)

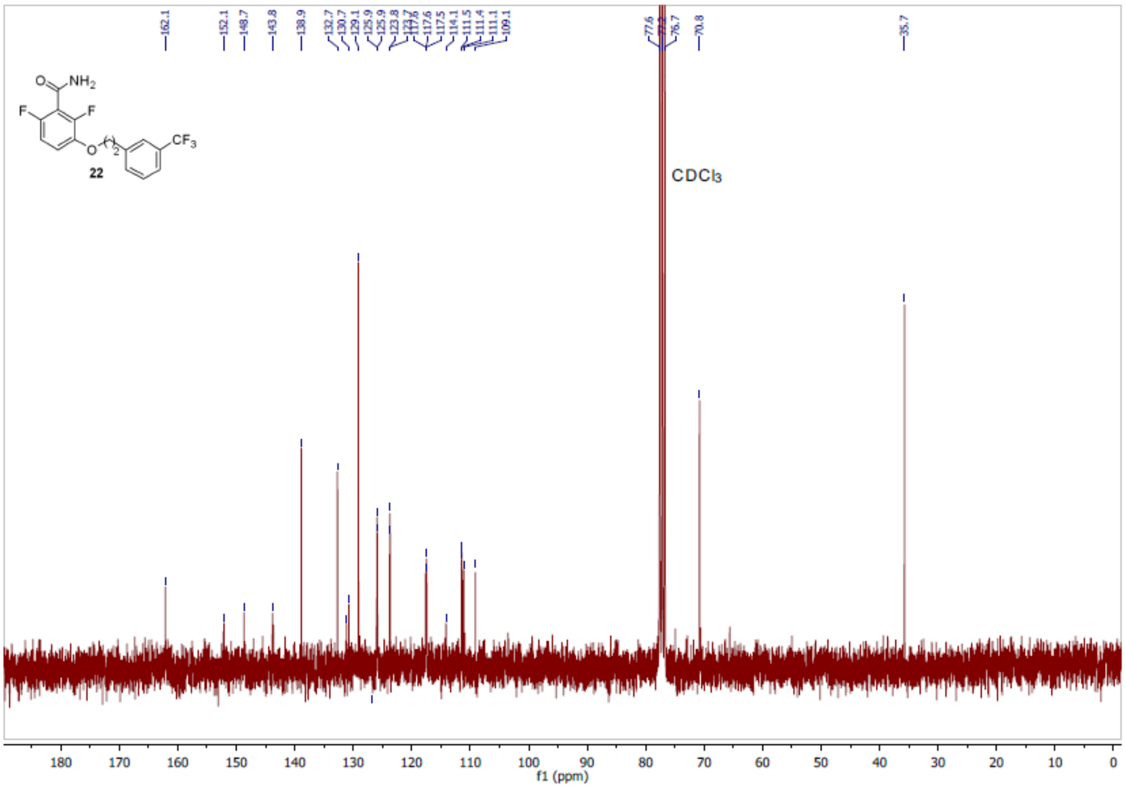

^{1}H NMR spectrum for **23** ($CDCl_3$, 300 MHz)

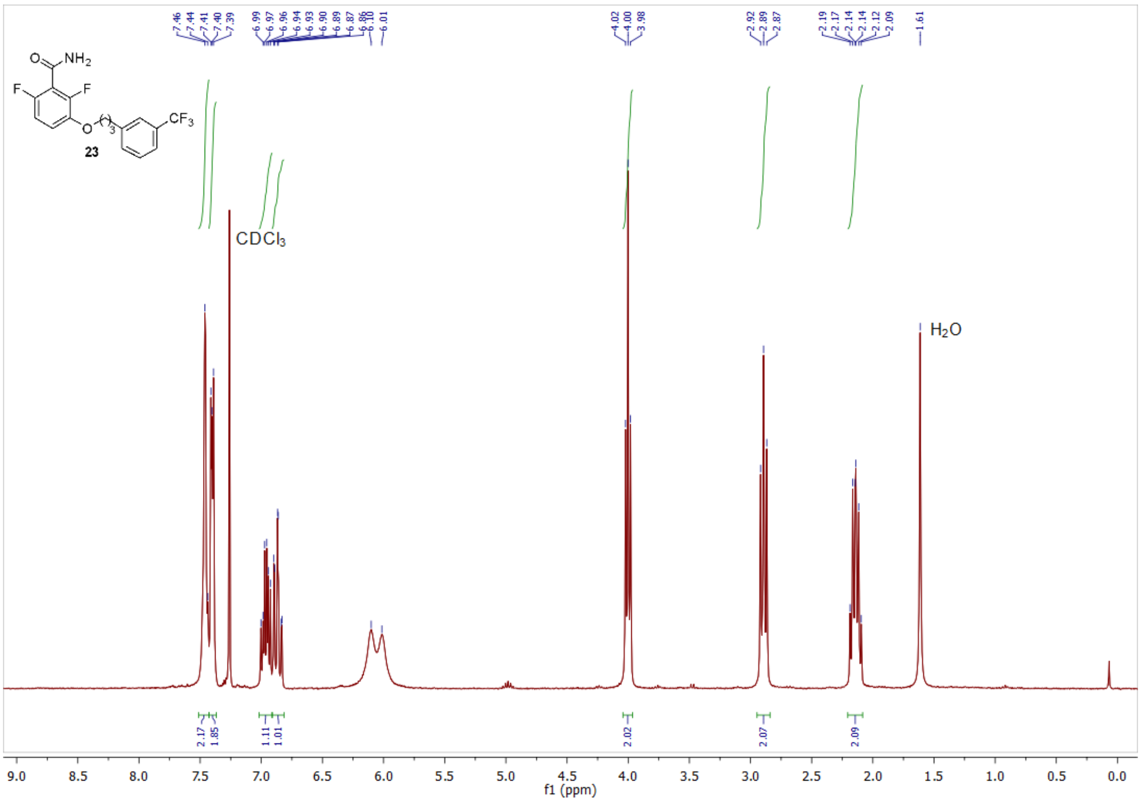

^{13}C NMR spectrum for **23** ($CDCl_3$, 75 MHz)

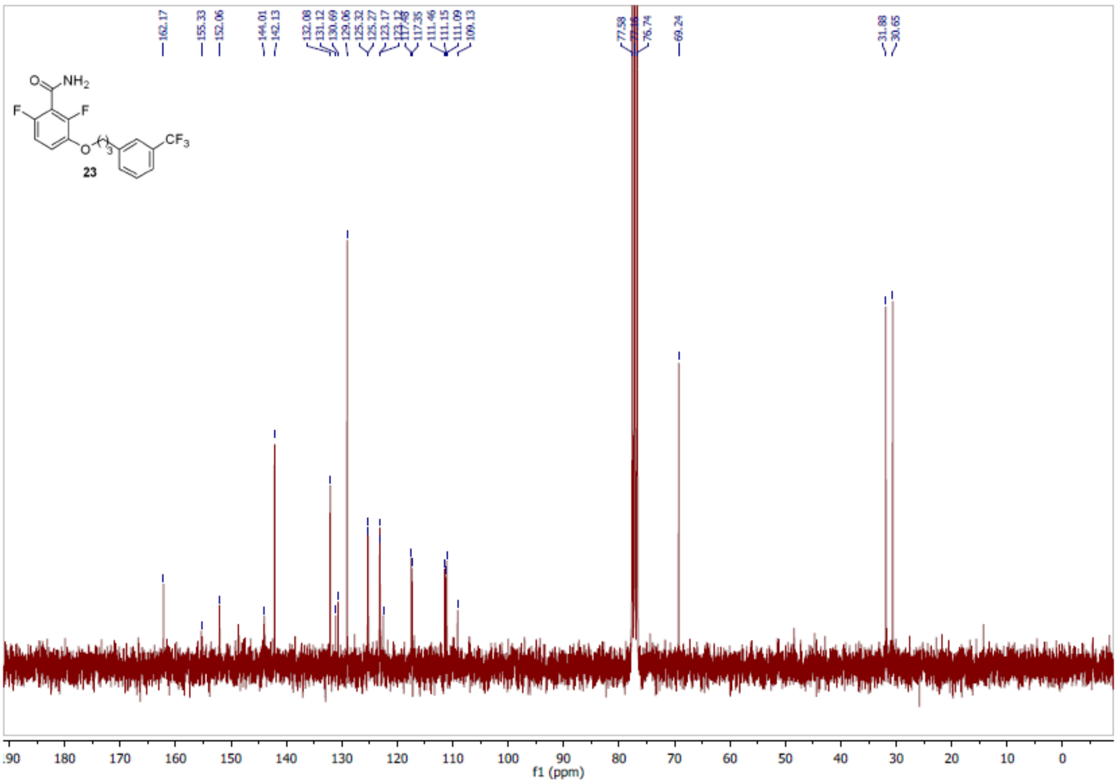

^{1}H NMR spectrum for **24** ($CDCl_3$, 300 MHz)

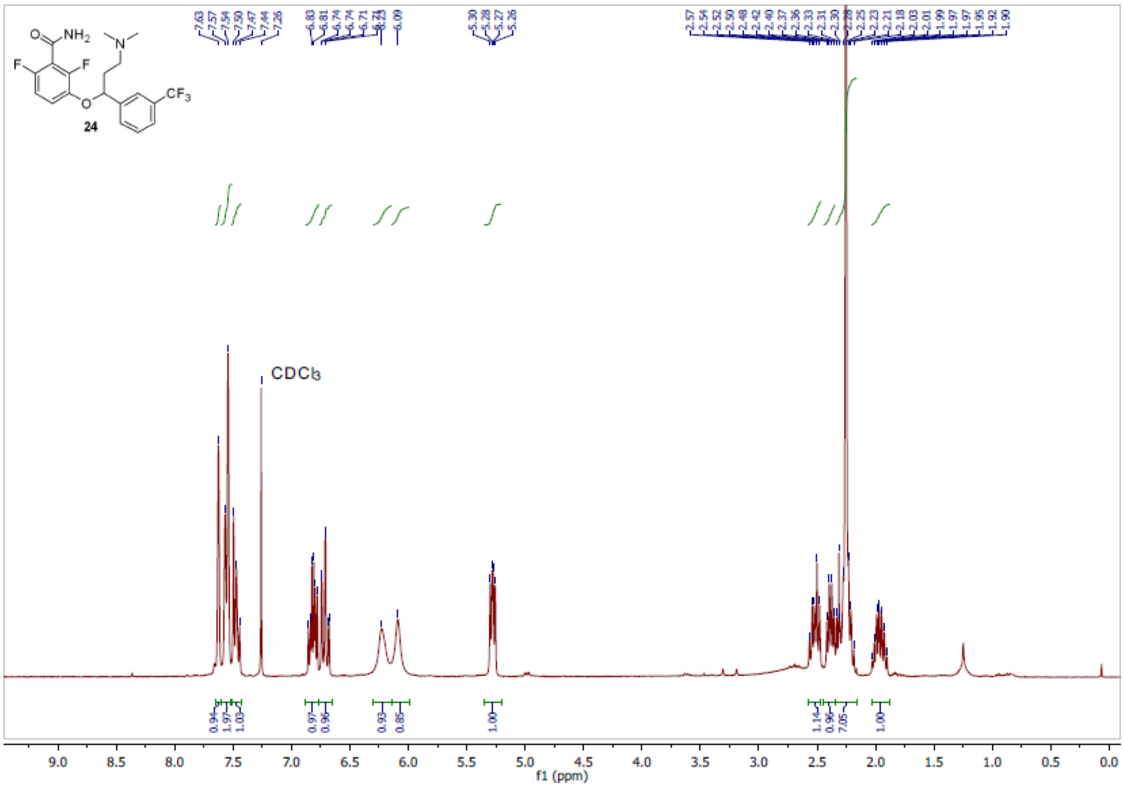

^{1}H NMR spectrum for **25** (CD_3OD, 300 MHz)

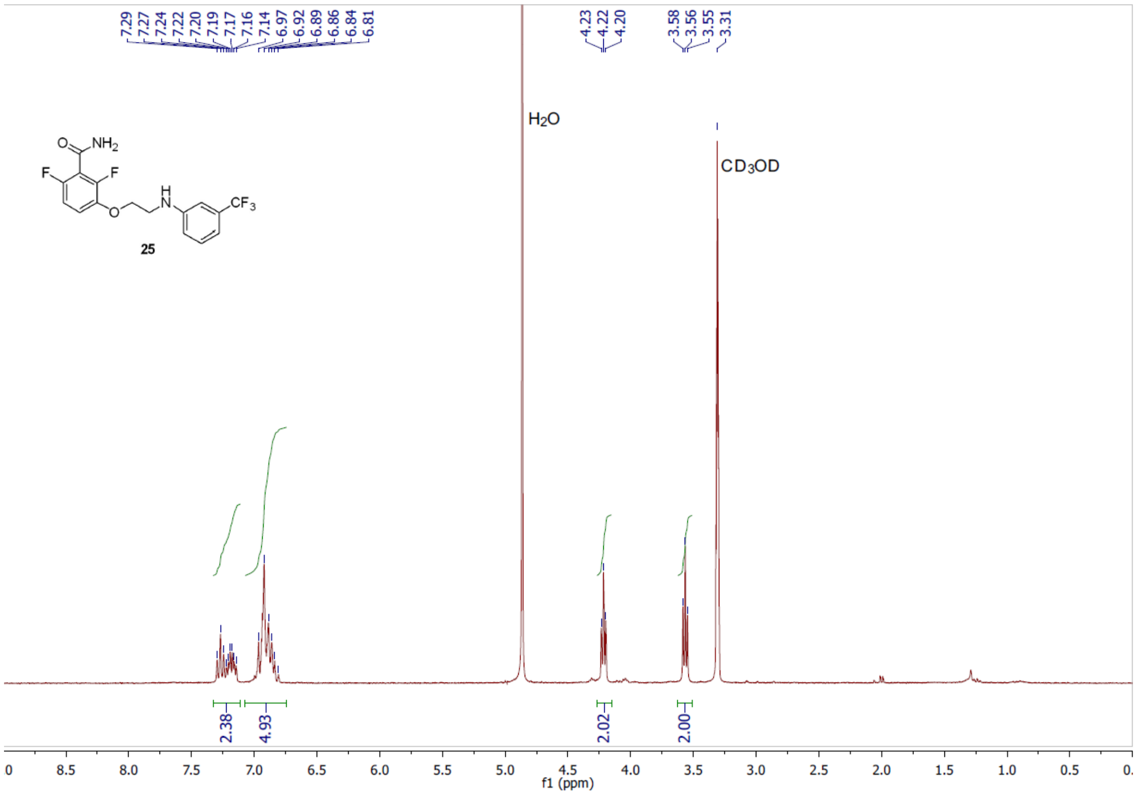

^{13}C NMR spectrum for **25** (CD_3OD, 75 MHz)

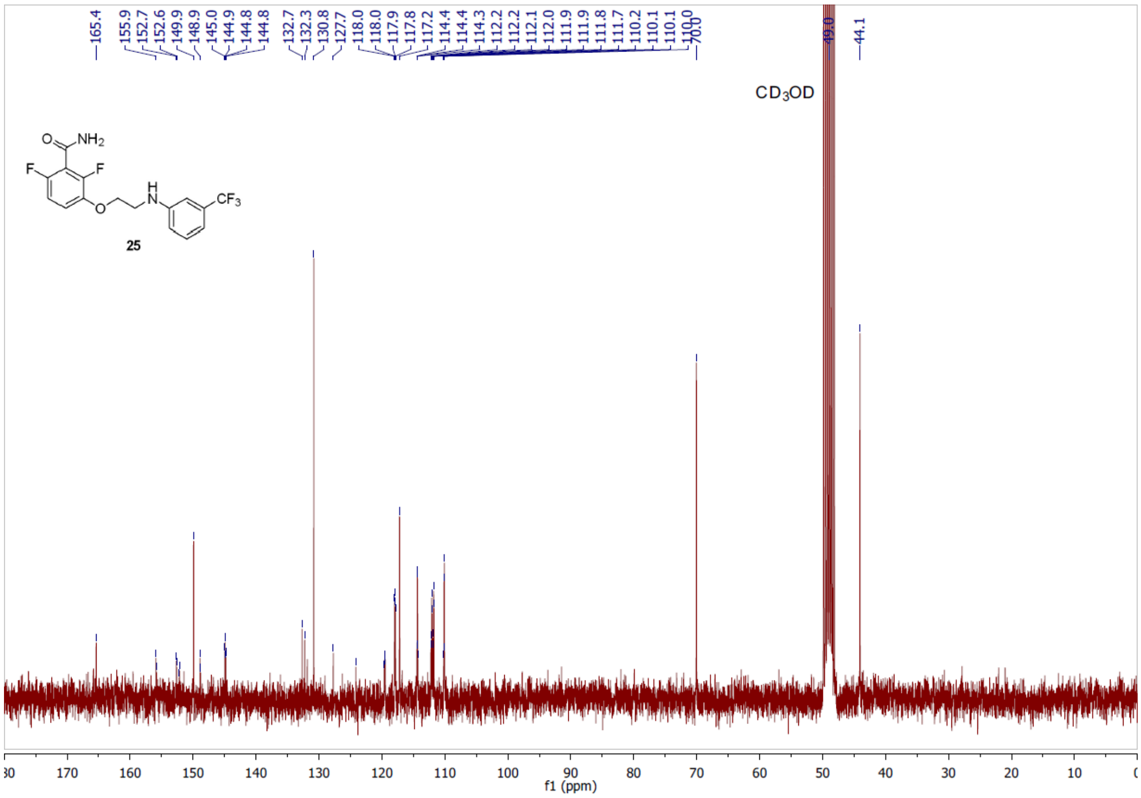

^{1}H NMR spectrum for **26** ($CDCl_3$, 300 MHz)

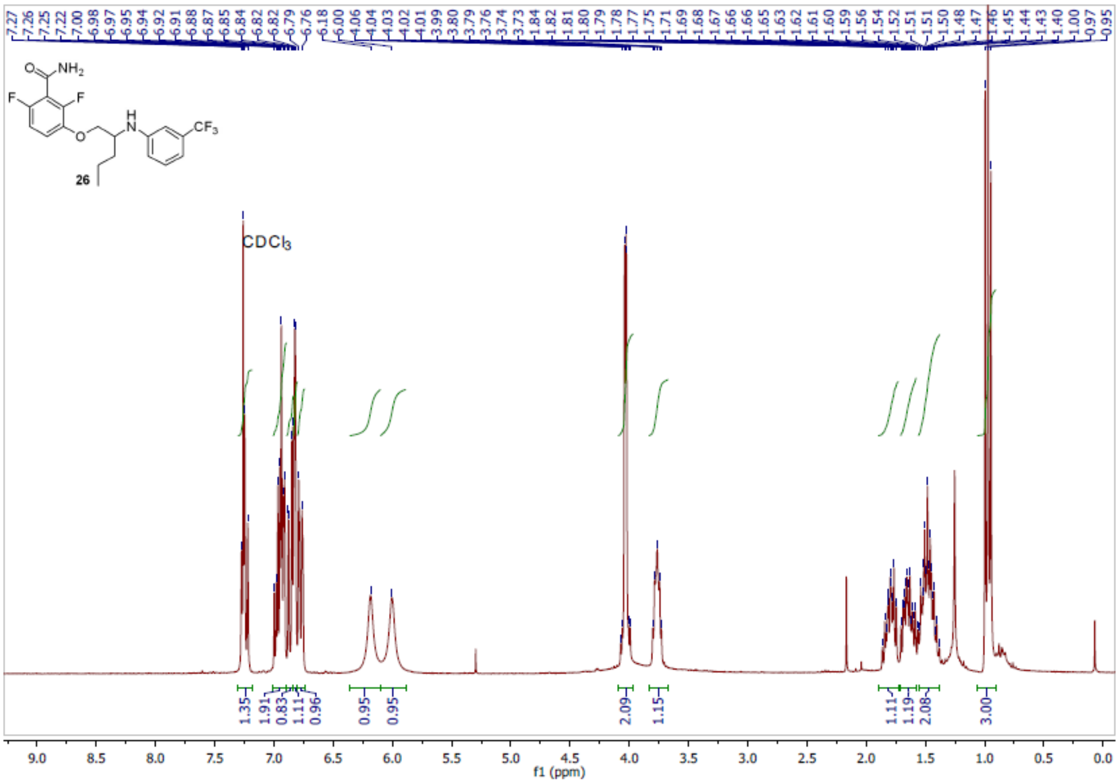

^{13}C NMR spectrum for **26** ($CDCl_3$, 75 MHz)

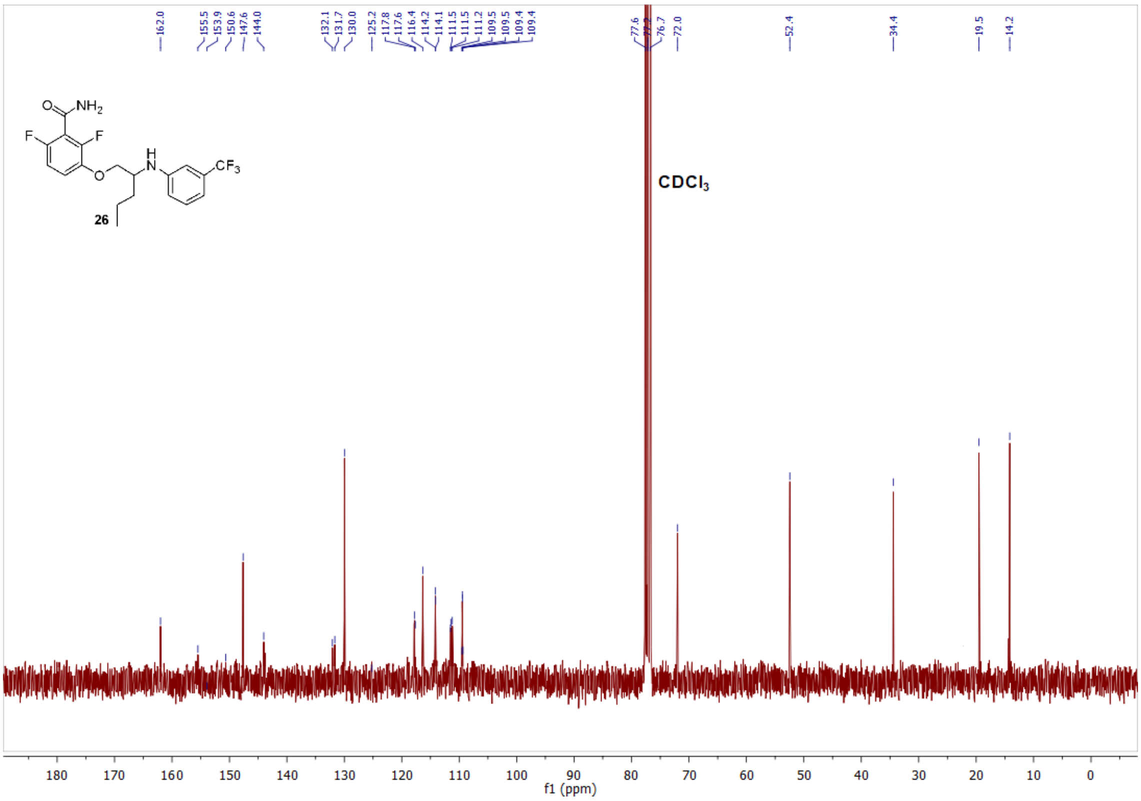

^{1}H NMR spectrum for **SP1** (CD_3OD, 700 MHz)

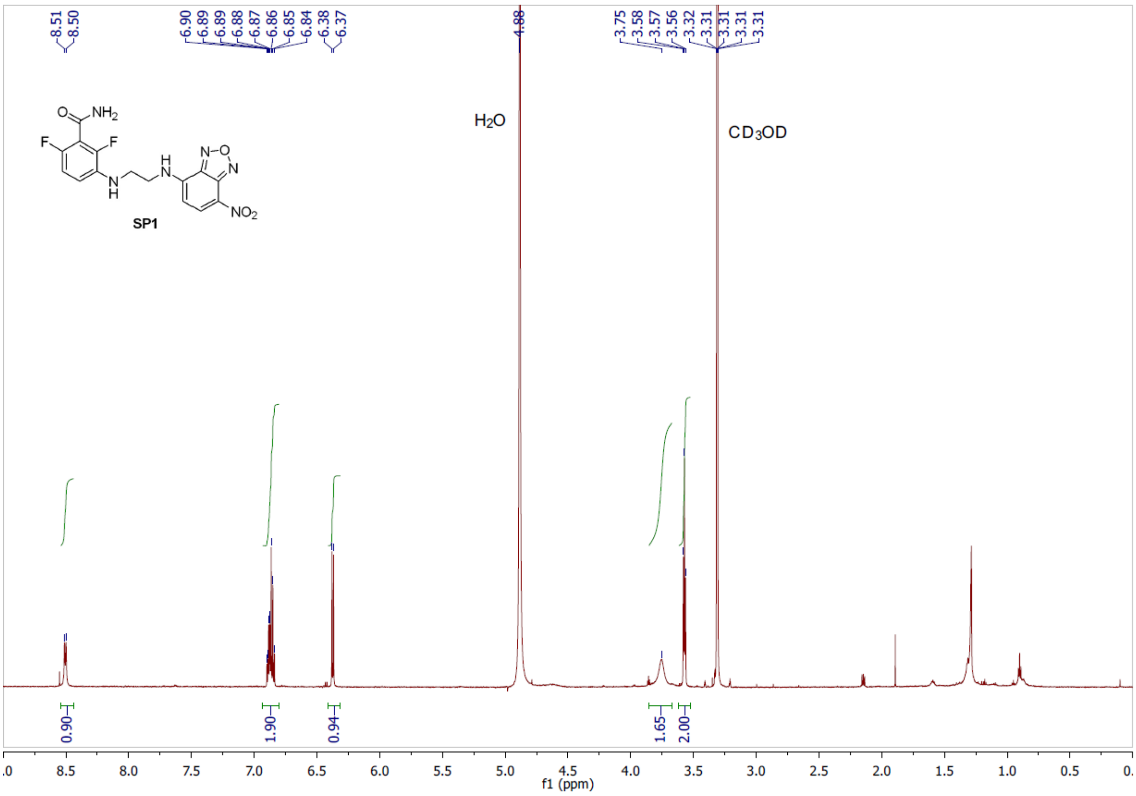

^{13}C NMR spectrum for **SP1** (CD_3OD, 175 MHz)

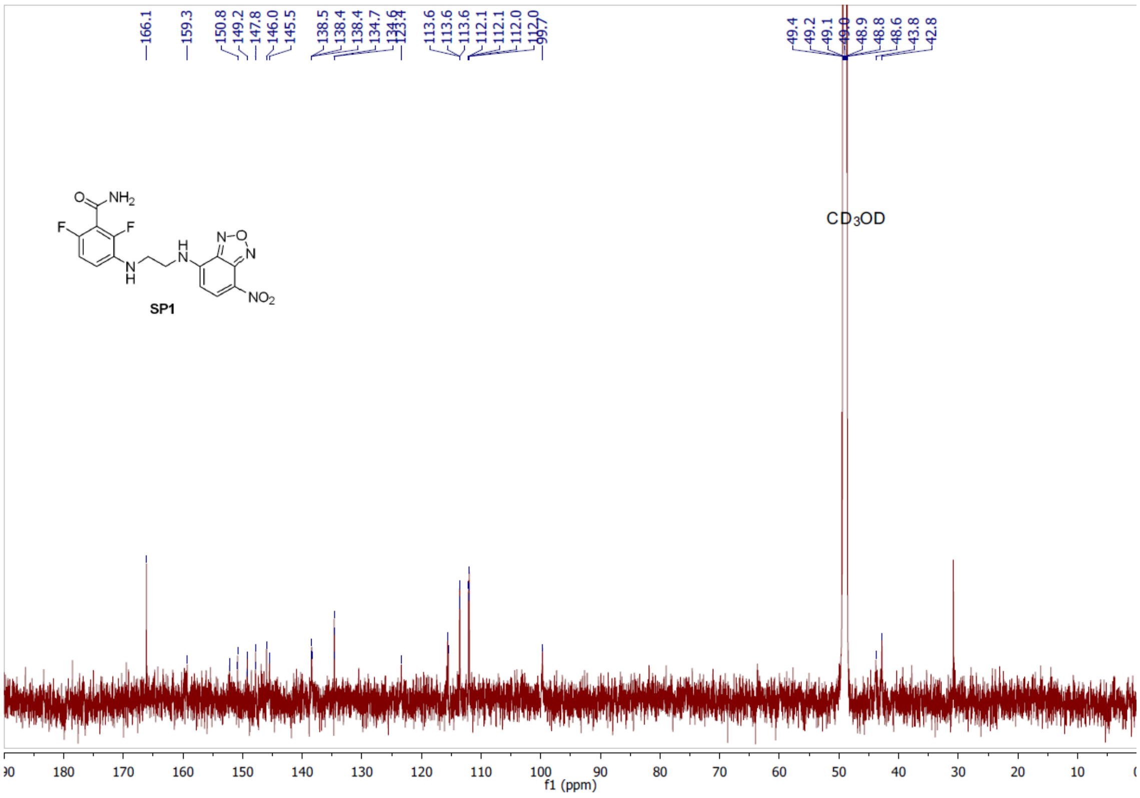

^1H NMR spectrum for **SP2** (CD_3OD, 300 MHz)

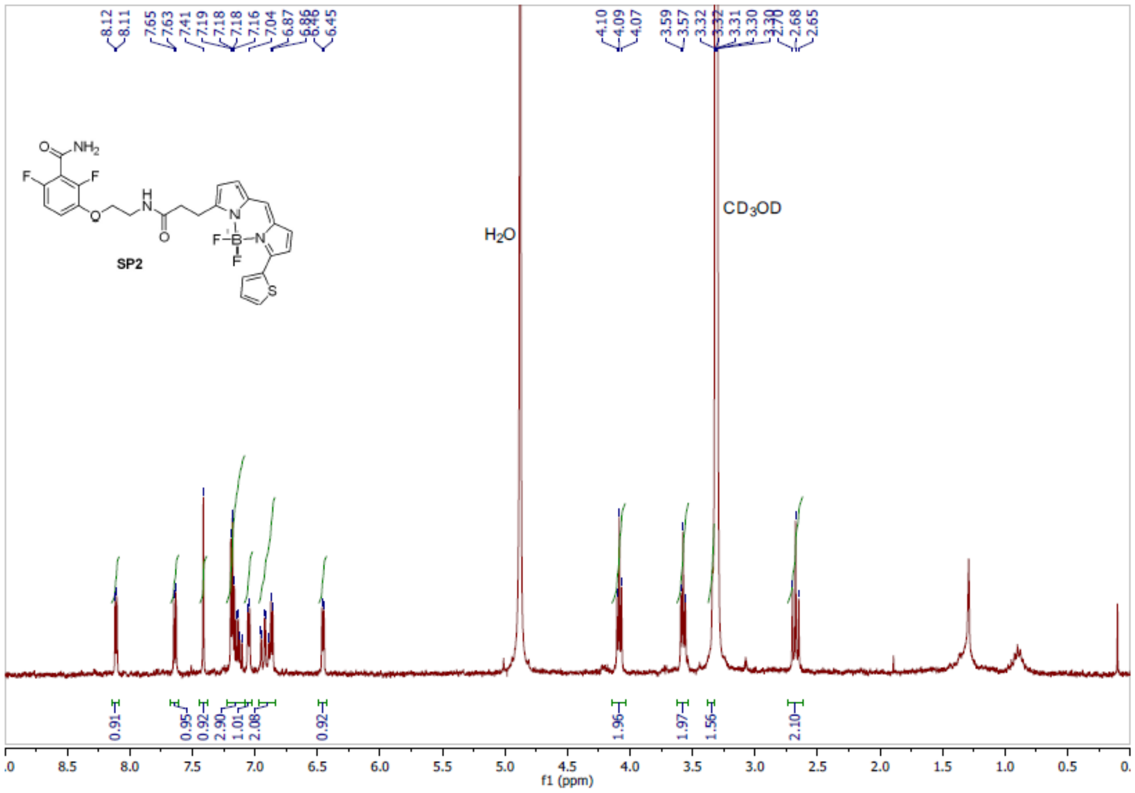

^1H NMR spectrum for **SP3** (CD_3OD, 700 MHz)

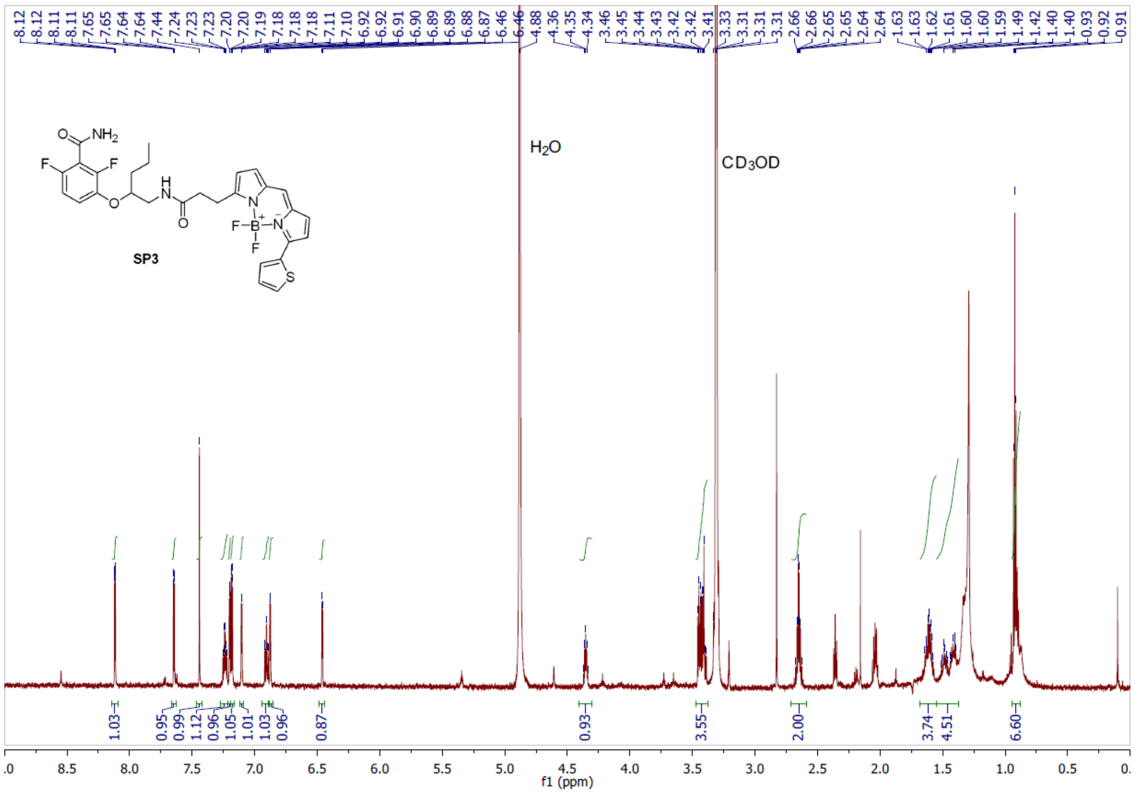

^{1}H NMR spectrum for **SP4** ($CDCl_3$, 700 MHz)

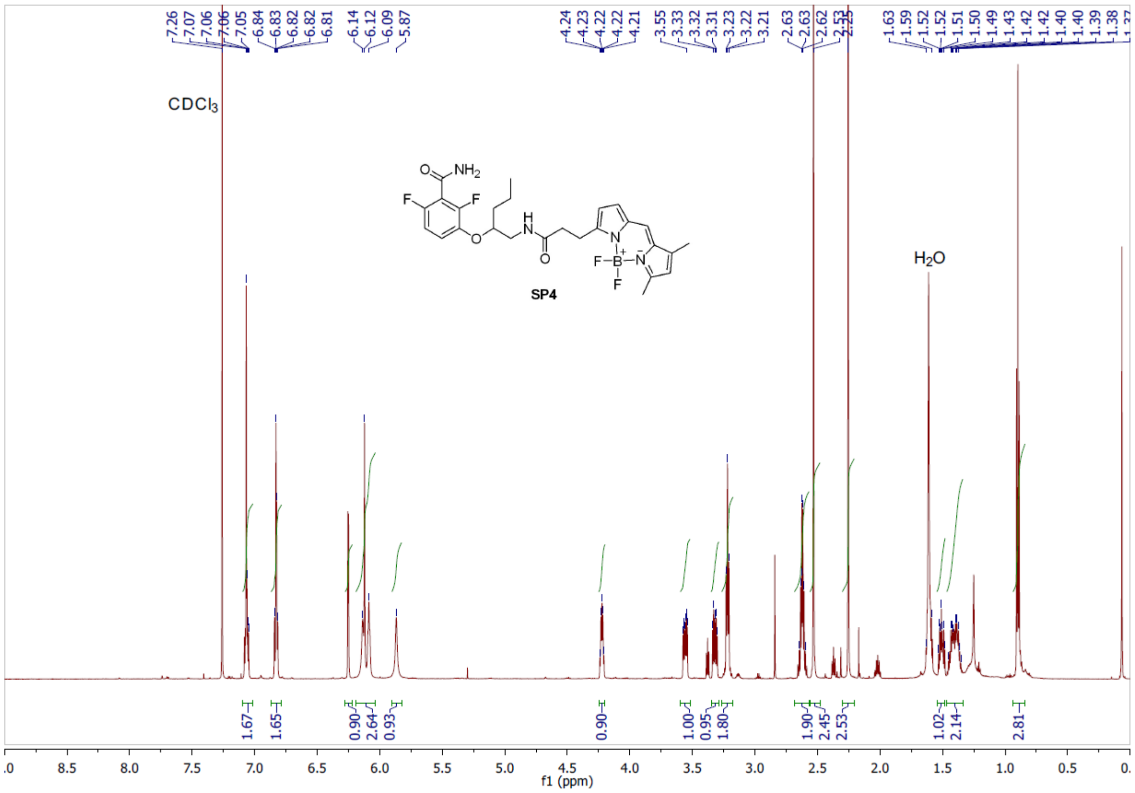

^{13}C NMR spectrum for **SP4** ($CDCl_3$, 175 MHz)

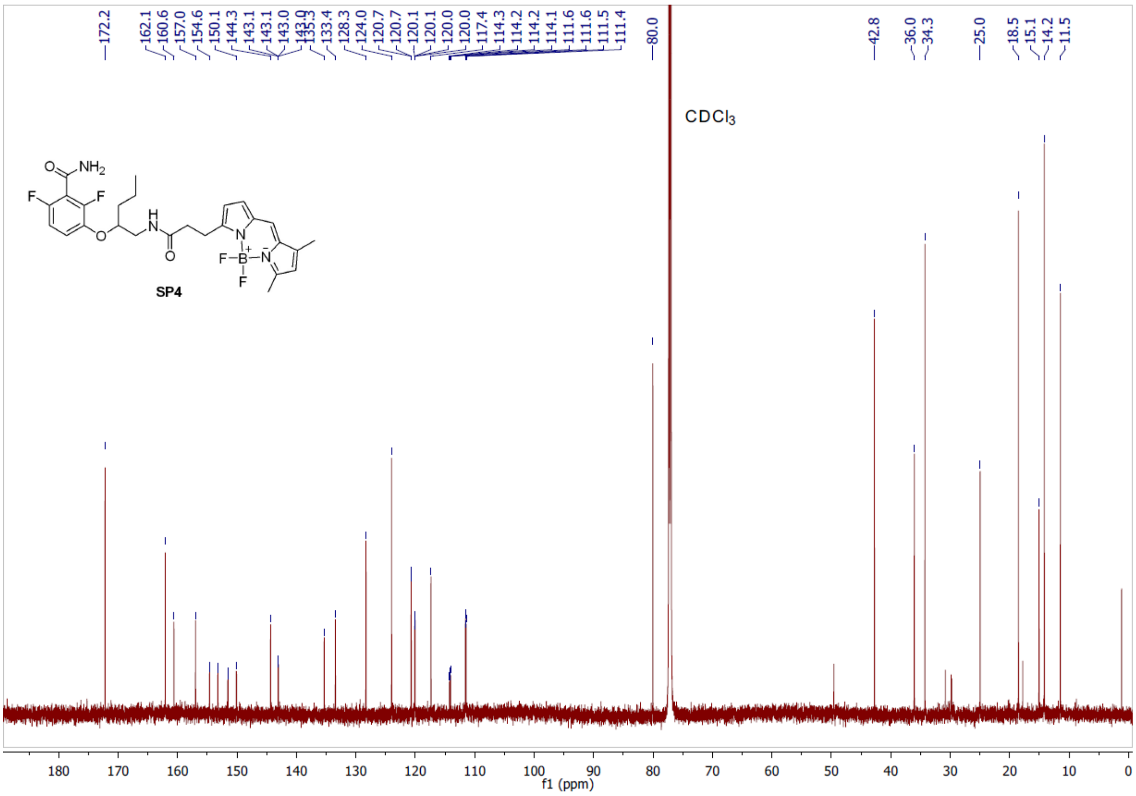

^{1}H NMR spectrum for **SP5** (CD_3OD, 700 MHz)

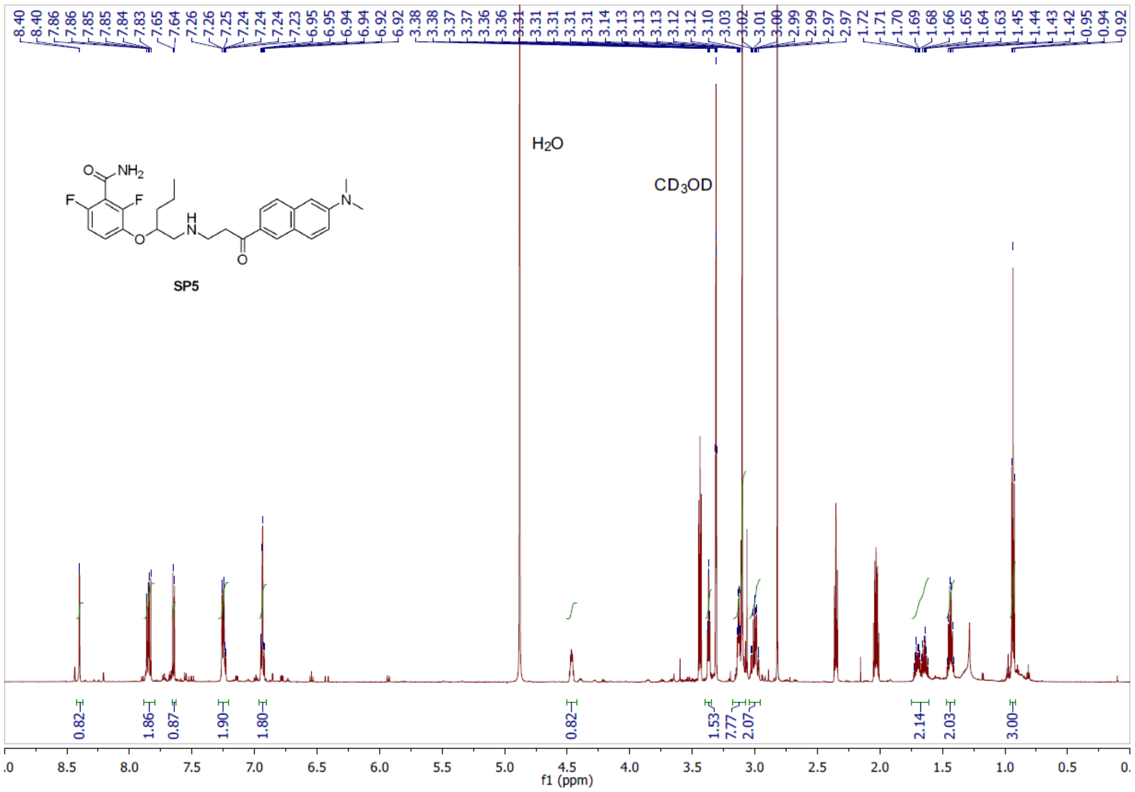

^{13}C NMR spectrum for **SP5** (CD_3OD, 175 MHz)

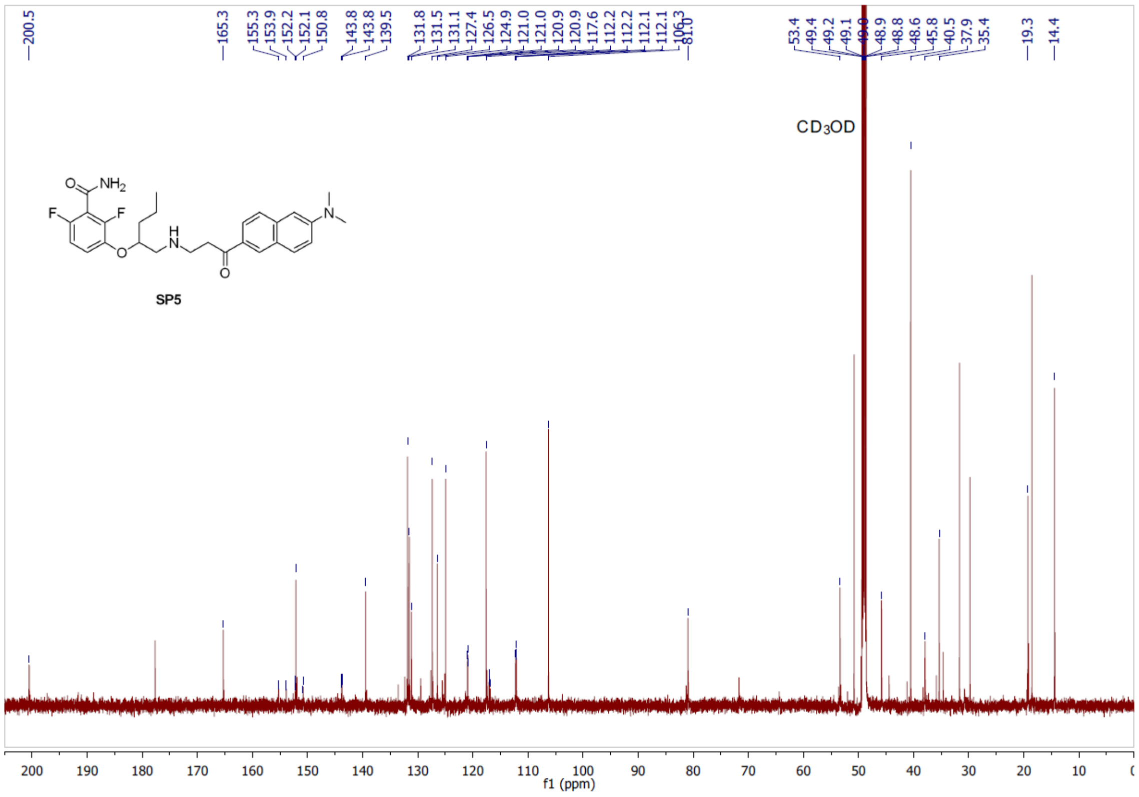

6. HPLC traces

Compound (*S*)-**2**

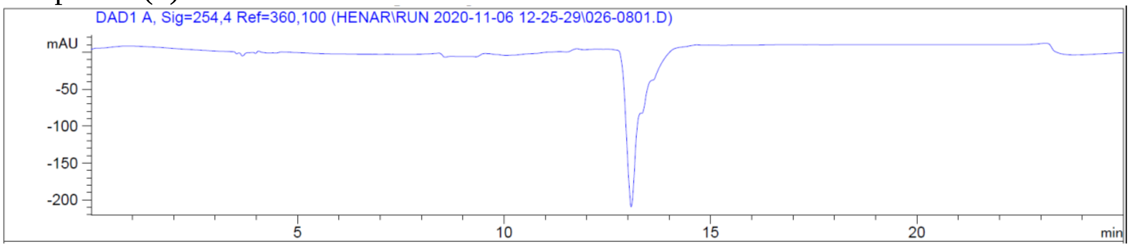

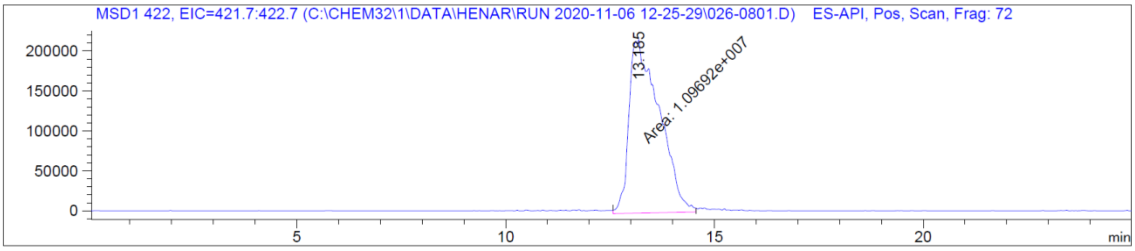

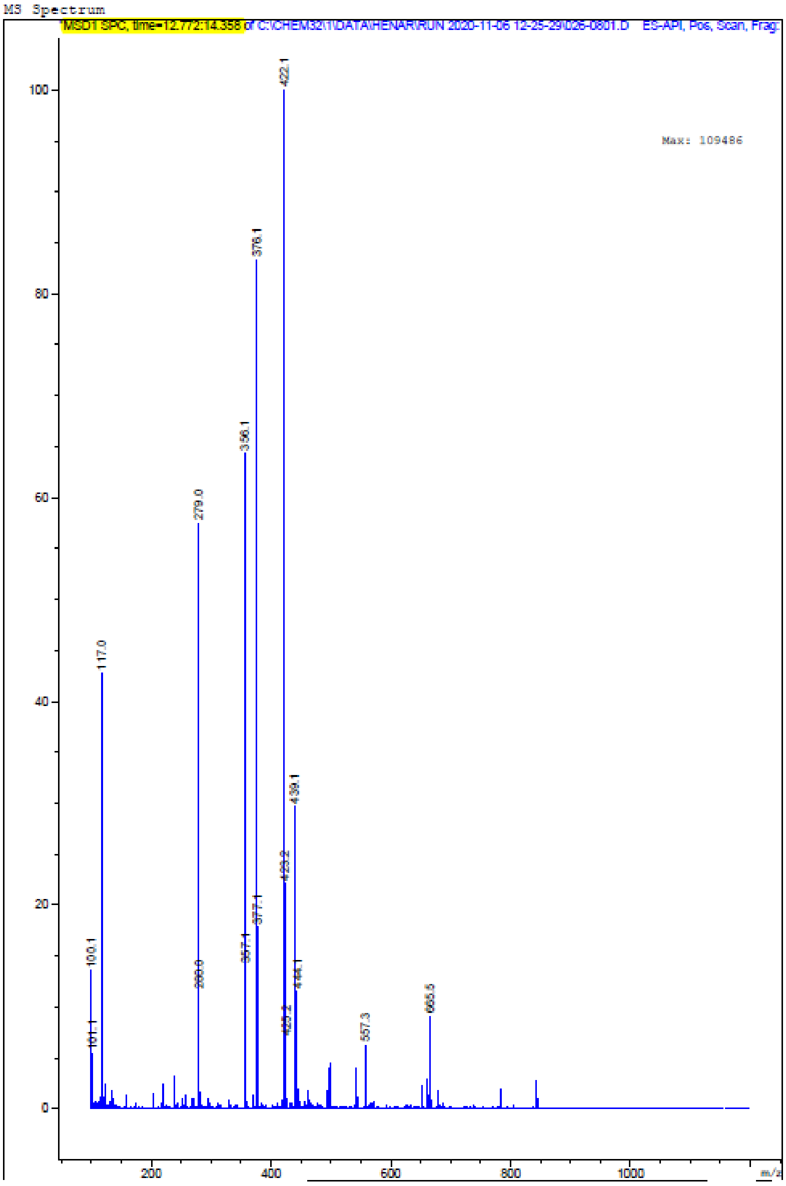

Compound (*R*)-**5**

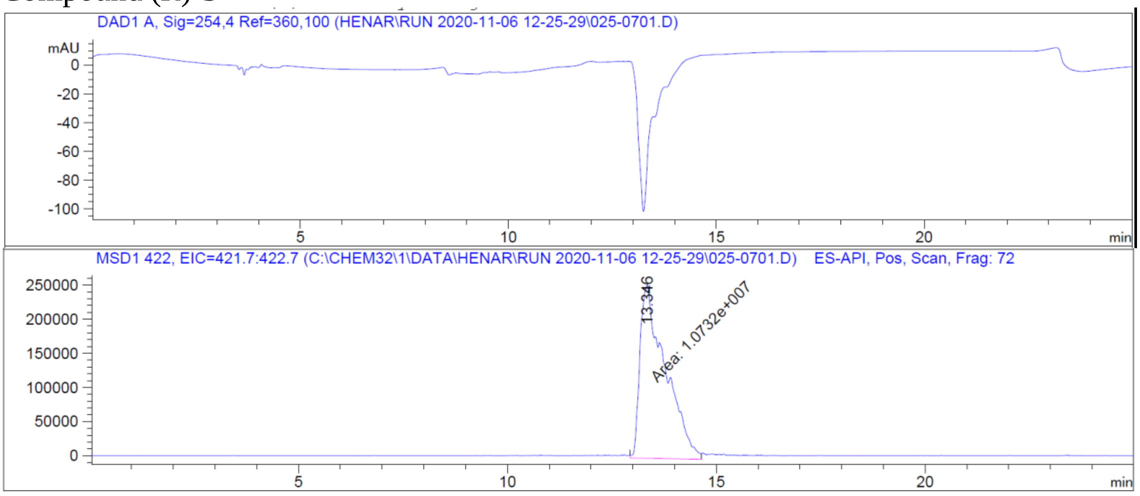

DAD1 A, Sig=254,4 Ref=360,100 (HENAR\RUN 2020-11-06 12-25-29\025-0701.D)
mAU
min
MSD1 422, EIC=421.7:422.7 (C:\CHEM32\1\DATA\HENAR\RUN 2020-11-06 12-25-29\025-0701.D) ES-API, Pos, Scan, Frag: 72
13.346
Area: 1.0732e+007
min

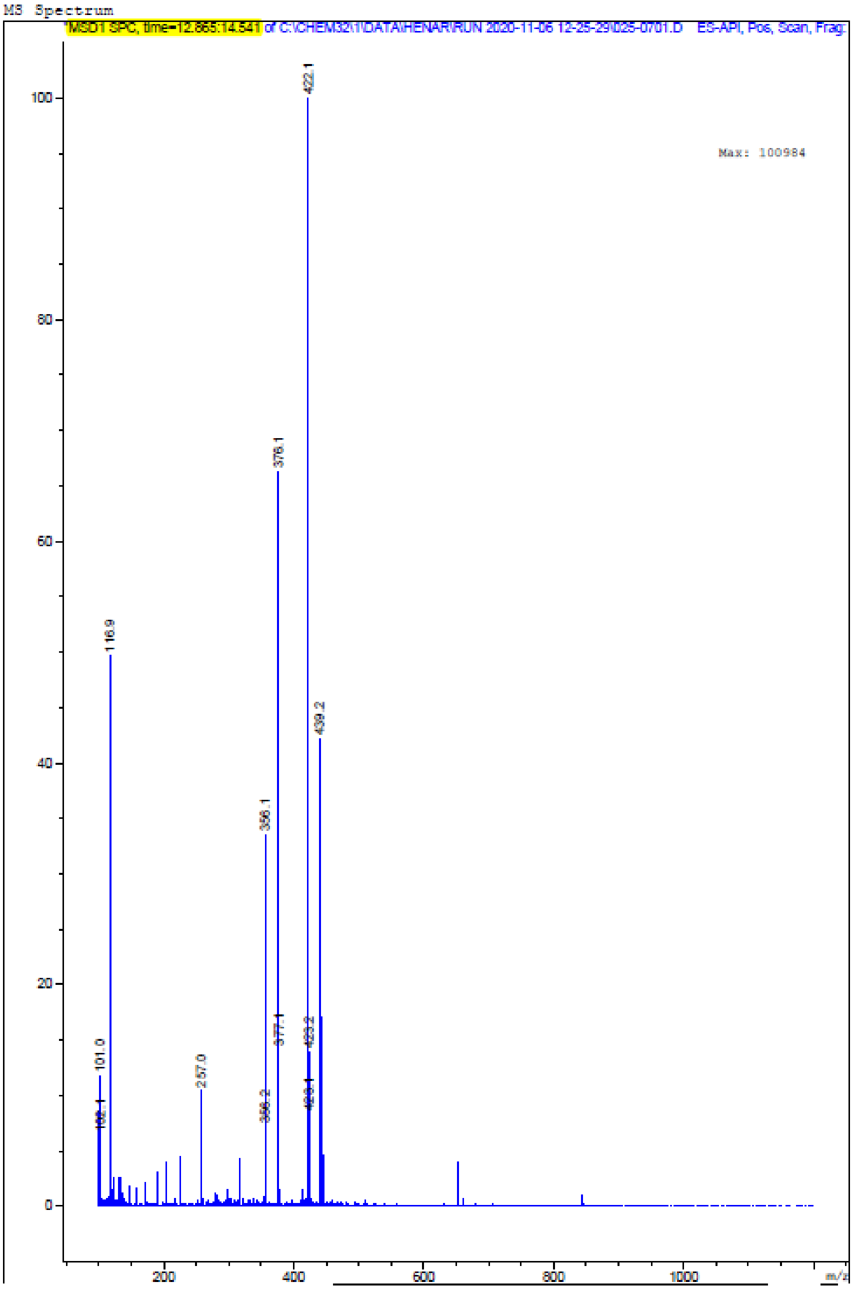

MS Spectrum
*MSD1 SPC, time=12.865:14.541 of C:\CHEM32\1\DATA\HENAR\RUN 2020-11-06 12-25-29\025-0701.D ES-API, Pos, Scan, Frag:
Max: 100984
422.1
376.1
116.9
439.2
356.1
101.0
257.0
377.1
423.2
m/z

Compound **18**

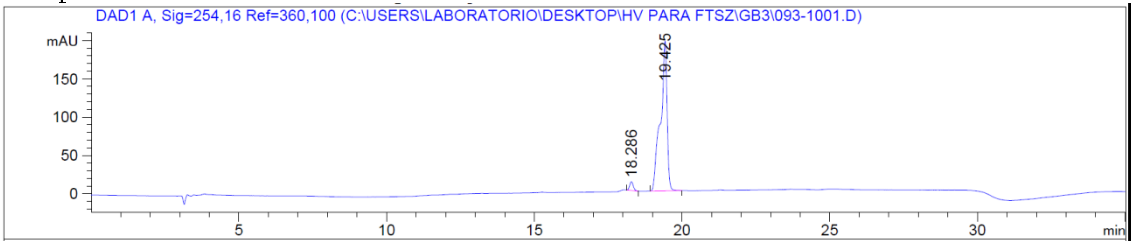


```
Signal 1: DAD1 A, Sig=254,16 Ref=360,100

Peak RetTime Type  Width      Area      Height     Area
  #   [min]        [min]    [mAU*s]     [mAU]       %
----|-------|----|-------|----------|----------|--------|
   1  18.286 BB    0.1317   98.58289   11.53026   3.1362
   2  19.425 BB    0.2157 3044.84351  195.52879  96.8638

Totals :                  3143.42640  207.05906
```

MS Spectrum

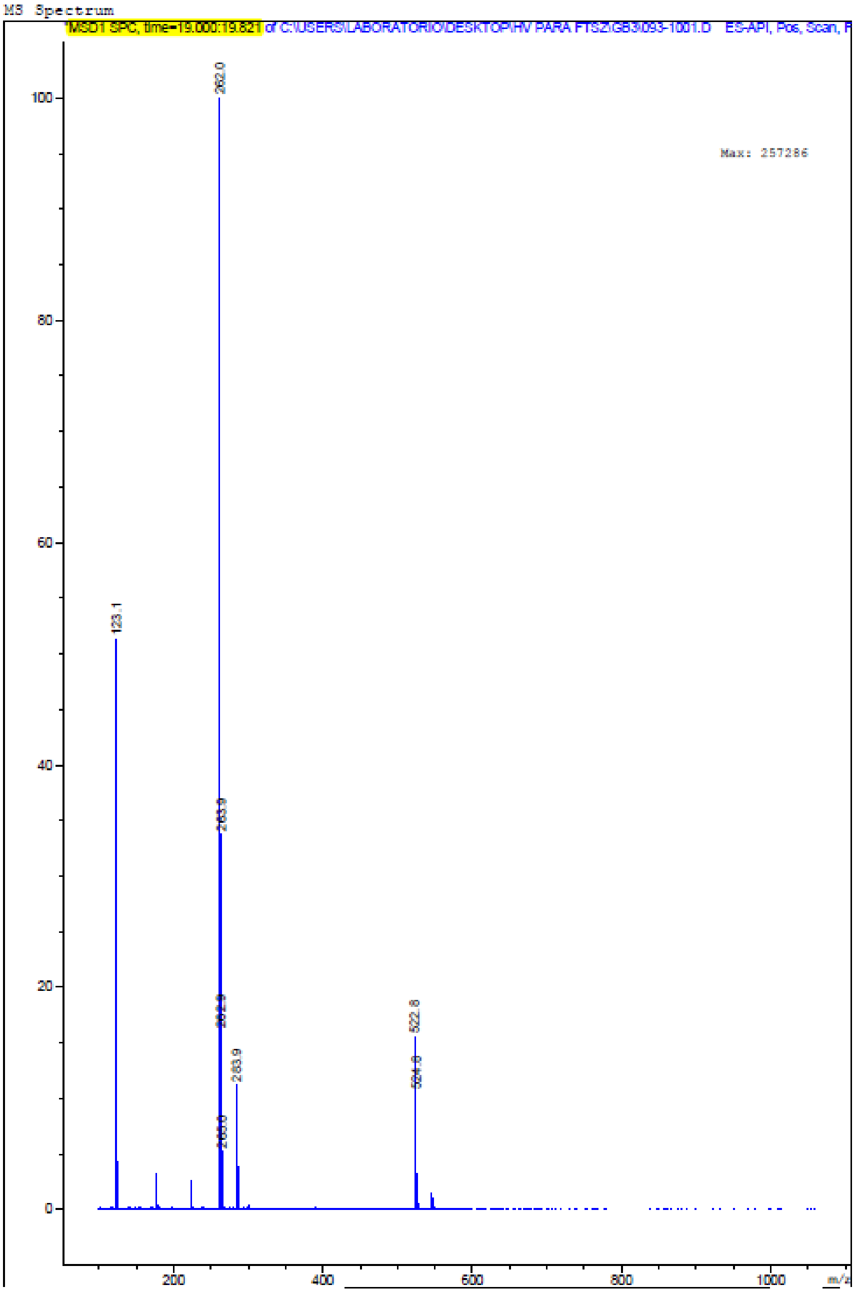

Compound **20**

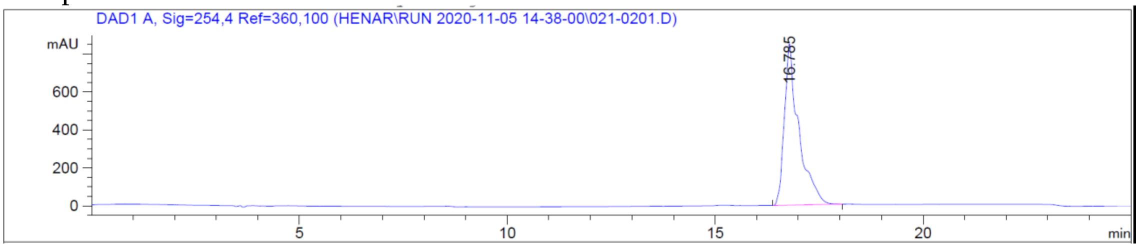
DAD1 A, Sig=254,4 Ref=360,100 (HENAR\RUN 2020-11-05 14-38-00\021-0201.D)
mAU
600
400
200
0
16.785
5
10
15
20
min

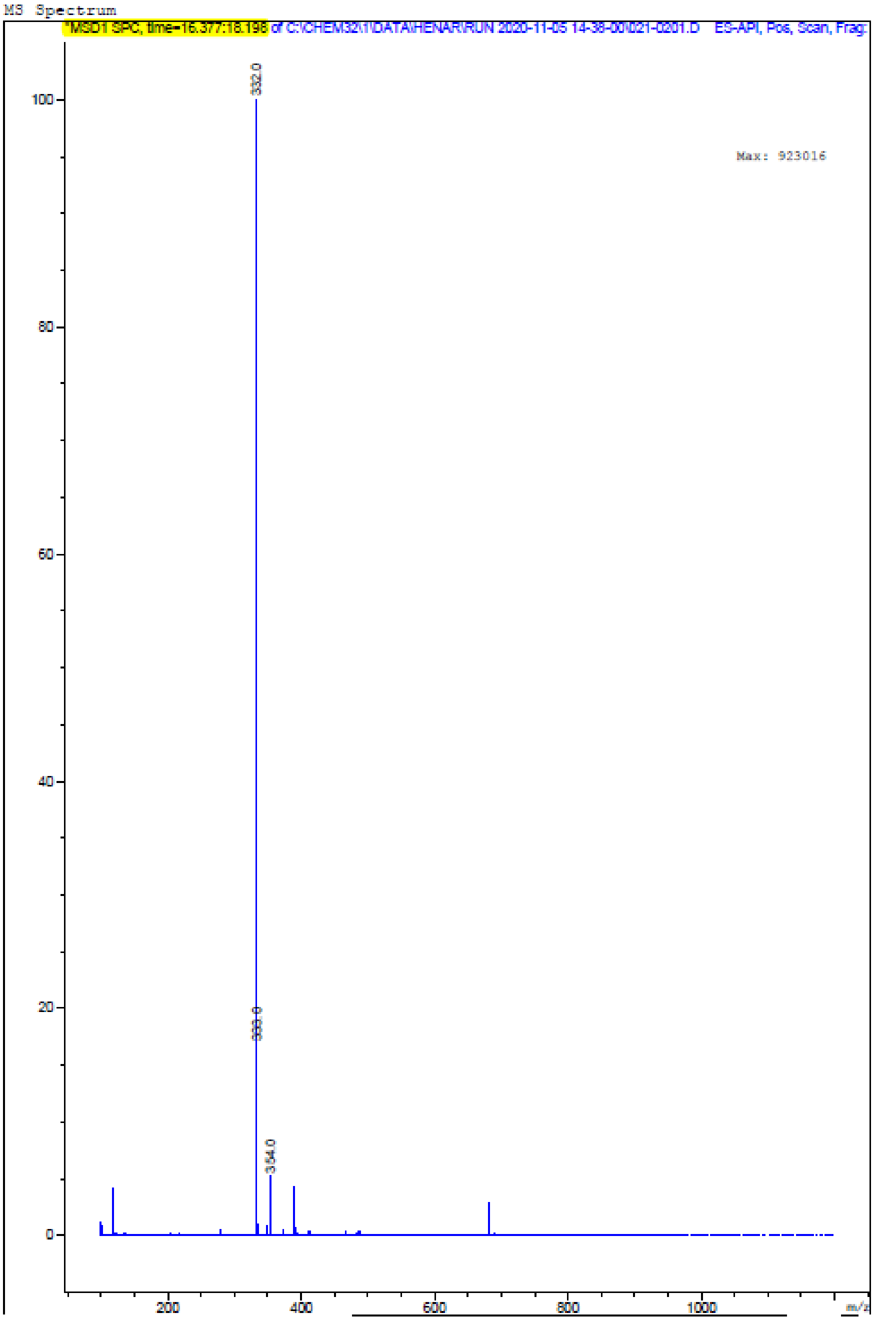
MS Spectrum
*MSD1 SPC, time=16.377:18.198 of C:\CHEM32\1\DATA\HENAR\RUN 2020-11-05 14-38-00\021-0201.D ES-API, Pos, Scan, Frag:
Max: 923016
332.0
333.0
354.0
100
80
60
40
20
0
200
400
600
800
1000
m/z

Compound **25**

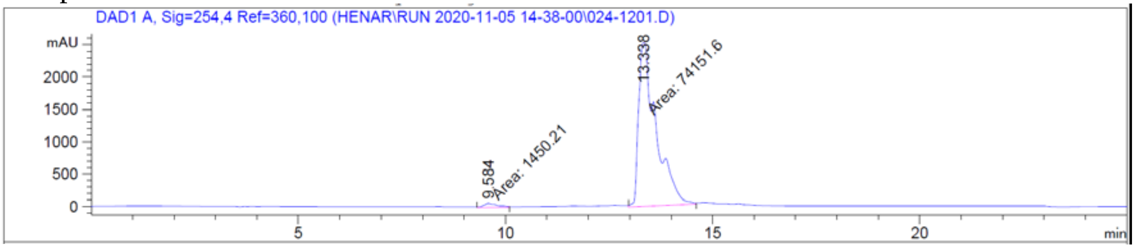

Signal 1: DAD1 A, Sig=254,4 Ref=360,100

Peak #	RetTime [min]	Type	Width [min]	Area [mAU*s]	Height [mAU]	Area %
1	9.584	MM	0.3743	1450.21472	64.57121	1.9182
2	13.338	MM	0.4884	7.41516e4	2530.22388	98.0818

Totals : 7.56018e4 2594.79508

MS Spectrum

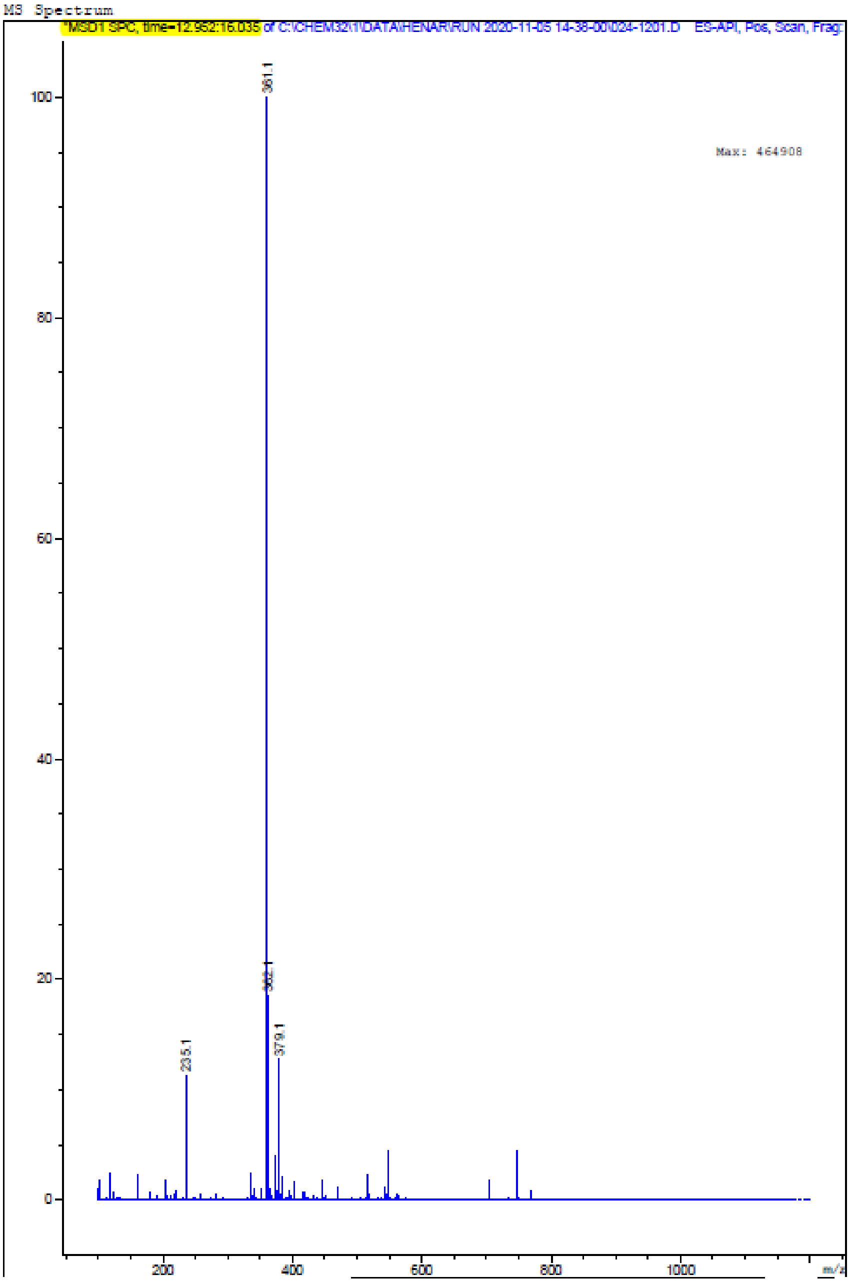

Compound (*R*)-**26**

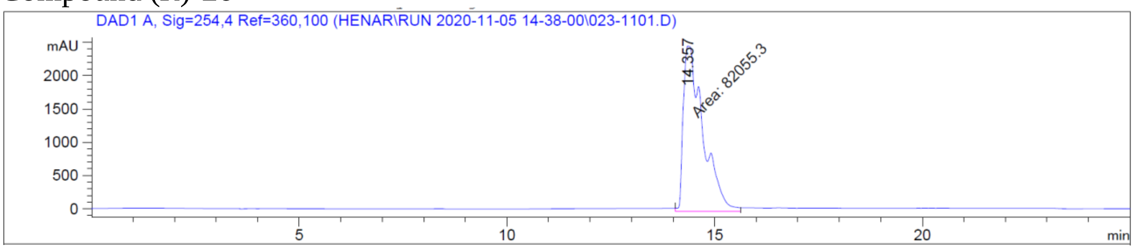

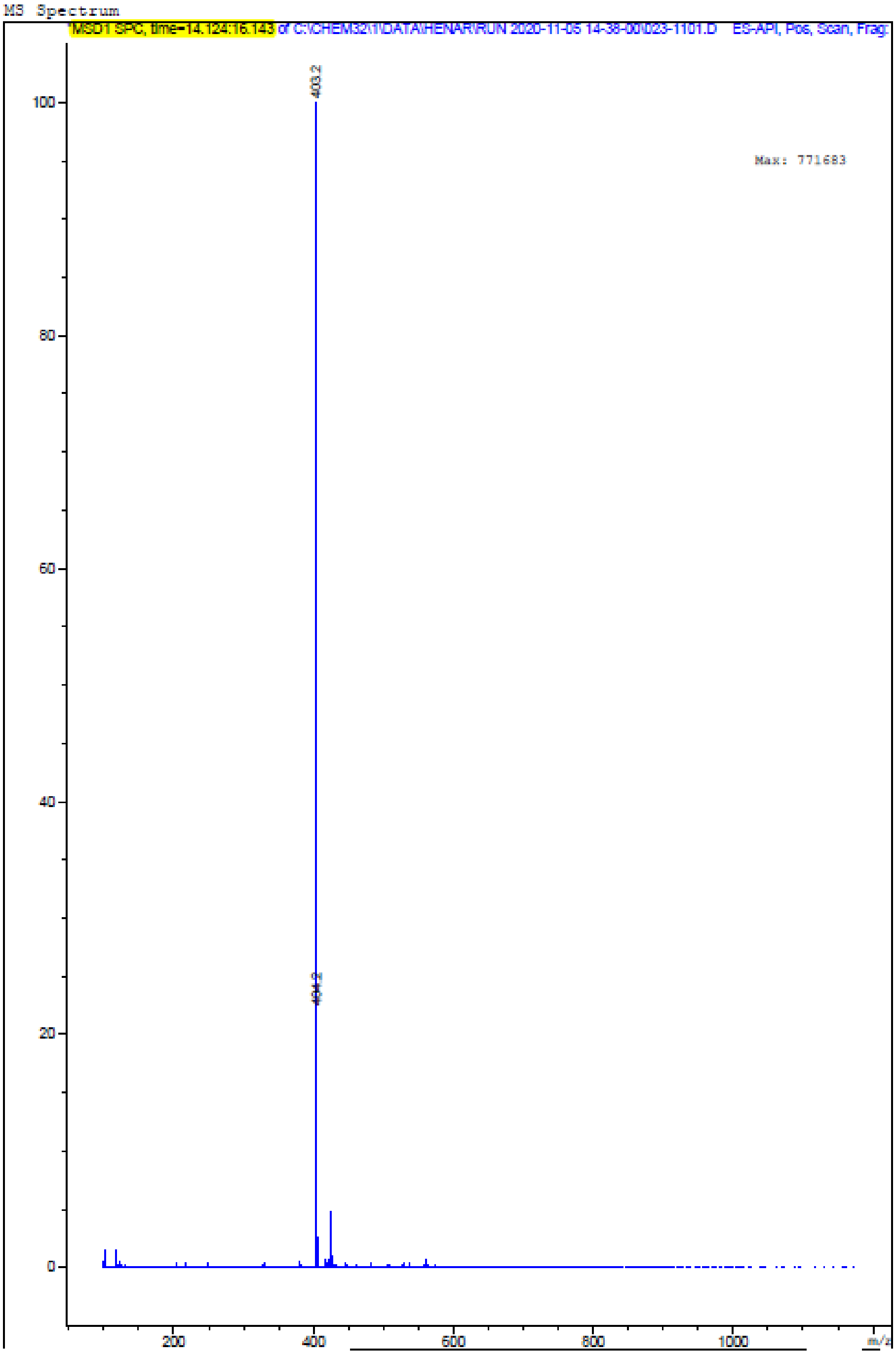

Supplement: Supplementary file 1 — jm0c02207_si_001.pdf [file jm0c02207_si_001.pdf]
